# HIV-1 T cell epitopes targeted to Rhesus macaque CD40 and DCIR: A comparative study of prototype dendritic cell targeting therapeutic vaccine candidates

**DOI:** 10.1371/journal.pone.0207794

**Published:** 2018-11-30

**Authors:** Anne-Laure Flamar, Henri Bonnabau, Sandra Zurawski, Christine Lacabaratz, Monica Montes, Laura Richert, Aurelie Wiedemann, Lindsey Galmin, Deborah Weiss, Anthony Cristillo, Lauren Hudacik, Andres Salazar, Cécile Peltekian, Rodolphe Thiebaut, Gerard Zurawski, Yves Levy

**Affiliations:** 1 Vaccine Research Institute, Université Paris-Est, Faculté de Médecine, INSERM U955, Créteil, France; 2 Baylor Institute for Immunology Research and INSERM U955, Dallas, Texas, United States of America; 3 Inserm, Bordeaux Population Health Research Center, UMR 1219, Inria SISTM, Université Bordeaux, ISPED, Bordeaux, France; 4 Assistance Publique-Hôpitaux de Paris, Groupe Henri-Mondor Albert-Chenevier, Service D’immunologie Clinique, Créteil, France; 5 Advanced BioScience Laboratories, Inc., Rockville, MD, United States of America; 6 Oncovir, Washington DC, United States of America; University of Arizona College of Medicine, UNITED STATES

## Abstract

HIV-1 infection can be controlled by anti-retroviral drug therapy, but this is a lifetime treatment and the virus remains latent and rapidly rebounds if therapy is stopped. HIV-1-infected individuals under this drug regimen have increased rates of cancers, cardiovascular diseases, and autoimmunity due to compromised immunity. A therapeutic vaccine boosting cellular immunity against HIV-1 is therefore desirable and, possibly combined with other immune modulating agents, could obviate the need for long-term drug therapies. An approach to elicit strong T cell-based immunity is to direct virus protein antigens specifically to dendritic cells (DCs), which are the key cell type for controlling immune responses. For eliciting therapeutic cellular immunity in HIV-1-infected individuals, we developed vaccines comprised of five T cell epitope-rich regions of HIV-1 Gag, Nef, and Pol (HIV5pep) fused to monoclonal antibodies that bind either, the antigen presenting cell activating receptor CD40, or the endocytic dendritic cell immunoreceptor DCIR. The study aimed to demonstrate vaccine safety, establish efficacy for broad T cell responses in both primed and naïve settings, and identify one candidate vaccine for human therapeutic development. The vaccines were administered to Rhesus macaques by intradermal injection with poly-ICLC adjuvant. The animals were either i) naïve or, ii) previously primed with modified vaccinia Ankara vector (MVA) encoding HIV-1 Gag, Pol, and Nef (MVA GagPolNef). In the MVA-primed groups, both DC-targeting vaccinations boosted HIV5pep-specific blood CD4^+^ T cells producing multiple cytokines, but did not affect the MVA-elicited CD8^+^ T cell responses. In the naive groups, both DC-targeting vaccines elicited antigen-specific polyfunctional CD4^+^ and CD8^+^ T cell responses to multiple epitopes and these responses were unchanged by a subsequent MVA GagPolNef boost. In both settings, the T cell responses elicited via the CD40-targeting vaccine were more robust and were detectable in all the animals, favoring further development of the CD40-targeting vaccine for therapeutic vaccination of HIV-1-infected individuals.

## Introduction

Elite controllers, a subpopulation of HIV-1-infected individuals, suppress viral replication mainly by mounting robust cellular T cell responses [1, 2]. This has raised the hope of evoking functional remission of HIV-1 infection by mimicking similar responses in chronically infected individuals. Combination anti-retroviral therapy (cART) prevents progression to disease in chronically infected individuals but is a lifetime treatment since the virus remains latent and rapidly rebounds if therapy is discontinued [3]. Furthermore, cART often does not restore functional immunity and such individuals have increased susceptibility to cancers and cardiovascular diseases [4]. Thus, there remains a need for therapeutic vaccination strategies, possibly combined with immune modulating agents, such as interleukin-7 [5], type 1 interferon [6], or programmed cell death protein 1 inhibitors [7], for releasing the latent virus reservoir to attain a functional cure and restore immunity [8, 9].

Dendritic cells play a central role in the immune response [10]. Vaccination based on targeting antigens to dendritic cells (DC-targeting) by fusing antigens to antibodies binding to internalizing DC receptors is a strategy being investigated over the past decade to enhance protein immunogenicity [10, 11, 12, 13]. DC-targeting encompasses a plethora of variables including: specific targeted receptor and/or DC subtype; targeting antibody isotype and/or configuration; intrinsic biological action of the antibody; characteristics of the antigen; requirement for co-administered adjuvant; utility for priming immunity vs. boosting immune memory; dose and regimen; route of administration; and manufacturability for clinical application. Comparative studies via animal *in vivo* models or human *in vitro* culture systems are still needed to address these important questions. To date the first human clinical studies are cancer antigens or HIV-1 Gag p24 directed to the mannose receptor or DEC-205 [14, 15], (ClinicalTrials.gov Identifiers NCT00648102 and NCT01127464).

In non-human primates (NHPs), targeting HIV-1 Gag p24 to various DC receptors including DEC-205 [16], Langerin [17], and DCIR [18] has demonstrated increased antigenicity, as well as the benefits of co-administration with poly-IC adjuvant and the utility for priming immune responses. In other studies, targeting HIV-1 Env gp140 to LOX-1 [19] and CD40 [20] evoked potentially protective humoral and cellular immunity in both priming and viral vector boost settings. To broaden the antigenic repertoire beyond HIV-1 Gag p24 and the highly variable Env gp140, we previously tested in NHPs and humans a set of five lipidated T cell epitope-rich peptides from HIV-1 Gag, Pol, and Nef (HIV5pep) that induced HIV-1-specific CD4^+^ and CD8^+^ T cell responses [21–25]. Also, vaccinating HV-1-infected individuals with autologous DCs loaded with these peptides demonstrated their potential for controlling HIV-1 rebound after cART interruption [26].

When fused to a monoclonal antibody that binds CD40, an activating antigen-presenting cell (APC) receptor [27], these five HIV-1 peptides efficiently expanded *in vitro* multifunctional CD4^+^ and CD8^+^ memory T cells specific to a variety of HIV-1 epitopes across diverse haplotypes of HIV-1-infected individuals [28]. CD40-targeting has shown superiority for evoking antigen-specific CD8^+^ T cell responses *in vitro* over targeting other receptors, including DCIR and LOX-1 [29]. However, direct comparisons of DC-targeting vehicles in NHP models are critical for selecting human clinical development candidates. DCIR, a C-type lectin surface receptor broadly expressed including on human DCs [30] has shown promise for cross-priming CD8^+^ T cells *in vitro* cultures [31] and is safe and efficacious for evoking humoral immunity in NHPs [18]. Thus, in this study we compare targeting of HIV5pep to either CD40 or DCIR for eliciting T cell responses in both naïve and viral vector-primed primed Rhesus macaques as a criterion to select the best vaccine candidate to progress towards clinical application.

## Results

### DC-targeting vaccines boost T cell responses in MVA-primed Rhesus macaques and elicit T cell responses in naïve animals

**Fig 1:**
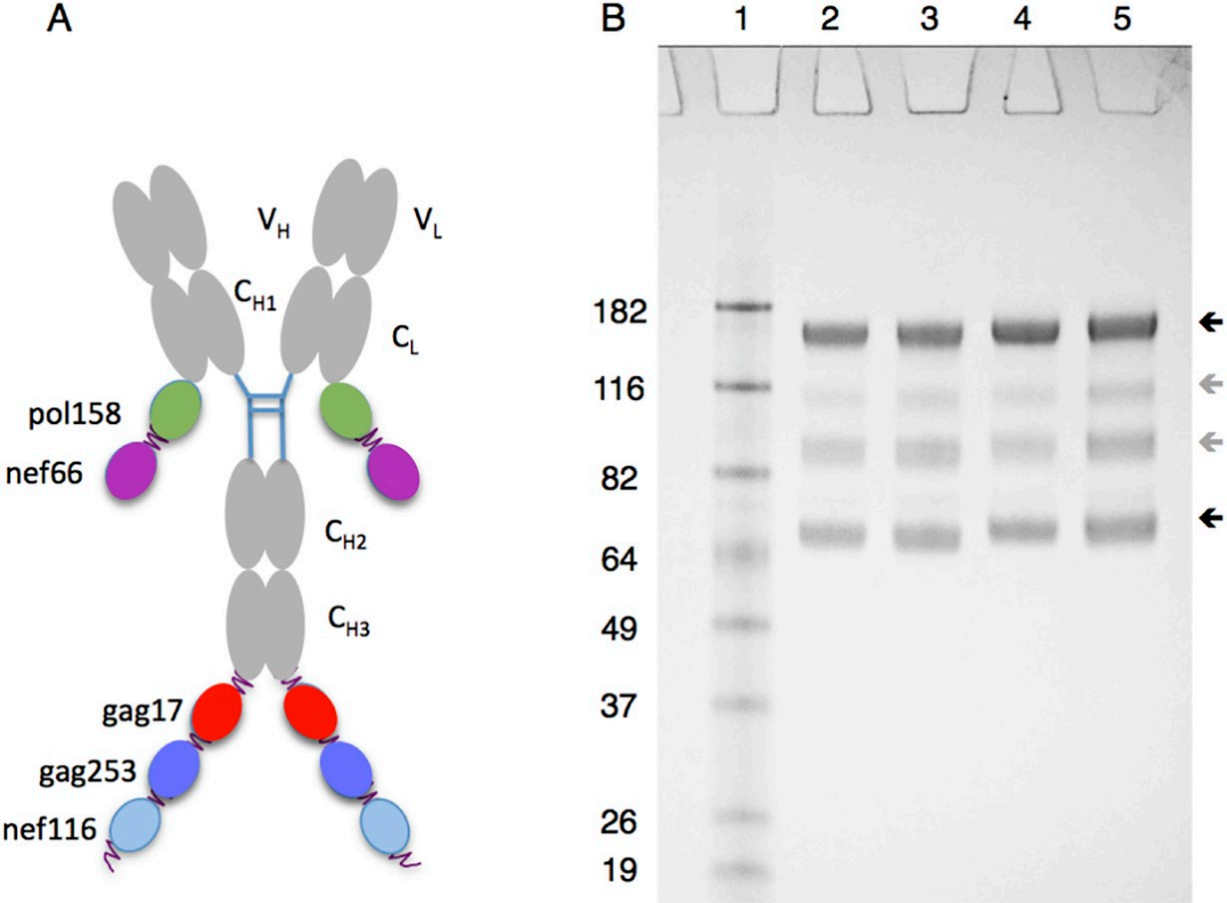
Overview of structure and biochemical analysis of αDCIR.HIV5pep and αCD40.HIV5pep vaccines. (A) Cartoon of the five HIV-1 peptide regions fused to the C-termini of either the heavy chain C_H_3 or light chain C_L_ regions of the targeting antibody and short flexible glycosylated peptide linkers are indicated as squiggles. V_L_ and V_H_ indicate, respectively, the L and H chain variable regions. (B) Reducing SDS PAGE analysis of αCD40.HIV5pep (Lanes 2, 3) and αDCIR.HIV5pep (Lanes 4, 5) vaccines. The dark upper arrow on the right indicates the position of the full heavy chains and the dark lower arrow indicates the position of light chains. The lighter grey arrows in between indicate minor breakdown or glycosylation variant products of the heavy chain. Molecular weight markers are shown in Lane 1 with mass (kDa) indicated beside each protein. Staining was with Coomassie Brilliant Blue.

We previously fused five T cell epitope-rich regions from HIV-1 Gag, Pol, and Nef to a chimeric mouse variable region-human IgG4 anti-human CD40 antibody [28]. For this present study the mouse variable regions were humanized to reduce potential antigenicity in humans and we relocated two of the HIV5pep regions onto the light chain C-terminus to improve yield and solubility (Fig 1A). Similar constructs were made for the humanized variable region form of an anti-human DCIR monoclonal antibody [18, 31]. For quality assurance, the prototype vaccines were appraised by reducing SDS PAGE analysis (Fig 1B), and were confirmed to bind their target human and Rhesus macaque receptors with affinities close to their parental counterparts not fused to the HIV5pep sequences (see Methods). These humanized prototype vaccines that we termed, respectively, αCD40.HIV5pep and αDCIR.HIV5pep, had efficacies similar to the chimeric forms with the five peptide regions grafted to the H-chain C-termini in expanding memory T cells specific to epitopes from all five HIV-1 peptide regions (see Methods) and elicited the same range of epitope responses (S1 Fig) in HIV-1-infected individual peripheral blood mononuclear cell (PBMC) *in vitro* cultures. To test immunogenicity of these two vaccines, two Rhesus macaques groups (G1 and G2) were primed twice with an attenuated poxvirus Modified Vaccinia Ankara (MVA) carrying HIV-1 genes Gag, Pol, Nef (MVA GagPolNef), followed by three administrations of each DC-targeting HIV5pep vaccine co-administered with poly-ICLC, an adjuvant that activates Toll-like receptor 3 [32]. Two other groups (G3 and G4) were injected three times at monthly intervals with each DC-targeting vaccine co-administered with poly-ICLC, followed by a boost with MVA GagPolNef. Groups are referred to as G1 MVA αDCIR, G2 MVA αCD40, G3 αDCIR MVA, and G4 αCD40 MVA (Table 1).

**Table 1:** Study design for testing immunogenicity of αDCIR.HIV5pep and αCD40.HIV5pep vaccines.

	**W0**	**W2**	**W8**	**W10**	**W12**	**W14**	**W16**	**W18**	**W24**	**W26**
**G1**	**MVA**		**MVA**		**αDCIR.HIV5pep** **⁄ poly-ICLC**		**αDCIR.HIV5pep** **⁄ poly-ICLC**		**αDCIR.HIV5pep** **⁄ poly-ICLC**	
**G2**	**MVA**		**MVA**		**αCD40.HIV5pep** **⁄ poly-ICLC**		**αCD40.HIV5pep** **⁄ poly-ICLC**		**αCD40.HIV5pep** **⁄ poly-ICLC**	
	**W0**			**W2**	**W4**	**W6**	**W12**	**W14**	**W22**	**W24**
**G3**	**αDCIR.HIV5pep** **⁄ poly-ICLC**				**αDCIR.HIV5pep** **⁄ poly-ICLC**		**αDCIR.HIV5pep** **⁄ poly-ICLC**		**MVA**	
**G4**	**αCD40.HIV5pep** **⁄ poly-ICLC**				**αCD40.HIV5pep** **⁄ poly-ICLC**		**αCD40.HIV5pep** **⁄ poly-ICLC**		**MVA**	

The table shows immunization regimens used within the four groups (G1-4) in this study each group had six animals. In G1-2, MVA GagPolNef (4.5x10^7^ pfu per animal) was administered subcutaneously (s.c.). The DC-targeting vaccines (250 μg per animal at each time point) were administered intradermally (i.d.). Poly-ICLC co-administration (1 mg per animal, s.c.) was adjacent to the site of DC-targeting vaccine administration. MVA = MVA GagPolNef. Samplings for fresh and frozen peripheral blood mononuclear cells (PBMC) and plasma were at the weeks (W) indicated by shading. Note the timelines for G1-2 are different from G3-4.

**Fig 2:**
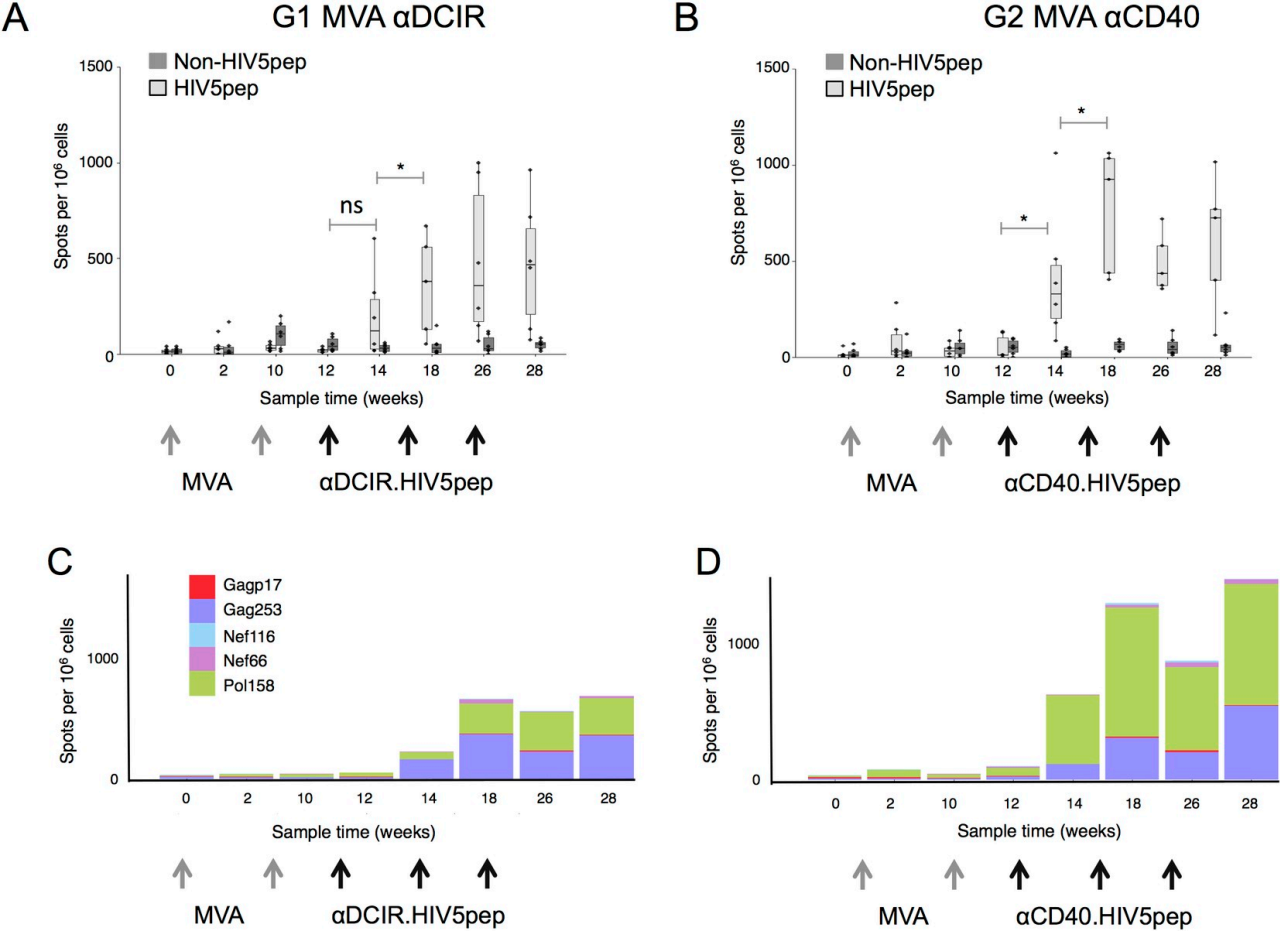
T cell responses to αDCIR.HIV5pep and αCD40.HIV5pep vaccines in MVA-primed Rhesus macaques. PBMCs were harvested at the indicated time points and analyzed by IFNγ ELISPOT using pools of overlapping Gag Pol and Nef peptides corresponding to sequences carried by the DC-targeting vector (HIV5 peptides, shown in light grey), or pools of overlapping Gag, Pol and Nef peptides corresponding to sequences not carried by the DC-targeting vaccines but carried by MVA GagPolNef (non-HIV5 pep, shown in dark grey). (A) IFNγ ELISPOT data from animals primed with two administrations of MVA GagPolNef, then vaccinated with 250 μg αDCIR.HIV5pep (G1) or (B) αCD40.HIV5pep (G2) plus 1 mg poly-ICLC according to the schedule shown in Table 1 and as indicated by the gray and black arrows below the timeline shown in weeks. (C) and (D) IFNγ ELISPOT data for individual Gag, Pol and Nef peptide stimulations corresponding to sequences carried by the DC-targeting vectors (individual HIV5pep peptides are indicated by colors). Responses for each peptide are the average response per sample point. The X-axis shows sampling time in weeks. S1 Table and S2 Table show data corresponding to this figure. Supporting information S1 File contains the primary data.

In G1 MVA αDCIR and G2 MVA αCD40, two administrations of MVA GagPolNef elicited low levels of HIV-1-specific IFNγ-producing T cells responding to peptides from the HIV5 peptide regions in the blood of the vaccinated animals. This was determined by ELISPOT analysis of PBMCs at weeks 0, 2, 10, and 12 (Fig 2A and 2B). In G1 MVA αDCIR, a single administration at week 12 of αDCIR.HIV5pep vaccine co-administered with poly-ICLC increased IFNγ-producing HIV5-specific T cells in three of the six animals at week 14, but overall the increase was not significant when compared to the week 12 response (p = 0.1) (Fig 2A). In G2 MVA αCD40, a single administration at week 12 of αCD40.HIV5pep vaccine co-administered with poly-ICLC significantly boosted IFNγ-producing T cells specific to epitopes within the HIV5pep sequences in all six animals (p = 0.03 at week 14 compared to week 12). We also observed low levels of IFNγ-producing T cells specific to Gag, Pol, and Nef peptide pools from outside of the HIV5pep regions (non-HIV5pep) in both G1 and G2 in response to the MVA vector, but as expected these were not boosted by the DC-targeting vaccination (p = 0.49 for G1 and p = 0.11 for G2 at week 14 compared to week 12). Two weeks after a second αDCIR.HIV5pep or αCD40.HIV5pep administration, HIV5pep-specific IFNγ^+^ T cells were further expanded compared to the week 14 response in both G1 and G2 (p = 0.03 for both groups comparing the medians of HIV5pep response over the sample times weeks 14, 18, 26 and 28 and responses over the sample times weeks 2, 10, and 12), but no subsequent increases were observed after a third DC-targeting vaccination (weeks 26 and 28, Fig 2A and 2B). Interestingly, when the ELISPOT data was examined for individual Gag Pol and Nef peptide stimulations corresponding to sequences carried by the DC-targeting vector, T cells specific to the Gag253 and Pol158 regions dominated the responses to the DC-targeting vaccines in both groups (Fig 2C and 2D, and Table 2). Overall, the αCD40.HIV5pep vaccine (G2 MVA αCD40) elicited higher levels of HIV5pep-specific T cells than αDCIR.HIV5pep (G1 MVA αDCIR), however this was not statistically significant based on comparison between G1 and G2 responses over weeks 14, 18, 26 and 28 (p = 0.16). However, we could detect T cell responses in all six animals in G2 after one αCD40.HIV5pep administration, while only three out of six animals mounted a T cell response following αDCIR.HIV5pep administration.

**Table 2:** Peptide-specific IFNγ-producing T cell responses to administrations of MVA, αDCIR.HIV5pep and αCD40.HIV5pep.

	Median (IQR)	Gagp17	Gag253	Nef116	Nef66	Pol158
	D0	2.5 (0–5)	7.5 (5–10)	5.0 (1–5)	0 (-)	5.0 (1–5)
**G1**	MVA	0 (-)	10.0 (6–11)	0 (-)	0 (0–1)	20.0 (13–27)
	αDCIR	2.5 (1–3)	162.5 (96–176)	0 (0–1)	5.0 (2–6)	60.0 (42–93)
	D0	5.0 (0–13)	0 (0–11)	2.5 (0–5)	0 (0–3)	10.0 (1–18)
**G2**	MVA	2.5 (1–3)	10.0 (5–13)	0 (0–1)	0 (0–2)	25.0 (23–31)
	αCD40	2.5 (1–6)	240.0 (170–427)	5.0 (3–10)	17.5 (8–25)	540.0 (380–617)
	D0	0 (-)	5.0 (5–12)	0 (-)	0 (0–3)	7.5 (5–77)
**G3**	αDCIR	6.2 (3–26)	18.7 (13–31)	8.5 (5–11)	5.0 (4–6)	116.2 (51–173)
	MVA	5.0 (3–6)	17.5 (17–18)	0 (-)	7.5 (5–10)	171.2 (151–190)
	D0	0 (0–3)	2.5 (0–16)	2.5 (0–5)	0 (0–3)	7.5 (1–13)
**G4**	αCD40	11.5 (8–20)	55.0 (52–55)	8.7 (1–16)	5.0 (1–8)	78.5 (40–138)
	MVA	5.0 (3–6)	67.5 (66–68)	0 (-)	2.5 (1–3)	185.0 (175–195)

IFNγ ELISPOT data (spots per 10^6^ cells) for Gag, Pol and Nef peptides and Gag, Pol and Nef fusion protein stimulations corresponding to sequences carried by the DC-targeting vector (HIV5pep peptides) were summed for each peptide category (Gag p17 represents the sum of p17 pool 1 and Gag17 protein; Gag253 represents the sum of p24 pool 3, p24 pool 4 and Gag253 protein; Nef116 represents the sum of Nef66 protein responses; Nef66 represents the sum of Nef66 protein responses; Pol158 represents the sum of Pol pool 2, Pol pool 3 and Pol158 protein responses). Values in the table correspond to the median calculated over some sample times and by group with the 25^th^ and 75^th^ percentiles in bracket. In G1 and G2, weeks 2, 10, and 12 were used for MVA responses and weeks 14, 18, 26, and 28 for the responses expanded by DC-targeting antibodies. In G3 and G4, weeks 2, 6, 14, 22 were used for the DC-targeting vaccines and 24, 26 for the MVA boost.

**Fig 3:**
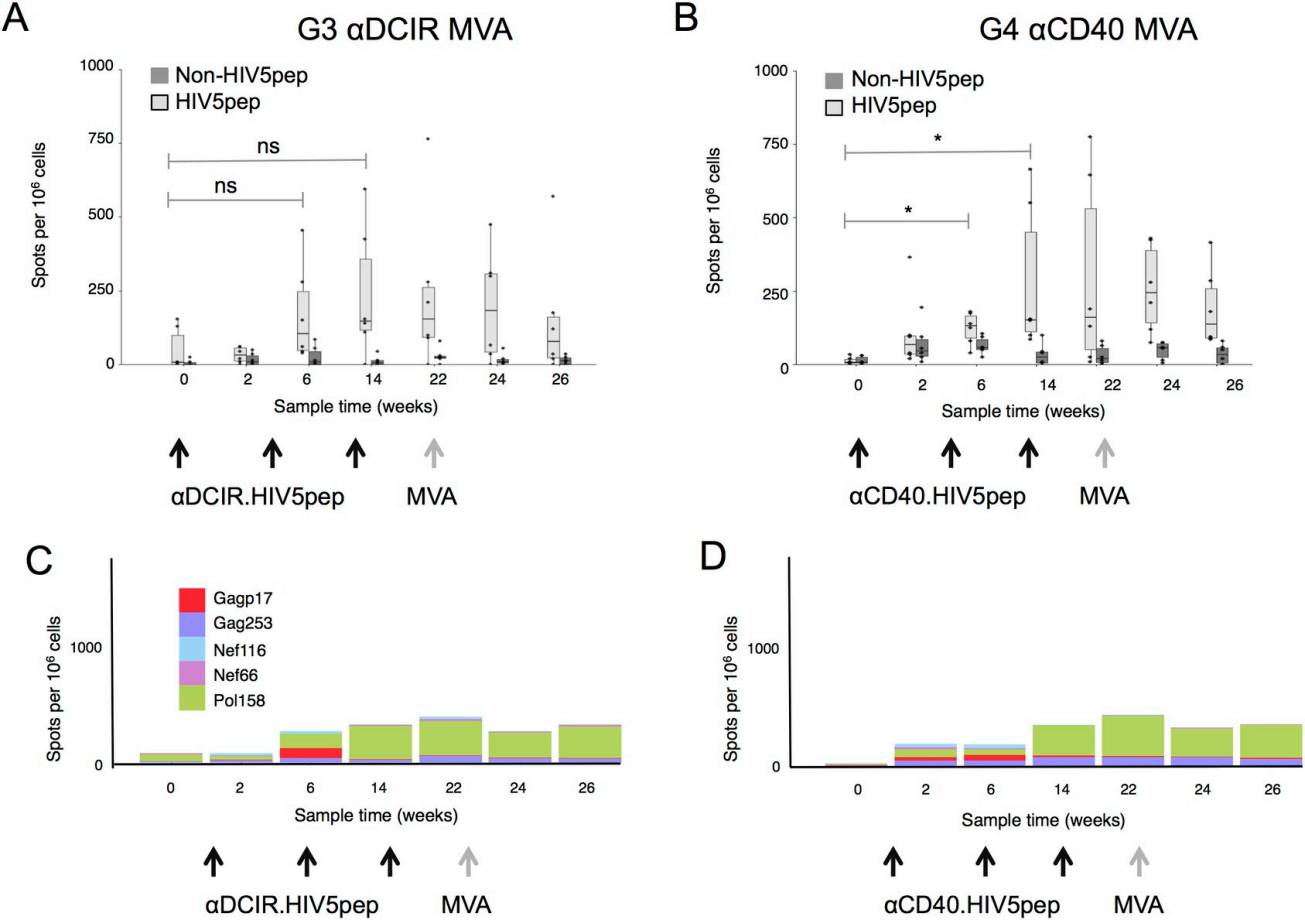
T cell responses to αDCIR.HIV5pep and αCD40.HIV5pep vaccines in naïve Rhesus macaques. PBMCs were harvested at the indicated time points and analyzed by IFNγ ELISPOT using pools of overlapping Gag Pol and Nef peptides corresponding to sequences carried by the DC-targeting vector (HIV5 peptides, shown in light grey), or pools of overlapping Gag Pol and Nef peptides corresponding to sequences not carried by the DC-targeting vaccines but carried by MVA GagPolNef (non-HIV5 pep, shown in dark grey). (A) IFNγ ELISPOT data from animals vaccinated with three administrations of 250 μg αDCIR.HIV5pep (G3) or (B) αCD40.HIV5pep (G4) plus 1 mg poly-ICLC followed by a boost with MVA GagPolNef. Responses from individual animals in the indicated groups are shown as points and were summed for these two pool sets. The mid-line of the box denotes the median, and the ends of the box denote the 25^th^ and 75^th^ percentiles. The whiskers are the minimum/maximum value higher/lower than 1.5* Inter-Quartile Interval. Values after the second and third αDCIR.HIV5pep vaccinations at week 0 compared to weeks 6 or 14 were not significantly different (G3; ns; respectively, p = 0.03, p = 0.1). Values after the second and third αCD40.HIV5pep vaccinations at week 0 compared to weeks 6 or 14 were not significantly different (G4; *; respectively, p = 0.15, p = 0.03). (C) and (D) IFNγ ELISPOT data for individual Gag, Pol and Nef peptides stimulations as described above. The X-axis shows sampling time in weeks. S6 Table and S3 Table and S1 File show data corresponding to this figure.

In naïve NHP G3 αDCIR MVA and G4 αCD40 MVA (Table 1), the αCD40.HIV5pep vaccine co-administered with poly-ICLC elicited circulating IFNγ^+^-producing T cells specific to epitopes within the HIV5pep sequences two weeks after a second DC-targeting vaccination (week 6), but comparison between week 0 and week 6 only reached significance in αCD40 HIV5pep vaccinated animals (p = 0.15 for G3 αDCIR MVA and p = 0.03 for G4 αCD40 MVA, Fig 3A to 3B). Two weeks after a third αDCIR.HIV5pep or αCD40.HIV5pep boost (week 14), HIV5pep-specific T cells producing IFNγ in G4 αCD40 MVA were increased compared to the week 0 values (p = 0.03), but this difference was not significant for G3 αDCIR MVA (p = 0.1) (Table 2). At week 14 there were no significant differences between groups G3 and G4 responses (p = 0.87). Although T cells specific to all the HIV5 peptide regions were detected (Table 2), interestingly T cell responses to the Pol 158 region were dominant in both vaccination groups (Table 2, Fig 3A to 3D). In both vaccine groups the T cell responses peaked at week 14 and remained stable until week 22, but were not further boosted by the MVA GagPolNef boost at week 22 (Fig 3A to 3D).

Thus in either prime or boost settings both DC-targeting vaccines were safe and elicited significant T cell responses covering a range of epitopes within the five HIV-1 long peptide regions. All animals vaccinated with αCD40.HIV5pep mounted HIV-1-specific T cell responses and these responses were detected as early as 2 weeks after the first vaccination.

### HIV-1-specific CD4^+^ and CD8^+^ T cell responses elicited by DC-targeting vaccines in MVA-primed NHPs and naïve animals

**Fig 4:**
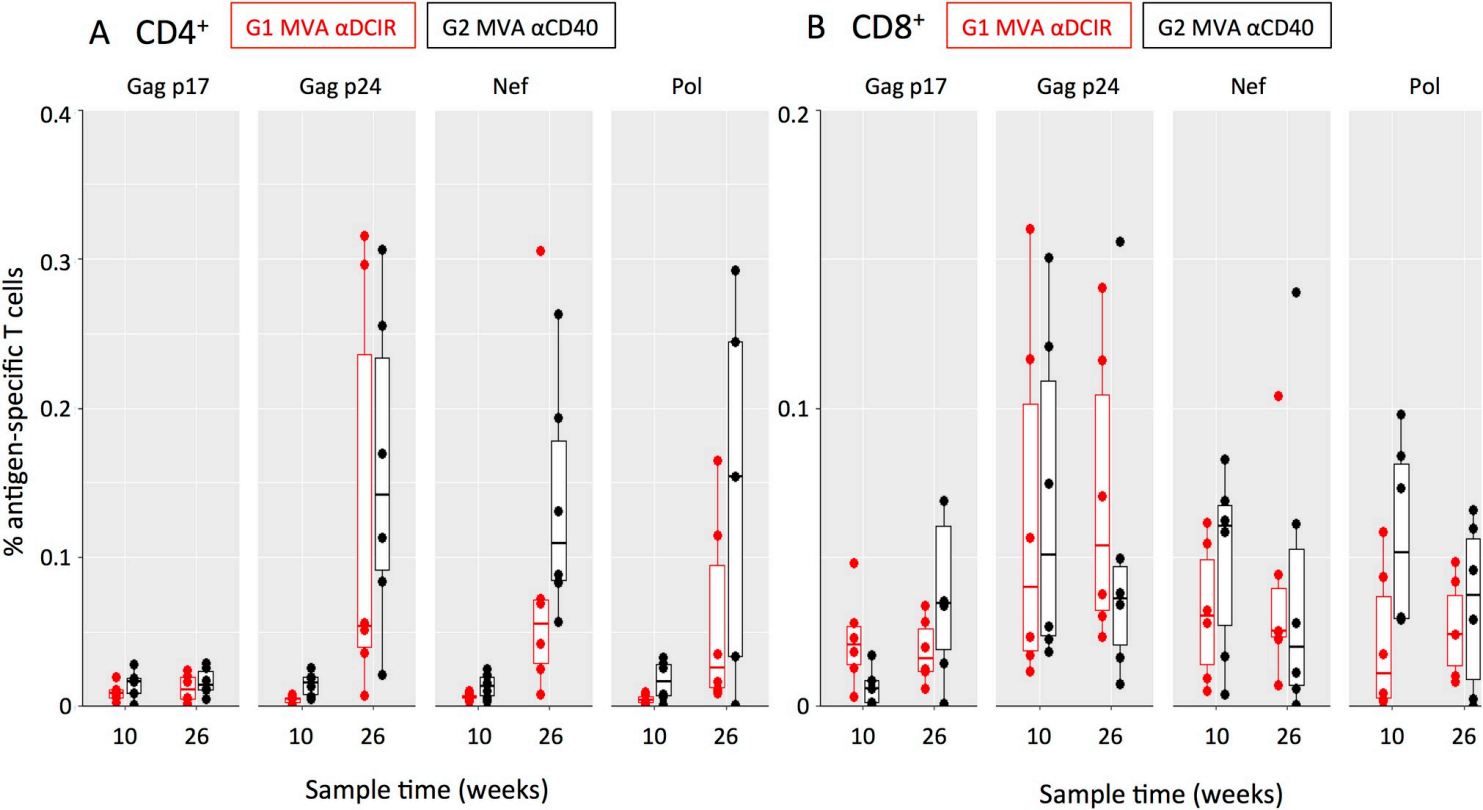
Analysis of HIV-1 epitope-specific CD4^+^ and CD8^+^ T cell responses elicited in MVA-primed NHPs by αDCIR.HIV5pep and αCD40.HIV5pep vaccines. PBMCs were collected from individual animals at week 10 (two weeks post MVA) and at peak response times of week 26 (2 weeks post DC-targeting vaccination) for G1 MVA αDCIR and G2 MVA αCD40. PBMC were stimulated in the presence of Brefeldin A for 6 h with pools of HIV-1 peptides corresponding to the four indicated gene regions and analyzed by flow cytometry. Each dot is the background-subtracted value for individual animals of (A) CD154^+^ CD4^+^ or (B) CD8^+^ T cells secreting IFNγ, TNFα, IL-2, or combinations thereof when stimulated with Gag p17, Gag p24, Nef and Pol peptides. Negative background subtracted values were set to zero. Responses from individual animals in the indicated groups are presented. The mid-line of the box denotes the median, and the ends of the box denote the 25^th^ and 75^th^ percentiles. The whiskers are the minimum/maximum value higher/lower than 1.5* Inter-Quartile Interval. One animal had a value (0.9%) outside the plotted scale for the CD4^+^ T cell response to Pol peptides. S4 Table shows the data corresponding to this figure.

Intracellular cytokine staining (ICS) was used to appraise the breadth of HIV-1-specific CD4^+^ and CD8^+^ T cell responses to the vaccines in naïve NHPs, as well as the quality of the responses as determined by simultaneous production of multiple cytokines (CD154, IFNγ, IL-2, TNFα). In G1 MVA αDCIR and G2 MVA αCD40, two weeks after the second MVA administration (week 10) low levels of circulating CD4^+^ T cells specific to epitopes within the Gag p17, Gag p24, Nef, and Pol regions (Fig 4A) were detected. CD8^+^ T cells specific to multiple HIV-1 epitopes (Fig 4B) were detected as early as week 10. Two weeks after the third DC-targeting vaccine boost in G1 MVA αDCIR and G2 MVA αCD40 (i.e., peak IFNγ ELISPOT response at week 26) the HIV-1-specific CD4^+^ T cell responses, but not the HIV-1-specific CD8^+^ T cell responses, increased in accord with the expansion of HIV5pep-specific T cells observed by ELISPOT (Fig 2). αCD40.HIV5pep expanded HIV5pep-specific CD4^+^ T cells to a greater extent compared to αDCIR.HIV5pep (Fig 4A), but this difference was not significant after summing 1, 2, or 3 cytokines for each animal (S1A and S1B Fig, p = 0.09). HIV-1-specific T cells producing at least one of the cytokines IFNγ, IL-2, and TNFα were detected amongst all positive responses (S2A and S2B Fig). CD4^+^ T cells specific to epitopes within the HIV5pep Gag, Nef, and Pol regions were detected in both groups, although this varied between animals (Fig 4A). Overall the CD8^+^ T cell responses in both groups were similar (Fig 4B and S1B Fig; summing responses producing at least one cytokine for each animal p = 0.69), and animals with CD8^+^ T cells specific to epitopes within all the HIV5pep Gag, Nef, and Pol regions were detectable in each group (Fig 4B).

**Fig 5:**
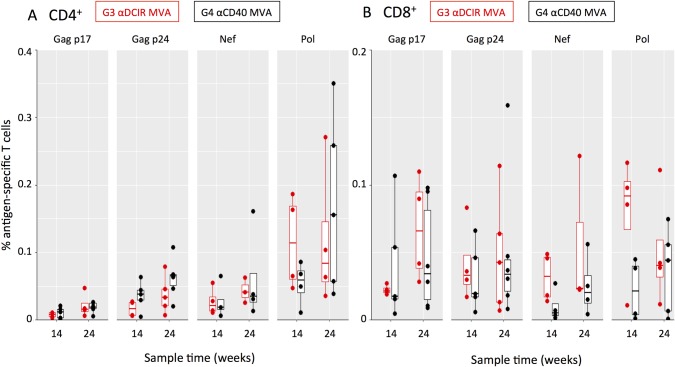
Analysis of HIV-1 epitope-specific CD4^+^ and CD8^+^ T cell responses elicited in naïve NHPs by αDCIR.HIV5pep and αCD40.HIV5pep vaccines. Intracellular cytokine staining analysis of HIV-1 antigen-specific CD154^**+**^ CD4^+^ and CD8^**+**^ T cells elicited by αDCIR.HIV5pep and αCD40.HIV5pep vaccines. PBMCs were collected from individual animals at week 14 (two weeks post DC-targeting vaccines) and week 24 (two weeks post MVA boost) for G3 αDCIR MVA and G4 αCD40 MVA. PBMC were stimulated in the presence of Brefeldin A for 6 h with pools of HIV-1 peptides corresponding to the indicated gene regions and analyzed by flow cytometry. Each dot is the background-subtracted value for individual animals of (A) CD154^+^ CD4^+^ and (B) CD8^+^ T cells secreting IFNγ, TNFα, IL-2, or combinations thereof when stimulated with Gag p17, Gag p24, Nef and Pol peptides. Negative background subtracted values were set to zero. Responses from individual animals in the indicated groups are presented. The mid-line of the box denotes the median, and the ends of the box denote the 25^th^ and 75^th^ percentiles. The whiskers are the minimum/maximum value higher/lower than 1.5* Inter-Quartile Interval. S4 Table shows the data corresponding to this figure.

In the naïve NHP G3 αDCIR MVA and G4 αCD40 MVA, at week 14, two weeks after the third DC-targeting vaccine administration, CD4^+^ and CD8^+^ specific to HIV-1 epitopes across the five long peptide regions T cells were detected in the blood (Fig 5A and 5B). αDCIR.HIV5pep and αCD40.HIV5pep vaccines were equally efficient at expanding HIV-1-specific CD4^+^ and CD8^+^ T cells (after summing 1, 2, or 3 cytokines for each animal, differences between G3 αDCIR MVA and G4 αCD40 MVA were not significant; p = 0.34 for CD4^+^ T cells and p = 0.11 for CD8^+^ T cells, S2C and S2D Fig). Each group had animals with detectable CD4^+^ T and CD8^+^ T cells specific to epitopes within each of the HIV5pep Gag, Nef, and Pol regions (Fig 5A and 5B) and the overall range of HIV5pep epitopes was not significantly different between the αDCIR.HIV5pep and αCD40.HIV5pep vaccine (p = 0.87). Two weeks after a single MVA GagPolNef boost (week 24), the HIV-1-specific CD4^+^ and CD8^+^ T cell responses were not expanded by the MVA administration, in line with HIV5pep-specific T cells observed by IFNγ ELISPOT (Fig 5A and 5B; S2C and S2D Fig).

Thus both DC-targeting vaccines in prime and boost settings elicited multi-functional CD4^+^ and CD8^+^ T cell responses covering a range of epitopes within the five HIV-1 long peptide regions.

### αDCIR.HIV5pep and αCD40.HIV5pep elicit antibody responses in MVA-primed and naïve Rhesus macaques

**Fig 6:**
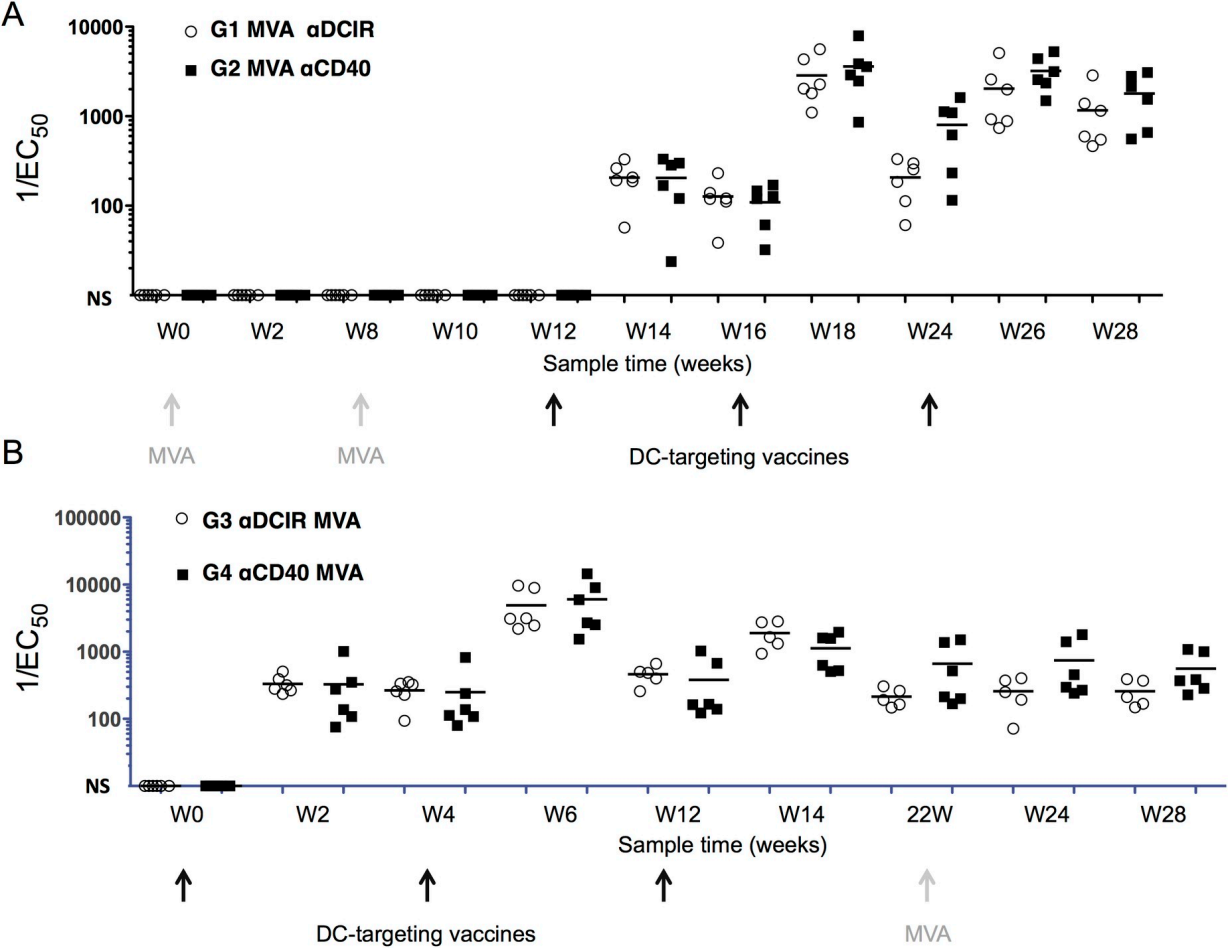
Serum HIV5pep-specific IgG responses elicited by αDCIR.HIV5pep or αCD40.HIV5pep vaccines. (A) Animals primed with two administrations of MVA GagPolNef and vaccinated with αDCIR.HIV5pep (G1) or αCD40.HIV5pep (G2) plus poly-ICLC according to the schedule shown in Table 1 and as indicated by the gray and black arrows below the timeline shown in weeks. Serum HIV5pep-specific IgG levels detected by ELISA using a mixture of fusion proteins for each of the five HIV-1 peptide regions are shown for individual animals. Bars indicate the median for the group at each sample time. 1/EC_50_ values lower than 10 are not shown, but were set to a baseline of 10 for graphical simplicity. There was no significant difference in overall magnitude in weeks 14, 18, and 26 between antibody responses evoked by αDCIR.HIV5pep versus αCD40.HIV5pep (ns; p = 0.36). (B) Similar analysis for NHPs vaccinated with αDCIR.HIV5pep (G3) or αCD40.HIV5pep (G4) plus poly-ICLC according to the schedule shown in Table 1 and as indicated by the arrows below the timeline shown in weeks. They were boosted at week 22 with MVA GagPolNef. S5 Table shows the data corresponding to the figure.

We assayed serum IgG levels reactive to HIV5pep in the NHPs in response to the αDCIR.HIV5pep and αCD40.HIV5pep vaccines. No significant antibody responses developed subsequent to the MVA GagPolNef administrations, but anti-HIV5pep antibody responses developed in all animals two weeks after a single boost vaccination with αDCIR.HIV5pep or αCD40.HIV5pep (G1 MVA αDCIR and G2 MVA αCD40, Fig 6A). These responses were further increased by a second αDCIR.HIV5pep or αCD40.HIV5pep vaccination at week 16, declined somewhat over the next six weeks, were restored to maximal levels at week 26 with a third αDCIR.HIV5pep or αCD40.HIV5pep vaccination, and remained maximal to the end of the study period (Fig 6A). There was no difference in overall magnitude at weeks 14, 18, and 26 between antibody responses evoked by αDCIR.HIV5pep versus αCD40.HIV5pep administrations (p = 0.36). Broad antibody responses were evoked since reactivity against all five regions was readily detectable by similar analyses with peptide-specific ELISAs (see Methods).

In the Rhesus macaque groups G3 αDCIR MVA and G4 αCD40 MVA, we analyzed the serum antibody responses elicited after each DC-targeting prime and the impact of a MVA boost on these responses. Two weeks after a single vaccination with αDCIR.HIV5pep or αCD40.HIV5pep, significant anti-HIV5pep antibody responses developed in all animals (Fig 6B). These were further boosted by a second αDCIR.HIV5pep or αCD40.HIV5pep vaccination at week 4, declined somewhat over the next six weeks, and increased again with a third αDCIR.HIV5pep or αCD40.HIV5pep vaccination. However, these responses were not boosted by the MVA GagPolNef administration at week 22 (Fig 6B). There was no significant difference in overall magnitude between responses evoked by αDCIR HIV5pep versus αCD40 HIV5pep (p = 0.73).

Thus both DC-targeting vaccines in prime and boost settings elicited similar antibody responses against epitopes within the five HIV-1 long peptide regions.

## Discussion

We used the Rhesus macaque model to compare the efficacy for inducing or boosting cellular immunity against HIV-1 by targeting five T cell epitope-rich regions from HIV-1 Gag, Pol, and Nef to the DC receptors DCIR and CD40. Preclinical studies *in vitro* and *in vivo* using model antigens provided a framework for selecting humanized anti-DCIR and anti-CD40 DC targeting antibodies as vehicles for delivery of the HIV5pep sequences to a broad array of antigen-presenting cells [18–20, 28–29, 33], as well as the benefit of poly-IC co-administration as adjuvant for improved T cell immunity [14, 19, 20, 32, 34]. In particular, studies in mice with a humanized immune system showed that poly-IC was superior to other adjuvants for inducing CD8^+^ cytotoxic T cells response to the CD40-targeting HIV5pep vaccine [35], while a study in Rhesus macaques demonstrated that CD4^+^ and CD8^+^ T cell responses to a high dose peptide vaccination were significantly enhanced when an agonistic αCD40 antibody was co-administered with poly-IC [36]. Our study was designed to select from the αDCIR.HIV5pep and αCD40.HIV5pep prototype vaccines one for clinical development based on safety as well as relative efficacy for both priming cellular immunity and boosting memory T cell responses. Based on the more robust and consistent response to the first DC-targeting boost vaccination after MVA priming, as well as after vaccination of naïve NHPs, our data favor targeting CD40 over DCIR.

The design setting in G1 MVA αDCIR and G2 MVA αCD40 partly recapitulates the therapeutic vaccination of HIV-1 individuals under cART, where the DC-targeting vaccines expand memory T cell responses previously primed with the MVA GagPolNef vector. Viewed through this lens, both αDCIR.HIV5pep and αCD40.HIV5pep effectively boosted polyfunctional T cells to a wide range of epitopes, but this was limited to the CD4^+^ T cell compartment. An important caveat is that the HIV5pep sequences were selected as relatively well conserved Class I and Class II T cell epitope-rich regions across a wide array of human haplotypes and the observed magnitude and breadth of HIV5pep-specific T cell responses in our Rhesus macaque study should be considered in this context. A related matter is the high MHC polymorphism of Rhesus macaques [37], which could itself translate into high T cell response variability, explaining why some animals mounted weaker responses. There is no evidence of CD40 or DCIR polymorphism to account for such variation. Both DC-targeting vaccines expanded similar ranges of T cell quality and epitope specificity, but αCD40.HIV5pep was capable of evoking T cell responses even after one DC-targeting vaccination and these response were further boosted by subsequent vaccinations and were sustained over time. The apparent lack of CD8^+^ T cell boosting in G1 MVA αDCIR and G2 MVA αCD40 did not reflect the ability of both DC-targeting vaccines to prime CD8^+^ T cell responses in the naïve G3 αDCIR MVA and G4 αCD40 MVA groups. The failure of MVA GagPolNef to boost the CD8^+^ T cell responses primed by DC-targeting, and conversely the failure of the DC-targeting vaccines to boost the CD8^+^ T cell responses primed by MVA GagPolNef in G1 MVA αDCIR and G2 MVA αCD40, may have similar root causes, i.e., fundamental differences in antigen processing between the two delivery routes. While MVA vectors efficiently infect DCs, there is evidence to suggest that primary CD8^+^ T cell responses induced by MVA vaccination does not depend on antigen presentation by directly infected DC, but rather is induced by DCs that acquire antigen from other infected cells and cross-present it to naïve T cells [38]. Clearly, variables like internalization route, early and late endocytic trafficking, together with TLR activation via poly-ICLC have a determining influence in how antigens are handled for cross-presentation by antigen-presenting cells/DCs, and whether they are DC-targeted antigens versus antigens passively acquired via nearby apoptotic MVA-infected cells [39]. Thus, while CD8^+^ T cell responses corresponding to the HIV5pep regions are elicited by either the DC-targeting vaccines, or via MVA infection, differences in processing may present different arrays of T cell epitopes, favoring new CD8^+^ T cell responses elicited by the heterologous vaccine rather than boosting the existing responses. In a therapeutic clinical setting, sequential αCD40.HIV5pep and MVA GagPolNef vaccinations may actually be beneficial for boosting a broader array of cellular responses in HIV-1-infected individuals.

In our study, delivering the HIV5 peptides via the DC-targeting vaccines evoked broad and sustained HIV-1-specific T cell responses detected as early as two weeks after the first vaccination. In contrast, Cynomolgus macaques vaccinated with a mixture of the HIV5pep peptides linked to a lipid TLR2 agonist (HIV-Lipo-5) did not elicit significant HIV-1-specific T cell responses even after four administrations [40]. Also, while in our study the DC-targeting vaccines efficiently boosted animals primed with MVA, T cell responses were not boosted by HIV-Lipo-5 in similarly primed Cynomolgus macaques [40]. These differences point to key advantages for delivering HIV5 peptides via DC-targeting, especially since the dose of the peptides within the DC-targeting vaccines was only ~3% of the pooled synthetic peptides within HIV-Lipo-5. Especially for CD40-targeting, *in vitro* studies show that much of the αCD40 remains at the targeted DC plasma membrane, but some is internalized mainly to the early endosomes, thus maintaining αCD40 on the plasma membrane with associated extended continuous release of antigens via early endosome processing for prolonged antigen cross-presentation to CD8^+^ T cells [29]. This is in contrast to DCs loaded via antibodies against other receptors such as LOX-1, Dectin-1, or DCIR that are rapidly localized into early and late endosome compartments [29, 41].

From a clinical stand point, prime boost vaccine strategies combining MVA and protein-based vaccines are promising. However, few studies have investigated the best timing for MVA administration as a prime or a boost. This preclinical NHP study of the αDCIR.HIV5pep and αCD40.HIV5pep vaccine candidates demonstrates their safety and utility in eliciting HIV-1 antigen-specific T cell responses in two settings of clinical relevance. Single vaccinations with αDCIR.HIV5pep and αCD40.HIV5pep combined with poly-ICLC as adjuvant efficiently expanded multifunctional memory CD4^+^ but not CD8^+^ T cells primed by a viral vector. However, based on our *in vitro* data [18, 28], it is likely that administration of these therapeutic vaccination strategies will effectively recall anamnestic HIV-1-specific responses in HIV-1-infected individuals. The ability of both αDCIR.HIV5pep and αCD40.HIV5pep vaccines to prime CD4^+^ and CD8^+^ T cells in naïve NHPs also supports this possibility. Because the anti-CD40 and anti-DCIR targeting vehicles are humanized antibodies built on IgG4 and human Kappa chain constant regions, these vaccines are expected to be immunogenic in the NHPs. We have previously noted this phenomenon [17–19], and it is likely that such elicited anti-DC-targeting vehicle antibodies encompass some that block antibody-DC receptor interaction. These could obviously compromise the mechanism of action of these vaccines in repeated vaccinations. However, as noted in this study and previous work, boost responses even after the third vaccination were routinely observed, indicating that anti-vaccine vehicle responses did not completely abrogate boost responses. However, it is likely that T cell responses in humans could be higher since such anti-DC-targeting vehicle responses are not expected. The significant T cell response level for therapeutic purposes is not known, given that the lack of a validated correlates for protection or HIV-1 cure remains one of the major challenges in HIV-1 vaccine research. In humans, therapeutic vaccination with dendritic cells generated *ex vivo* and loaded with HIV-1 lipopeptides (same as the HIV5 peptides used in the present study) in 19 HIV-infected patients on ART induced an increase in HIV-1-specific CD4^+^ T cell cytokine responses after vaccination. This HIV-1-specific CD4^+^ T cell response inversely correlated with maximum HIV-1 viral load values after antiretroviral treatment interruption (r = −0.63, p = 0.007), suggesting that HIV-1-specific CD4^+^ T cell responses after vaccination are associated with a better control of viral replication [42]. Nevertheless, in the absence of known correlates of protection and subsequent bridging studies between species, it is highly speculative to extrapolate results from NHP vaccine trials to humans and vice versa, and from one vaccine candidate to the other. Also, the functional antiviral capabilities of the humoral response are evoked via antibodies that target the HIV-1 gp140 envelope protein. However, levels of antibodies to structural proteins, such as anti-Gag and anti-Nef responses, have no known direct antiviral activity, but can be indicative of an active T helper cell response [43]. Thus the antibody responses we observed against the HIV5pep regions would not be expected to contribute to viral protection or cure, but are a key reflection of productive antigen-specific helper T cell responses.

Our NHP vaccine study was focussed on demonstrating safety and immunogenicity, but efficacy analysis based on demonstrating control of viral load was not planned for several practical reasons: i) foremost is the actual design of this therapeutic vaccine candidate: the long peptides from Gag Nef and Pol were selected and validated to encompass a high density of known CD4^+^ and CD8^+^ T cell HIV-1 epitopes across a wide range of human HLA types, and ii) based on sequence variation between HIV-1 and SIV and the different Rhesus MHC repertoire, the HIV-1 sequences within the vaccine would not be expected to evoke a predictable focussed immune response sufficient to exert an effect on viral load in the context of an NHP model infected with a related non-HIV-1 virus such as SIV or a SHIV. However, in humanized mice infected with HIV-1 and treated with antiviral drugs, αCD40.HIV5pep plus poly-IC vaccination evoked substantial HIV-1-specific CD4^+^ T cell and CD8^+^ T cell responses producing cytokines, leading to significant control of HIV-1 rebound after cART withdrawal as compared to control groups (PBS or poly-IC alone) [44].

In summary, the safety and antigenicity demonstrated in our study suggests that further clinical development of αCD40.HIV5pep is warranted for therapeutic treatment of HIV-1-infected individuals.

## Materials and methods

### Production of αCD40.HIV5pep and αDCIR.HIV5pep vaccines

A first-generation prototype of αCD40.HIV5pep was previously described as a chimera of anti-human CD40 mouse 12E12 monoclonal antibody variable regions grafted to human kappa light chain constant region and to human IgG4 heavy chain constant region containing two mutations to stabilize the labile disulfide bond and to abrogate residual FcR binding [28]. For this study, we ‘humanized’ the mouse variable regions to reduce antigenicity in humans and relocated two of the HIV5pep regions onto the light chain C-terminus to improve productivity. These sequences, hAnti-CD40VH3-LV-hIgG4H-C-Flex-v1-Pep-gag17-f1-gag253-f2-nef116-f3 (GenBank KM660791) and hAnti-CD40VK2-LV-hIgGK-C-pol158-f3-nef66 (GenBank KM660792), were configured in vectors and expressed and purified as protein secreted from stably transfected CHO-S cells as described previously [28, 45]. Similar constructs (hAnti-DCIRVH4-LV-hIgG4H-C-Flex-v1-Pep-gag17-f1-gag253-f2-nef116-f3; GenBank KT363873 and hAnti-DCIRVK2-LV-hIgGK-C-pol158-f3-nef66; GenBank KT363874) were made for αDCIR.HIV5pep production with humanized variable regions from the anti-human DCIR monoclonal antibody 9E8 [31]. The sequences appended to the H chain are respectively: Flex-v1-asqtptntisvtptnnstptnnsnpkpnpas; gag17-ekirlrpggkkkyklkhiv; f1-asssvspttsvhptptsvpptptksspas; gag253-nppipvgeiykrwiilglnkivrmysptsild, f2-asptstpadsstitptatptatptikgas; nef116-htqgyfpdwqnytpgpgvrypltfgwlykl; f3-astvtptatatpsaivttitptattkpas. The sequences appended to the L chain are respectively: pol158-aifqssmtkilepfrkqnpdiviyqymddly; f3-astvtptatatpsaivttitptattkpas; nef66-vgfpvtpqvplrpmtykaavdlshflkekggl(as). Also produced was a humanized DCIR-targeting analog of the first-generation CD40-targeting vaccine: hAnti-DCIRVH4-LV-hIgG4H-C-Flex-v1-gag17-f1-gag253-f2-nef116-f3-nef66-f4-pol158 with anti-hDCIRVK2-hIgGK based on combining relevant C-terminal sequences from GenBank KM660789/ KM660790 and N-terminal sequences from Genbank KT363873/ KT363874. The anti-CD40 and anti-DCIR antibodies were originally developed against human ectodomains with mouse hybridoma technology and subsequently validated to bind to Cynomolgous macaque immune cell types expressing these receptors [46]. The CD40 ectodomain is 95% identical between human and Rhesus macaque and 99% identical between the Cynomolgous and Rhesus macaques. The DCIR ectodomain is 92% identical between human and Rhesus macaque and 100% identical between Rhesus and Cynomolgous macaques. For quality assurance, the vaccines were appraised by reducing SDS PAGE analysis (as shown in Fig 1A). An adaption of a multiplexed bead-based assay [33] using bead-bound human and Rhesus macaque DCIR and CD40 ectodomains (encoded respectively by GenBank gb|AAF14348.1|AF109146_1 residues 70–237, ref|XP_005570094.1| residues 69–237, gb|AAO43990.1| residues 22–193, and ref|NP_001252791.1| residues 21–193) fused via the cohesin C-terminus (GenBank gb|CP000568.1|residues 3622666–3623172 with a Nhe I site linker) was used to confirm binding to their target human and Rhesus macaque receptors with affinities close to their antibody counterparts not fused to the HIV5pep sequences, we used. In this assay, beads were coated with human DCIR, NHP DCIR, human CD40, or NHP CD40 ectodomains and incubated overnight with 10 ng/ml of the parental mouse αDCIR 9E8 or αCD40 12E12 mAbs and varying concentrations of humanized αDCIR, αDCIR.HIV5pep, αCD40, or αCD40.HIV5pep, then probed with PE-labeled anti-mouse IgG, and analyzed with a Bio-Plex 200 instrument. In this test there was no significant difference between the binding of humanized αDCIR vs. αDCIR.HIV5pep to human (EC_50_ 0.1 nM) or NHP DCIR (IC_50_ 0.1 nM) coated beads, and the binding of humanized αCD40 vs. αCD40.HIV5pep to human (IC_50_ 0.25 nM vs. 0.05 nM) or NHP (IC_50_ 0.2 nM vs. 0.1 nM). CD40 coated beads was similar (data not shown). The vaccines had low lipopolysaccharide levels of 0.04–0.07 ng/mg of protein. These prototype vaccines were tested to confirm that the second-generation configurations had similar efficacies to the first-generation forms in expanding memory T cells *in vitro* from CART patient PBMCs specific to epitopes from all five HIV-1 peptide regions and elicited a similar range of epitope responses. Specifically, PBMCs from an HIV-infected patient were cultured for 10 days with a dose range from 3 pM—3 nM of first and second generation humanized αCD40.HIV5pep, 0.3 nM and 3nM doses of first generation αDCIR.HIV5pep and second generation αDCIR.HIV5pep, or left unstimulated. The cultures were then restimulated for 48 h with or without 19–32 residue long peptides covering the HIV5pep Gag, Nef and Pol peptides and culture supernatants were then analyzed by multiplex bead-based assay for IFNγ secretion. Responses compared by a Spearman correlation analysis were r = 0.893, p<0.0001 for second generation αCD40.HIV5pep vs. first generation αCD40.HIV5pep and r = 0.8303, p<0.0047 for second generation αDCIR.HIV5pep vs. first generation αDCIR.HIV5pep (S1 Fig). For the above tests PBMCs were prepared from apheresis donations performed on cART-treated HIV-1-infected individuals after written informed consent was collected. This protocol was reviewed and approved by the Baylor Research Institute Institutional Review Board. The HIV-1-infected individuals were not selected based on their CD4^+^ T cell counts (253–480 cells/μl) but they all controlled their viral load (HIV-1 RNA viral load level < 50 copies/ml) under cART. Procedures for the expansion of HIV-1-specific T cells within peripheral blood mononuclear cells from HIV-1 individuals under cART were as described [28, 47].

### Production of HIV5pep fusion proteins

Proteins with the individual five HIV-1 peptide regions fused to cohesin were expressed as soluble intracellular proteins from *E*. *coli* as described [44] using the pET-28C (Novagen) vector modified by the insertion of GenBank KT388713 residues 1–558 into the Nco I-Not I interval followed by insertion into the resulting Nhe I-Not I interval of Spe I-Not I peptide-coding fragments terminated by a stop codon corresponding to GenBank KM660789.1 residues 883–993, 1483–1550, 1627–1733, 1810–1910, and 2170–2277. Cells were grown and supernatant fractions prepared as described [48]. To remove LPS, Triton X-114 (Thermo Scientific) was added to the supernatant fractions to a final concentration of 1% (w/w) and incubated in an ice bath with intermittent shaking until the solution became clear and homogeneous, then transferred to a 37°C water bath for 10 min, and the two-phase system was separated by centrifugation at 2,000 g at room temperature for 10 min. After discarding the bottom Triton X-114 phase, another two cycles of treatment were performed with the top aqueous phase. Binding buffer (0.5 M NaCl, 20 mM Tris.HCl, 5 mM Imidazole, pH 7.9) was added to the solution and loaded onto a 5 ml HiTrap Chelating Ni^++^ column (GE Lifescience), washed with 0.5 M NaCl, 20 mM Tris.HCl, 20 mM Imidazole, pH 7.9 and eluted with a gradient to 0.5 M NaCl, 20 mM Tris, 500 mM Imidazole, pH 7.9. Fractions containing the proteins were dialyzed into DPBS (Gibco) and quantitated by UV spectroscopy.

### Analysis of serum antibody responses

For ELISA, the plates were coated with 2 μg/ml of the cohesin fusion protein pool (i.e., 10 μg/ml total protein) in 0.2 M sodium carbonate-bicarbonate buffer, pH 9.4. Serial dilutions of serum starting at 1:75 in TBS blocking solution (StartingBlock T20, Pierce) were incubated in the wells overnight at 4°C. After washing, plates were incubated with HRP-conjugated goat anti-human IgG (Jackson ImmunoResearch) in TBS blocking solution (Thermo Scientific) for 2 h at 37°C, then washed and developed with HRP substrate (TMB, Life Technologies), stopped with equal volume of 1 N HCl and read at 450 nm. The EC_50_ values were calculated for each animal at each time point using Prism 6 software (GraphPad) and were based on Log_10_ transformed and normalized data with non-linear regression curve fit using sigmoidal dose response with variable slope constraints. The EC_50_ value is calculated from samples with high antibody responses that yield suitable sigmoidal curves, including a clear 100% plateau. In some cases samples generate low but detectable absorbance signals, but the maximal signal absorbance signal at the starting dilution for the sample falls below the calculated EC_50_ value. Here the software extrapolates the curve to estimate the sample EC_50_ value, which will yield 1/ EC_50_ values less than the starting dilution. S3 Fig and S6 Table exemplify average titration curves used to calculate EC_50_ values. Similar analysis with ELISA specific to each HIV5pep region indicated that these antibody responses were broad, with reactivity detectable against all five regions.

### Intracellular cytokine staining

Cryopreserved non-human primate PBMC were thawed and rested overnight in RPMI 1640 (Life Technologies) with 10% fetal bovine serum, 2 mM L-glutamine, 100 U/ml penicillin, 100 μg/ml streptomycin in a 37°C/5% CO_2_ incubator (Complete RPMI). The following morning, cells were stimulated with peptide pools (2 μg/ml) in the presence of Brefeldin A (10 μg/ml; Sigma-Aldrich) for 6 h. Negative controls received an equal concentration of DMSO peptide solvent without peptides. Five groups of pooled peptides covering Gag p2.p6.p7 (non-HIV5pep), Gag p17, Gag p24, Nef and Pol, as well as Staphylococcal Enterotoxin B (1 μg/ml) as a positive control, were used for the stimulations [49]. Intracellular cytokine staining was performed as described [50]. In particular, we used gating on CD154^+^ CD4^+^ T cells to characterize the antigen-specific CD4^+^ T cell responses in macaques [51]. S4 Fig shows a representative ICS analysis. The following monoclonal antibodies were used: CD4-PerCPCy5.5 (clone L200; BD Biosciences), CD8-PECy7 (clone RPA-T8; BD Biosciences), CD3-APCCy7 (clone SP34.2; BD Biosciences), CD154-FITC (clone TRAP1; BD Biosciences) IFN-γ-V450 (clone B27; BD Biosciences), IL-2-APC (clone MQ1-17H12; BD Biosciences), and TNFα-PE (clone Mab11; BD Biosciences). Aqua LIVE/DEAD Kit (Invitrogen) was used to exclude dead cells. Samples were acquired on a Canto II flow cytometer (BD Biosciences) and analyzed using FlowJo version 9.8 software (Treestar).

### ELISPOT

96 well filtration plates (Millipore) were pre-treated with 70% EtOH, washed five times with 1X phosphate buffered saline and then coated with 5 μg/ml mouse-anti-human-IFNγ antibody (BD Pharmingen) overnight at 4°C. After blocking with complete RPMI for 2 h at 37°C, 2x10^5^ PBMCs were stimulated in triplicates with peptide pools at 1 μg/ml, or phytohaemagglutinin (2.5 μg/ml) as positive control, while addition of medium only served as negative control. The peptides and peptide pools used are those previously described [47]. Plates were incubated at 37°C for 18–24 h before washing with cold H_2_O twice and five times with phosphate buffered saline containing 0.1% (v/v) Tween 20. Biotinylated anti-human IFNγ antibody (cross reactive with IFNγ from non-human primates, Mabtech) was added at 1 μg/ml for 1 h at 37°C and, after washing, a 1: 2000 dilution of Avidin-HRP (Vector Laboratories) was added for 1 h at 37°C. After final washing, stable DAB (Invitrogen) was added to the plate, incubated for 2 min at RT, and then the reaction was stopped by thorough rinsing with water. After drying, the numbers of spots in each well were counted with an automated ELISPOT plate reader (CTL Immunospot).

### Statistics

Ethical considerations limit NHP group sizes, but statistical approaches were based on each group size and support the conclusions. Thus due to the sample sizes in our study, we used non-parametrical statistical tests and limited the number of tests performed. Wilcoxon signed-rank tests were used to compare change of marker value at specific time points within each group. Comparison between groups at specific time points used the Wilcoxon rank sum test. The magnitudes of ELISPOT and ELISA responses were compared between groups over time by fitting a linear random effect model using log-transformed data. No adjustments are made for multiple comparisons, as these are exploratory analyses. A p-value of less than or equal to 0.05 is considered statistically significant. Statistical analyses were done with R (version 3.1.2; The R foundation for Statistical Computing, Vienna, Austria).

### Study approval

Twenty four male Rhesus macaques ranging in age from 3 to 6 years and weighing at least 4 kg were procured from Harlan Laboratories and housed at the Advanced Bioscience Laboratories (ABL) animal facility, which is accredited by the American Association for the Accreditation of Laboratory Animal Care International. ABL’s veterinary practices comply with all policies of the "Guide for the Care and Use of Laboratory Animals," DHHS (NIH 85–23), Animal Welfare (DHHS-TN 73–2) the NIH Manual Issuance 4206 and 6000-3-4-58, "Responsibility for Care and Use of Animals CDC/NIH 4th edition”, “Biosafety in Microbiological and Biomedical Laboratories,” and Public Health Service Policy on Humane Care and Use of Laboratory Animals under a Category 1 assurance from OLAW and complied with compliance with ARRIVE guidelines. ABL’s Institutional Animal Care and Use Committee approved of the study protocol (AUP521). Additional details concerning animal care are given in below. Each group has six animals. For vaccination, modified vaccinia virus Ankara (MVA) HIV-B lot Z568 was thawed at 4°C and administered at 4.5x10^7^ pfu per animal by subcutaneous injection (s.c.) of 450 μl in the upper back. MVA stock was 10^8^ pfu/ml, 0.5 ml/vial and the vector encoded the full-length codon-optimized sequence of Gag (encoding amino acids [aa] 1 to 512) fused with fragments from Pol (encoding aa 172 to 219, 325 to 383, and 461 to 519) and Nef (encoding aa 66 to 147 and 182 to 206) from the HIV-1 Bru/Lai isolate (Los Alamos database accession number K02013) [52]. The poly-ICLC adjuvant (Hiltonol Lot: PJ215-1-10-01) was administered via two s.c. injections of 0.5 mg in 250 μl at the center of each circular injection pattern (3–4 cm diameter) formed by the intradermal (i.d.) administrations of the DC-targeting vaccines were performed at four sites on each side of the dorsal thoracic area in a circular pattern of 3–4 cm of diameter with injection of poly-ICLC performed s.c. in the middle of each circle. αDCIR.HIV5pep or αCD40.HIV5pep vaccine components were stored in 1 M Arginine + 100 mM Tris.HCl buffer pH 6–8. Protein vaccine administrations were given at a 250 μg dose i.d. in a total of 8 injections of 250 μl each (2 ml total injection)—four injections were performed on each side of the dorsal thoracic area arranged in a circular pattern of 3–4 cm of diameter. To avoid toxicity of 1 M Arginine buffer, the concentrated protein was diluted to an appropriate concentration to at least 1:4 in PBS before use. The skin was shaved before injection and cleaned with 70% alcohol solution. Intradermal injections were performed using an insulin syringe. The injection of poly-ICLC was performed after the protein injections in the middle of each circle with 250 μl injected s.c. This administration procedure was designed to promote drainage of antigen and adjuvant to the same lymph node site.

### Animal care and vaccine safety

The animals were grouped based on equal distribution of MHC and TRIM resistance across each group followed by assessment of average weights according to ARRIVE guidelines. All procedures were carried out under ketamine anesthesia by trained personnel under the supervision of veterinary staff and all efforts were made to ameliorate the welfare and to minimize animal suffering in accordance with the “Weatherall report for the use of non-human primates” recommendations. Toys or enrichment was provided to the study animals. Environmental enrichment can be described as follows: The ABL primate environmental enrichment program aimed to promote the psychological and physiological well being of non-human primates, including engagement in species-typical behavior. The main strategies included: social enrichment through group housing; sensory and cognitive enrichment through a novel food program, auditory and visual enrichment through presentation of approved music and visual stimuli plus distribution of foraging or novel toys/devices; identification of and individualized treatment for psychological distress; training to reduce or eliminate stress during human-animal interactions; and program evaluation and documentation via daily assessments conducted by husbandry staff and quarterly behavioral assessments performed by the Program Veterinarian. Euthanasia was used at the conclusion of the study. ABL followed the current AVMA Guidance on Euthanasia—which was conducted by an overdose of IV barbiturate. Other than for a seven-day post-inoculation follow up observation period, animals were pair-housed in adjoining primate cages allowing social interactions, under controlled conditions of humidity, temperature and light (12 h light/12 h dark cycles). Water was available *ad libitum* and animals were monitored and fed standard laboratory rations twice daily. Trained personnel offered dietary supplements with fresh fruit and occasional treats at least once a day. Early endpoint criteria, as proposed by the project team and approved by the Institutional IACUC, were used to determine when animals should be humanely euthanized. The ABL veterinarian was authorized to determine whether animals met such criteria and if necessary, was tasked to stabilize any affected animals prior to consulting with the lead investigators. One animal was lost part of the way through the study for issues unrelated to the vaccine or its administration. Specifically, Monkey R387 had exploratory laporatomy resulting from non responsive diarrhea for the previous 6 weeks, a palpably thickened, abnormal colon, and hazy, thickened loops of bowel radiographically. Surgical findings included a uniformly thickened, firm, and edematous colon and markedly enlarged mesenteric lymph nodes. Biopsy of the colon and mesenteric lymph node was submitted for histopath. Microscopic finding was a chronic, lymphoplasmacytic, eosinophilic colitis and chronic, proliferative lympadenopathy. Thus, a presumptive diagnosis of Inflammatory Bowel Disease was reached. This monkey was treated with restricted diet and metronidazole and tylan and appeared initially to respond however there continued to be intermittent episodes of severe diarrhea. Immodium was added to this monkeys treatment on an as needed basis. Subsequently, this monkey presented with diarrhea with blood and mucous and a decreased appetite. Bloodwork and further treatment including iv fluid therapy was instituted but the animal's condition continued to deteriorate. However, he was still eating (although appetite was diminished) and was not in any apparent pain. Four days later, the monkey appeared hunched and in pain, pale and weak. Within an hour he was minimally responsive with severe hypotension and resuscitative efforts were unsuccessful. At that time the monkey was euthanized. Ulcerative colitis with a perforation of the colon and septic peritonitis were found on necropsy. The pathologist noted that a number of sections of colon had marked thickened smooth muscle tunics suggestive of a neoplastic process such as leiomyomas/leiomyosarcomas. Otherwise, no reactions or adverse events were reported for this study showing that the vaccine administrations did not elicit adverse reactions.

## Supporting information

S1 Fig Analysis of multi-functional cytokine production by antigen-specific CD4^+^ and CD8^+^ T cell responses in NHPs. (A) CD4^+^ T cell responses and (B) CD8^+^ T cell responses in animals were primed with MVA GagPolNef then boosted with αDCIR.HIV5pep or αCD40.HIV5pep (G1 MVA αDCIR and G2 MVA αCD40. (C) CD4^+^ T cell responses and (D) CD8^+^ T cell responses in animals vaccinated three times (3x) with αDCIR.HIV5pep or αCD40.HIV5pep then boosted with MVA GagPolNef (G3 αDCIR MVA and G4 αCD40 MVA. PBMCs were collected from individual animals two weeks after the second MVA GagPolNef administration (week 10) and two weeks after the second DC-targeting vaccine boost (i.e., peak response at week 26; see Fig 2). Cells were stimulated with pools of HIV-1 peptides in the presence of Brefeldin A for 6 h, permeabilized, then analyzed by flow cytometry, and categorized as secreting one, two, or three analyzed cytokines. Each dot is the background-subtracted value for individual animals of CD154^+^ CD4^+^ or CD8^+^ T cells secreting IFNγ, TNFα, IL-2, or combinations thereof when stimulated with Gag p17, Gag p24, Nef and Pol peptides. Negative background subtracted values were set to zero. Boxes represent the 25^th^ and 75^th^ percentile, the horizontal bar is the median, and the whiskers are the minimum/maximum value higher/lower than 1.5* Inter-Quartile Interval and are the % of CD154^+^ CD4^+^ or CD8^+^ cells expressing 1, 2 or 3 cytokines (IL-2, IFNγ, TNFα) after summing for Gag p17/24, Nef and Pol peptides. S3 Table shows the data that corresponds to this figure.
(PDF)

S2 Fig Humanized αCD40 HIV5pep and αDCIR HIV5pep vaccines expand a similar range of HIV-1-specific T cells. PBMCs from an HIV-1-infected individual were cultured for 10 days with a dose range from 30 pM to 3 nM of αCD40.HIV5pep (black-grey filled bars), 30 pM to 3 nM of αDCIR.HIV5pep) dark blue-light blue bars), or left unstimulated and then restimulated (C-) for 48 hours with or without 19–32 residue long peptides covering the specified HIV-1 Gag, Nef and Pol long peptide regions. The culture supernatants were then harvested and the total T cell secreted IFNγ was analyzed by multiplex bead-based assay. The error bars are the standard error of the mean of replicates.
(PDF)

S3 Fig Titration curves used for the calculation of the serum antibody response presented in Fig 6. These data are for weeks 0–16 in G1 and G2, and for weeks 0–14 in G3 and G4. Vaccine or vaccine inj refer to administration of the DC-targeting vaccines with adjuvant. The raw data for this graph is contained in S6 Table.
(PDF)

S4 Fig Flow cytometric analysis of intracellular staining analysis for Gag p24-specific IL-2, TNFα and IFNγ-producing CD4^+^ and CD8^+^ T cells. Cryopreserved cells were stimulated with peptide pools (2 μg/ml) in the presence of Brefeldin A for 6 h. Intracellular cytokine staining was performed (see Methods) and anyzed by flow cytometry. Upper two panels are CD4^+^ T cells and the lowe two panels are CD8^+^ T cells. The annotated quadrants indicate the gates used to quantify % cytokine positve cells.
(PDF)

S1 Table IFNγ ELISPOT data using pools of overlapping Gag, Pol and Nef peptides corresponding to sequences carried by the DC-targeting vector or specifically by the MVA vector. This table is the data that relates to Fig 2 (G1 and G2) and Fig 3 (G3 and G4) panels A and B. Animal name, group, DC-targeting peptides or MVA-specific peptides and sample time in weeks are identified. The values are the sum of spots for each peptide set.
(PDF)

S2 Table IFNγ ELISPOT data for individual Gag, Pol and Nef peptide stimulations corresponding to sequence carried by the DC-targeting vectors. This table is the data that relates to Fig 2 (G1 and G2) and Fig 3 (G3 and G4) panels C and D. Peptide names, group, and sample time in weeks are identified. The values are the sum of spots for each peptide set.
(PDF)

S3 Table Analysis of HIV-1 epitope-specific CD4^+^ and CD8^+^ T cell responses elicited in MVA-primed NHPs by αDCIR.HIV5pep and αCD40.HIV5pep vaccines and in naïve NHPs by αDCIR.HIV5pep and αCD40.HIV5pep vaccines. This table is the data that relates to S1 Fig. Animal name, group, T cell type and sample time in weeks are identified. The % response values for either HIV-1 antigen-specific CD4^+^ or CD8^+^ T cells are the sum of 1 cytokine, 2 cytokines, and three cytokines as determined by the ICS analysis.
(PDF)

S4 Table Analysis of HIV-1 epitope-specific CD4^+^ and CD8^+^ T cell responses elicited in MVA-primed NHPs by αDCIR.HIV5pep and αCD40.HIV5pep vaccines and in naïve NHPs by αDCIR.HIV5pep and αCD40.HIV5pep vaccines. This table is the data that relates to Fig 4 (G1 and G2) and Fig 5 (G3 and G4). Animal and peptides name, group, T cell types and sample time in weeks are identified. The % response values for either HIV-1 antigen-specific CD4^+^ or CD8^+^ T cells are the sum of 1 cytokine, 2 cytokines, and three cytokines as determined by the ICS analysis.
(PDF)

S5 Table Serum HIV5pep-specific IgG responses elicited by αDCIR.HIV5pep or αCD40.HIV5pep vaccines. This table is the data that relates to Fig 6. Animal numbers, group, and sample time in weeks are identified. D 0 is sample just prior to study initiation. The 1/EC_50_ values were calculated as indicated in the Methods section.
(PDF)

S6 Table Sample titration curves used for the calculation of the serum antibody response presented in Fig 6. These are the primary readings for titration of the sera corresponding to the indicated samples for each of the six animals in each group. The dilution series from top to bottom were, respectively, 1/75, 1/225, 1/675, 1/2024, 1/6060, 1/18214, 1/54644, and 1/163944. These data are plotted as average values in S3 Fig.
(PDF)

S1 File ELISPOT primary data for Fig 2 and Fig 3. The tables show interferon gamma ELISPOT responses to each peptide pool tested for each NHP at every sample time.
(PDF)
