# Supplementary material for: HIV-1 T cell epitopes targeted to Rhesus macaque CD40 and DCIR: A comparative study of prototype dendritic cell targeting therapeutic vaccine candidates
Source: PLoS One. 2018 Nov 30;13(11):e0207794. doi: 10.1371/journal.pone.0207794 (PMC6267996; doi:10.1371/journal.pone.0207794)
Supplement: S1 Fig — (A) CD4+ T cell responses and (B) CD8+ T cell responses in animals were primed with MVA GagPolNef then boosted with αDCIR.HIV5pep or αCD40.HIV5pep (G1 MVA αDCIR and G2 MVA αCD40. (C) CD4+ T cell responses and (D) CD8+ T cell responses in animals vaccinated three times (3x) with αDCIR.HIV5pep or αCD40.HIV5pep then boosted with MVA GagPolNef (G3 αDCIR MVA and G4 αCD40 MVA. PBMCs were collected from individual animals two weeks after the second MVA GagPolNef administration (week 10) and two weeks after the second DC-targeting vaccine boost (i.e., peak response at week 26; see Fig 2). Cells were stimulated with pools of HIV-1 peptides in the presence of Brefeldin A for 6 h, permeabilized, then analyzed by flow cytometry, and categorized as secreting one, two, or three analyzed cytokines. Each dot is the background-subtracted value for individual animals of CD154+ CD4+ or CD8+ T cells secreting IFNγ, TNFα, IL-2, or combinations thereof when stimulated with Gag p17, Gag p24, Nef and Pol peptides. Negative background subtracted values were set to zero. Boxes represent the 25th and 75th percentile, the horizontal bar is the median, and the whiskers are the minimum/maximum value higher/lower than 1.5* Inter-Quartile Interval and are the % of CD154+ CD4+ or CD8+ cells expressing 1, 2 or 3 cytokines (IL-2, IFNγ, TNFα) after summing for Gag p17/24, Nef and Pol peptides. S3 Table shows the data that corresponds to this figure. (PDF) [file pone.0207794.s001.pdf]

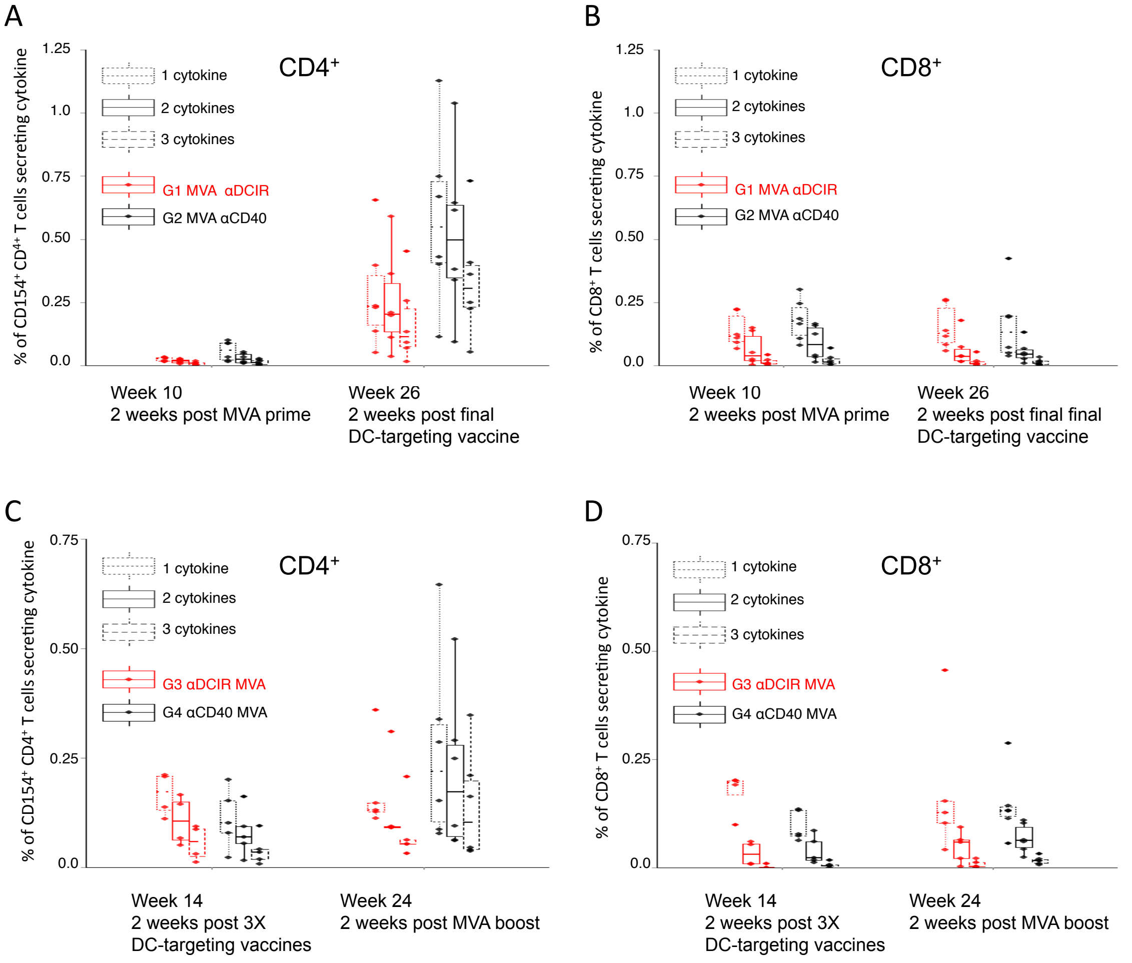

S1 Fig. Analysis of multi-functional cytokine production by antigen-specific $CD4^+$ and $CD8^+$ T cell responses in NHPs. (A) $CD4^+$ T cell responses and (B) $CD8^+$ T cell responses in animals were primed with MVA GagPolNef then boosted with αDCIR.HIV5pep or αCD40.HIV5pep (G1 MVA αDCIR and G2 MVA αCD40. (C) $CD4^+$ T cell responses and (D) $CD8^+$ T cell responses in animals vaccinated three times (3x) with αDCIR.HIV5pep or αCD40.HIV5pep then boosted with MVA GagPolNef (G3 αDCIR MVA and G4 αCD40 MVA. PBMCs were collected from individual animals two weeks after the second MVA GagPolNef administration (week 10) and two weeks after the second DC-targeting vaccine boost (i.e., peak response at week 26; see Fig. 2). Cells

were stimulated with pools of HIV-1 peptides in the presence of Brefeldin A for 6 h, permeabilized, then analyzed by flow cytometry, and categorized as secreting one, two, or three analyzed cytokines. Each dot is the background-subtracted value for individual animals of $CD154^+$ $CD4^+$ or $CD8^+$ T cells secreting IFNγ, TNFα, IL-2, or combinations thereof when stimulated with Gag p17, Gag p24, Nef and Pol peptides. Negative background subtracted values were set to zero. Boxes represent the 25th and 75th percentile, the horizontal bar is the median, and the whiskers are the minimum/maximum value higher/lower than 1.5* Inter-Quartile Interval and are the % of $CD154^+$ $CD4^+$ or $CD8^+$ cells expressing 1, 2 or 3 cytokines (IL-2, IFNγ, TNFα) after summing for Gag p17/24, Nef and Pol peptides. S3 Table shows the data that corresponds to this figure.
