# Supplementary material for: HIV-1 T cell epitopes targeted to Rhesus macaque CD40 and DCIR: A comparative study of prototype dendritic cell targeting therapeutic vaccine candidates
Source: PLoS One. 2018 Nov 30;13(11):e0207794. doi: 10.1371/journal.pone.0207794 (PMC6267996; doi:10.1371/journal.pone.0207794)
Supplement: S2 Fig — PBMCs from an HIV-1-infected individual were cultured for 10 days with a dose range from 30 pM to 3 nM of αCD40.HIV5pep (black-grey filled bars), 30 pM to 3 nM of αDCIR.HIV5pep) dark blue-light blue bars), or left unstimulated and then restimulated (C-) for 48 hours with or without 19–32 residue long peptides covering the specified HIV-1 Gag, Nef and Pol long peptide regions. The culture supernatants were then harvested and the total T cell secreted IFNγ was analyzed by multiplex bead-based assay. The error bars are the standard error of the mean of replicates. (PDF) [file pone.0207794.s002.pdf]

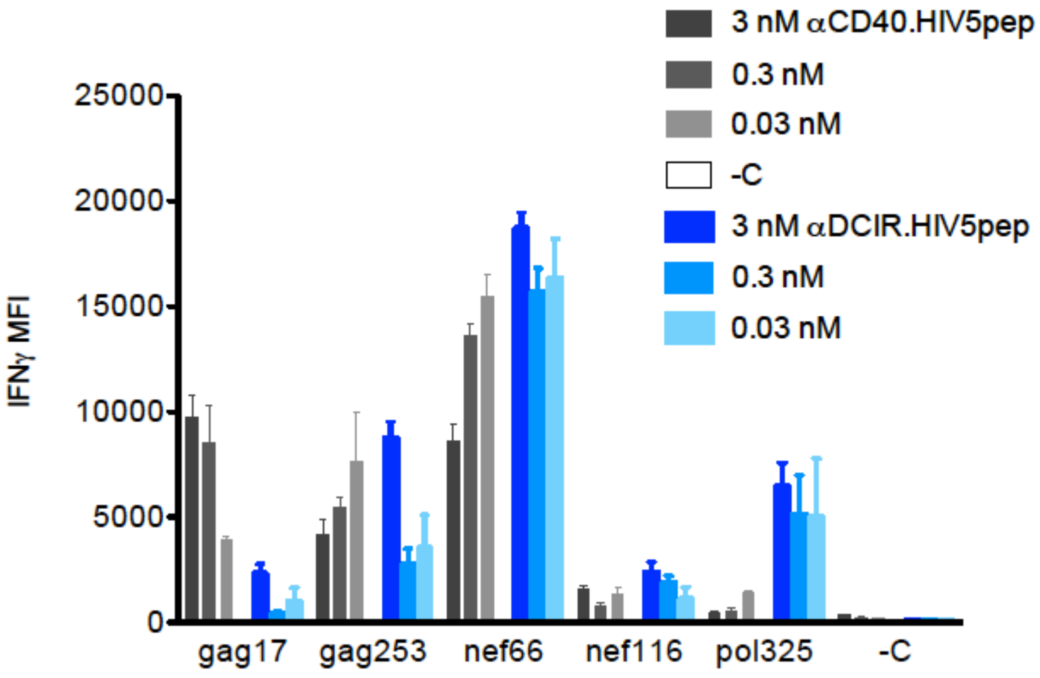

S2 Fig. Humanized αCD40 HIV5pep and αDCIR HIV5pep vaccines expand a similar range of HIV-1-specific T cells. PBMCs from an HIV-1-infected individual were cultured for 10 days with a dose range from 30 pM to 3 nM of αCD40.HIV5pep (black-grey filled bars), 30 pM to 3 nM of αDCIR.HIV5pep) dark blue-light blue bars), or left unstimulated and then restimulated (C-) for 48 hours with or without 19-32 residue long peptides covering the specified HIV-1 Gag, Nef and Pol long peptide regions. The culture supernatants were then harvested and the total T cell secreted IFNγ was analyzed by multiplex bead-based assay. The error bars are the standard error of the mean of replicates.
