# Supplementary material for: HIV-1 T cell epitopes targeted to Rhesus macaque CD40 and DCIR: A comparative study of prototype dendritic cell targeting therapeutic vaccine candidates
Source: PLoS One. 2018 Nov 30;13(11):e0207794. doi: 10.1371/journal.pone.0207794 (PMC6267996; doi:10.1371/journal.pone.0207794)
Supplement: S3 Fig — These data are for weeks 0–16 in G1 and G2, and for weeks 0–14 in G3 and G4. Vaccine or vaccine inj refer to administration of the DC-targeting vaccines with adjuvant. The raw data for this graph is contained in S6 Table. (PDF) [file pone.0207794.s003.pdf]

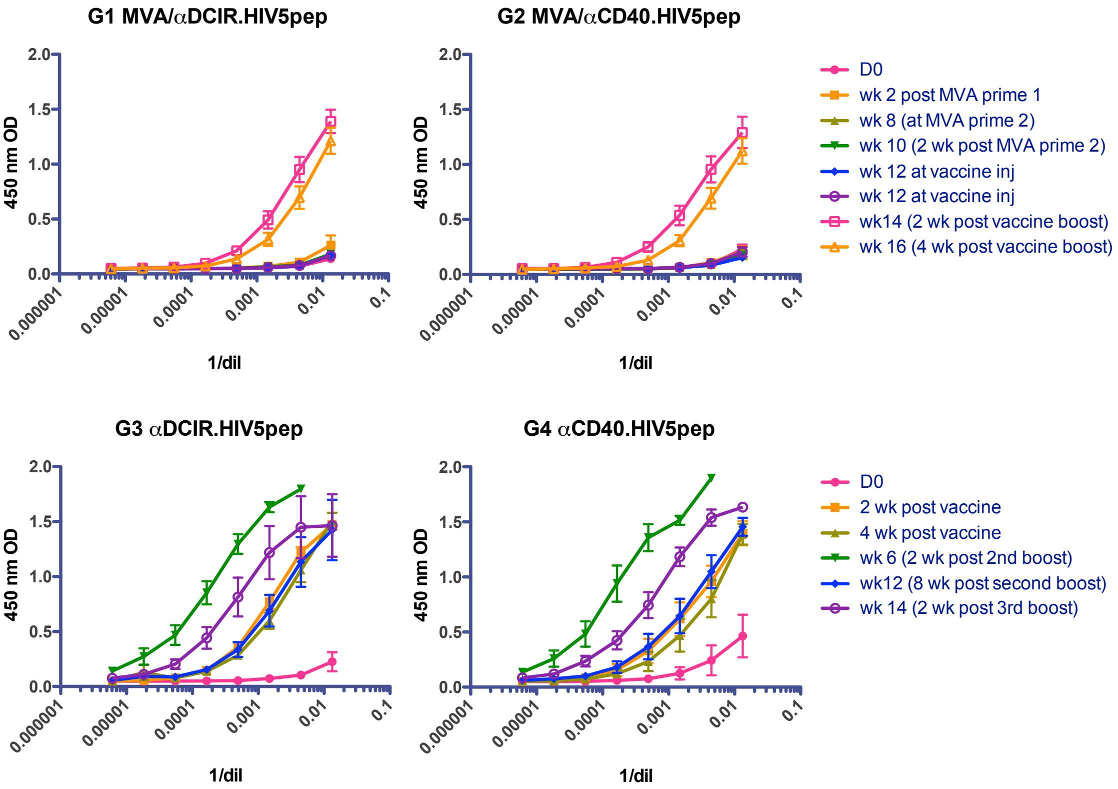

S3 Fig. Titration curves used for the calculation of the serum antibody response presented in Fig 6. These data are for weeks 0-16 in G1 and G2, and for weeks 0-14 in G3 and G4. Vaccine or vaccine inj refer to administration of the DC-targeting vaccines with adjuvant. The raw data for this graph is contained in S6 Table**.**
