# Supplementary material for: HIV-1 T cell epitopes targeted to Rhesus macaque CD40 and DCIR: A comparative study of prototype dendritic cell targeting therapeutic vaccine candidates
Source: PLoS One. 2018 Nov 30;13(11):e0207794. doi: 10.1371/journal.pone.0207794 (PMC6267996; doi:10.1371/journal.pone.0207794)
Supplement: S4 Fig — Cryopreserved cells were stimulated with peptide pools (2 μg/ml) in the presence of Brefeldin A for 6 h. Intracellular cytokine staining was performed (see Methods) and anyzed by flow cytometry. Upper two panels are CD4+ T cells and the lowe two panels are CD8+ T cells. The annotated quadrants indicate the gates used to quantify % cytokine positve cells. (PDF) [file pone.0207794.s004.pdf]

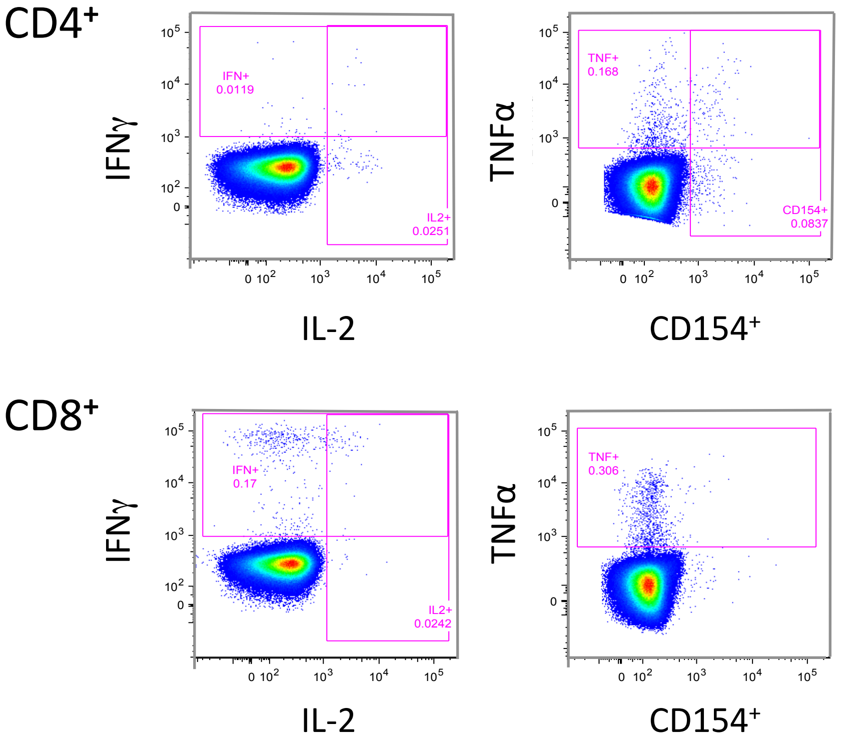

S4 Fig. Flow cytometric analysis of intracellular staining analysis for Gag p24-specific IL-2, TNFα and IFNγ-producing CD4^{+} and CD8^{+} T cells. Cryopreserved cells were stimulated with peptide pools (2 µg/ml) in the presence of Brefeldin A for 6 h. Intracellular cytokine staining was performed (see Methods) and anyzed by flow cytometry. Upper two panels are CD4^{+} T cells and the lowe two panels are CD8^{+} T cells. The annotated quadrants indicate the gates used to quantify % cytokine positive cells.
