# Supplementary material for: HIV-1 T cell epitopes targeted to Rhesus macaque CD40 and DCIR: A comparative study of prototype dendritic cell targeting therapeutic vaccine candidates
Source: PLoS One. 2018 Nov 30;13(11):e0207794. doi: 10.1371/journal.pone.0207794 (PMC6267996; doi:10.1371/journal.pone.0207794)
Supplement: S1 Table — This table is the data that relates to Fig 2 (G1 and G2) and Fig 3 (G3 and G4) panels A and B. Animal name, group, DC-targeting peptides or MVA-specific peptides and sample time in weeks are identified. The values are the sum of spots for each peptide set. (PDF) [file pone.0207794.s005.pdf]

S1 Table. IFNγ ELISPOT data using pools of overlapping Gag, Pol and Nef peptides corresponding to sequences carried by the DC-targeting vector or specifically by the MVA vector. This table is the data that relates to Fig 2 (G1 and G2) and Fig 3 (G3 and G4) panels A and B. Animal name, group, DC-targeting peptides or MVA-specific peptides and sample time in weeks are identified. The values are the sum of spots for each peptide set.

Animal	Group	Type of peptides	Sample Time	Spots per 1^E6
R370	G1 MVA DCIR	DC-Targeting peptites	Wk0	0
R371	G1 MVA DCIR	DC-Targeting peptites	Wk0	10
R373	G1 MVA DCIR	DC-Targeting peptites	Wk0	25
R374	G1 MVA DCIR	DC-Targeting peptites	Wk0	40
R369	G1 MVA DCIR	DC-Targeting peptites	Wk0	15
R385	G1 MVA DCIR	DC-Targeting peptites	Wk0	5
R370	G1 MVA DCIR	Non DC-Targeting peptites	Wk0	0
R371	G1 MVA DCIR	Non DC-Targeting peptites	Wk0	20
R373	G1 MVA DCIR	Non DC-Targeting peptites	Wk0	30
R374	G1 MVA DCIR	Non DC-Targeting peptites	Wk0	40
R369	G1 MVA DCIR	Non DC-Targeting peptites	Wk0	10
R385	G1 MVA DCIR	Non DC-Targeting peptites	Wk0	5
R370	G1 MVA DCIR	DC-Targeting peptites	Wk2	0
R371	G1 MVA DCIR	DC-Targeting peptites	Wk2	45
R373	G1 MVA DCIR	DC-Targeting peptites	Wk2	0
R374	G1 MVA DCIR	DC-Targeting peptites	Wk2	30
R369	G1 MVA DCIR	DC-Targeting peptites	Wk2	120
R385	G1 MVA DCIR	DC-Targeting peptites	Wk2	25
R370	G1 MVA DCIR	Non DC-Targeting peptites	Wk2	5
R371	G1 MVA DCIR	Non DC-Targeting peptites	Wk2	5
R373	G1 MVA DCIR	Non DC-Targeting peptites	Wk2	15
R374	G1 MVA DCIR	Non DC-Targeting peptites	Wk2	45
R369	G1 MVA DCIR	Non DC-Targeting peptites	Wk2	170
R385	G1 MVA DCIR	Non DC-Targeting peptites	Wk2	5
R370	G1 MVA DCIR	DC-Targeting peptites	Wk10	15
R371	G1 MVA DCIR	DC-Targeting peptites	Wk10	20
R373	G1 MVA DCIR	DC-Targeting peptites	Wk10	35
R374	G1 MVA DCIR	DC-Targeting peptites	Wk10	50
R369	G1 MVA DCIR	DC-Targeting peptites	Wk10	65

R385	G1 MVA DCIR	DC-Targeting peptites	Wk10	30
R370	G1 MVA DCIR	Non DC-Targeting peptites	Wk10	15
R371	G1 MVA DCIR	Non DC-Targeting peptites	Wk10	200
R373	G1 MVA DCIR	Non DC-Targeting peptites	Wk10	30
R374	G1 MVA DCIR	Non DC-Targeting peptites	Wk10	95
R369	G1 MVA DCIR	Non DC-Targeting peptites	Wk10	160
R385	G1 MVA DCIR	Non DC-Targeting peptites	Wk10	115
R370	G1 MVA DCIR	DC-Targeting peptites	Wk12	20
R371	G1 MVA DCIR	DC-Targeting peptites	Wk12	25
R373	G1 MVA DCIR	DC-Targeting peptites	Wk12	25
R374	G1 MVA DCIR	DC-Targeting peptites	Wk12	40
R369	G1 MVA DCIR	DC-Targeting peptites	Wk12	10
R385	G1 MVA DCIR	DC-Targeting peptites	Wk12	10
R370	G1 MVA DCIR	Non DC-Targeting peptites	Wk12	25
R371	G1 MVA DCIR	Non DC-Targeting peptites	Wk12	105
R373	G1 MVA DCIR	Non DC-Targeting peptites	Wk12	90
R374	G1 MVA DCIR	Non DC-Targeting peptites	Wk12	55
R369	G1 MVA DCIR	Non DC-Targeting peptites	Wk12	15
R385	G1 MVA DCIR	Non DC-Targeting peptites	Wk12	20
R370	G1 MVA DCIR	DC-Targeting peptites	Wk14	20
R371	G1 MVA DCIR	DC-Targeting peptites	Wk14	190
R373	G1 MVA DCIR	DC-Targeting peptites	Wk14	20
R374	G1 MVA DCIR	DC-Targeting peptites	Wk14	320
R369	G1 MVA DCIR	DC-Targeting peptites	Wk14	605
R385	G1 MVA DCIR	DC-Targeting peptites	Wk14	55
R370	G1 MVA DCIR	Non DC-Targeting peptites	Wk14	25
R371	G1 MVA DCIR	Non DC-Targeting peptites	Wk14	40
R373	G1 MVA DCIR	Non DC-Targeting peptites	Wk14	10
R374	G1 MVA DCIR	Non DC-Targeting peptites	Wk14	60
R369	G1 MVA DCIR	Non DC-Targeting peptites	Wk14	50
R385	G1 MVA DCIR	Non DC-Targeting peptites	Wk14	15
R370	G1 MVA DCIR	DC-Targeting peptites	Wk18	55
R371	G1 MVA DCIR	DC-Targeting peptites	Wk18	560
R373	G1 MVA DCIR	DC-Targeting peptites	Wk18	130
R374	G1 MVA DCIR	DC-Targeting peptites	Wk18	380
R369	G1 MVA DCIR	DC-Targeting peptites	Wk18	1640
R385	G1 MVA DCIR	DC-Targeting peptites	Wk18	670
R370	G1 MVA DCIR	Non DC-Targeting peptites	Wk18	0
R371	G1 MVA DCIR	Non DC-Targeting peptites	Wk18	55
R373	G1 MVA DCIR	Non DC-Targeting peptites	Wk18	15
R374	G1 MVA DCIR	Non DC-Targeting peptites	Wk18	10
R369	G1 MVA DCIR	Non DC-Targeting peptites	Wk18	150
R385	G1 MVA DCIR	Non DC-Targeting peptites	Wk18	50

R370	G1 MVA DCIR	DC-Targeting peptites	Wk26	70
R371	G1 MVA DCIR	DC-Targeting peptites	Wk26	1000
R373	G1 MVA DCIR	DC-Targeting peptites	Wk26	150
R374	G1 MVA DCIR	DC-Targeting peptites	Wk26	240
R369	G1 MVA DCIR	DC-Targeting peptites	Wk26	950
R385	G1 MVA DCIR	DC-Targeting peptites	Wk26	475
R370	G1 MVA DCIR	Non DC-Targeting peptites	Wk26	0
R371	G1 MVA DCIR	Non DC-Targeting peptites	Wk26	105
R373	G1 MVA DCIR	Non DC-Targeting peptites	Wk26	20
R374	G1 MVA DCIR	Non DC-Targeting peptites	Wk26	20
R369	G1 MVA DCIR	Non DC-Targeting peptites	Wk26	120
R385	G1 MVA DCIR	Non DC-Targeting peptites	Wk26	40
R370	G1 MVA DCIR	DC-Targeting peptites	Wk28	75
R371	G1 MVA DCIR	DC-Targeting peptites	Wk28	715
R373	G1 MVA DCIR	DC-Targeting peptites	Wk28	130
R374	G1 MVA DCIR	DC-Targeting peptites	Wk28	485
R369	G1 MVA DCIR	DC-Targeting peptites	Wk28	960
R385	G1 MVA DCIR	DC-Targeting peptites	Wk28	450
R370	G1 MVA DCIR	Non DC-Targeting peptites	Wk28	30
R371	G1 MVA DCIR	Non DC-Targeting peptites	Wk28	85
R373	G1 MVA DCIR	Non DC-Targeting peptites	Wk28	15
R374	G1 MVA DCIR	Non DC-Targeting peptites	Wk28	50
R369	G1 MVA DCIR	Non DC-Targeting peptites	Wk28	55
R385	G1 MVA DCIR	Non DC-Targeting peptites	Wk28	65
R376	G2 MVA CD40	DC-Targeting peptites	Wk0	0
R377	G2 MVA CD40	DC-Targeting peptites	Wk0	60
R378	G2 MVA CD40	DC-Targeting peptites	Wk0	15
R380	G2 MVA CD40	DC-Targeting peptites	Wk0	15
R382	G2 MVA CD40	DC-Targeting peptites	Wk0	5
R384	G2 MVA CD40	DC-Targeting peptites	Wk0	0
R376	G2 MVA CD40	Non DC-Targeting peptites	Wk0	5
R377	G2 MVA CD40	Non DC-Targeting peptites	Wk0	70
R378	G2 MVA CD40	Non DC-Targeting peptites	Wk0	35
R380	G2 MVA CD40	Non DC-Targeting peptites	Wk0	0
R382	G2 MVA CD40	Non DC-Targeting peptites	Wk0	15
R384	G2 MVA CD40	Non DC-Targeting peptites	Wk0	5
R376	G2 MVA CD40	DC-Targeting peptites	Wk2	25
R377	G2 MVA CD40	DC-Targeting peptites	Wk2	285
R378	G2 MVA CD40	DC-Targeting peptites	Wk2	0
R380	G2 MVA CD40	DC-Targeting peptites	Wk2	10
R382	G2 MVA CD40	DC-Targeting peptites	Wk2	145
R384	G2 MVA CD40	DC-Targeting peptites	Wk2	40
R376	G2 MVA CD40	Non DC-Targeting peptites	Wk2	35

R377	G2 MVA CD40	Non DC-Targeting peptites	Wk2	20
R378	G2 MVA CD40	Non DC-Targeting peptites	Wk2	0
R380	G2 MVA CD40	Non DC-Targeting peptites	Wk2	0
R382	G2 MVA CD40	Non DC-Targeting peptites	Wk2	120
R384	G2 MVA CD40	Non DC-Targeting peptites	Wk2	25
R376	G2 MVA CD40	DC-Targeting peptites	Wk10	0
R377	G2 MVA CD40	DC-Targeting peptites	Wk10	85
R378	G2 MVA CD40	DC-Targeting peptites	Wk10	20
R380	G2 MVA CD40	DC-Targeting peptites	Wk10	50
R382	G2 MVA CD40	DC-Targeting peptites	Wk10	45
R384	G2 MVA CD40	DC-Targeting peptites	Wk10	0
R376	G2 MVA CD40	Non DC-Targeting peptites	Wk10	140
R377	G2 MVA CD40	Non DC-Targeting peptites	Wk10	45
R378	G2 MVA CD40	Non DC-Targeting peptites	Wk10	45
R380	G2 MVA CD40	Non DC-Targeting peptites	Wk10	85
R382	G2 MVA CD40	Non DC-Targeting peptites	Wk10	0
R384	G2 MVA CD40	Non DC-Targeting peptites	Wk10	10
R376	G2 MVA CD40	DC-Targeting peptites	Wk12	15
R377	G2 MVA CD40	DC-Targeting peptites	Wk12	10
R378	G2 MVA CD40	DC-Targeting peptites	Wk12	0
R380	G2 MVA CD40	DC-Targeting peptites	Wk12	10
R382	G2 MVA CD40	DC-Targeting peptites	Wk12	135
R384	G2 MVA CD40	DC-Targeting peptites	Wk12	130
R376	G2 MVA CD40	Non DC-Targeting peptites	Wk12	100
R377	G2 MVA CD40	Non DC-Targeting peptites	Wk12	20
R378	G2 MVA CD40	Non DC-Targeting peptites	Wk12	0
R380	G2 MVA CD40	Non DC-Targeting peptites	Wk12	95
R382	G2 MVA CD40	Non DC-Targeting peptites	Wk12	60
R384	G2 MVA CD40	Non DC-Targeting peptites	Wk12	45
R376	G2 MVA CD40	DC-Targeting peptites	Wk14	85
R377	G2 MVA CD40	DC-Targeting peptites	Wk14	1060
R378	G2 MVA CD40	DC-Targeting peptites	Wk14	180
R380	G2 MVA CD40	DC-Targeting peptites	Wk14	385
R382	G2 MVA CD40	DC-Targeting peptites	Wk14	510
R384	G2 MVA CD40	DC-Targeting peptites	Wk14	275
R376	G2 MVA CD40	Non DC-Targeting peptites	Wk14	50
R377	G2 MVA CD40	Non DC-Targeting peptites	Wk14	0
R378	G2 MVA CD40	Non DC-Targeting peptites	Wk14	20
R380	G2 MVA CD40	Non DC-Targeting peptites	Wk14	0
R382	G2 MVA CD40	Non DC-Targeting peptites	Wk14	15
R384	G2 MVA CD40	Non DC-Targeting peptites	Wk14	40
R376	G2 MVA CD40	DC-Targeting peptites	Wk18	440
R377	G2 MVA CD40	DC-Targeting peptites	Wk18	1815

R378	G2 MVA CD40	DC-Targeting peptites	Wk18	1060
R380	G2 MVA CD40	DC-Targeting peptites	Wk18	925
R382	G2 MVA CD40	DC-Targeting peptites	Wk18	1035
R384	G2 MVA CD40	DC-Targeting peptites	Wk18	405
R376	G2 MVA CD40	Non DC-Targeting peptites	Wk18	55
R377	G2 MVA CD40	Non DC-Targeting peptites	Wk18	30
R378	G2 MVA CD40	Non DC-Targeting peptites	Wk18	80
R380	G2 MVA CD40	Non DC-Targeting peptites	Wk18	95
R382	G2 MVA CD40	Non DC-Targeting peptites	Wk18	35
R384	G2 MVA CD40	Non DC-Targeting peptites	Wk18	80
R376	G2 MVA CD40	DC-Targeting peptites	Wk26	375
R377	G2 MVA CD40	DC-Targeting peptites	Wk26	1560
R378	G2 MVA CD40	DC-Targeting peptites	Wk26	435
R380	G2 MVA CD40	DC-Targeting peptites	Wk26	355
R382	G2 MVA CD40	DC-Targeting peptites	Wk26	720
R384	G2 MVA CD40	DC-Targeting peptites	Wk26	580
R376	G2 MVA CD40	Non DC-Targeting peptites	Wk26	90
R377	G2 MVA CD40	Non DC-Targeting peptites	Wk26	140
R378	G2 MVA CD40	Non DC-Targeting peptites	Wk26	25
R380	G2 MVA CD40	Non DC-Targeting peptites	Wk26	55
R382	G2 MVA CD40	Non DC-Targeting peptites	Wk26	10
R384	G2 MVA CD40	Non DC-Targeting peptites	Wk26	20
R376	G2 MVA CD40	DC-Targeting peptites	Wk28	115
R377	G2 MVA CD40	DC-Targeting peptites	Wk28	1555
R378	G2 MVA CD40	DC-Targeting peptites	Wk28	400
R380	G2 MVA CD40	DC-Targeting peptites	Wk28	725
R382	G2 MVA CD40	DC-Targeting peptites	Wk28	770
R384	G2 MVA CD40	DC-Targeting peptites	Wk28	1015
R376	G2 MVA CD40	Non DC-Targeting peptites	Wk28	25
R377	G2 MVA CD40	Non DC-Targeting peptites	Wk28	230
R378	G2 MVA CD40	Non DC-Targeting peptites	Wk28	60
R380	G2 MVA CD40	Non DC-Targeting peptites	Wk28	40
R382	G2 MVA CD40	Non DC-Targeting peptites	Wk28	65
R384	G2 MVA CD40	Non DC-Targeting peptites	Wk28	10
R368	G3 DCIR MVA	DC-Targeting peptites	Wk0	5
R375	G3 DCIR MVA	DC-Targeting peptites	Wk0	0
R379	G3 DCIR MVA	DC-Targeting peptites	Wk0	10
R381	G3 DCIR MVA	DC-Targeting peptites	Wk0	155
R387	G3 DCIR MVA	DC-Targeting peptites	Wk0	0
R388	G3 DCIR MVA	DC-Targeting peptites	Wk0	130
R368	G3 DCIR MVA	Non DC-Targeting peptites	Wk0	25
R375	G3 DCIR MVA	Non DC-Targeting peptites	Wk0	0
R379	G3 DCIR MVA	Non DC-Targeting peptites	Wk0	10

R381	G3 DCIR MVA	Non DC-Targeting peptites	Wk0	0
R387	G3 DCIR MVA	Non DC-Targeting peptites	Wk0	0
R388	G3 DCIR MVA	Non DC-Targeting peptites	Wk0	0
R368	G3 DCIR MVA	DC-Targeting peptites	Wk2	0
R375	G3 DCIR MVA	DC-Targeting peptites	Wk2	60
R379	G3 DCIR MVA	DC-Targeting peptites	Wk2	20
R381	G3 DCIR MVA	DC-Targeting peptites	Wk2	60
R387	G3 DCIR MVA	DC-Targeting peptites	Wk2	10
R388	G3 DCIR MVA	DC-Targeting peptites	Wk2	45
R368	G3 DCIR MVA	Non DC-Targeting peptites	Wk2	0
R375	G3 DCIR MVA	Non DC-Targeting peptites	Wk2	35
R379	G3 DCIR MVA	Non DC-Targeting peptites	Wk2	15
R381	G3 DCIR MVA	Non DC-Targeting peptites	Wk2	50
R387	G3 DCIR MVA	Non DC-Targeting peptites	Wk2	0
R388	G3 DCIR MVA	Non DC-Targeting peptites	Wk2	5
R368	G3 DCIR MVA	DC-Targeting peptites	Wk6	60
R375	G3 DCIR MVA	DC-Targeting peptites	Wk6	150
R379	G3 DCIR MVA	DC-Targeting peptites	Wk6	455
R381	G3 DCIR MVA	DC-Targeting peptites	Wk6	280
R387	G3 DCIR MVA	DC-Targeting peptites	Wk6	40
R388	G3 DCIR MVA	DC-Targeting peptites	Wk6	45
R368	G3 DCIR MVA	Non DC-Targeting peptites	Wk6	5
R375	G3 DCIR MVA	Non DC-Targeting peptites	Wk6	55
R379	G3 DCIR MVA	Non DC-Targeting peptites	Wk6	85
R381	G3 DCIR MVA	Non DC-Targeting peptites	Wk6	1
R387	G3 DCIR MVA	Non DC-Targeting peptites	Wk6	0
R388	G3 DCIR MVA	Non DC-Targeting peptites	Wk6	15
R368	G3 DCIR MVA	DC-Targeting peptites	Wk14	110
R375	G3 DCIR MVA	DC-Targeting peptites	Wk14	425
R379	G3 DCIR MVA	DC-Targeting peptites	Wk14	595
R381	G3 DCIR MVA	DC-Targeting peptites	Wk14	155
R387	G3 DCIR MVA	DC-Targeting peptites	Wk14	0
R388	G3 DCIR MVA	DC-Targeting peptites	Wk14	140
R368	G3 DCIR MVA	Non DC-Targeting peptites	Wk14	15
R375	G3 DCIR MVA	Non DC-Targeting peptites	Wk14	45
R379	G3 DCIR MVA	Non DC-Targeting peptites	Wk14	10
R381	G3 DCIR MVA	Non DC-Targeting peptites	Wk14	0
R387	G3 DCIR MVA	Non DC-Targeting peptites	Wk14	0
R388	G3 DCIR MVA	Non DC-Targeting peptites	Wk14	0
R368	G3 DCIR MVA	DC-Targeting peptites	Wk22	100
R375	G3 DCIR MVA	DC-Targeting peptites	Wk22	280
R379	G3 DCIR MVA	DC-Targeting peptites	Wk22	765
R381	G3 DCIR MVA	DC-Targeting peptites	Wk22	210

R387	G3 DCIR MVA	DC-Targeting peptites	Wk22	0
R388	G3 DCIR MVA	DC-Targeting peptites	Wk22	90
R368	G3 DCIR MVA	Non DC-Targeting peptites	Wk22	80
R375	G3 DCIR MVA	Non DC-Targeting peptites	Wk22	25
R379	G3 DCIR MVA	Non DC-Targeting peptites	Wk22	30
R381	G3 DCIR MVA	Non DC-Targeting peptites	Wk22	20
R387	G3 DCIR MVA	Non DC-Targeting peptites	Wk22	0
R388	G3 DCIR MVA	Non DC-Targeting peptites	Wk22	25
R368	G3 DCIR MVA	DC-Targeting peptites	Wk24	65
R375	G3 DCIR MVA	DC-Targeting peptites	Wk24	310
R379	G3 DCIR MVA	DC-Targeting peptites	Wk24	475
R381	G3 DCIR MVA	DC-Targeting peptites	Wk24	300
R387	G3 DCIR MVA	DC-Targeting peptites	Wk24	0
R388	G3 DCIR MVA	DC-Targeting peptites	Wk24	35
R368	G3 DCIR MVA	Non DC-Targeting peptites	Wk24	5
R375	G3 DCIR MVA	Non DC-Targeting peptites	Wk24	20
R379	G3 DCIR MVA	Non DC-Targeting peptites	Wk24	10
R381	G3 DCIR MVA	Non DC-Targeting peptites	Wk24	55
R387	G3 DCIR MVA	Non DC-Targeting peptites	Wk24	0
R388	G3 DCIR MVA	Non DC-Targeting peptites	Wk24	10
R368	G3 DCIR MVA	DC-Targeting peptites	Wk26	20
R375	G3 DCIR MVA	DC-Targeting peptites	Wk26	175
R379	G3 DCIR MVA	DC-Targeting peptites	Wk26	570
R381	G3 DCIR MVA	DC-Targeting peptites	Wk26	120
R387	G3 DCIR MVA	DC-Targeting peptites	Wk26	0
R388	G3 DCIR MVA	DC-Targeting peptites	Wk26	35
R368	G3 DCIR MVA	Non DC-Targeting peptites	Wk26	10
R375	G3 DCIR MVA	Non DC-Targeting peptites	Wk26	15
R379	G3 DCIR MVA	Non DC-Targeting peptites	Wk26	35
R381	G3 DCIR MVA	Non DC-Targeting peptites	Wk26	25
R387	G3 DCIR MVA	Non DC-Targeting peptites	Wk26	0
R388	G3 DCIR MVA	Non DC-Targeting peptites	Wk26	0
R386	G4 CD40 MVA	DC-Targeting peptites	Wk0	10
R389	G4 CD40 MVA	DC-Targeting peptites	Wk0	5
R390	G4 CD40 MVA	DC-Targeting peptites	Wk0	20
R391	G4 CD40 MVA	DC-Targeting peptites	Wk0	35
R372	G4 CD40 MVA	DC-Targeting peptites	Wk0	5
R383	G4 CD40 MVA	DC-Targeting peptites	Wk0	0
R386	G4 CD40 MVA	Non DC-Targeting peptites	Wk0	10
R389	G4 CD40 MVA	Non DC-Targeting peptites	Wk0	5
R390	G4 CD40 MVA	Non DC-Targeting peptites	Wk0	30
R391	G4 CD40 MVA	Non DC-Targeting peptites	Wk0	30
R372	G4 CD40 MVA	Non DC-Targeting peptites	Wk0	5

R383	G4 CD40 MVA	Non DC-Targeting peptites	Wk0	0
R386	G4 CD40 MVA	DC-Targeting peptites	Wk2	95
R389	G4 CD40 MVA	DC-Targeting peptites	Wk2	100
R390	G4 CD40 MVA	DC-Targeting peptites	Wk2	40
R391	G4 CD40 MVA	DC-Targeting peptites	Wk2	365
R372	G4 CD40 MVA	DC-Targeting peptites	Wk2	20
R383	G4 CD40 MVA	DC-Targeting peptites	Wk2	35
R386	G4 CD40 MVA	Non DC-Targeting peptites	Wk2	95
R389	G4 CD40 MVA	Non DC-Targeting peptites	Wk2	55
R390	G4 CD40 MVA	Non DC-Targeting peptites	Wk2	40
R391	G4 CD40 MVA	Non DC-Targeting peptites	Wk2	195
R372	G4 CD40 MVA	Non DC-Targeting peptites	Wk2	10
R383	G4 CD40 MVA	Non DC-Targeting peptites	Wk2	25
R386	G4 CD40 MVA	DC-Targeting peptites	Wk6	80
R389	G4 CD40 MVA	DC-Targeting peptites	Wk6	180
R390	G4 CD40 MVA	DC-Targeting peptites	Wk6	40
R391	G4 CD40 MVA	DC-Targeting peptites	Wk6	175
R372	G4 CD40 MVA	DC-Targeting peptites	Wk6	125
R383	G4 CD40 MVA	DC-Targeting peptites	Wk6	140
R386	G4 CD40 MVA	Non DC-Targeting peptites	Wk6	50
R389	G4 CD40 MVA	Non DC-Targeting peptites	Wk6	95
R390	G4 CD40 MVA	Non DC-Targeting peptites	Wk6	25
R391	G4 CD40 MVA	Non DC-Targeting peptites	Wk6	105
R372	G4 CD40 MVA	Non DC-Targeting peptites	Wk6	60
R383	G4 CD40 MVA	Non DC-Targeting peptites	Wk6	55
R386	G4 CD40 MVA	DC-Targeting peptites	Wk14	155
R389	G4 CD40 MVA	DC-Targeting peptites	Wk14	665
R390	G4 CD40 MVA	DC-Targeting peptites	Wk14	85
R391	G4 CD40 MVA	DC-Targeting peptites	Wk14	100
R372	G4 CD40 MVA	DC-Targeting peptites	Wk14	150
R383	G4 CD40 MVA	DC-Targeting peptites	Wk14	550
R386	G4 CD40 MVA	Non DC-Targeting peptites	Wk14	40
R389	G4 CD40 MVA	Non DC-Targeting peptites	Wk14	45
R390	G4 CD40 MVA	Non DC-Targeting peptites	Wk14	10
R391	G4 CD40 MVA	Non DC-Targeting peptites	Wk14	100
R372	G4 CD40 MVA	Non DC-Targeting peptites	Wk14	5
R383	G4 CD40 MVA	Non DC-Targeting peptites	Wk14	5
R386	G4 CD40 MVA	DC-Targeting peptites	Wk22	130
R389	G4 CD40 MVA	DC-Targeting peptites	Wk22	645
R390	G4 CD40 MVA	DC-Targeting peptites	Wk22	10
R391	G4 CD40 MVA	DC-Targeting peptites	Wk22	25
R372	G4 CD40 MVA	DC-Targeting peptites	Wk22	190
R383	G4 CD40 MVA	DC-Targeting peptites	Wk22	775

R386	G4 CD40 MVA	Non DC-Targeting peptites	Wk22	65
R389	G4 CD40 MVA	Non DC-Targeting peptites	Wk22	5
R390	G4 CD40 MVA	Non DC-Targeting peptites	Wk22	0
R391	G4 CD40 MVA	Non DC-Targeting peptites	Wk22	25
R372	G4 CD40 MVA	Non DC-Targeting peptites	Wk22	15
R383	G4 CD40 MVA	Non DC-Targeting peptites	Wk22	80
R386	G4 CD40 MVA	DC-Targeting peptites	Wk24	210
R389	G4 CD40 MVA	DC-Targeting peptites	Wk24	425
R390	G4 CD40 MVA	DC-Targeting peptites	Wk24	75
R391	G4 CD40 MVA	DC-Targeting peptites	Wk24	120
R372	G4 CD40 MVA	DC-Targeting peptites	Wk24	280
R383	G4 CD40 MVA	DC-Targeting peptites	Wk24	430
R386	G4 CD40 MVA	Non DC-Targeting peptites	Wk24	75
R389	G4 CD40 MVA	Non DC-Targeting peptites	Wk24	75
R390	G4 CD40 MVA	Non DC-Targeting peptites	Wk24	5
R391	G4 CD40 MVA	Non DC-Targeting peptites	Wk24	15
R372	G4 CD40 MVA	Non DC-Targeting peptites	Wk24	55
R383	G4 CD40 MVA	Non DC-Targeting peptites	Wk24	60
R386	G4 CD40 MVA	DC-Targeting peptites	Wk26	95
R389	G4 CD40 MVA	DC-Targeting peptites	Wk26	415
R390	G4 CD40 MVA	DC-Targeting peptites	Wk26	85
R391	G4 CD40 MVA	DC-Targeting peptites	Wk26	90
R372	G4 CD40 MVA	DC-Targeting peptites	Wk26	180
R383	G4 CD40 MVA	DC-Targeting peptites	Wk26	285
R386	G4 CD40 MVA	Non DC-Targeting peptites	Wk26	60
R389	G4 CD40 MVA	Non DC-Targeting peptites	Wk26	80
R390	G4 CD40 MVA	Non DC-Targeting peptites	Wk26	50
R391	G4 CD40 MVA	Non DC-Targeting peptites	Wk26	0
R372	G4 CD40 MVA	Non DC-Targeting peptites	Wk26	0
R383	G4 CD40 MVA	Non DC-Targeting peptites	Wk26	20
