# Supplementary material for: HIV-1 T cell epitopes targeted to Rhesus macaque CD40 and DCIR: A comparative study of prototype dendritic cell targeting therapeutic vaccine candidates
Source: PLoS One. 2018 Nov 30;13(11):e0207794. doi: 10.1371/journal.pone.0207794 (PMC6267996; doi:10.1371/journal.pone.0207794)
Supplement: S2 Table — This table is the data that relates to Fig 2 (G1 and G2) and Fig 3 (G3 and G4) panels C and D. Peptide names, group, and sample time in weeks are identified. The values are the sum of spots for each peptide set. (PDF) [file pone.0207794.s006.pdf]

S2 Table. IFNγ ELISPOT data for individual Gag, Pol and Nef peptide stimulations corresponding to sequence carried by the DC-targeting vectors. This table is the data that relates to Fig 2 (G1 and G2) and Fig 3 (G3 and G4) panels C and D. Peptide names, group, and sample time in weeks are identified. The values are the sum of spots for each peptide set.

peptides	Group	Sample time	% Response
gag253	G1 MVA DCIR	Wk0	7.5
gag253	G2 MVA CD40	Wk0	0
gag253	G3 DCIR MVA	Wk0	5
gag253	G4 CD40 MVA	Wk0	2.5
gag253	G1 MVA DCIR	Wk10	12.5
gag253	G2 MVA CD40	Wk10	0
gag253	G1 MVA DCIR	Wk12	10
gag253	G2 MVA CD40	Wk12	17.5
gag253	G1 MVA DCIR	Wk14	30
gag253	G2 MVA CD40	Wk14	100
gag253	G3 DCIR MVA	Wk14	15
gag253	G4 CD40 MVA	Wk14	57.5
gag253	G1 MVA DCIR	Wk18	162.5
gag253	G2 MVA CD40	Wk18	240
gag253	G1 MVA DCIR	Wk2	2.5
gag253	G2 MVA CD40	Wk2	10
gag253	G3 DCIR MVA	Wk2	10
gag253	G4 CD40 MVA	Wk2	45
gag253	G3 DCIR MVA	Wk22	57.5
gag253	G4 CD40 MVA	Wk22	55
gag253	G1 MVA DCIR	Wk24	142.5
gag253	G2 MVA CD40	Wk24	187.5
gag253	G3 DCIR MVA	Wk24	17.5
gag253	G4 CD40 MVA	Wk24	65
gag253	G3 DCIR MVA	Wk26	17.5
gag253	G4 CD40 MVA	Wk26	70
gag253	G1 MVA DCIR	Wk28	190
gag253	G2 MVA CD40	Wk28	615
gag253	G3 DCIR MVA	Wk6	22.5
gag253	G4 CD40 MVA	Wk6	55
gag17	G1 MVA DCIR	Wk0	2.5
gag17	G2 MVA CD40	Wk0	5

gag17	G3 DCIR MVA	Wk0	0
gag17	G4 CD40 MVA	Wk0	0
gag17	G1 MVA DCIR	Wk10	0
gag17	G2 MVA CD40	Wk10	0
gag17	G1 MVA DCIR	Wk12	0
gag17	G2 MVA CD40	Wk12	5
gag17	G1 MVA DCIR	Wk14	0
gag17	G2 MVA CD40	Wk14	0
gag17	G3 DCIR MVA	Wk14	0
gag17	G4 CD40 MVA	Wk14	10
gag17	G1 MVA DCIR	Wk18	2.5
gag17	G2 MVA CD40	Wk18	10
gag17	G1 MVA DCIR	Wk2	0
gag17	G2 MVA CD40	Wk2	2.5
gag17	G3 DCIR MVA	Wk2	7.5
gag17	G4 CD40 MVA	Wk2	12.5
gag17	G3 DCIR MVA	Wk22	5
gag17	G4 CD40 MVA	Wk22	2.5
gag17	G1 MVA DCIR	Wk24	7.5
gag17	G2 MVA CD40	Wk24	12.5
gag17	G3 DCIR MVA	Wk24	2.5
gag17	G4 CD40 MVA	Wk24	7.5
gag17	G3 DCIR MVA	Wk26	7.5
gag17	G4 CD40 MVA	Wk26	2.5
gag17	G1 MVA DCIR	Wk28	2.5
gag17	G2 MVA CD40	Wk28	2.5
gag17	G3 DCIR MVA	Wk6	82.5
gag17	G4 CD40 MVA	Wk6	42.5
pol158	G1 MVA DCIR	Wk0	5
pol158	G2 MVA CD40	Wk0	10
pol158	G3 DCIR MVA	Wk0	7.5
pol158	G4 CD40 MVA	Wk0	7.5
pol158	G1 MVA DCIR	Wk10	20
pol158	G2 MVA CD40	Wk10	25
pol158	G1 MVA DCIR	Wk12	35
pol158	G2 MVA CD40	Wk12	37.5
pol158	G1 MVA DCIR	Wk14	25
pol158	G2 MVA CD40	Wk14	220
pol158	G3 DCIR MVA	Wk14	175
pol158	G4 CD40 MVA	Wk14	115
pol158	G1 MVA DCIR	Wk18	60
pol158	G2 MVA CD40	Wk18	540
pol158	G1 MVA DCIR	Wk2	7.5

pol158	G2 MVA CD40	Wk2	22.5
pol158	G3 DCIR MVA	Wk2	25
pol158	G4 CD40 MVA	Wk2	32.5
pol158	G3 DCIR MVA	Wk22	172.5
pol158	G4 CD40 MVA	Wk22	210
pol158	G1 MVA DCIR	Wk24	75
pol158	G2 MVA CD40	Wk24	405
pol158	G3 DCIR MVA	Wk24	132.5
pol158	G4 CD40 MVA	Wk24	165
pol158	G3 DCIR MVA	Wk26	210
pol158	G4 CD40 MVA	Wk26	205
pol158	G1 MVA DCIR	Wk28	127.5
pol158	G2 MVA CD40	Wk28	695
pol158	G3 DCIR MVA	Wk6	60
pol158	G4 CD40 MVA	Wk6	42.5
nef66	G1 MVA DCIR	Wk0	0
nef66	G2 MVA CD40	Wk0	0
nef66	G3 DCIR MVA	Wk0	0
nef66	G4 CD40 MVA	Wk0	0
nef66	G1 MVA DCIR	Wk10	2.5
nef66	G2 MVA CD40	Wk10	0
nef66	G1 MVA DCIR	Wk12	0
nef66	G2 MVA CD40	Wk12	5
nef66	G1 MVA DCIR	Wk14	0
nef66	G2 MVA CD40	Wk14	0
nef66	G3 DCIR MVA	Wk14	5
nef66	G4 CD40 MVA	Wk14	0
nef66	G1 MVA DCIR	Wk18	5
nef66	G2 MVA CD40	Wk18	17.5
nef66	G1 MVA DCIR	Wk2	0
nef66	G2 MVA CD40	Wk2	0
nef66	G3 DCIR MVA	Wk2	5
nef66	G4 CD40 MVA	Wk2	7.5
nef66	G3 DCIR MVA	Wk22	7.5
nef66	G4 CD40 MVA	Wk22	2.5
nef66	G1 MVA DCIR	Wk24	0
nef66	G2 MVA CD40	Wk24	22.5
nef66	G3 DCIR MVA	Wk24	2.5
nef66	G4 CD40 MVA	Wk24	5
nef66	G3 DCIR MVA	Wk26	12.5
nef66	G4 CD40 MVA	Wk26	0
nef66	G1 MVA DCIR	Wk28	7.5
nef66	G2 MVA CD40	Wk28	32.5

nef66	G3 DCIR MVA	Wk6	2.5
nef66	G4 CD40 MVA	Wk6	10
nef116	G1 MVA DCIR	Wk0	5
nef116	G2 MVA CD40	Wk0	2.5
nef116	G3 DCIR MVA	Wk0	0
nef116	G4 CD40 MVA	Wk0	2.5
nef116	G1 MVA DCIR	Wk10	0
nef116	G2 MVA CD40	Wk10	0
nef116	G1 MVA DCIR	Wk12	0
nef116	G2 MVA CD40	Wk12	0
nef116	G1 MVA DCIR	Wk14	0
nef116	G2 MVA CD40	Wk14	2.5
nef116	G3 DCIR MVA	Wk14	0
nef116	G4 CD40 MVA	Wk14	0
nef116	G1 MVA DCIR	Wk18	2.5
nef116	G2 MVA CD40	Wk18	15
nef116	G1 MVA DCIR	Wk2	0
nef116	G2 MVA CD40	Wk2	2.5
nef116	G3 DCIR MVA	Wk2	7.5
nef116	G4 CD40 MVA	Wk2	15
nef116	G3 DCIR MVA	Wk22	10
nef116	G4 CD40 MVA	Wk22	2.5
nef116	G1 MVA DCIR	Wk24	0
nef116	G2 MVA CD40	Wk24	15
nef116	G3 DCIR MVA	Wk24	0
nef116	G4 CD40 MVA	Wk24	0
nef116	G3 DCIR MVA	Wk26	0
nef116	G4 CD40 MVA	Wk26	0
nef116	G1 MVA DCIR	Wk28	0
nef116	G2 MVA CD40	Wk28	5
nef116	G3 DCIR MVA	Wk6	17.5
nef116	G4 CD40 MVA	Wk6	22.5
