# Supplementary material for: HIV-1 T cell epitopes targeted to Rhesus macaque CD40 and DCIR: A comparative study of prototype dendritic cell targeting therapeutic vaccine candidates
Source: PLoS One. 2018 Nov 30;13(11):e0207794. doi: 10.1371/journal.pone.0207794 (PMC6267996; doi:10.1371/journal.pone.0207794)
Supplement: S3 Table — This table is the data that relates to S1 Fig. Animal name, group, T cell type and sample time in weeks are identified. The % response values for either HIV-1 antigen-specific CD4+ or CD8+ T cells are the sum of 1 cytokine, 2 cytokines, and three cytokines as determined by the ICS analysis. (PDF) [file pone.0207794.s007.pdf]

S3 Table. Analysis of HIV-1 epitope-specific CD4$^+$ and CD8$^+$ T cell responses elicited in MVA-primed NHPs by αDCIR.HIV5pep and αCD40.HIV5pep vaccines and in naïve NHPs by αDCIR.HIV5pep and αCD40.HIV5pep vaccines. This table is the data that relates to S1 Fig. Animal name, group, T cell type and sample time in weeks are identified. The % response values for either HIV-1 antigen-specific CD4$^+$ or CD8$^+$ T cells are the sum of 1 cytokine, 2 cytokines, and three cytokines as determined by the ICS analysis.

Animal	Group	Sample time	T cell type	Cytokine No.	% Response
R368	G3 DCIR MVA	Wk14	CD4	3 cytokines	0.03074
R368	G3 DCIR MVA	Wk14	CD4	2 cytokines	0.06682
R368	G3 DCIR MVA	Wk14	CD4	1 cytokine	0.13843
R368	G3 DCIR MVA	Wk14	CD8	3 cytokines	0.000
R368	G3 DCIR MVA	Wk14	CD8	2 cytokines	0.05352
R368	G3 DCIR MVA	Wk14	CD8	1 cytokine	0.20025
R368	G3 DCIR MVA	Wk24	CD4	3 cytokines	0.05371
R368	G3 DCIR MVA	Wk24	CD4	2 cytokines	0.09228
R368	G3 DCIR MVA	Wk24	CD4	1 cytokine	0.14663
R368	G3 DCIR MVA	Wk24	CD8	3 cytokines	0.01262
R368	G3 DCIR MVA	Wk24	CD8	2 cytokines	0.09317
R368	G3 DCIR MVA	Wk24	CD8	1 cytokine	0.45628
R369	G1 MVA DCIR	Wk10	CD4	3 cytokines	0.0118
R369	G1 MVA DCIR	Wk10	CD4	2 cytokines	0.01991
R369	G1 MVA DCIR	Wk10	CD4	1 cytokine	0.03261
R369	G1 MVA DCIR	Wk10	CD8	3 cytokines	0.02334
R369	G1 MVA DCIR	Wk10	CD8	2 cytokines	0.13895
R369	G1 MVA DCIR	Wk10	CD8	1 cytokine	0.22342
R369	G1 MVA DCIR	Wk26	CD4	3 cytokines	0.45237
R369	G1 MVA DCIR	Wk26	CD4	2 cytokines	0.59087
R369	G1 MVA DCIR	Wk26	CD4	1 cytokine	0.65393
R369	G1 MVA DCIR	Wk26	CD8	3 cytokines	0.05383
R369	G1 MVA DCIR	Wk26	CD8	2 cytokines	0.17892
R369	G1 MVA DCIR	Wk26	CD8	1 cytokine	0.26124
R370	G1 MVA DCIR	Wk10	CD4	3 cytokines	0.00297
R370	G1 MVA DCIR	Wk10	CD4	2 cytokines	0.00775
R370	G1 MVA DCIR	Wk10	CD4	1 cytokine	0.01605
R370	G1 MVA DCIR	Wk10	CD8	3 cytokines	0.00126
R370	G1 MVA DCIR	Wk10	CD8	2 cytokines	0.00374

R370	G1 MVA DCIR	Wk10	CD8	1 cytokine	0.06696
R370	G1 MVA DCIR	Wk26	CD4	3 cytokines	0.01572
R370	G1 MVA DCIR	Wk26	CD4	2 cytokines	0.03512
R370	G1 MVA DCIR	Wk26	CD4	1 cytokine	0.05185
R370	G1 MVA DCIR	Wk26	CD8	3 cytokines	0.00169
R370	G1 MVA DCIR	Wk26	CD8	2 cytokines	0.03676
R370	G1 MVA DCIR	Wk26	CD8	1 cytokine	0.2566
R371	G1 MVA DCIR	Wk10	CD4	3 cytokines	0.00772
R371	G1 MVA DCIR	Wk10	CD4	2 cytokines	0.02508
R371	G1 MVA DCIR	Wk10	CD4	1 cytokine	0.03256
R371	G1 MVA DCIR	Wk10	CD8	3 cytokines	0.04259
R371	G1 MVA DCIR	Wk10	CD8	2 cytokines	0.15067
R371	G1 MVA DCIR	Wk10	CD8	1 cytokine	0.22197
R371	G1 MVA DCIR	Wk26	CD4	3 cytokines	0.09251
R371	G1 MVA DCIR	Wk26	CD4	2 cytokines	0.19856
R371	G1 MVA DCIR	Wk26	CD4	1 cytokine	0.23085
R371	G1 MVA DCIR	Wk26	CD8	3 cytokines	0.01922
R371	G1 MVA DCIR	Wk26	CD8	2 cytokines	0.07473
R371	G1 MVA DCIR	Wk26	CD8	1 cytokine	0.13913
R372	G4 CD40 MVA	Wk14	CD4	1 cytokine	0.03464
R372	G4 CD40 MVA	Wk14	CD4	2 cytokines	0.06996
R372	G4 CD40 MVA	Wk14	CD4	3 cytokines	0.10164
R372	G4 CD40 MVA	Wk14	CD8	1 cytokine	0.00383
R372	G4 CD40 MVA	Wk14	CD8	2 cytokines	0.06095
R372	G4 CD40 MVA	Wk14	CD8	3 cytokines	0.13539
R372	G4 CD40 MVA	Wk24	CD4	1 cytokine	0.16168
R372	G4 CD40 MVA	Wk24	CD4	2 cytokines	0.24919
R372	G4 CD40 MVA	Wk24	CD4	3 cytokines	0.28593
R372	G4 CD40 MVA	Wk24	CD8	1 cytokine	0.02015
R372	G4 CD40 MVA	Wk24	CD8	2 cytokines	0.11009
R372	G4 CD40 MVA	Wk24	CD8	3 cytokines	0.14294
R373	G1 MVA DCIR	Wk10	CD4	3 cytokines	0.00168
R373	G1 MVA DCIR	Wk10	CD4	2 cytokines	0.01105
R373	G1 MVA DCIR	Wk10	CD4	1 cytokine	0.02048
R373	G1 MVA DCIR	Wk10	CD8	3 cytokines	0.00535
R373	G1 MVA DCIR	Wk10	CD8	2 cytokines	0.02438
R373	G1 MVA DCIR	Wk10	CD8	1 cytokine	0.11029
R373	G1 MVA DCIR	Wk26	CD4	3 cytokines	0.07056
R373	G1 MVA DCIR	Wk26	CD4	2 cytokines	0.1113
R373	G1 MVA DCIR	Wk26	CD4	1 cytokine	0.13659
R373	G1 MVA DCIR	Wk26	CD8	3 cytokines	0.00516
R373	G1 MVA DCIR	Wk26	CD8	2 cytokines	0.01574
R373	G1 MVA DCIR	Wk26	CD8	1 cytokine	0.05849
R374	G1 MVA DCIR	Wk10	CD4	3 cytokines	0.01825
R374	G1 MVA DCIR	Wk10	CD4	2 cytokines	0.02705
R374	G1 MVA DCIR	Wk10	CD4	1 cytokine	0.03334

R374	G1 MVA DCIR	Wk10	CD8	3 cytokines	0.00984
R374	G1 MVA DCIR	Wk10	CD8	2 cytokines	0.05077
R374	G1 MVA DCIR	Wk10	CD8	1 cytokine	0.12235
R374	G1 MVA DCIR	Wk26	CD4	3 cytokines	0.25642
R374	G1 MVA DCIR	Wk26	CD4	2 cytokines	0.3638
R374	G1 MVA DCIR	Wk26	CD4	1 cytokine	0.39763
R374	G1 MVA DCIR	Wk26	CD8	3 cytokines	0.00877
R374	G1 MVA DCIR	Wk26	CD8	2 cytokines	0.01649
R374	G1 MVA DCIR	Wk26	CD8	1 cytokine	0.08304
R375	G3 DCIR MVA	Wk14	CD4	3 cytokines	0.01233
R375	G3 DCIR MVA	Wk14	CD4	2 cytokines	0.05096
R375	G3 DCIR MVA	Wk14	CD4	1 cytokine	0.11093
R375	G3 DCIR MVA	Wk14	CD8	3 cytokines	0.000
R375	G3 DCIR MVA	Wk14	CD8	2 cytokines	0.00946
R375	G3 DCIR MVA	Wk14	CD8	1 cytokine	0.19172
R375	G3 DCIR MVA	Wk24	CD4	3 cytokines	0.06256
R375	G3 DCIR MVA	Wk24	CD4	2 cytokines	0.09147
R375	G3 DCIR MVA	Wk24	CD4	1 cytokine	0.11269
R375	G3 DCIR MVA	Wk24	CD8	3 cytokines	0.00044
R375	G3 DCIR MVA	Wk24	CD8	2 cytokines	0.00296
R375	G3 DCIR MVA	Wk24	CD8	1 cytokine	0.04115
R376	G2 MVA CD40	Wk10	CD4	1 cytokine	0.00419
R376	G2 MVA CD40	Wk10	CD4	2 cytokines	0.01604
R376	G2 MVA CD40	Wk10	CD4	3 cytokines	0.03366
R376	G2 MVA CD40	Wk10	CD8	1 cytokine	0.01723
R376	G2 MVA CD40	Wk10	CD8	2 cytokines	0.15902
R376	G2 MVA CD40	Wk10	CD8	3 cytokines	0.18492
R376	G2 MVA CD40	Wk26	CD4	1 cytokine	0.05331
R376	G2 MVA CD40	Wk26	CD4	2 cytokines	0.09442
R376	G2 MVA CD40	Wk26	CD4	3 cytokines	0.11503
R376	G2 MVA CD40	Wk26	CD8	1 cytokine	0.00449
R376	G2 MVA CD40	Wk26	CD8	2 cytokines	0.04321
R376	G2 MVA CD40	Wk26	CD8	3 cytokines	0.07239
R377	G2 MVA CD40	Wk10	CD4	1 cytokine	0.02731
R377	G2 MVA CD40	Wk10	CD4	2 cytokines	0.03412
R377	G2 MVA CD40	Wk10	CD4	3 cytokines	0.08706
R377	G2 MVA CD40	Wk10	CD8	1 cytokine	0.07005
R377	G2 MVA CD40	Wk10	CD8	2 cytokines	0.16465
R377	G2 MVA CD40	Wk10	CD8	3 cytokines	0.24485
R377	G2 MVA CD40	Wk26	CD4	1 cytokine	0.73122
R377	G2 MVA CD40	Wk26	CD4	2 cytokines	1.0375
R377	G2 MVA CD40	Wk26	CD4	3 cytokines	1.12712
R377	G2 MVA CD40	Wk26	CD8	1 cytokine	0.03289
R377	G2 MVA CD40	Wk26	CD8	2 cytokines	0.06683
R377	G2 MVA CD40	Wk26	CD8	3 cytokines	0.19642
R378	G2 MVA CD40	Wk10	CD4	1 cytokine	0.00734

R378	G2 MVA CD40	Wk10	CD4	2 cytokines	0.01112
R378	G2 MVA CD40	Wk10	CD4	3 cytokines	0.01993
R378	G2 MVA CD40	Wk10	CD8	1 cytokine	0.01253
R378	G2 MVA CD40	Wk10	CD8	2 cytokines	0.03976
R378	G2 MVA CD40	Wk10	CD8	3 cytokines	0.10622
R378	G2 MVA CD40	Wk26	CD4	1 cytokine	0.22588
R378	G2 MVA CD40	Wk26	CD4	2 cytokines	0.33796
R378	G2 MVA CD40	Wk26	CD4	3 cytokines	0.40142
R378	G2 MVA CD40	Wk26	CD8	1 cytokine	0.00309
R378	G2 MVA CD40	Wk26	CD8	2 cytokines	0.00835
R378	G2 MVA CD40	Wk26	CD8	3 cytokines	0.03884
R379	G3 DCIR MVA	Wk14	CD4	3 cytokines	0.0944
R379	G3 DCIR MVA	Wk14	CD4	2 cytokines	0.16628
R379	G3 DCIR MVA	Wk14	CD4	1 cytokine	0.20692
R379	G3 DCIR MVA	Wk14	CD8	3 cytokines	0.0091
R379	G3 DCIR MVA	Wk14	CD8	2 cytokines	0.06072
R379	G3 DCIR MVA	Wk14	CD8	1 cytokine	0.20188
R379	G3 DCIR MVA	Wk24	CD4	3 cytokines	0.20703
R379	G3 DCIR MVA	Wk24	CD4	2 cytokines	0.31013
R379	G3 DCIR MVA	Wk24	CD4	1 cytokine	0.35971
R379	G3 DCIR MVA	Wk24	CD8	3 cytokines	0.02116
R379	G3 DCIR MVA	Wk24	CD8	2 cytokines	0.06479
R379	G3 DCIR MVA	Wk24	CD8	1 cytokine	0.10311
R380	G2 MVA CD40	Wk10	CD4	1 cytokine	0.01559
R380	G2 MVA CD40	Wk10	CD4	2 cytokines	0.04755
R380	G2 MVA CD40	Wk10	CD4	3 cytokines	0.10124
R380	G2 MVA CD40	Wk10	CD8	1 cytokine	0.00575
R380	G2 MVA CD40	Wk10	CD8	2 cytokines	0.01421
R380	G2 MVA CD40	Wk10	CD8	3 cytokines	0.07953
R380	G2 MVA CD40	Wk26	CD4	1 cytokine	0.25071
R380	G2 MVA CD40	Wk26	CD4	2 cytokines	0.38121
R380	G2 MVA CD40	Wk26	CD4	3 cytokines	0.4306
R380	G2 MVA CD40	Wk26	CD8	1 cytokine	0.00467
R380	G2 MVA CD40	Wk26	CD8	2 cytokines	0.02783
R380	G2 MVA CD40	Wk26	CD8	3 cytokines	0.19237
R381	G3 DCIR MVA	Wk14	CD4	3 cytokines	0.0873
R381	G3 DCIR MVA	Wk14	CD4	2 cytokines	0.1443
R381	G3 DCIR MVA	Wk14	CD4	1 cytokine	0.21125
R381	G3 DCIR MVA	Wk14	CD8	3 cytokines	0.000
R381	G3 DCIR MVA	Wk14	CD8	2 cytokines	0.00862
R381	G3 DCIR MVA	Wk14	CD8	1 cytokine	0.09946
R381	G3 DCIR MVA	Wk24	CD4	3 cytokines	0.05399
R381	G3 DCIR MVA	Wk24	CD4	2 cytokines	0.09098
R381	G3 DCIR MVA	Wk24	CD4	1 cytokine	0.13078
R381	G3 DCIR MVA	Wk24	CD8	3 cytokines	0.0025
R381	G3 DCIR MVA	Wk24	CD8	2 cytokines	0.05951

R381	G3 DCIR MVA	Wk24	CD8	1 cytokine	0.12659
R382	G2 MVA CD40	Wk10	CD4	1 cytokine	0.02165
R382	G2 MVA CD40	Wk10	CD4	2 cytokines	0.05283
R382	G2 MVA CD40	Wk10	CD4	3 cytokines	0.08917
R382	G2 MVA CD40	Wk10	CD8	1 cytokine	0.03186
R382	G2 MVA CD40	Wk10	CD8	2 cytokines	0.12541
R382	G2 MVA CD40	Wk10	CD8	3 cytokines	0.16623
R382	G2 MVA CD40	Wk26	CD4	1 cytokine	0.40823
R382	G2 MVA CD40	Wk26	CD4	2 cytokines	0.61383
R382	G2 MVA CD40	Wk26	CD4	3 cytokines	0.66772
R382	G2 MVA CD40	Wk26	CD8	1 cytokine	0.01403
R382	G2 MVA CD40	Wk26	CD8	2 cytokines	0.04856
R382	G2 MVA CD40	Wk26	CD8	3 cytokines	0.04856
R383	G4 CD40 MVA	Wk14	CD4	1 cytokine	0.095
R383	G4 CD40 MVA	Wk14	CD4	2 cytokines	0.16239
R383	G4 CD40 MVA	Wk14	CD4	3 cytokines	0.20061
R383	G4 CD40 MVA	Wk14	CD8	1 cytokine	0.0068
R383	G4 CD40 MVA	Wk14	CD8	2 cytokines	0.01225
R383	G4 CD40 MVA	Wk14	CD8	3 cytokines	0.0773
R383	G4 CD40 MVA	Wk24	CD4	1 cytokine	0.3473
R383	G4 CD40 MVA	Wk24	CD4	2 cytokines	0.52121
R383	G4 CD40 MVA	Wk24	CD4	3 cytokines	0.64513
R383	G4 CD40 MVA	Wk24	CD8	1 cytokine	0.03229
R383	G4 CD40 MVA	Wk24	CD8	2 cytokines	0.10328
R383	G4 CD40 MVA	Wk24	CD8	3 cytokines	0.28769
R384	G2 MVA CD40	Wk10	CD4	1 cytokine	0.00044
R384	G2 MVA CD40	Wk10	CD4	2 cytokines	0.01034
R384	G2 MVA CD40	Wk10	CD4	3 cytokines	0.0165
R384	G2 MVA CD40	Wk10	CD8	1 cytokine	0.00446
R384	G2 MVA CD40	Wk10	CD8	2 cytokines	0.03381
R384	G2 MVA CD40	Wk10	CD8	3 cytokines	0.30161
R384	G2 MVA CD40	Wk26	CD4	1 cytokine	0.36171
R384	G2 MVA CD40	Wk26	CD4	2 cytokines	0.64327
R384	G2 MVA CD40	Wk26	CD4	3 cytokines	0.74921
R384	G2 MVA CD40	Wk26	CD8	1 cytokine	0.01858
R384	G2 MVA CD40	Wk26	CD8	2 cytokines	0.13196
R384	G2 MVA CD40	Wk26	CD8	3 cytokines	0.4231
R385	G1 MVA DCIR	Wk10	CD4	3 cytokines	0.01066
R385	G1 MVA DCIR	Wk10	CD4	2 cytokines	0.01518
R385	G1 MVA DCIR	Wk10	CD4	1 cytokine	0.01686
R385	G1 MVA DCIR	Wk10	CD8	3 cytokines	0.00853
R385	G1 MVA DCIR	Wk10	CD8	2 cytokines	0.01939
R385	G1 MVA DCIR	Wk10	CD8	1 cytokine	0.09135
R385	G1 MVA DCIR	Wk26	CD4	3 cytokines	0.13356
R385	G1 MVA DCIR	Wk26	CD4	2 cytokines	0.20902
R385	G1 MVA DCIR	Wk26	CD4	1 cytokine	0.23624

R385	G1 MVA DCIR	Wk26	CD8	3 cytokines	0.00763
R385	G1 MVA DCIR	Wk26	CD8	2 cytokines	0.03759
R385	G1 MVA DCIR	Wk26	CD8	1 cytokine	0.11601
R386	G4 CD40 MVA	Wk14	CD4	1 cytokine	0.04131
R386	G4 CD40 MVA	Wk14	CD4	2 cytokines	0.09357
R386	G4 CD40 MVA	Wk14	CD4	3 cytokines	0.15287
R386	G4 CD40 MVA	Wk14	CD8	1 cytokine	0.01726
R386	G4 CD40 MVA	Wk14	CD8	2 cytokines	0.08592
R386	G4 CD40 MVA	Wk14	CD8	3 cytokines	0.132
R386	G4 CD40 MVA	Wk24	CD4	1 cytokine	0.04544
R386	G4 CD40 MVA	Wk24	CD4	2 cytokines	0.09527
R386	G4 CD40 MVA	Wk24	CD4	3 cytokines	0.1529
R386	G4 CD40 MVA	Wk24	CD8	1 cytokine	0.01585
R386	G4 CD40 MVA	Wk24	CD8	2 cytokines	0.06504
R386	G4 CD40 MVA	Wk24	CD8	3 cytokines	0.13216
R388	G3 DCIR MVA	Wk24	CD4	3 cytokines	0.0317
R388	G3 DCIR MVA	Wk24	CD4	2 cytokines	0.09314
R388	G3 DCIR MVA	Wk24	CD4	1 cytokine	0.12653
R388	G3 DCIR MVA	Wk24	CD8	3 cytokines	0.0023
R388	G3 DCIR MVA	Wk24	CD8	2 cytokines	0.02222
R388	G3 DCIR MVA	Wk24	CD8	1 cytokine	0.15366
R389	G4 CD40 MVA	Wk24	CD4	1 cytokine	0.20965
R389	G4 CD40 MVA	Wk24	CD4	2 cytokines	0.28967
R389	G4 CD40 MVA	Wk24	CD4	3 cytokines	0.33882
R389	G4 CD40 MVA	Wk24	CD8	1 cytokine	0.01633
R389	G4 CD40 MVA	Wk24	CD8	2 cytokines	0.05975
R389	G4 CD40 MVA	Wk24	CD8	3 cytokines	0.11429
R390	G4 CD40 MVA	Wk14	CD4	1 cytokine	0.00876
R390	G4 CD40 MVA	Wk14	CD4	2 cytokines	0.0166
R390	G4 CD40 MVA	Wk14	CD4	3 cytokines	0.02288
R390	G4 CD40 MVA	Wk14	CD8	1 cytokine	0.000
R390	G4 CD40 MVA	Wk14	CD8	2 cytokines	0.01722
R390	G4 CD40 MVA	Wk14	CD8	3 cytokines	0.06344
R390	G4 CD40 MVA	Wk24	CD4	1 cytokine	0.04056
R390	G4 CD40 MVA	Wk24	CD4	2 cytokines	0.06347
R390	G4 CD40 MVA	Wk24	CD4	3 cytokines	0.07834
R390	G4 CD40 MVA	Wk24	CD8	1 cytokine	0.00757
R390	G4 CD40 MVA	Wk24	CD8	2 cytokines	0.04337
R390	G4 CD40 MVA	Wk24	CD8	3 cytokines	0.13132
R391	G4 CD40 MVA	Wk14	CD4	1 cytokine	0.01963
R391	G4 CD40 MVA	Wk14	CD4	2 cytokines	0.05489
R391	G4 CD40 MVA	Wk14	CD4	3 cytokines	0.07945
R391	G4 CD40 MVA	Wk14	CD8	1 cytokine	0.00478
R391	G4 CD40 MVA	Wk14	CD8	2 cytokines	0.0229
R391	G4 CD40 MVA	Wk14	CD8	3 cytokines	0.07322
R391	G4 CD40 MVA	Wk24	CD4	1 cytokine	0.0377

R391	G4 CD40 MVA	Wk24	CD4	2 cytokines	0.06141
R391	G4 CD40 MVA	Wk24	CD4	3 cytokines	0.08772
R391	G4 CD40 MVA	Wk24	CD8	1 cytokine	0.00971
R391	G4 CD40 MVA	Wk24	CD8	2 cytokines	0.02435
R391	G4 CD40 MVA	Wk24	CD8	3 cytokines	0.05628
