# Supplementary material for: HIV-1 T cell epitopes targeted to Rhesus macaque CD40 and DCIR: A comparative study of prototype dendritic cell targeting therapeutic vaccine candidates
Source: PLoS One. 2018 Nov 30;13(11):e0207794. doi: 10.1371/journal.pone.0207794 (PMC6267996; doi:10.1371/journal.pone.0207794)
Supplement: S4 Table — This table is the data that relates to Fig 4 (G1 and G2) and Fig 5 (G3 and G4). Animal and peptides name, group, T cell types and sample time in weeks are identified. The % response values for either HIV-1 antigen-specific CD4+ or CD8+ T cells are the sum of 1 cytokine, 2 cytokines, and three cytokines as determined by the ICS analysis. (PDF) [file pone.0207794.s008.pdf]

**S4 Table. Analysis of HIV-1 epitope-specific CD4$^+$ and CD8$^+$ T cell responses elicited in MVA-primed NHPs by αDCIR.HIV5pep and αCD40.HIV5pep vaccines and in naïve NHPs by αDCIR.HIV5pep and αCD40.HIV5pep vaccines.** This table is the data that relates to Fig 4 (G1 and G2) and Fig 5 (G3 and G4). Animal and peptides name, group, T cell types and sample time in weeks are identified. The % response values for either HIV-1 antigen-specific CD4$^+$ or CD8$^+$ T cells are the sum of 1 cytokine, 2 cytokines, and three cytokines as determined by the ICS analysis.

| Animal | Peptides | Group | Sample time | T cell type | Cytokine No. | % Response |
|---|---|---|---|---|---|---|
| R368 | Gag p17 | G3 DCIR MVA | Wk14 | CD4 | 1 cytokine | 0.01061 |
| R368 | Gag p17 | G3 DCIR MVA | Wk14 | CD8 | 1 cytokine | 0.02712 |
| R368 | Gag p17 | G3 DCIR MVA | Wk24 | CD4 | 1 cytokine | 0.01458 |
| R368 | Gag p17 | G3 DCIR MVA | Wk24 | CD8 | 1 cytokine | 0.10983 |
| R368 | Gag p24 | G3 DCIR MVA | Wk14 | CD4 | 1 cytokine | 0.02553 |
| R368 | Gag p24 | G3 DCIR MVA | Wk14 | CD8 | 1 cytokine | 0.02968 |
| R368 | Gag p24 | G3 DCIR MVA | Wk24 | CD4 | 1 cytokine | 0.03344 |
| R368 | Gag p24 | G3 DCIR MVA | Wk24 | CD8 | 1 cytokine | 0.11399 |
| R368 | Nef | G3 DCIR MVA | Wk14 | CD4 | 1 cytokine | 0.05474 |
| R368 | Nef | G3 DCIR MVA | Wk14 | CD8 | 1 cytokine | 0.04571 |
| R368 | Nef | G3 DCIR MVA | Wk24 | CD4 | 1 cytokine | 0.06318 |
| R368 | Nef | G3 DCIR MVA | Wk24 | CD8 | 1 cytokine | 0.12163 |
| R368 | Pol | G3 DCIR MVA | Wk14 | CD4 | 1 cytokine | 0.04755 |
| R368 | Pol | G3 DCIR MVA | Wk14 | CD8 | 1 cytokine | 0.09774 |
| R368 | Pol | G3 DCIR MVA | Wk24 | CD4 | 1 cytokine | 0.03543 |
| R368 | Pol | G3 DCIR MVA | Wk24 | CD8 | 1 cytokine | 0.11083 |
| R369 | Gag p17 | G1 MVA DCIR | Wk10 | CD4 | 1 cytokine | 0.01128 |
| R369 | Gag p17 | G1 MVA DCIR | Wk10 | CD8 | 1 cytokine | 0.02779 |
| R369 | Gag p17 | G1 MVA DCIR | Wk26 | CD4 | 1 cytokine | 0.01637 |
| R369 | Gag p17 | G1 MVA DCIR | Wk26 | CD8 | 1 cytokine | 0.02819 |
| R369 | Gag p24 | G1 MVA DCIR | Wk10 | CD4 | 1 cytokine | 0.00629 |
| R369 | Gag p24 | G1 MVA DCIR | Wk10 | CD8 | 1 cytokine | 0.11651 |
| R369 | Gag p24 | G1 MVA DCIR | Wk26 | CD4 | 1 cytokine | 0.31576 |
| R369 | Gag p24 | G1 MVA DCIR | Wk26 | CD8 | 1 cytokine | 0.14028 |
| R369 | Nef | G1 MVA DCIR | Wk10 | CD4 | 1 cytokine | 0.00782 |
| R369 | Nef | G1 MVA DCIR | Wk10 | CD8 | 1 cytokine | 0.06174 |
| R369 | Nef | G1 MVA DCIR | Wk26 | CD4 | 1 cytokine | 0.30562 |
| R369 | Nef | G1 MVA DCIR | Wk26 | CD8 | 1 cytokine | 0.04434 |
| R369 | Pol | G1 MVA DCIR | Wk10 | CD4 | 1 cytokine | 0.00722 |
| R369 | Pol | G1 MVA DCIR | Wk10 | CD8 | 1 cytokine | 0.01738 |
| R369 | Pol | G1 MVA DCIR | Wk26 | CD4 | 1 cytokine | 0.01618 |
| R369 | Pol | G1 MVA DCIR | Wk26 | CD8 | 1 cytokine | 0.04843 |
| R370 | Gag p17 | G1 MVA DCIR | Wk10 | CD4 | 1 cytokine | 0.00272 |
| R370 | Gag p17 | G1 MVA DCIR | Wk10 | CD8 | 1 cytokine | 0.00336 |
| R370 | Gag p17 | G1 MVA DCIR | Wk26 | CD4 | 1 cytokine | 0.00167 |
| R370 | Gag p17 | G1 MVA DCIR | Wk26 | CD8 | 1 cytokine | 0.01247 |
| R370 | Gag p24 | G1 MVA DCIR | Wk10 | CD4 | 1 cytokine | 0.00454 |
| R370 | Gag p24 | G1 MVA DCIR | Wk10 | CD8 | 1 cytokine | 0.05634 |

| | | | | | | |
|---|---|---|---|---|---|---|
| R370 | Gag p24 | G1 MVA DCIR | Wk26 | CD4 | 1 cytokine | 0.00702 |
| R370 | Gag p24 | G1 MVA DCIR | Wk26 | CD8 | 1 cytokine | 0.11599 |
| R370 | Nef | G1 MVA DCIR | Wk10 | CD4 | 1 cytokine | 0.00664 |
| R370 | Nef | G1 MVA DCIR | Wk10 | CD8 | 1 cytokine | 0.00498 |
| R370 | Nef | G1 MVA DCIR | Wk26 | CD4 | 1 cytokine | 0.00811 |
| R370 | Nef | G1 MVA DCIR | Wk26 | CD8 | 1 cytokine | 0.1042 |
| R370 | Pol | G1 MVA DCIR | Wk10 | CD4 | 1 cytokine | 0.00215 |
| R370 | Pol | G1 MVA DCIR | Wk10 | CD8 | 1 cytokine | 0.00228 |
| R370 | Pol | G1 MVA DCIR | Wk26 | CD4 | 1 cytokine | 0.03505 |
| R370 | Pol | G1 MVA DCIR | Wk26 | CD8 | 1 cytokine | 0.02394 |
| R371 | Gag p17 | G1 MVA DCIR | Wk10 | CD4 | 1 cytokine | 0.00755 |
| R371 | Gag p17 | G1 MVA DCIR | Wk10 | CD8 | 1 cytokine | 0.04784 |
| R371 | Gag p17 | G1 MVA DCIR | Wk26 | CD4 | 1 cytokine | 0.00551 |
| R371 | Gag p17 | G1 MVA DCIR | Wk26 | CD8 | 1 cytokine | 0.03358 |
| R371 | Gag p24 | G1 MVA DCIR | Wk10 | CD4 | 1 cytokine | 0.00507 |
| R371 | Gag p24 | G1 MVA DCIR | Wk10 | CD8 | 1 cytokine | 0.16023 |
| R371 | Gag p24 | G1 MVA DCIR | Wk26 | CD4 | 1 cytokine | 0.03551 |
| R371 | Gag p24 | G1 MVA DCIR | Wk26 | CD8 | 1 cytokine | 0.07039 |
| R371 | Nef | G1 MVA DCIR | Wk10 | CD4 | 1 cytokine | 0.01046 |
| R371 | Nef | G1 MVA DCIR | Wk10 | CD8 | 1 cytokine | 0.00933 |
| R371 | Nef | G1 MVA DCIR | Wk26 | CD4 | 1 cytokine | 0.02488 |
| R371 | Nef | G1 MVA DCIR | Wk26 | CD8 | 1 cytokine | 0.02488 |
| R371 | Pol | G1 MVA DCIR | Wk10 | CD4 | 1 cytokine | 0.00948 |
| R371 | Pol | G1 MVA DCIR | Wk10 | CD8 | 1 cytokine | 0.00456 |
| R371 | Pol | G1 MVA DCIR | Wk26 | CD4 | 1 cytokine | 0.16495 |
| R371 | Pol | G1 MVA DCIR | Wk26 | CD8 | 1 cytokine | 0.01028 |
| R372 | Gag p17 | G4 CD40 MVA | Wk14 | CD4 | 1 cytokine | 0.00324 |
| R372 | Gag p17 | G4 CD40 MVA | Wk14 | CD8 | 1 cytokine | 0.0175 |
| R372 | Gag p17 | G4 CD40 MVA | Wk24 | CD4 | 1 cytokine | 0.02602 |
| R372 | Gag p17 | G4 CD40 MVA | Wk24 | CD8 | 1 cytokine | 0.09521 |
| R372 | Gag p24 | G4 CD40 MVA | Wk14 | CD4 | 1 cytokine | 0.0299 |
| R372 | Gag p24 | G4 CD40 MVA | Wk14 | CD8 | 1 cytokine | 0.04593 |
| R372 | Gag p24 | G4 CD40 MVA | Wk24 | CD4 | 1 cytokine | 0.06647 |
| R372 | Gag p24 | G4 CD40 MVA | Wk24 | CD8 | 1 cytokine | 0.03675 |
| R372 | Nef | G4 CD40 MVA | Wk14 | CD4 | 1 cytokine | 0.01852 |
| R372 | Nef | G4 CD40 MVA | Wk14 | CD8 | 1 cytokine | 0.02702 |
| R372 | Nef | G4 CD40 MVA | Wk24 | CD4 | 1 cytokine | 0.03794 |
| R372 | Nef | G4 CD40 MVA | Wk24 | CD8 | 1 cytokine | 0.00425 |
| R372 | Pol | G4 CD40 MVA | Wk14 | CD4 | 1 cytokine | 0.04998 |
| R372 | Pol | G4 CD40 MVA | Wk14 | CD8 | 1 cytokine | 0.04494 |
| R372 | Pol | G4 CD40 MVA | Wk24 | CD4 | 1 cytokine | 0.1555 |
| R372 | Pol | G4 CD40 MVA | Wk24 | CD8 | 1 cytokine | 0.00673 |
| R373 | Gag p17 | G1 MVA DCIR | Wk10 | CD4 | 1 cytokine | 0.00987 |
| R373 | Gag p17 | G1 MVA DCIR | Wk10 | CD8 | 1 cytokine | 0.02311 |
| R373 | Gag p17 | G1 MVA DCIR | Wk26 | CD4 | 1 cytokine | 0.00452 |
| R373 | Gag p17 | G1 MVA DCIR | Wk26 | CD8 | 1 cytokine | 0.01966 |
| R373 | Gag p24 | G1 MVA DCIR | Wk10 | CD4 | 1 cytokine | 0.00162 |
| R373 | Gag p24 | G1 MVA DCIR | Wk10 | CD8 | 1 cytokine | 0.01153 |
| R373 | Gag p24 | G1 MVA DCIR | Wk26 | CD4 | 1 cytokine | 0.051 |
| R373 | Gag p24 | G1 MVA DCIR | Wk26 | CD8 | 1 cytokine | 0.02342 |
| R373 | Nef | G1 MVA DCIR | Wk10 | CD4 | 1 cytokine | 0.00557 |
| R373 | Nef | G1 MVA DCIR | Wk10 | CD8 | 1 cytokine | 0.03232 |
| R373 | Nef | G1 MVA DCIR | Wk26 | CD4 | 1 cytokine | 0.07213 |
| R373 | Nef | G1 MVA DCIR | Wk26 | CD8 | 1 cytokine | 0.00708 |
| R373 | Pol | G1 MVA DCIR | Wk10 | CD4 | 1 cytokine | 0.00342 |
| R373 | Pol | G1 MVA DCIR | Wk10 | CD8 | 1 cytokine | 0.04333 |
| R373 | Pol | G1 MVA DCIR | Wk26 | CD4 | 1 cytokine | 0.00894 |
| R373 | Pol | G1 MVA DCIR | Wk26 | CD8 | 1 cytokine | 0.00833 |

| | | | | | | |
|---|---|---|---|---|---|---|
| R374 | Gag p17 | G1 MVA DCIR | Wk10 | CD4 | 1 cytokine | 0.01929 |
| R374 | Gag p17 | G1 MVA DCIR | Wk10 | CD8 | 1 cytokine | 0.01277 |
| R374 | Gag p17 | G1 MVA DCIR | Wk26 | CD4 | 1 cytokine | 0.0207 |
| R374 | Gag p17 | G1 MVA DCIR | Wk26 | CD8 | 1 cytokine | 0.00579 |
| R374 | Gag p24 | G1 MVA DCIR | Wk10 | CD4 | 1 cytokine | 0.00782 |
| R374 | Gag p24 | G1 MVA DCIR | Wk10 | CD8 | 1 cytokine | 0.02324 |
| R374 | Gag p24 | G1 MVA DCIR | Wk26 | CD4 | 1 cytokine | 0.29654 |
| R374 | Gag p24 | G1 MVA DCIR | Wk26 | CD8 | 1 cytokine | 0.03045 |
| R374 | Nef | G1 MVA DCIR | Wk10 | CD4 | 1 cytokine | 0.00364 |
| R374 | Nef | G1 MVA DCIR | Wk10 | CD8 | 1 cytokine | 0.02803 |
| R374 | Nef | G1 MVA DCIR | Wk26 | CD4 | 1 cytokine | 0.06906 |
| R374 | Nef | G1 MVA DCIR | Wk26 | CD8 | 1 cytokine | 0.02258 |
| R374 | Pol | G1 MVA DCIR | Wk10 | CD4 | 1 cytokine | 0.00259 |
| R374 | Pol | G1 MVA DCIR | Wk10 | CD8 | 1 cytokine | 0.05831 |
| R374 | Pol | G1 MVA DCIR | Wk26 | CD4 | 1 cytokine | 0.01133 |
| R374 | Pol | G1 MVA DCIR | Wk26 | CD8 | 1 cytokine | 0.02422 |
| R375 | Gag p17 | G3 DCIR MVA | Wk14 | CD4 | 1 cytokine | 0.01099 |
| R375 | Gag p17 | G3 DCIR MVA | Wk14 | CD8 | 1 cytokine | 0.02067 |
| R375 | Gag p24 | G3 DCIR MVA | Wk14 | CD4 | 1 cytokine | 0.00735 |
| R375 | Gag p24 | G3 DCIR MVA | Wk14 | CD8 | 1 cytokine | 0.03611 |
| R375 | Gag p24 | G3 DCIR MVA | Wk24 | CD4 | 1 cytokine | 0.00744 |
| R375 | Gag p24 | G3 DCIR MVA | Wk24 | CD8 | 1 cytokine | 0.00698 |
| R375 | Nef | G3 DCIR MVA | Wk14 | CD4 | 1 cytokine | 0.02779 |
| R375 | Nef | G3 DCIR MVA | Wk14 | CD8 | 1 cytokine | 0.01833 |
| R375 | Nef | G3 DCIR MVA | Wk24 | CD4 | 1 cytokine | 0.04134 |
| R375 | Nef | G3 DCIR MVA | Wk24 | CD8 | 1 cytokine | 0.02254 |
| R375 | Pol | G3 DCIR MVA | Wk14 | CD4 | 1 cytokine | 0.0648 |
| R375 | Pol | G3 DCIR MVA | Wk14 | CD8 | 1 cytokine | 0.11661 |
| R375 | Pol | G3 DCIR MVA | Wk24 | CD4 | 1 cytokine | 0.06391 |
| R375 | Pol | G3 DCIR MVA | Wk24 | CD8 | 1 cytokine | 0.01163 |
| R376 | Gag p17 | G2 MVA CD40 | Wk10 | CD4 | 1 cytokine | 0.00899 |
| R376 | Gag p17 | G2 MVA CD40 | Wk10 | CD8 | 1 cytokine | 0.00115 |
| R376 | Gag p17 | G2 MVA CD40 | Wk26 | CD4 | 1 cytokine | 0.00452 |
| R376 | Gag p17 | G2 MVA CD40 | Wk26 | CD8 | 1 cytokine | 0.03384 |
| R376 | Gag p24 | G2 MVA CD40 | Wk10 | CD4 | 1 cytokine | 0.0133 |
| R376 | Gag p24 | G2 MVA CD40 | Wk10 | CD8 | 1 cytokine | 0.15032 |
| R376 | Gag p24 | G2 MVA CD40 | Wk26 | CD4 | 1 cytokine | 0.02098 |
| R376 | Gag p24 | G2 MVA CD40 | Wk26 | CD8 | 1 cytokine | 0.03805 |
| R376 | Nef | G2 MVA CD40 | Wk10 | CD4 | 1 cytokine | 0.01058 |
| R376 | Nef | G2 MVA CD40 | Wk10 | CD8 | 1 cytokine | 0.00399 |
| R376 | Nef | G2 MVA CD40 | Wk26 | CD4 | 1 cytokine | 0.08813 |
| R376 | Nef | G2 MVA CD40 | Wk26 | CD8 | 1 cytokine | 0,0005 |
| R376 | Pol | G2 MVA CD40 | Wk10 | CD4 | 1 cytokine | 0.00078 |
| R376 | Pol | G2 MVA CD40 | Wk10 | CD8 | 1 cytokine | 0.02946 |
| R376 | Pol | G2 MVA CD40 | Wk26 | CD4 | 1 cytokine | 0.0014 |
| R376 | Pol | G2 MVA CD40 | Wk26 | CD8 | 1 cytokine | 0 |
| R377 | Gag p17 | G2 MVA CD40 | Wk10 | CD4 | 1 cytokine | 0.0191 |
| R377 | Gag p17 | G2 MVA CD40 | Wk10 | CD8 | 1 cytokine | 0.0169 |
| R377 | Gag p17 | G2 MVA CD40 | Wk26 | CD4 | 1 cytokine | 0.02897 |
| R377 | Gag p17 | G2 MVA CD40 | Wk26 | CD8 | 1 cytokine | 0.03516 |
| R377 | Gag p24 | G2 MVA CD40 | Wk10 | CD4 | 1 cytokine | 0.01946 |
| R377 | Gag p24 | G2 MVA CD40 | Wk10 | CD8 | 1 cytokine | 0.0748 |
| R377 | Gag p24 | G2 MVA CD40 | Wk26 | CD4 | 1 cytokine | 0.11326 |
| R377 | Gag p24 | G2 MVA CD40 | Wk26 | CD8 | 1 cytokine | 0.03427 |
| R377 | Nef | G2 MVA CD40 | Wk10 | CD4 | 1 cytokine | 0.01583 |
| R377 | Nef | G2 MVA CD40 | Wk10 | CD8 | 1 cytokine | 0.0690 |
| R377 | Nef | G2 MVA CD40 | Wk26 | CD4 | 1 cytokine | 0.08305 |
| R377 | Nef | G2 MVA CD40 | Wk26 | CD8 | 1 cytokine | 0.061 |

| | | | | | | |
|---|---|---|---|---|---|---|
| R377 | Pol | G2 MVA CD40 | Wk10 | CD4 | 1 cytokine | 0.03267 |
| R377 | Pol | G2 MVA CD40 | Wk10 | CD8 | 1 cytokine | 0.08404 |
| R377 | Pol | G2 MVA CD40 | Wk26 | CD4 | 1 cytokine | 0.90184 |
| R377 | Pol | G2 MVA CD40 | Wk26 | CD8 | 1 cytokine | 0.06599 |
| R378 | Gag p17 | G2 MVA CD40 | Wk10 | CD4 | 1 cytokine | 0.0008 |
| R378 | Gag p17 | G2 MVA CD40 | Wk10 | CD8 | 1 cytokine | 0,00008 |
| R378 | Gag p17 | G2 MVA CD40 | Wk26 | CD4 | 1 cytokine | 0.01696 |
| R378 | Gag p17 | G2 MVA CD40 | Wk26 | CD8 | 1 cytokine | 0.01436 |
| R378 | Gag p24 | G2 MVA CD40 | Wk10 | CD4 | 1 cytokine | 0.00637 |
| R378 | Gag p24 | G2 MVA CD40 | Wk10 | CD8 | 1 cytokine | 0.01844 |
| R378 | Gag p24 | G2 MVA CD40 | Wk26 | CD4 | 1 cytokine | 0.08406 |
| R378 | Gag p24 | G2 MVA CD40 | Wk26 | CD8 | 1 cytokine | 0.0162 |
| R378 | Nef | G2 MVA CD40 | Wk10 | CD4 | 1 cytokine | 0.0059 |
| R378 | Nef | G2 MVA CD40 | Wk10 | CD8 | 1 cytokine | 0.05844 |
| R378 | Nef | G2 MVA CD40 | Wk26 | CD4 | 1 cytokine | 0.05628 |
| R378 | Nef | G2 MVA CD40 | Wk26 | CD8 | 1 cytokine | 0.00581 |
| R378 | Pol | G2 MVA CD40 | Wk10 | CD4 | 1 cytokine | 0.0068 |
| R378 | Pol | G2 MVA CD40 | Wk10 | CD8 | 1 cytokine | 0.02926 |
| R378 | Pol | G2 MVA CD40 | Wk26 | CD4 | 1 cytokine | 0.24412 |
| R378 | Pol | G2 MVA CD40 | Wk26 | CD8 | 1 cytokine | 0.00247 |
| R379 | Gag p17 | G3 DCIR MVA | Wk14 | CD4 | 1 cytokine | 0.00564 |
| R379 | Gag p17 | G3 DCIR MVA | Wk14 | CD8 | 1 cytokine | 0.01923 |
| R379 | Gag p17 | G3 DCIR MVA | Wk24 | CD4 | 1 cytokine | 0.01753 |
| R379 | Gag p17 | G3 DCIR MVA | Wk24 | CD8 | 1 cytokine | 0.02815 |
| R379 | Gag p24 | G3 DCIR MVA | Wk14 | CD4 | 1 cytokine | 0.02753 |
| R379 | Gag p24 | G3 DCIR MVA | Wk14 | CD8 | 1 cytokine | 0.08315 |
| R379 | Gag p24 | G3 DCIR MVA | Wk24 | CD4 | 1 cytokine | 0.04588 |
| R379 | Gag p24 | G3 DCIR MVA | Wk24 | CD8 | 1 cytokine | 0.01309 |
| R379 | Nef | G3 DCIR MVA | Wk14 | CD4 | 1 cytokine | 0.01079 |
| R379 | Nef | G3 DCIR MVA | Wk14 | CD8 | 1 cytokine | 0.01385 |
| R379 | Nef | G3 DCIR MVA | Wk24 | CD4 | 1 cytokine | 0.02565 |
| R379 | Nef | G3 DCIR MVA | Wk24 | CD8 | 1 cytokine | 0.02319 |
| R379 | Pol | G3 DCIR MVA | Wk14 | CD4 | 1 cytokine | 0.16296 |
| R379 | Pol | G3 DCIR MVA | Wk14 | CD8 | 1 cytokine | 0.08565 |
| R379 | Pol | G3 DCIR MVA | Wk24 | CD4 | 1 cytokine | 0.27065 |
| R379 | Pol | G3 DCIR MVA | Wk24 | CD8 | 1 cytokine | 0.03868 |
| R380 | Gag p17 | G2 MVA CD40 | Wk10 | CD4 | 1 cytokine | 0.02833 |
| R380 | Gag p17 | G2 MVA CD40 | Wk10 | CD8 | 1 cytokine | 0.00596 |
| R380 | Gag p17 | G2 MVA CD40 | Wk26 | CD4 | 1 cytokine | 0.01108 |
| R380 | Gag p17 | G2 MVA CD40 | Wk26 | CD8 | 1 cytokine | 0.06883 |
| R380 | Gag p24 | G2 MVA CD40 | Wk10 | CD4 | 1 cytokine | 0.02607 |
| R380 | Gag p24 | G2 MVA CD40 | Wk10 | CD8 | 1 cytokine | 0.02696 |
| R380 | Gag p24 | G2 MVA CD40 | Wk26 | CD4 | 1 cytokine | 0.25522 |
| R380 | Gag p24 | G2 MVA CD40 | Wk26 | CD8 | 1 cytokine | 0.04976 |
| R380 | Nef | G2 MVA CD40 | Wk10 | CD4 | 1 cytokine | 0.02101 |
| R380 | Nef | G2 MVA CD40 | Wk10 | CD8 | 1 cytokine | 0.01669 |
| R380 | Nef | G2 MVA CD40 | Wk26 | CD4 | 1 cytokine | 0.13099 |
| R380 | Nef | G2 MVA CD40 | Wk26 | CD8 | 1 cytokine | 0.02802 |
| R380 | Pol | G2 MVA CD40 | Wk10 | CD4 | 1 cytokine | 0.02583 |
| R380 | Pol | G2 MVA CD40 | Wk10 | CD8 | 1 cytokine | 0.02992 |
| R380 | Pol | G2 MVA CD40 | Wk26 | CD4 | 1 cytokine | 0.03331 |
| R380 | Pol | G2 MVA CD40 | Wk26 | CD8 | 1 cytokine | 0.04576 |
| R381 | Gag p17 | G3 DCIR MVA | Wk14 | CD4 | 1 cytokine | 0.00362 |
| R381 | Gag p17 | G3 DCIR MVA | Wk14 | CD8 | 1 cytokine | 0.02239 |
| R381 | Gag p17 | G3 DCIR MVA | Wk24 | CD4 | 1 cytokine | 0.00607 |
| R381 | Gag p17 | G3 DCIR MVA | Wk24 | CD8 | 1 cytokine | 0.04178 |
| R381 | Gag p24 | G3 DCIR MVA | Wk14 | CD4 | 1 cytokine | 0.00637 |
| R381 | Gag p24 | G3 DCIR MVA | Wk14 | CD8 | 1 cytokine | 0.01716 |

| | | | | | | |
|---|---|---|---|---|---|---|
| R381 | Gag p24 | G3 DCIR MVA | Wk24 | CD4 | 1 cytokine | 0.02114 |
| R381 | Gag p24 | G3 DCIR MVA | Wk24 | CD8 | 1 cytokine | 0.04258 |
| R381 | Nef | G3 DCIR MVA | Wk14 | CD4 | 1 cytokine | 0.0145 |
| R381 | Nef | G3 DCIR MVA | Wk14 | CD8 | 1 cytokine | 0.04893 |
| R381 | Pol | G3 DCIR MVA | Wk14 | CD4 | 1 cytokine | 0.18676 |
| R381 | Pol | G3 DCIR MVA | Wk14 | CD8 | 1 cytokine | 0.01098 |
| R381 | Pol | G3 DCIR MVA | Wk24 | CD4 | 1 cytokine | 0.10357 |
| R381 | Pol | G3 DCIR MVA | Wk24 | CD8 | 1 cytokine | 0.04223 |
| R382 | Gag p17 | G2 MVA CD40 | Wk10 | CD4 | 1 cytokine | 0.01657 |
| R382 | Gag p17 | G2 MVA CD40 | Wk10 | CD8 | 1 cytokine | 0.00849 |
| R382 | Gag p17 | G2 MVA CD40 | Wk26 | CD4 | 1 cytokine | 0.01191 |
| R382 | Gag p17 | G2 MVA CD40 | Wk26 | CD8 | 1 cytokine | 0.00074 |
| R382 | Gag p24 | G2 MVA CD40 | Wk10 | CD4 | 1 cytokine | 0.01846 |
| R382 | Gag p24 | G2 MVA CD40 | Wk10 | CD8 | 1 cytokine | 0.02239 |
| R382 | Gag p24 | G2 MVA CD40 | Wk26 | CD4 | 1 cytokine | 0.16948 |
| R382 | Gag p24 | G2 MVA CD40 | Wk26 | CD8 | 1 cytokine | 0.00747 |
| R382 | Nef | G2 MVA CD40 | Wk10 | CD4 | 1 cytokine | 0.02529 |
| R382 | Nef | G2 MVA CD40 | Wk10 | CD8 | 1 cytokine | 0.06221 |
| R382 | Nef | G2 MVA CD40 | Wk26 | CD4 | 1 cytokine | 0.19382 |
| R382 | Nef | G2 MVA CD40 | Wk26 | CD8 | 1 cytokine | 0.01132 |
| R382 | Pol | G2 MVA CD40 | Wk10 | CD4 | 1 cytokine | 0.02885 |
| R382 | Pol | G2 MVA CD40 | Wk10 | CD8 | 1 cytokine | 0.07314 |
| R382 | Pol | G2 MVA CD40 | Wk26 | CD4 | 1 cytokine | 0.29251 |
| R382 | Pol | G2 MVA CD40 | Wk26 | CD8 | 1 cytokine | 0.02903 |
| R383 | Gag p17 | G4 CD40 MVA | Wk14 | CD4 | 1 cytokine | 0.01162 |
| R383 | Gag p17 | G4 CD40 MVA | Wk14 | CD8 | 1 cytokine | 0.00468 |
| R383 | Gag p17 | G4 CD40 MVA | Wk24 | CD4 | 1 cytokine | 0.02613 |
| R383 | Gag p17 | G4 CD40 MVA | Wk24 | CD8 | 1 cytokine | 0.02833 |
| R383 | Gag p24 | G4 CD40 MVA | Wk14 | CD4 | 1 cytokine | 0.03786 |
| R383 | Gag p24 | G4 CD40 MVA | Wk14 | CD8 | 1 cytokine | 0.066 |
| R383 | Gag p24 | G4 CD40 MVA | Wk24 | CD4 | 1 cytokine | 0.10746 |
| R383 | Gag p24 | G4 CD40 MVA | Wk24 | CD8 | 1 cytokine | 0.15899 |
| R383 | Nef | G4 CD40 MVA | Wk14 | CD4 | 1 cytokine | 0.06544 |
| R383 | Nef | G4 CD40 MVA | Wk14 | CD8 | 1 cytokine | 0.00183 |
| R383 | Nef | G4 CD40 MVA | Wk24 | CD4 | 1 cytokine | 0.16124 |
| R383 | Nef | G4 CD40 MVA | Wk24 | CD8 | 1 cytokine | 0.05627 |
| R383 | Pol | G4 CD40 MVA | Wk14 | CD4 | 1 cytokine | 0.08569 |
| R383 | Pol | G4 CD40 MVA | Wk14 | CD8 | 1 cytokine | 0.0047 |
| R383 | Pol | G4 CD40 MVA | Wk24 | CD4 | 1 cytokine | 0.3503 |
| R383 | Pol | G4 CD40 MVA | Wk24 | CD8 | 1 cytokine | 0.0441 |
| R384 | Gag p17 | G2 MVA CD40 | Wk26 | CD4 | 1 cytokine | 0.02572 |
| R384 | Gag p17 | G2 MVA CD40 | Wk26 | CD8 | 1 cytokine | 0.06902 |
| R384 | Gag p24 | G2 MVA CD40 | Wk10 | CD4 | 1 cytokine | 0.00508 |
| R384 | Gag p24 | G2 MVA CD40 | Wk10 | CD8 | 1 cytokine | 0.12067 |
| R384 | Gag p24 | G2 MVA CD40 | Wk26 | CD4 | 1 cytokine | 0.30606 |
| R384 | Gag p24 | G2 MVA CD40 | Wk26 | CD8 | 1 cytokine | 0.15596 |
| R384 | Nef | G2 MVA CD40 | Wk10 | CD4 | 1 cytokine | 0.0036 |
| R384 | Nef | G2 MVA CD40 | Wk10 | CD8 | 1 cytokine | 0.0829 |
| R384 | Nef | G2 MVA CD40 | Wk26 | CD4 | 1 cytokine | 0.26326 |
| R384 | Nef | G2 MVA CD40 | Wk26 | CD8 | 1 cytokine | 0.13867 |
| R384 | Pol | G2 MVA CD40 | Wk10 | CD4 | 1 cytokine | 0.00782 |
| R384 | Pol | G2 MVA CD40 | Wk10 | CD8 | 1 cytokine | 0.09804 |
| R384 | Pol | G2 MVA CD40 | Wk26 | CD4 | 1 cytokine | 0.15417 |
| R384 | Pol | G2 MVA CD40 | Wk26 | CD8 | 1 cytokine | 0.05945 |
| R385 | Gag p17 | G1 MVA DCIR | Wk10 | CD4 | 1 cytokine | 0.00549 |
| R385 | Gag p17 | G1 MVA DCIR | Wk10 | CD8 | 1 cytokine | 0.01812 |
| R385 | Gag p17 | G1 MVA DCIR | Wk26 | CD4 | 1 cytokine | 0.02415 |
| R385 | Gag p17 | G1 MVA DCIR | Wk26 | CD8 | 1 cytokine | 0.01153 |

| | | | | | | |
|---|---|---|---|---|---|---|
| R385 | Gag p24 | G1 MVA DCIR | Wk10 | CD4 | 1 cytokine | 0.00058 |
| R385 | Gag p24 | G1 MVA DCIR | Wk10 | CD8 | 1 cytokine | 0.01703 |
| R385 | Gag p24 | G1 MVA DCIR | Wk26 | CD4 | 1 cytokine | 0.05559 |
| R385 | Gag p24 | G1 MVA DCIR | Wk26 | CD8 | 1 cytokine | 0.0376 |
| R385 | Nef | G1 MVA DCIR | Wk10 | CD4 | 1 cytokine | 0.00661 |
| R385 | Nef | G1 MVA DCIR | Wk10 | CD8 | 1 cytokine | 0.0546 |
| R385 | Nef | G1 MVA DCIR | Wk26 | CD4 | 1 cytokine | 0.04188 |
| R385 | Nef | G1 MVA DCIR | Wk26 | CD8 | 1 cytokine | 0.02521 |
| R385 | Pol | G1 MVA DCIR | Wk10 | CD4 | 1 cytokine | 0.00418 |
| R385 | Pol | G1 MVA DCIR | Wk10 | CD8 | 1 cytokine | 0.0016 |
| R385 | Pol | G1 MVA DCIR | Wk26 | CD4 | 1 cytokine | 0.11462 |
| R385 | Pol | G1 MVA DCIR | Wk26 | CD8 | 1 cytokine | 0.04167 |
| R386 | Gag p17 | G4 CD40 MVA | Wk14 | CD4 | 1 cytokine | 0.02115 |
| R386 | Gag p17 | G4 CD40 MVA | Wk14 | CD8 | 1 cytokine | 0.10675 |
| R386 | Gag p17 | G4 CD40 MVA | Wk24 | CD4 | 1 cytokine | 0.01736 |
| R386 | Gag p17 | G4 CD40 MVA | Wk24 | CD8 | 1 cytokine | 0.09791 |
| R386 | Gag p24 | G4 CD40 MVA | Wk14 | CD4 | 1 cytokine | 0.04452 |
| R386 | Gag p24 | G4 CD40 MVA | Wk14 | CD8 | 1 cytokine | 0.01701 |
| R386 | Gag p24 | G4 CD40 MVA | Wk24 | CD4 | 1 cytokine | 0.04692 |
| R386 | Gag p24 | G4 CD40 MVA | Wk24 | CD8 | 1 cytokine | 0.00841 |
| R386 | Nef | G4 CD40 MVA | Wk14 | CD4 | 1 cytokine | 0.01865 |
| R386 | Nef | G4 CD40 MVA | Wk14 | CD8 | 1 cytokine | 0.0069 |
| R386 | Nef | G4 CD40 MVA | Wk24 | CD4 | 1 cytokine | 0.03145 |
| R386 | Nef | G4 CD40 MVA | Wk24 | CD8 | 1 cytokine | 0.02513 |
| R386 | Pol | G4 CD40 MVA | Wk14 | CD4 | 1 cytokine | 0.06855 |
| R386 | Pol | G4 CD40 MVA | Wk14 | CD8 | 1 cytokine | 0.00134 |
| R386 | Pol | G4 CD40 MVA | Wk24 | CD4 | 1 cytokine | 0.05717 |
| R386 | Pol | G4 CD40 MVA | Wk24 | CD8 | 1 cytokine | 0.0007 |
| R388 | Gag p17 | G3 DCIR MVA | Wk24 | CD4 | 1 cytokine | 0.04734 |
| R388 | Gag p17 | G3 DCIR MVA | Wk24 | CD8 | 1 cytokine | 0.08995 |
| R388 | Gag p24 | G3 DCIR MVA | Wk24 | CD4 | 1 cytokine | 0.07919 |
| R388 | Gag p24 | G3 DCIR MVA | Wk24 | CD8 | 1 cytokine | 0.06371 |
| R389 | Gag p17 | G4 CD40 MVA | Wk24 | CD4 | 1 cytokine | 0.01676 |
| R389 | Gag p17 | G4 CD40 MVA | Wk24 | CD8 | 1 cytokine | 0.04 |
| R389 | Gag p24 | G4 CD40 MVA | Wk24 | CD4 | 1 cytokine | 0.06398 |
| R389 | Gag p24 | G4 CD40 MVA | Wk24 | CD8 | 1 cytokine | 0.0184 |
| R389 | Pol | G4 CD40 MVA | Wk24 | CD4 | 1 cytokine | 0.25808 |
| R389 | Pol | G4 CD40 MVA | Wk24 | CD8 | 1 cytokine | 0.05589 |
| R390 | Gag p17 | G4 CD40 MVA | Wk14 | CD4 | 1 cytokine | 0.00111 |
| R390 | Gag p17 | G4 CD40 MVA | Wk14 | CD8 | 1 cytokine | 0.01574 |
| R390 | Gag p17 | G4 CD40 MVA | Wk24 | CD4 | 1 cytokine | 0.00592 |
| R390 | Gag p17 | G4 CD40 MVA | Wk24 | CD8 | 1 cytokine | 0.01056 |
| R390 | Gag p24 | G4 CD40 MVA | Wk14 | CD4 | 1 cytokine | 0.00452 |
| R390 | Gag p24 | G4 CD40 MVA | Wk14 | CD8 | 1 cytokine | 0.00577 |
| R390 | Gag p24 | G4 CD40 MVA | Wk24 | CD4 | 1 cytokine | 0.02044 |
| R390 | Gag p24 | G4 CD40 MVA | Wk24 | CD8 | 1 cytokine | 0.0308 |
| R390 | Nef | G4 CD40 MVA | Wk14 | CD4 | 1 cytokine | 0.00651 |
| R390 | Nef | G4 CD40 MVA | Wk14 | CD8 | 1 cytokine | 0.00359 |
| R390 | Nef | G4 CD40 MVA | Wk24 | CD4 | 1 cytokine | 0.01316 |
| R390 | Nef | G4 CD40 MVA | Wk24 | CD8 | 1 cytokine | 0.01517 |
| R390 | Pol | G4 CD40 MVA | Wk14 | CD4 | 1 cytokine | 0.01074 |
| R390 | Pol | G4 CD40 MVA | Wk14 | CD8 | 1 cytokine | 0.03834 |
| R390 | Pol | G4 CD40 MVA | Wk24 | CD4 | 1 cytokine | 0.03882 |
| R390 | Pol | G4 CD40 MVA | Wk24 | CD8 | 1 cytokine | 0.07479 |
| R391 | Gag p17 | G4 CD40 MVA | Wk14 | CD4 | 1 cytokine | 0.01549 |
| R391 | Gag p17 | G4 CD40 MVA | Wk14 | CD8 | 1 cytokine | 0.05389 |
| R391 | Gag p17 | G4 CD40 MVA | Wk24 | CD4 | 1 cytokine | 0.02039 |
| R391 | Gag p17 | G4 CD40 MVA | Wk24 | CD8 | 1 cytokine | 0.00888 |

| | | | | | | |
|---|---|---|---|---|---|---|
| R391 | Gag p24 | G4 CD40 MVA | Wk14 | CD4 | 1 cytokine | 0.06396 |
| R391 | Gag p24 | G4 CD40 MVA | Wk14 | CD8 | 1 cytokine | 0.01933 |
| R391 | Gag p24 | G4 CD40 MVA | Wk24 | CD4 | 1 cytokine | 0.06733 |
| R391 | Gag p24 | G4 CD40 MVA | Wk24 | CD8 | 1 cytokine | 0.0474 |
| R368 | Gag p17 | G3 DCIR MVA | Wk14 | CD4 | 2 cytokines | 0.00102 |
| R368 | Gag p17 | G3 DCIR MVA | Wk14 | CD8 | 2 cytokines | 0.014 |
| R368 | Gag p17 | G3 DCIR MVA | Wk24 | CD4 | 2 cytokines | 0.00537 |
| R368 | Gag p17 | G3 DCIR MVA | Wk24 | CD8 | 2 cytokines | 0.01681 |
| R368 | Gag p24 | G3 DCIR MVA | Wk14 | CD4 | 2 cytokines | 0.00574 |
| R368 | Gag p24 | G3 DCIR MVA | Wk14 | CD8 | 2 cytokines | 0.01384 |
| R368 | Gag p24 | G3 DCIR MVA | Wk24 | CD4 | 2 cytokines | 0.01463 |
| R368 | Gag p24 | G3 DCIR MVA | Wk24 | CD8 | 2 cytokines | 0.02195 |
| R368 | Nef | G3 DCIR MVA | Wk14 | CD4 | 2 cytokines | 0.03182 |
| R368 | Nef | G3 DCIR MVA | Wk14 | CD8 | 2 cytokines | 0.01032 |
| R368 | Nef | G3 DCIR MVA | Wk24 | CD4 | 2 cytokines | 0.04744 |
| R368 | Nef | G3 DCIR MVA | Wk24 | CD8 | 2 cytokines | 0.02738 |
| R368 | Pol | G3 DCIR MVA | Wk14 | CD4 | 2 cytokines | 0.02824 |
| R368 | Pol | G3 DCIR MVA | Wk14 | CD8 | 2 cytokines | 0.01536 |
| R368 | Pol | G3 DCIR MVA | Wk24 | CD4 | 2 cytokines | 0.02484 |
| R368 | Pol | G3 DCIR MVA | Wk24 | CD8 | 2 cytokines | 0.02703 |
| R369 | Gag p17 | G1 MVA DCIR | Wk10 | CD4 | 2 cytokines | 0.00767 |
| R369 | Gag p17 | G1 MVA DCIR | Wk10 | CD8 | 2 cytokines | 0.00398 |
| R369 | Gag p17 | G1 MVA DCIR | Wk26 | CD4 | 2 cytokines | 0.01188 |
| R369 | Gag p17 | G1 MVA DCIR | Wk26 | CD8 | 2 cytokines | 0.00649 |
| R369 | Gag p24 | G1 MVA DCIR | Wk10 | CD4 | 2 cytokines | 0.0036 |
| R369 | Gag p24 | G1 MVA DCIR | Wk10 | CD8 | 2 cytokines | 0.09657 |
| R369 | Gag p24 | G1 MVA DCIR | Wk26 | CD4 | 2 cytokines | 0.29446 |
| R369 | Gag p24 | G1 MVA DCIR | Wk26 | CD8 | 2 cytokines | 0.11466 |
| R369 | Nef | G1 MVA DCIR | Wk10 | CD4 | 2 cytokines | 0.00507 |
| R369 | Nef | G1 MVA DCIR | Wk10 | CD8 | 2 cytokines | 0.02631 |
| R369 | Nef | G1 MVA DCIR | Wk26 | CD4 | 2 cytokines | 0.2782 |
| R369 | Nef | G1 MVA DCIR | Wk26 | CD8 | 2 cytokines | 0.03534 |
| R369 | Pol | G1 MVA DCIR | Wk10 | CD4 | 2 cytokines | 0.00357 |
| R369 | Pol | G1 MVA DCIR | Wk10 | CD8 | 2 cytokines | 0.01209 |
| R369 | Pol | G1 MVA DCIR | Wk26 | CD4 | 2 cytokines | 0.00633 |
| R369 | Pol | G1 MVA DCIR | Wk26 | CD8 | 2 cytokines | 0.02243 |
| R370 | Gag p17 | G1 MVA DCIR | Wk10 | CD4 | 2 cytokines | 0.00151 |
| R370 | Gag p17 | G1 MVA DCIR | Wk10 | CD8 | 2 cytokines | 0,0007 |
| R370 | Gag p17 | G1 MVA DCIR | Wk26 | CD4 | 2 cytokines | 0.00167 |
| R370 | Gag p17 | G1 MVA DCIR | Wk26 | CD8 | 2 cytokines | 0.00431 |
| R370 | Gag p24 | G1 MVA DCIR | Wk10 | CD4 | 2 cytokines | 0.00249 |
| R370 | Gag p24 | G1 MVA DCIR | Wk10 | CD8 | 2 cytokines | 0.00283 |
| R370 | Gag p24 | G1 MVA DCIR | Wk26 | CD4 | 2 cytokines | 0.00246 |
| R370 | Gag p24 | G1 MVA DCIR | Wk26 | CD8 | 2 cytokines | 0.01207 |
| R370 | Nef | G1 MVA DCIR | Wk10 | CD4 | 2 cytokines | 0.00294 |
| R370 | Nef | G1 MVA DCIR | Wk10 | CD8 | 2 cytokines | 0.00011 |
| R370 | Nef | G1 MVA DCIR | Wk26 | CD4 | 2 cytokines | 0.00579 |
| R370 | Nef | G1 MVA DCIR | Wk26 | CD8 | 2 cytokines | 0.01595 |
| R370 | Pol | G1 MVA DCIR | Wk10 | CD4 | 2 cytokines | 0.00081 |
| R370 | Pol | G1 MVA DCIR | Wk10 | CD8 | 2 cytokines | 9.9e-05 |
| R370 | Pol | G1 MVA DCIR | Wk26 | CD4 | 2 cytokines | 0.0252 |
| R370 | Pol | G1 MVA DCIR | Wk26 | CD8 | 2 cytokines | 0.00443 |
| R371 | Gag p17 | G1 MVA DCIR | Wk10 | CD4 | 2 cytokines | 0.00529 |
| R371 | Gag p17 | G1 MVA DCIR | Wk10 | CD8 | 2 cytokines | 0.00191 |
| R371 | Gag p17 | G1 MVA DCIR | Wk26 | CD4 | 2 cytokines | 0.00444 |
| R371 | Gag p17 | G1 MVA DCIR | Wk26 | CD8 | 2 cytokines | 0.00373 |
| R371 | Gag p24 | G1 MVA DCIR | Wk10 | CD4 | 2 cytokines | 0.00483 |
| R371 | Gag p24 | G1 MVA DCIR | Wk10 | CD8 | 2 cytokines | 0.13816 |

| | | | | | | |
|---|---|---|---|---|---|---|
| R371 | Gag p24 | G1 MVA DCIR | Wk26 | CD4 | 2 cytokines | 0.03082 |
| R371 | Gag p24 | G1 MVA DCIR | Wk26 | CD8 | 2 cytokines | 0.05214 |
| R371 | Nef | G1 MVA DCIR | Wk10 | CD4 | 2 cytokines | 0.00717 |
| R371 | Nef | G1 MVA DCIR | Wk10 | CD8 | 2 cytokines | 0.00604 |
| R371 | Nef | G1 MVA DCIR | Wk26 | CD4 | 2 cytokines | 0.02215 |
| R371 | Nef | G1 MVA DCIR | Wk26 | CD8 | 2 cytokines | 0.00997 |
| R371 | Pol | G1 MVA DCIR | Wk10 | CD4 | 2 cytokines | 0.00779 |
| R371 | Pol | G1 MVA DCIR | Wk10 | CD8 | 2 cytokines | 0.00456 |
| R371 | Pol | G1 MVA DCIR | Wk26 | CD4 | 2 cytokines | 0.14115 |
| R371 | Pol | G1 MVA DCIR | Wk26 | CD8 | 2 cytokines | 0.00889 |
| R372 | Gag p17 | G4 CD40 MVA | Wk14 | CD4 | 2 cytokines | 0.00162 |
| R372 | Gag p17 | G4 CD40 MVA | Wk14 | CD8 | 2 cytokines | 0.00416 |
| R372 | Gag p17 | G4 CD40 MVA | Wk24 | CD4 | 2 cytokines | 0.02082 |
| R372 | Gag p17 | G4 CD40 MVA | Wk24 | CD8 | 2 cytokines | 0.0682 |
| R372 | Gag p24 | G4 CD40 MVA | Wk14 | CD4 | 2 cytokines | 0.01839 |
| R372 | Gag p24 | G4 CD40 MVA | Wk14 | CD8 | 2 cytokines | 0.02486 |
| R372 | Gag p24 | G4 CD40 MVA | Wk24 | CD4 | 2 cytokines | 0.05688 |
| R372 | Gag p24 | G4 CD40 MVA | Wk24 | CD8 | 2 cytokines | 0.03091 |
| R372 | Nef | G4 CD40 MVA | Wk14 | CD4 | 2 cytokines | 0.01158 |
| R372 | Nef | G4 CD40 MVA | Wk14 | CD8 | 2 cytokines | 0.01272 |
| R372 | Nef | G4 CD40 MVA | Wk24 | CD4 | 2 cytokines | 0.03208 |
| R372 | Nef | G4 CD40 MVA | Wk24 | CD8 | 2 cytokines | 0.00425 |
| R372 | Pol | G4 CD40 MVA | Wk14 | CD4 | 2 cytokines | 0.03837 |
| R372 | Pol | G4 CD40 MVA | Wk14 | CD8 | 2 cytokines | 0.01921 |
| R372 | Pol | G4 CD40 MVA | Wk24 | CD4 | 2 cytokines | 0.13941 |
| R372 | Pol | G4 CD40 MVA | Wk24 | CD8 | 2 cytokines | 0.00673 |
| R373 | Gag p17 | G1 MVA DCIR | Wk10 | CD4 | 2 cytokines | 0.00532 |
| R373 | Gag p17 | G1 MVA DCIR | Wk10 | CD8 | 2 cytokines | 0.00337 |
| R373 | Gag p17 | G1 MVA DCIR | Wk26 | CD4 | 2 cytokines | 0.0023 |
| R373 | Gag p17 | G1 MVA DCIR | Wk26 | CD8 | 2 cytokines | 0.00336 |
| R373 | Gag p24 | G1 MVA DCIR | Wk10 | CD4 | 2 cytokines | 0.00082 |
| R373 | Gag p24 | G1 MVA DCIR | Wk10 | CD8 | 2 cytokines | 0.01004 |
| R373 | Gag p24 | G1 MVA DCIR | Wk26 | CD4 | 2 cytokines | 0.04 |
| R373 | Gag p24 | G1 MVA DCIR | Wk26 | CD8 | 2 cytokines | 0.00188 |
| R373 | Nef | G1 MVA DCIR | Wk10 | CD4 | 2 cytokines | 0.00218 |
| R373 | Nef | G1 MVA DCIR | Wk10 | CD8 | 2 cytokines | 0.0052 |
| R373 | Nef | G1 MVA DCIR | Wk26 | CD4 | 2 cytokines | 0.06125 |
| R373 | Nef | G1 MVA DCIR | Wk26 | CD8 | 2 cytokines | 0.00708 |
| R373 | Pol | G1 MVA DCIR | Wk10 | CD4 | 2 cytokines | 0.00273 |
| R373 | Pol | G1 MVA DCIR | Wk10 | CD8 | 2 cytokines | 0.00577 |
| R373 | Pol | G1 MVA DCIR | Wk26 | CD4 | 2 cytokines | 0.00775 |
| R373 | Pol | G1 MVA DCIR | Wk26 | CD8 | 2 cytokines | 0.00342 |
| R374 | Gag p17 | G1 MVA DCIR | Wk10 | CD4 | 2 cytokines | 0.0165 |
| R374 | Gag p17 | G1 MVA DCIR | Wk10 | CD8 | 2 cytokines | 0.00047 |
| R374 | Gag p17 | G1 MVA DCIR | Wk26 | CD4 | 2 cytokines | 0.01987 |
| R374 | Gag p17 | G1 MVA DCIR | Wk26 | CD8 | 2 cytokines | 0 |
| R374 | Gag p24 | G1 MVA DCIR | Wk10 | CD4 | 2 cytokines | 0.00602 |
| R374 | Gag p24 | G1 MVA DCIR | Wk10 | CD8 | 2 cytokines | 0.02023 |
| R374 | Gag p24 | G1 MVA DCIR | Wk26 | CD4 | 2 cytokines | 0.27054 |
| R374 | Gag p24 | G1 MVA DCIR | Wk26 | CD8 | 2 cytokines | 0.01128 |
| R374 | Nef | G1 MVA DCIR | Wk10 | CD4 | 2 cytokines | 0.00323 |
| R374 | Nef | G1 MVA DCIR | Wk10 | CD8 | 2 cytokines | 0.01935 |
| R374 | Nef | G1 MVA DCIR | Wk26 | CD4 | 2 cytokines | 0.06281 |
| R374 | Nef | G1 MVA DCIR | Wk26 | CD8 | 2 cytokines | 0.0033 |
| R374 | Pol | G1 MVA DCIR | Wk10 | CD4 | 2 cytokines | 0.0013 |
| R374 | Pol | G1 MVA DCIR | Wk10 | CD8 | 2 cytokines | 0.01072 |
| R374 | Pol | G1 MVA DCIR | Wk26 | CD4 | 2 cytokines | 0.01058 |
| R374 | Pol | G1 MVA DCIR | Wk26 | CD8 | 2 cytokines | 0.00191 |

| | | | | | | |
|---|---|---|---|---|---|---|
| R375 | Gag p17 | G3 DCIR MVA | Wk14 | CD4 | 2 cytokines | 0.00116 |
| R375 | Gag p17 | G3 DCIR MVA | Wk14 | CD8 | 2 cytokines | 0.00539 |
| R375 | Gag p24 | G3 DCIR MVA | Wk14 | CD4 | 2 cytokines | 0.00277 |
| R375 | Gag p24 | G3 DCIR MVA | Wk14 | CD8 | 2 cytokines | 0.00029 |
| R375 | Gag p24 | G3 DCIR MVA | Wk24 | CD4 | 2 cytokines | 0.00432 |
| R375 | Gag p24 | G3 DCIR MVA | Wk24 | CD8 | 2 cytokines | 0.00047 |
| R375 | Nef | G3 DCIR MVA | Wk14 | CD4 | 2 cytokines | 0.02246 |
| R375 | Nef | G3 DCIR MVA | Wk14 | CD8 | 2 cytokines | 0.00378 |
| R375 | Nef | G3 DCIR MVA | Wk24 | CD4 | 2 cytokines | 0.03146 |
| R375 | Nef | G3 DCIR MVA | Wk24 | CD8 | 2 cytokines | 0.00199 |
| R375 | Pol | G3 DCIR MVA | Wk14 | CD4 | 2 cytokines | 0.02457 |
| R375 | Pol | G3 DCIR MVA | Wk14 | CD8 | 2 cytokines | 0 |
| R375 | Pol | G3 DCIR MVA | Wk24 | CD4 | 2 cytokines | 0.05569 |
| R375 | Pol | G3 DCIR MVA | Wk24 | CD8 | 2 cytokines | 0,0005 |
| R376 | Gag p17 | G2 MVA CD40 | Wk10 | CD4 | 2 cytokines | 0.00377 |
| R376 | Gag p17 | G2 MVA CD40 | Wk10 | CD8 | 2 cytokines | 0.00115 |
| R376 | Gag p17 | G2 MVA CD40 | Wk26 | CD4 | 2 cytokines | 0.00318 |
| R376 | Gag p17 | G2 MVA CD40 | Wk26 | CD8 | 2 cytokines | 0.01002 |
| R376 | Gag p24 | G2 MVA CD40 | Wk10 | CD4 | 2 cytokines | 0.00761 |
| R376 | Gag p24 | G2 MVA CD40 | Wk10 | CD8 | 2 cytokines | 0.14098 |
| R376 | Gag p24 | G2 MVA CD40 | Wk26 | CD4 | 2 cytokines | 0.01399 |
| R376 | Gag p24 | G2 MVA CD40 | Wk26 | CD8 | 2 cytokines | 0.03269 |
| R376 | Nef | G2 MVA CD40 | Wk10 | CD4 | 2 cytokines | 0.00413 |
| R376 | Nef | G2 MVA CD40 | Wk10 | CD8 | 2 cytokines | 0.00399 |
| R376 | Nef | G2 MVA CD40 | Wk26 | CD4 | 2 cytokines | 0.07657 |
| R376 | Nef | G2 MVA CD40 | Wk26 | CD8 | 2 cytokines | 0,0005 |
| R376 | Pol | G2 MVA CD40 | Wk10 | CD4 | 2 cytokines | 0.00052 |
| R376 | Pol | G2 MVA CD40 | Wk10 | CD8 | 2 cytokines | 0.0129 |
| R376 | Pol | G2 MVA CD40 | Wk26 | CD4 | 2 cytokines | 0.00068 |
| R376 | Pol | G2 MVA CD40 | Wk26 | CD8 | 2 cytokines | 0 |
| R377 | Gag p17 | G2 MVA CD40 | Wk10 | CD4 | 2 cytokines | 0.00531 |
| R377 | Gag p17 | G2 MVA CD40 | Wk10 | CD8 | 2 cytokines | 0 |
| R377 | Gag p17 | G2 MVA CD40 | Wk26 | CD4 | 2 cytokines | 0.02777 |
| R377 | Gag p17 | G2 MVA CD40 | Wk26 | CD8 | 2 cytokines | 0,00001 |
| R377 | Gag p24 | G2 MVA CD40 | Wk10 | CD4 | 2 cytokines | 0.01425 |
| R377 | Gag p24 | G2 MVA CD40 | Wk10 | CD8 | 2 cytokines | 0.05464 |
| R377 | Gag p24 | G2 MVA CD40 | Wk26 | CD4 | 2 cytokines | 0.10438 |
| R377 | Gag p24 | G2 MVA CD40 | Wk26 | CD8 | 2 cytokines | 0.03229 |
| R377 | Nef | G2 MVA CD40 | Wk10 | CD4 | 2 cytokines | 0.00292 |
| R377 | Nef | G2 MVA CD40 | Wk10 | CD8 | 2 cytokines | 0.0535 |
| R377 | Nef | G2 MVA CD40 | Wk26 | CD4 | 2 cytokines | 0.07078 |
| R377 | Nef | G2 MVA CD40 | Wk26 | CD8 | 2 cytokines | 0.01637 |
| R377 | Pol | G2 MVA CD40 | Wk10 | CD4 | 2 cytokines | 0.01164 |
| R377 | Pol | G2 MVA CD40 | Wk10 | CD8 | 2 cytokines | 0.05651 |
| R377 | Pol | G2 MVA CD40 | Wk26 | CD4 | 2 cytokines | 0.83457 |
| R377 | Pol | G2 MVA CD40 | Wk26 | CD8 | 2 cytokines | 0.01816 |
| R378 | Gag p17 | G2 MVA CD40 | Wk10 | CD4 | 2 cytokines | 0 |
| R378 | Gag p17 | G2 MVA CD40 | Wk10 | CD8 | 2 cytokines | 0,00008 |
| R378 | Gag p17 | G2 MVA CD40 | Wk26 | CD4 | 2 cytokines | 0.00893 |
| R378 | Gag p17 | G2 MVA CD40 | Wk26 | CD8 | 2 cytokines | 0.00236 |
| R378 | Gag p24 | G2 MVA CD40 | Wk10 | CD4 | 2 cytokines | 0.00263 |
| R378 | Gag p24 | G2 MVA CD40 | Wk10 | CD8 | 2 cytokines | 0.00856 |
| R378 | Gag p24 | G2 MVA CD40 | Wk26 | CD4 | 2 cytokines | 0.06464 |
| R378 | Gag p24 | G2 MVA CD40 | Wk26 | CD8 | 2 cytokines | 0.00295 |
| R378 | Nef | G2 MVA CD40 | Wk10 | CD4 | 2 cytokines | 0.00242 |
| R378 | Nef | G2 MVA CD40 | Wk10 | CD8 | 2 cytokines | 0.01379 |
| R378 | Nef | G2 MVA CD40 | Wk26 | CD4 | 2 cytokines | 0.04647 |
| R378 | Nef | G2 MVA CD40 | Wk26 | CD8 | 2 cytokines | 0.00057 |

| | | | | | | |
|---|---|---|---|---|---|---|
| R378 | Pol | G2 MVA CD40 | Wk10 | CD4 | 2 cytokines | 0.00607 |
| R378 | Pol | G2 MVA CD40 | Wk10 | CD8 | 2 cytokines | 0.01733 |
| R378 | Pol | G2 MVA CD40 | Wk26 | CD4 | 2 cytokines | 0.21792 |
| R378 | Pol | G2 MVA CD40 | Wk26 | CD8 | 2 cytokines | 0.00247 |
| R379 | Gag p17 | G3 DCIR MVA | Wk14 | CD4 | 2 cytokines | 0.00472 |
| R379 | Gag p17 | G3 DCIR MVA | Wk14 | CD8 | 2 cytokines | 0.01605 |
| R379 | Gag p17 | G3 DCIR MVA | Wk24 | CD4 | 2 cytokines | 0.00943 |
| R379 | Gag p17 | G3 DCIR MVA | Wk24 | CD8 | 2 cytokines | 0.01176 |
| R379 | Gag p24 | G3 DCIR MVA | Wk14 | CD4 | 2 cytokines | 0.02216 |
| R379 | Gag p24 | G3 DCIR MVA | Wk14 | CD8 | 2 cytokines | 0.01517 |
| R379 | Gag p24 | G3 DCIR MVA | Wk24 | CD4 | 2 cytokines | 0.04119 |
| R379 | Gag p24 | G3 DCIR MVA | Wk24 | CD8 | 2 cytokines | 0.00476 |
| R379 | Nef | G3 DCIR MVA | Wk14 | CD4 | 2 cytokines | 0.00573 |
| R379 | Nef | G3 DCIR MVA | Wk14 | CD8 | 2 cytokines | 0.00555 |
| R379 | Nef | G3 DCIR MVA | Wk24 | CD4 | 2 cytokines | 0.01892 |
| R379 | Nef | G3 DCIR MVA | Wk24 | CD8 | 2 cytokines | 0.02127 |
| R379 | Pol | G3 DCIR MVA | Wk14 | CD4 | 2 cytokines | 0.13367 |
| R379 | Pol | G3 DCIR MVA | Wk14 | CD8 | 2 cytokines | 0.02395 |
| R379 | Pol | G3 DCIR MVA | Wk24 | CD4 | 2 cytokines | 0.24059 |
| R379 | Pol | G3 DCIR MVA | Wk24 | CD8 | 2 cytokines | 0.027 |
| R380 | Gag p17 | G2 MVA CD40 | Wk10 | CD4 | 2 cytokines | 0.01403 |
| R380 | Gag p17 | G2 MVA CD40 | Wk10 | CD8 | 2 cytokines | 0 |
| R380 | Gag p17 | G2 MVA CD40 | Wk26 | CD4 | 2 cytokines | 0.00558 |
| R380 | Gag p17 | G2 MVA CD40 | Wk26 | CD8 | 2 cytokines | 0.01511 |
| R380 | Gag p24 | G2 MVA CD40 | Wk10 | CD4 | 2 cytokines | 0.0124 |
| R380 | Gag p24 | G2 MVA CD40 | Wk10 | CD8 | 2 cytokines | 0.00778 |
| R380 | Gag p24 | G2 MVA CD40 | Wk26 | CD4 | 2 cytokines | 0.23177 |
| R380 | Gag p24 | G2 MVA CD40 | Wk26 | CD8 | 2 cytokines | 0.00461 |
| R380 | Nef | G2 MVA CD40 | Wk10 | CD4 | 2 cytokines | 0.00795 |
| R380 | Nef | G2 MVA CD40 | Wk10 | CD8 | 2 cytokines | 0.00383 |
| R380 | Nef | G2 MVA CD40 | Wk26 | CD4 | 2 cytokines | 0.11337 |
| R380 | Nef | G2 MVA CD40 | Wk26 | CD8 | 2 cytokines | 0.00259 |
| R380 | Pol | G2 MVA CD40 | Wk10 | CD4 | 2 cytokines | 0.01317 |
| R380 | Pol | G2 MVA CD40 | Wk10 | CD8 | 2 cytokines | 0.0026 |
| R380 | Pol | G2 MVA CD40 | Wk26 | CD4 | 2 cytokines | 0.03049 |
| R380 | Pol | G2 MVA CD40 | Wk26 | CD8 | 2 cytokines | 0.00552 |
| R381 | Gag p17 | G3 DCIR MVA | Wk14 | CD4 | 2 cytokines | 0.00068 |
| R381 | Gag p17 | G3 DCIR MVA | Wk14 | CD8 | 2 cytokines | 0.00185 |
| R381 | Gag p17 | G3 DCIR MVA | Wk24 | CD4 | 2 cytokines | 0.00305 |
| R381 | Gag p17 | G3 DCIR MVA | Wk24 | CD8 | 2 cytokines | 0.01767 |
| R381 | Gag p24 | G3 DCIR MVA | Wk14 | CD4 | 2 cytokines | 0.0049 |
| R381 | Gag p24 | G3 DCIR MVA | Wk14 | CD8 | 2 cytokines | 6.7e-05 |
| R381 | Gag p24 | G3 DCIR MVA | Wk24 | CD4 | 2 cytokines | 0.01021 |
| R381 | Gag p24 | G3 DCIR MVA | Wk24 | CD8 | 2 cytokines | 0.01997 |
| R381 | Nef | G3 DCIR MVA | Wk14 | CD4 | 2 cytokines | 0.0023 |
| R381 | Nef | G3 DCIR MVA | Wk14 | CD8 | 2 cytokines | 0.0027 |
| R381 | Pol | G3 DCIR MVA | Wk14 | CD4 | 2 cytokines | 0.13642 |
| R381 | Pol | G3 DCIR MVA | Wk14 | CD8 | 2 cytokines | 0.004 |
| R381 | Pol | G3 DCIR MVA | Wk24 | CD4 | 2 cytokines | 0.07772 |
| R381 | Pol | G3 DCIR MVA | Wk24 | CD8 | 2 cytokines | 0.02187 |
| R382 | Gag p17 | G2 MVA CD40 | Wk10 | CD4 | 2 cytokines | 0.00949 |
| R382 | Gag p17 | G2 MVA CD40 | Wk10 | CD8 | 2 cytokines | 0.00416 |
| R382 | Gag p17 | G2 MVA CD40 | Wk26 | CD4 | 2 cytokines | 0.00819 |
| R382 | Gag p17 | G2 MVA CD40 | Wk26 | CD8 | 2 cytokines | 0.00074 |
| R382 | Gag p24 | G2 MVA CD40 | Wk10 | CD4 | 2 cytokines | 0.01004 |
| R382 | Gag p24 | G2 MVA CD40 | Wk10 | CD8 | 2 cytokines | 0.01364 |
| R382 | Gag p24 | G2 MVA CD40 | Wk26 | CD4 | 2 cytokines | 0.15097 |
| R382 | Gag p24 | G2 MVA CD40 | Wk26 | CD8 | 2 cytokines | 0.00747 |

| | | | | | | |
|---|---|---|---|---|---|---|
| R382 | Nef | G2 MVA CD40 | Wk10 | CD4 | 2 cytokines | 0.01617 |
| R382 | Nef | G2 MVA CD40 | Wk10 | CD8 | 2 cytokines | 0.04367 |
| R382 | Nef | G2 MVA CD40 | Wk26 | CD4 | 2 cytokines | 0.17801 |
| R382 | Nef | G2 MVA CD40 | Wk26 | CD8 | 2 cytokines | 0.01132 |
| R382 | Pol | G2 MVA CD40 | Wk10 | CD4 | 2 cytokines | 0.01713 |
| R382 | Pol | G2 MVA CD40 | Wk10 | CD8 | 2 cytokines | 0.06394 |
| R382 | Pol | G2 MVA CD40 | Wk26 | CD4 | 2 cytokines | 0.27666 |
| R382 | Pol | G2 MVA CD40 | Wk26 | CD8 | 2 cytokines | 0.02903 |
| R383 | Gag p17 | G4 CD40 MVA | Wk14 | CD4 | 2 cytokines | 0.00966 |
| R383 | Gag p17 | G4 CD40 MVA | Wk14 | CD8 | 2 cytokines | 0.00468 |
| R383 | Gag p17 | G4 CD40 MVA | Wk24 | CD4 | 2 cytokines | 0.01207 |
| R383 | Gag p17 | G4 CD40 MVA | Wk24 | CD8 | 2 cytokines | 0.00333 |
| R383 | Gag p24 | G4 CD40 MVA | Wk14 | CD4 | 2 cytokines | 0.03019 |
| R383 | Gag p24 | G4 CD40 MVA | Wk14 | CD8 | 2 cytokines | 0.0027 |
| R383 | Gag p24 | G4 CD40 MVA | Wk24 | CD4 | 2 cytokines | 0.09113 |
| R383 | Gag p24 | G4 CD40 MVA | Wk24 | CD8 | 2 cytokines | 0.05055 |
| R383 | Nef | G4 CD40 MVA | Wk14 | CD4 | 2 cytokines | 0.0489 |
| R383 | Nef | G4 CD40 MVA | Wk14 | CD8 | 2 cytokines | 0.00111 |
| R383 | Nef | G4 CD40 MVA | Wk24 | CD4 | 2 cytokines | 0.13006 |
| R383 | Nef | G4 CD40 MVA | Wk24 | CD8 | 2 cytokines | 0.0291 |
| R383 | Pol | G4 CD40 MVA | Wk14 | CD4 | 2 cytokines | 0.07364 |
| R383 | Pol | G4 CD40 MVA | Wk14 | CD8 | 2 cytokines | 0.00376 |
| R383 | Pol | G4 CD40 MVA | Wk24 | CD4 | 2 cytokines | 0.28795 |
| R383 | Pol | G4 CD40 MVA | Wk24 | CD8 | 2 cytokines | 0.0203 |
| R384 | Gag p17 | G2 MVA CD40 | Wk26 | CD4 | 2 cytokines | 0.01385 |
| R384 | Gag p17 | G2 MVA CD40 | Wk26 | CD8 | 2 cytokines | 0.02098 |
| R384 | Gag p24 | G2 MVA CD40 | Wk10 | CD4 | 2 cytokines | 0.00267 |
| R384 | Gag p24 | G2 MVA CD40 | Wk10 | CD8 | 2 cytokines | 0.01797 |
| R384 | Gag p24 | G2 MVA CD40 | Wk26 | CD4 | 2 cytokines | 0.26504 |
| R384 | Gag p24 | G2 MVA CD40 | Wk26 | CD8 | 2 cytokines | 0.04681 |
| R384 | Nef | G2 MVA CD40 | Wk10 | CD4 | 2 cytokines | 0.00155 |
| R384 | Nef | G2 MVA CD40 | Wk10 | CD8 | 2 cytokines | 0.0074 |
| R384 | Nef | G2 MVA CD40 | Wk26 | CD4 | 2 cytokines | 0.22704 |
| R384 | Nef | G2 MVA CD40 | Wk26 | CD8 | 2 cytokines | 0.03869 |
| R384 | Pol | G2 MVA CD40 | Wk10 | CD4 | 2 cytokines | 0.00612 |
| R384 | Pol | G2 MVA CD40 | Wk10 | CD8 | 2 cytokines | 0.00844 |
| R384 | Pol | G2 MVA CD40 | Wk26 | CD4 | 2 cytokines | 0.13734 |
| R384 | Pol | G2 MVA CD40 | Wk26 | CD8 | 2 cytokines | 0.02548 |
| R385 | Gag p17 | G1 MVA DCIR | Wk10 | CD4 | 2 cytokines | 0.00439 |
| R385 | Gag p17 | G1 MVA DCIR | Wk10 | CD8 | 2 cytokines | 0.00166 |
| R385 | Gag p17 | G1 MVA DCIR | Wk26 | CD4 | 2 cytokines | 0.01682 |
| R385 | Gag p17 | G1 MVA DCIR | Wk26 | CD8 | 2 cytokines | 0.01153 |
| R385 | Gag p24 | G1 MVA DCIR | Wk10 | CD4 | 2 cytokines | 0 |
| R385 | Gag p24 | G1 MVA DCIR | Wk10 | CD8 | 2 cytokines | 0.01453 |
| R385 | Gag p24 | G1 MVA DCIR | Wk26 | CD4 | 2 cytokines | 0.04912 |
| R385 | Gag p24 | G1 MVA DCIR | Wk26 | CD8 | 2 cytokines | 0.01065 |
| R385 | Nef | G1 MVA DCIR | Wk10 | CD4 | 2 cytokines | 0.00661 |
| R385 | Nef | G1 MVA DCIR | Wk10 | CD8 | 2 cytokines | 0.0032 |
| R385 | Nef | G1 MVA DCIR | Wk26 | CD4 | 2 cytokines | 0.03627 |
| R385 | Nef | G1 MVA DCIR | Wk26 | CD8 | 2 cytokines | 0.00329 |
| R385 | Pol | G1 MVA DCIR | Wk10 | CD4 | 2 cytokines | 0.00418 |
| R385 | Pol | G1 MVA DCIR | Wk10 | CD8 | 2 cytokines | 0 |
| R385 | Pol | G1 MVA DCIR | Wk26 | CD4 | 2 cytokines | 0.10681 |
| R385 | Pol | G1 MVA DCIR | Wk26 | CD8 | 2 cytokines | 0.01212 |
| R386 | Gag p17 | G4 CD40 MVA | Wk14 | CD4 | 2 cytokines | 0.01184 |
| R386 | Gag p17 | G4 CD40 MVA | Wk14 | CD8 | 2 cytokines | 0.06657 |
| R386 | Gag p17 | G4 CD40 MVA | Wk24 | CD4 | 2 cytokines | 0.00732 |
| R386 | Gag p17 | G4 CD40 MVA | Wk24 | CD8 | 2 cytokines | 0.05745 |

| | | | | | | |
|---|---|---|---|---|---|---|
| R386 | Gag p24 | G4 CD40 MVA | Wk14 | CD4 | 2 cytokines | 0.02628 |
| R386 | Gag p24 | G4 CD40 MVA | Wk14 | CD8 | 2 cytokines | 0.01111 |
| R386 | Gag p24 | G4 CD40 MVA | Wk24 | CD4 | 2 cytokines | 0.02886 |
| R386 | Gag p24 | G4 CD40 MVA | Wk24 | CD8 | 2 cytokines | 0.00608 |
| R386 | Nef | G4 CD40 MVA | Wk14 | CD4 | 2 cytokines | 0.00814 |
| R386 | Nef | G4 CD40 MVA | Wk14 | CD8 | 2 cytokines | 0.0069 |
| R386 | Nef | G4 CD40 MVA | Wk24 | CD4 | 2 cytokines | 0.02034 |
| R386 | Nef | G4 CD40 MVA | Wk24 | CD8 | 2 cytokines | 0.00080 |
| R386 | Pol | G4 CD40 MVA | Wk14 | CD4 | 2 cytokines | 0.04731 |
| R386 | Pol | G4 CD40 MVA | Wk14 | CD8 | 2 cytokines | 0.00134 |
| R386 | Pol | G4 CD40 MVA | Wk24 | CD4 | 2 cytokines | 0.03875 |
| R386 | Pol | G4 CD40 MVA | Wk24 | CD8 | 2 cytokines | 0.00071 |
| R388 | Gag p17 | G3 DCIR MVA | Wk24 | CD4 | 2 cytokines | 0.0345 |
| R388 | Gag p17 | G3 DCIR MVA | Wk24 | CD8 | 2 cytokines | 0.01009 |
| R388 | Gag p24 | G3 DCIR MVA | Wk24 | CD4 | 2 cytokines | 0.05864 |
| R388 | Gag p24 | G3 DCIR MVA | Wk24 | CD8 | 2 cytokines | 0.01213 |
| R389 | Gag p17 | G4 CD40 MVA | Wk24 | CD4 | 2 cytokines | 0.01135 |
| R389 | Gag p17 | G4 CD40 MVA | Wk24 | CD8 | 2 cytokines | 0.0348 |
| R389 | Gag p24 | G4 CD40 MVA | Wk24 | CD4 | 2 cytokines | 0.0551 |
| R389 | Gag p24 | G4 CD40 MVA | Wk24 | CD8 | 2 cytokines | 0.00276 |
| R389 | Pol | G4 CD40 MVA | Wk24 | CD4 | 2 cytokines | 0.22322 |
| R389 | Pol | G4 CD40 MVA | Wk24 | CD8 | 2 cytokines | 0.02219 |
| R390 | Gag p17 | G4 CD40 MVA | Wk14 | CD4 | 2 cytokines | 0.00103 |
| R390 | Gag p17 | G4 CD40 MVA | Wk14 | CD8 | 2 cytokines | 0.00295 |
| R390 | Gag p17 | G4 CD40 MVA | Wk24 | CD4 | 2 cytokines | 0.00388 |
| R390 | Gag p17 | G4 CD40 MVA | Wk24 | CD8 | 2 cytokines | 0.00926 |
| R390 | Gag p24 | G4 CD40 MVA | Wk14 | CD4 | 2 cytokines | 0.00395 |
| R390 | Gag p24 | G4 CD40 MVA | Wk14 | CD8 | 2 cytokines | 0 |
| R390 | Gag p24 | G4 CD40 MVA | Wk24 | CD4 | 2 cytokines | 0.01454 |
| R390 | Gag p24 | G4 CD40 MVA | Wk24 | CD8 | 2 cytokines | 0.0179 |
| R390 | Nef | G4 CD40 MVA | Wk14 | CD4 | 2 cytokines | 0.00427 |
| R390 | Nef | G4 CD40 MVA | Wk14 | CD8 | 2 cytokines | 0.00275 |
| R390 | Nef | G4 CD40 MVA | Wk24 | CD4 | 2 cytokines | 0.01144 |
| R390 | Nef | G4 CD40 MVA | Wk24 | CD8 | 2 cytokines | 0.01031 |
| R390 | Pol | G4 CD40 MVA | Wk14 | CD4 | 2 cytokines | 0.00735 |
| R390 | Pol | G4 CD40 MVA | Wk14 | CD8 | 2 cytokines | 0.01152 |
| R390 | Pol | G4 CD40 MVA | Wk24 | CD4 | 2 cytokines | 0.03361 |
| R390 | Pol | G4 CD40 MVA | Wk24 | CD8 | 2 cytokines | 0.0059 |
| R391 | Gag p17 | G4 CD40 MVA | Wk14 | CD4 | 2 cytokines | 0.00808 |
| R391 | Gag p17 | G4 CD40 MVA | Wk14 | CD8 | 2 cytokines | 0.0154 |
| R391 | Gag p17 | G4 CD40 MVA | Wk24 | CD4 | 2 cytokines | 0.01478 |
| R391 | Gag p17 | G4 CD40 MVA | Wk24 | CD8 | 2 cytokines | 0.00207 |
| R391 | Gag p24 | G4 CD40 MVA | Wk14 | CD4 | 2 cytokines | 0.04681 |
| R391 | Gag p24 | G4 CD40 MVA | Wk14 | CD8 | 2 cytokines | 0.0075 |
| R391 | Gag p24 | G4 CD40 MVA | Wk24 | CD4 | 2 cytokines | 0.04663 |
| R391 | Gag p24 | G4 CD40 MVA | Wk24 | CD8 | 2 cytokines | 0.02228 |
| R368 | Gag p17 | G3 DCIR MVA | Wk14 | CD4 | 3 cytokines | 0 |
| R368 | Gag p17 | G3 DCIR MVA | Wk14 | CD8 | 3 cytokines | 0 |
| R368 | Gag p17 | G3 DCIR MVA | Wk24 | CD4 | 3 cytokines | 0.00031 |
| R368 | Gag p17 | G3 DCIR MVA | Wk24 | CD8 | 3 cytokines | 0.00022 |
| R368 | Gag p24 | G3 DCIR MVA | Wk14 | CD4 | 3 cytokines | 0 |
| R368 | Gag p24 | G3 DCIR MVA | Wk14 | CD8 | 3 cytokines | 0 |
| R368 | Gag p24 | G3 DCIR MVA | Wk24 | CD4 | 3 cytokines | 0.00666 |
| R368 | Gag p24 | G3 DCIR MVA | Wk24 | CD8 | 3 cytokines | 0.00002 |
| R368 | Nef | G3 DCIR MVA | Wk14 | CD4 | 3 cytokines | 0.01872 |
| R368 | Nef | G3 DCIR MVA | Wk14 | CD8 | 3 cytokines | 0 |
| R368 | Nef | G3 DCIR MVA | Wk24 | CD4 | 3 cytokines | 0.03457 |
| R368 | Nef | G3 DCIR MVA | Wk24 | CD8 | 3 cytokines | 0.01045 |

| | | | | | | |
|---|---|---|---|---|---|---|
| R368 | Pol | G3 DCIR MVA | Wk14 | CD4 | 3 cytokines | 0.01202 |
| R368 | Pol | G3 DCIR MVA | Wk14 | CD8 | 3 cytokines | 0 |
| R368 | Pol | G3 DCIR MVA | Wk24 | CD4 | 3 cytokines | 0.01217 |
| R368 | Pol | G3 DCIR MVA | Wk24 | CD8 | 3 cytokines | 0.00193 |
| R369 | Gag p17 | G1 MVA DCIR | Wk10 | CD4 | 3 cytokines | 0.00522 |
| R369 | Gag p17 | G1 MVA DCIR | Wk10 | CD8 | 3 cytokines | 0.00076 |
| R369 | Gag p17 | G1 MVA DCIR | Wk26 | CD4 | 3 cytokines | 0.00776 |
| R369 | Gag p17 | G1 MVA DCIR | Wk26 | CD8 | 3 cytokines | 0 |
| R369 | Gag p24 | G1 MVA DCIR | Wk10 | CD4 | 3 cytokines | 0.00241 |
| R369 | Gag p24 | G1 MVA DCIR | Wk10 | CD8 | 3 cytokines | 0.01653 |
| R369 | Gag p24 | G1 MVA DCIR | Wk26 | CD4 | 3 cytokines | 0.23436 |
| R369 | Gag p24 | G1 MVA DCIR | Wk26 | CD8 | 3 cytokines | 0.0347 |
| R369 | Nef | G1 MVA DCIR | Wk10 | CD4 | 3 cytokines | 0.00188 |
| R369 | Nef | G1 MVA DCIR | Wk10 | CD8 | 3 cytokines | 0.00322 |
| R369 | Nef | G1 MVA DCIR | Wk26 | CD4 | 3 cytokines | 0.20636 |
| R369 | Nef | G1 MVA DCIR | Wk26 | CD8 | 3 cytokines | 0.01313 |
| R369 | Pol | G1 MVA DCIR | Wk10 | CD4 | 3 cytokines | 0.00229 |
| R369 | Pol | G1 MVA DCIR | Wk10 | CD8 | 3 cytokines | 0.00283 |
| R369 | Pol | G1 MVA DCIR | Wk26 | CD4 | 3 cytokines | 0.00389 |
| R369 | Pol | G1 MVA DCIR | Wk26 | CD8 | 3 cytokines | 0.006 |
| R370 | Gag p17 | G1 MVA DCIR | Wk10 | CD4 | 3 cytokines | 0.00068 |
| R370 | Gag p17 | G1 MVA DCIR | Wk10 | CD8 | 3 cytokines | 0 |
| R370 | Gag p17 | G1 MVA DCIR | Wk26 | CD4 | 3 cytokines | 0 |
| R370 | Gag p17 | G1 MVA DCIR | Wk26 | CD8 | 3 cytokines | 0 |
| R370 | Gag p24 | G1 MVA DCIR | Wk10 | CD4 | 3 cytokines | 0.00057 |
| R370 | Gag p24 | G1 MVA DCIR | Wk10 | CD8 | 3 cytokines | 0.00121 |
| R370 | Gag p24 | G1 MVA DCIR | Wk26 | CD4 | 3 cytokines | 0.00065 |
| R370 | Gag p24 | G1 MVA DCIR | Wk26 | CD8 | 3 cytokines | 0.00169 |
| R370 | Nef | G1 MVA DCIR | Wk10 | CD4 | 3 cytokines | 0.00101 |
| R370 | Nef | G1 MVA DCIR | Wk10 | CD8 | 3 cytokines | 0 |
| R370 | Nef | G1 MVA DCIR | Wk26 | CD4 | 3 cytokines | 0.00247 |
| R370 | Nef | G1 MVA DCIR | Wk26 | CD8 | 3 cytokines | 0 |
| R370 | Pol | G1 MVA DCIR | Wk10 | CD4 | 3 cytokines | 0.00071 |
| R370 | Pol | G1 MVA DCIR | Wk10 | CD8 | 3 cytokines | 0,00005 |
| R370 | Pol | G1 MVA DCIR | Wk26 | CD4 | 3 cytokines | 0.0126 |
| R370 | Pol | G1 MVA DCIR | Wk26 | CD8 | 3 cytokines | 0 |
| R371 | Gag p17 | G1 MVA DCIR | Wk10 | CD4 | 3 cytokines | 0.00146 |
| R371 | Gag p17 | G1 MVA DCIR | Wk10 | CD8 | 3 cytokines | 0.00185 |
| R371 | Gag p17 | G1 MVA DCIR | Wk26 | CD4 | 3 cytokines | 0 |
| R371 | Gag p17 | G1 MVA DCIR | Wk26 | CD8 | 3 cytokines | 0 |
| R371 | Gag p24 | G1 MVA DCIR | Wk10 | CD4 | 3 cytokines | 0.00178 |
| R371 | Gag p24 | G1 MVA DCIR | Wk10 | CD8 | 3 cytokines | 0.034 |
| R371 | Gag p24 | G1 MVA DCIR | Wk26 | CD4 | 3 cytokines | 0.015 |
| R371 | Gag p24 | G1 MVA DCIR | Wk26 | CD8 | 3 cytokines | 0.01466 |
| R371 | Nef | G1 MVA DCIR | Wk10 | CD4 | 3 cytokines | 0.00136 |
| R371 | Nef | G1 MVA DCIR | Wk10 | CD8 | 3 cytokines | 0.00263 |
| R371 | Nef | G1 MVA DCIR | Wk26 | CD4 | 3 cytokines | 0.00791 |
| R371 | Nef | G1 MVA DCIR | Wk26 | CD8 | 3 cytokines | 0.00167 |
| R371 | Pol | G1 MVA DCIR | Wk10 | CD4 | 3 cytokines | 0.00312 |
| R371 | Pol | G1 MVA DCIR | Wk10 | CD8 | 3 cytokines | 0.00411 |
| R371 | Pol | G1 MVA DCIR | Wk26 | CD4 | 3 cytokines | 0.0696 |
| R371 | Pol | G1 MVA DCIR | Wk26 | CD8 | 3 cytokines | 0.00289 |
| R372 | Gag p17 | G4 CD40 MVA | Wk14 | CD4 | 3 cytokines | 0.00081 |
| R372 | Gag p17 | G4 CD40 MVA | Wk14 | CD8 | 3 cytokines | 0 |
| R372 | Gag p17 | G4 CD40 MVA | Wk24 | CD4 | 3 cytokines | 0.00782 |
| R372 | Gag p17 | G4 CD40 MVA | Wk24 | CD8 | 3 cytokines | 0.0089 |
| R372 | Gag p24 | G4 CD40 MVA | Wk14 | CD4 | 3 cytokines | 0.0069 |
| R372 | Gag p24 | G4 CD40 MVA | Wk14 | CD8 | 3 cytokines | 0.00383 |

| | | | | | | |
|---|---|---|---|---|---|---|
| R372 | Gag p24 | G4 CD40 MVA | Wk24 | CD4 | 3 cytokines | 0.03202 |
| R372 | Gag p24 | G4 CD40 MVA | Wk24 | CD8 | 3 cytokines | 0.01125 |
| R372 | Nef | G4 CD40 MVA | Wk14 | CD4 | 3 cytokines | 0.00463 |
| R372 | Nef | G4 CD40 MVA | Wk14 | CD8 | 3 cytokines | 0 |
| R372 | Nef | G4 CD40 MVA | Wk24 | CD4 | 3 cytokines | 0.02132 |
| R372 | Nef | G4 CD40 MVA | Wk24 | CD8 | 3 cytokines | 0 |
| R372 | Pol | G4 CD40 MVA | Wk14 | CD4 | 3 cytokines | 0.0223 |
| R372 | Pol | G4 CD40 MVA | Wk14 | CD8 | 3 cytokines | 0 |
| R372 | Pol | G4 CD40 MVA | Wk24 | CD4 | 3 cytokines | 0.10052 |
| R372 | Pol | G4 CD40 MVA | Wk24 | CD8 | 3 cytokines | 0 |
| R373 | Gag p17 | G1 MVA DCIR | Wk10 | CD4 | 3 cytokines | 0.00162 |
| R373 | Gag p17 | G1 MVA DCIR | Wk10 | CD8 | 3 cytokines | 0.00054 |
| R373 | Gag p17 | G1 MVA DCIR | Wk26 | CD4 | 3 cytokines | 0 |
| R373 | Gag p17 | G1 MVA DCIR | Wk26 | CD8 | 3 cytokines | 0.00168 |
| R373 | Gag p24 | G1 MVA DCIR | Wk10 | CD4 | 3 cytokines | 0.00006 |
| R373 | Gag p24 | G1 MVA DCIR | Wk10 | CD8 | 3 cytokines | 0.00257 |
| R373 | Gag p24 | G1 MVA DCIR | Wk26 | CD4 | 3 cytokines | 0.02518 |
| R373 | Gag p24 | G1 MVA DCIR | Wk26 | CD8 | 3 cytokines | 0 |
| R373 | Nef | G1 MVA DCIR | Wk10 | CD4 | 3 cytokines | 0 |
| R373 | Nef | G1 MVA DCIR | Wk10 | CD8 | 3 cytokines | 0.00063 |
| R373 | Nef | G1 MVA DCIR | Wk26 | CD4 | 3 cytokines | 0.04538 |
| R373 | Nef | G1 MVA DCIR | Wk26 | CD8 | 3 cytokines | 0.00177 |
| R373 | Pol | G1 MVA DCIR | Wk10 | CD4 | 3 cytokines | 0 |
| R373 | Pol | G1 MVA DCIR | Wk10 | CD8 | 3 cytokines | 0.00161 |
| R373 | Pol | G1 MVA DCIR | Wk26 | CD4 | 3 cytokines | 0 |
| R373 | Pol | G1 MVA DCIR | Wk26 | CD8 | 3 cytokines | 0.00171 |
| R374 | Gag p17 | G1 MVA DCIR | Wk10 | CD4 | 3 cytokines | 0.01087 |
| R374 | Gag p17 | G1 MVA DCIR | Wk10 | CD8 | 3 cytokines | 0.00047 |
| R374 | Gag p17 | G1 MVA DCIR | Wk26 | CD4 | 3 cytokines | 0.01164 |
| R374 | Gag p17 | G1 MVA DCIR | Wk26 | CD8 | 3 cytokines | 0 |
| R374 | Gag p24 | G1 MVA DCIR | Wk10 | CD4 | 3 cytokines | 0.00577 |
| R374 | Gag p24 | G1 MVA DCIR | Wk10 | CD8 | 3 cytokines | 0.00282 |
| R374 | Gag p24 | G1 MVA DCIR | Wk26 | CD4 | 3 cytokines | 0.19874 |
| R374 | Gag p24 | G1 MVA DCIR | Wk26 | CD8 | 3 cytokines | 0.00456 |
| R374 | Nef | G1 MVA DCIR | Wk10 | CD4 | 3 cytokines | 0.00133 |
| R374 | Nef | G1 MVA DCIR | Wk10 | CD8 | 3 cytokines | 0.00509 |
| R374 | Nef | G1 MVA DCIR | Wk26 | CD4 | 3 cytokines | 0.04194 |
| R374 | Nef | G1 MVA DCIR | Wk26 | CD8 | 3 cytokines | 0.00272 |
| R374 | Pol | G1 MVA DCIR | Wk10 | CD4 | 3 cytokines | 0.00028 |
| R374 | Pol | G1 MVA DCIR | Wk10 | CD8 | 3 cytokines | 0.00146 |
| R374 | Pol | G1 MVA DCIR | Wk26 | CD4 | 3 cytokines | 0.0041 |
| R374 | Pol | G1 MVA DCIR | Wk26 | CD8 | 3 cytokines | 0.00149 |
| R375 | Gag p17 | G3 DCIR MVA | Wk14 | CD4 | 3 cytokines | 0 |
| R375 | Gag p17 | G3 DCIR MVA | Wk14 | CD8 | 3 cytokines | 0 |
| R375 | Gag p24 | G3 DCIR MVA | Wk14 | CD4 | 3 cytokines | 0 |
| R375 | Gag p24 | G3 DCIR MVA | Wk14 | CD8 | 3 cytokines | 0 |
| R375 | Gag p24 | G3 DCIR MVA | Wk24 | CD4 | 3 cytokines | 0.0031 |
| R375 | Gag p24 | G3 DCIR MVA | Wk24 | CD8 | 3 cytokines | 0 |
| R375 | Nef | G3 DCIR MVA | Wk14 | CD4 | 3 cytokines | 0.01233 |
| R375 | Nef | G3 DCIR MVA | Wk14 | CD8 | 3 cytokines | 0 |
| R375 | Nef | G3 DCIR MVA | Wk24 | CD4 | 3 cytokines | 0.01943 |
| R375 | Nef | G3 DCIR MVA | Wk24 | CD8 | 3 cytokines | 0.00044 |
| R375 | Pol | G3 DCIR MVA | Wk14 | CD4 | 3 cytokines | 0 |
| R375 | Pol | G3 DCIR MVA | Wk14 | CD8 | 3 cytokines | 0 |
| R375 | Pol | G3 DCIR MVA | Wk24 | CD4 | 3 cytokines | 0.04003 |
| R375 | Pol | G3 DCIR MVA | Wk24 | CD8 | 3 cytokines | 0 |
| R376 | Gag p17 | G2 MVA CD40 | Wk10 | CD4 | 3 cytokines | 0.00147 |
| R376 | Gag p17 | G2 MVA CD40 | Wk10 | CD8 | 3 cytokines | 0 |

| | | | | | | |
|---|---|---|---|---|---|---|
| R376 | Gag p17 | G2 MVA CD40 | Wk26 | CD4 | 3 cytokines | 0 |
| R376 | Gag p17 | G2 MVA CD40 | Wk26 | CD8 | 3 cytokines | 0 |
| R376 | Gag p24 | G2 MVA CD40 | Wk10 | CD4 | 3 cytokines | 0.00192 |
| R376 | Gag p24 | G2 MVA CD40 | Wk10 | CD8 | 3 cytokines | 0.01564 |
| R376 | Gag p24 | G2 MVA CD40 | Wk26 | CD4 | 3 cytokines | 0.00543 |
| R376 | Gag p24 | G2 MVA CD40 | Wk26 | CD8 | 3 cytokines | 0.00449 |
| R376 | Nef | G2 MVA CD40 | Wk10 | CD4 | 3 cytokines | 0.00054 |
| R376 | Nef | G2 MVA CD40 | Wk10 | CD8 | 3 cytokines | 0 |
| R376 | Nef | G2 MVA CD40 | Wk26 | CD4 | 3 cytokines | 0.04788 |
| R376 | Nef | G2 MVA CD40 | Wk26 | CD8 | 3 cytokines | 0 |
| R376 | Pol | G2 MVA CD40 | Wk10 | CD4 | 3 cytokines | 0.00026 |
| R376 | Pol | G2 MVA CD40 | Wk10 | CD8 | 3 cytokines | 0.00159 |
| R376 | Pol | G2 MVA CD40 | Wk26 | CD4 | 3 cytokines | 0 |
| R376 | Pol | G2 MVA CD40 | Wk26 | CD8 | 3 cytokines | 0 |
| R377 | Gag p17 | G2 MVA CD40 | Wk10 | CD4 | 3 cytokines | 0.00501 |
| R377 | Gag p17 | G2 MVA CD40 | Wk10 | CD8 | 3 cytokines | 0 |
| R377 | Gag p17 | G2 MVA CD40 | Wk26 | CD4 | 3 cytokines | 0.00748 |
| R377 | Gag p17 | G2 MVA CD40 | Wk26 | CD8 | 3 cytokines | 0 |
| R377 | Gag p24 | G2 MVA CD40 | Wk10 | CD4 | 3 cytokines | 0.01131 |
| R377 | Gag p24 | G2 MVA CD40 | Wk10 | CD8 | 3 cytokines | 0.02115 |
| R377 | Gag p24 | G2 MVA CD40 | Wk26 | CD4 | 3 cytokines | 0.06048 |
| R377 | Gag p24 | G2 MVA CD40 | Wk26 | CD8 | 3 cytokines | 0.01844 |
| R377 | Nef | G2 MVA CD40 | Wk10 | CD4 | 3 cytokines | 0.00028 |
| R377 | Nef | G2 MVA CD40 | Wk10 | CD8 | 3 cytokines | 0.02629 |
| R377 | Nef | G2 MVA CD40 | Wk26 | CD4 | 3 cytokines | 0.04348 |
| R377 | Nef | G2 MVA CD40 | Wk26 | CD8 | 3 cytokines | 0.00787 |
| R377 | Pol | G2 MVA CD40 | Wk10 | CD4 | 3 cytokines | 0.01071 |
| R377 | Pol | G2 MVA CD40 | Wk10 | CD8 | 3 cytokines | 0.02261 |
| R377 | Pol | G2 MVA CD40 | Wk26 | CD4 | 3 cytokines | 0.61978 |
| R377 | Pol | G2 MVA CD40 | Wk26 | CD8 | 3 cytokines | 0.00658 |
| R378 | Gag p17 | G2 MVA CD40 | Wk10 | CD4 | 3 cytokines | 0 |
| R378 | Gag p17 | G2 MVA CD40 | Wk10 | CD8 | 3 cytokines | 0,00008 |
| R378 | Gag p17 | G2 MVA CD40 | Wk26 | CD4 | 3 cytokines | 0.00131 |
| R378 | Gag p17 | G2 MVA CD40 | Wk26 | CD8 | 3 cytokines | 0.00074 |
| R378 | Gag p24 | G2 MVA CD40 | Wk10 | CD4 | 3 cytokines | 0.00044 |
| R378 | Gag p24 | G2 MVA CD40 | Wk10 | CD8 | 3 cytokines | 0.0062 |
| R378 | Gag p24 | G2 MVA CD40 | Wk26 | CD4 | 3 cytokines | 0.03339 |
| R378 | Gag p24 | G2 MVA CD40 | Wk26 | CD8 | 3 cytokines | 0.00143 |
| R378 | Nef | G2 MVA CD40 | Wk10 | CD4 | 3 cytokines | 0.00129 |
| R378 | Nef | G2 MVA CD40 | Wk10 | CD8 | 3 cytokines | 0 |
| R378 | Nef | G2 MVA CD40 | Wk26 | CD4 | 3 cytokines | 0.02269 |
| R378 | Nef | G2 MVA CD40 | Wk26 | CD8 | 3 cytokines | 0 |
| R378 | Pol | G2 MVA CD40 | Wk10 | CD4 | 3 cytokines | 0.00561 |
| R378 | Pol | G2 MVA CD40 | Wk10 | CD8 | 3 cytokines | 0.00625 |
| R378 | Pol | G2 MVA CD40 | Wk26 | CD4 | 3 cytokines | 0.16849 |
| R378 | Pol | G2 MVA CD40 | Wk26 | CD8 | 3 cytokines | 0.00092 |
| R379 | Gag p17 | G3 DCIR MVA | Wk14 | CD4 | 3 cytokines | 0.00243 |
| R379 | Gag p17 | G3 DCIR MVA | Wk14 | CD8 | 3 cytokines | 0.00374 |
| R379 | Gag p17 | G3 DCIR MVA | Wk24 | CD4 | 3 cytokines | 0.00543 |
| R379 | Gag p17 | G3 DCIR MVA | Wk24 | CD8 | 3 cytokines | 0.00325 |
| R379 | Gag p24 | G3 DCIR MVA | Wk14 | CD4 | 3 cytokines | 0.01379 |
| R379 | Gag p24 | G3 DCIR MVA | Wk14 | CD8 | 3 cytokines | 0.0018 |
| R379 | Gag p24 | G3 DCIR MVA | Wk24 | CD4 | 3 cytokines | 0.0288 |
| R379 | Gag p24 | G3 DCIR MVA | Wk24 | CD8 | 3 cytokines | 0.00314 |
| R379 | Nef | G3 DCIR MVA | Wk14 | CD4 | 3 cytokines | 0.00399 |
| R379 | Nef | G3 DCIR MVA | Wk14 | CD8 | 3 cytokines | 0 |
| R379 | Nef | G3 DCIR MVA | Wk24 | CD4 | 3 cytokines | 0.0138 |
| R379 | Nef | G3 DCIR MVA | Wk24 | CD8 | 3 cytokines | 0.00722 |

| | | | | | | |
|---|---|---|---|---|---|---|
| R379 | Pol | G3 DCIR MVA | Wk14 | CD4 | 3 cytokines | 0.07419 |
| R379 | Pol | G3 DCIR MVA | Wk14 | CD8 | 3 cytokines | 0.00356 |
| R379 | Pol | G3 DCIR MVA | Wk24 | CD4 | 3 cytokines | 0.159 |
| R379 | Pol | G3 DCIR MVA | Wk24 | CD8 | 3 cytokines | 0.00755 |
| R380 | Gag p17 | G2 MVA CD40 | Wk10 | CD4 | 3 cytokines | 0.00933 |
| R380 | Gag p17 | G2 MVA CD40 | Wk10 | CD8 | 3 cytokines | 0 |
| R380 | Gag p17 | G2 MVA CD40 | Wk26 | CD4 | 3 cytokines | 0.00274 |
| R380 | Gag p17 | G2 MVA CD40 | Wk26 | CD8 | 3 cytokines | 0 |
| R380 | Gag p24 | G2 MVA CD40 | Wk10 | CD4 | 3 cytokines | 0.00355 |
| R380 | Gag p24 | G2 MVA CD40 | Wk10 | CD8 | 3 cytokines | 0.00315 |
| R380 | Gag p24 | G2 MVA CD40 | Wk26 | CD4 | 3 cytokines | 0.16389 |
| R380 | Gag p24 | G2 MVA CD40 | Wk26 | CD8 | 3 cytokines | 0.00233 |
| R380 | Nef | G2 MVA CD40 | Wk10 | CD4 | 3 cytokines | 0.00271 |
| R380 | Nef | G2 MVA CD40 | Wk10 | CD8 | 3 cytokines | 0.0026 |
| R380 | Nef | G2 MVA CD40 | Wk26 | CD4 | 3 cytokines | 0.06739 |
| R380 | Nef | G2 MVA CD40 | Wk26 | CD8 | 3 cytokines | 0.00118 |
| R380 | Pol | G2 MVA CD40 | Wk10 | CD4 | 3 cytokines | 0 |
| R380 | Pol | G2 MVA CD40 | Wk10 | CD8 | 3 cytokines | 0 |
| R380 | Pol | G2 MVA CD40 | Wk26 | CD4 | 3 cytokines | 0.01669 |
| R380 | Pol | G2 MVA CD40 | Wk26 | CD8 | 3 cytokines | 0.00116 |
| R381 | Gag p17 | G3 DCIR MVA | Wk14 | CD4 | 3 cytokines | 0 |
| R381 | Gag p17 | G3 DCIR MVA | Wk14 | CD8 | 3 cytokines | 0 |
| R381 | Gag p17 | G3 DCIR MVA | Wk24 | CD4 | 3 cytokines | 0.00267 |
| R381 | Gag p17 | G3 DCIR MVA | Wk24 | CD8 | 3 cytokines | 0.0017 |
| R381 | Gag p24 | G3 DCIR MVA | Wk14 | CD4 | 3 cytokines | 0.00223 |
| R381 | Gag p24 | G3 DCIR MVA | Wk14 | CD8 | 3 cytokines | 0 |
| R381 | Gag p24 | G3 DCIR MVA | Wk24 | CD4 | 3 cytokines | 0.0056 |
| R381 | Gag p24 | G3 DCIR MVA | Wk24 | CD8 | 3 cytokines | 0,0008 |
| R381 | Nef | G3 DCIR MVA | Wk14 | CD4 | 3 cytokines | 0.00077 |
| R381 | Nef | G3 DCIR MVA | Wk14 | CD8 | 3 cytokines | 0 |
| R381 | Pol | G3 DCIR MVA | Wk14 | CD4 | 3 cytokines | 0.0843 |
| R381 | Pol | G3 DCIR MVA | Wk14 | CD8 | 3 cytokines | 0 |
| R381 | Pol | G3 DCIR MVA | Wk24 | CD4 | 3 cytokines | 0.04572 |
| R381 | Pol | G3 DCIR MVA | Wk24 | CD8 | 3 cytokines | 0 |
| R382 | Gag p17 | G2 MVA CD40 | Wk10 | CD4 | 3 cytokines | 0.00101 |
| R382 | Gag p17 | G2 MVA CD40 | Wk10 | CD8 | 3 cytokines | 0 |
| R382 | Gag p17 | G2 MVA CD40 | Wk26 | CD4 | 3 cytokines | 0.00247 |
| R382 | Gag p17 | G2 MVA CD40 | Wk26 | CD8 | 3 cytokines | 0,00006 |
| R382 | Gag p24 | G2 MVA CD40 | Wk10 | CD4 | 3 cytokines | 0.00198 |
| R382 | Gag p24 | G2 MVA CD40 | Wk10 | CD8 | 3 cytokines | 0.00526 |
| R382 | Gag p24 | G2 MVA CD40 | Wk26 | CD4 | 3 cytokines | 0.09792 |
| R382 | Gag p24 | G2 MVA CD40 | Wk26 | CD8 | 3 cytokines | 0.00048 |
| R382 | Nef | G2 MVA CD40 | Wk10 | CD4 | 3 cytokines | 0.00913 |
| R382 | Nef | G2 MVA CD40 | Wk10 | CD8 | 3 cytokines | 0.01059 |
| R382 | Nef | G2 MVA CD40 | Wk26 | CD4 | 3 cytokines | 0.10592 |
| R382 | Nef | G2 MVA CD40 | Wk26 | CD8 | 3 cytokines | 0.00256 |
| R382 | Pol | G2 MVA CD40 | Wk10 | CD4 | 3 cytokines | 0.00953 |
| R382 | Pol | G2 MVA CD40 | Wk10 | CD8 | 3 cytokines | 0.01601 |
| R382 | Pol | G2 MVA CD40 | Wk26 | CD4 | 3 cytokines | 0.20192 |
| R382 | Pol | G2 MVA CD40 | Wk26 | CD8 | 3 cytokines | 0.01093 |
| R383 | Gag p17 | G4 CD40 MVA | Wk14 | CD4 | 3 cytokines | 0.0028 |
| R383 | Gag p17 | G4 CD40 MVA | Wk14 | CD8 | 3 cytokines | 0.00453 |
| R383 | Gag p17 | G4 CD40 MVA | Wk24 | CD4 | 3 cytokines | 0.008 |
| R383 | Gag p17 | G4 CD40 MVA | Wk24 | CD8 | 3 cytokines | 0.00115 |
| R383 | Gag p24 | G4 CD40 MVA | Wk14 | CD4 | 3 cytokines | 0.0155 |
| R383 | Gag p24 | G4 CD40 MVA | Wk14 | CD8 | 3 cytokines | 0.00116 |
| R383 | Gag p24 | G4 CD40 MVA | Wk24 | CD4 | 3 cytokines | 0.0615 |
| R383 | Gag p24 | G4 CD40 MVA | Wk24 | CD8 | 3 cytokines | 0.01912 |

| | | | | | | |
|---|---|---|---|---|---|---|
| R383 | Nef | G4 CD40 MVA | Wk14 | CD4 | 3 cytokines | 0.0309 |
| R383 | Nef | G4 CD40 MVA | Wk14 | CD8 | 3 cytokines | 0.00111 |
| R383 | Nef | G4 CD40 MVA | Wk24 | CD4 | 3 cytokines | 0.0894 |
| R383 | Nef | G4 CD40 MVA | Wk24 | CD8 | 3 cytokines | 0.0065 |
| R383 | Pol | G4 CD40 MVA | Wk14 | CD4 | 3 cytokines | 0.0458 |
| R383 | Pol | G4 CD40 MVA | Wk14 | CD8 | 3 cytokines | 0 |
| R383 | Pol | G4 CD40 MVA | Wk24 | CD4 | 3 cytokines | 0.1884 |
| R383 | Pol | G4 CD40 MVA | Wk24 | CD8 | 3 cytokines | 0.00552 |
| R384 | Gag p17 | G2 MVA CD40 | Wk26 | CD4 | 3 cytokines | 0.00465 |
| R384 | Gag p17 | G2 MVA CD40 | Wk26 | CD8 | 3 cytokines | 0.00134 |
| R384 | Gag p24 | G2 MVA CD40 | Wk10 | CD4 | 3 cytokines | 0.00019 |
| R384 | Gag p24 | G2 MVA CD40 | Wk10 | CD8 | 3 cytokines | 0.00123 |
| R384 | Gag p24 | G2 MVA CD40 | Wk26 | CD4 | 3 cytokines | 0.17362 |
| R384 | Gag p24 | G2 MVA CD40 | Wk26 | CD8 | 3 cytokines | 0.00689 |
| R384 | Nef | G2 MVA CD40 | Wk10 | CD4 | 3 cytokines | 0.00025 |
| R384 | Nef | G2 MVA CD40 | Wk10 | CD8 | 3 cytokines | 0.00193 |
| R384 | Nef | G2 MVA CD40 | Wk26 | CD4 | 3 cytokines | 0.10562 |
| R384 | Nef | G2 MVA CD40 | Wk26 | CD8 | 3 cytokines | 0.0049 |
| R384 | Pol | G2 MVA CD40 | Wk10 | CD4 | 3 cytokines | 0 |
| R384 | Pol | G2 MVA CD40 | Wk10 | CD8 | 3 cytokines | 0.0013 |
| R384 | Pol | G2 MVA CD40 | Wk26 | CD4 | 3 cytokines | 0.07782 |
| R384 | Pol | G2 MVA CD40 | Wk26 | CD8 | 3 cytokines | 0.00545 |
| R385 | Gag p17 | G1 MVA DCIR | Wk10 | CD4 | 3 cytokines | 0.00199 |
| R385 | Gag p17 | G1 MVA DCIR | Wk10 | CD8 | 3 cytokines | 0.00166 |
| R385 | Gag p17 | G1 MVA DCIR | Wk26 | CD4 | 3 cytokines | 0.00594 |
| R385 | Gag p17 | G1 MVA DCIR | Wk26 | CD8 | 3 cytokines | 0.00152 |
| R385 | Gag p24 | G1 MVA DCIR | Wk10 | CD4 | 3 cytokines | 0 |
| R385 | Gag p24 | G1 MVA DCIR | Wk10 | CD8 | 3 cytokines | 0.00527 |
| R385 | Gag p24 | G1 MVA DCIR | Wk26 | CD4 | 3 cytokines | 0.03104 |
| R385 | Gag p24 | G1 MVA DCIR | Wk26 | CD8 | 3 cytokines | 0.00523 |
| R385 | Nef | G1 MVA DCIR | Wk10 | CD4 | 3 cytokines | 0.00551 |
| R385 | Nef | G1 MVA DCIR | Wk10 | CD8 | 3 cytokines | 0.0016 |
| R385 | Nef | G1 MVA DCIR | Wk26 | CD4 | 3 cytokines | 0.02744 |
| R385 | Nef | G1 MVA DCIR | Wk26 | CD8 | 3 cytokines | 0 |
| R385 | Pol | G1 MVA DCIR | Wk10 | CD4 | 3 cytokines | 0.00316 |
| R385 | Pol | G1 MVA DCIR | Wk10 | CD8 | 3 cytokines | 0 |
| R385 | Pol | G1 MVA DCIR | Wk26 | CD4 | 3 cytokines | 0.06914 |
| R385 | Pol | G1 MVA DCIR | Wk26 | CD8 | 3 cytokines | 0.00088 |
| R386 | Gag p17 | G4 CD40 MVA | Wk14 | CD4 | 3 cytokines | 0.00457 |
| R386 | Gag p17 | G4 CD40 MVA | Wk14 | CD8 | 3 cytokines | 0.0157 |
| R386 | Gag p17 | G4 CD40 MVA | Wk24 | CD4 | 3 cytokines | 0.00132 |
| R386 | Gag p17 | G4 CD40 MVA | Wk24 | CD8 | 3 cytokines | 0.01402 |
| R386 | Gag p24 | G4 CD40 MVA | Wk14 | CD4 | 3 cytokines | 0.00905 |
| R386 | Gag p24 | G4 CD40 MVA | Wk14 | CD8 | 3 cytokines | 0 |
| R386 | Gag p24 | G4 CD40 MVA | Wk24 | CD4 | 3 cytokines | 0.01254 |
| R386 | Gag p24 | G4 CD40 MVA | Wk24 | CD8 | 3 cytokines | 0.00183 |
| R386 | Nef | G4 CD40 MVA | Wk14 | CD4 | 3 cytokines | 0.00189 |
| R386 | Nef | G4 CD40 MVA | Wk14 | CD8 | 3 cytokines | 0.00077 |
| R386 | Nef | G4 CD40 MVA | Wk24 | CD4 | 3 cytokines | 0.01084 |
| R386 | Nef | G4 CD40 MVA | Wk24 | CD8 | 3 cytokines | 0 |
| R386 | Pol | G4 CD40 MVA | Wk14 | CD4 | 3 cytokines | 0.0258 |
| R386 | Pol | G4 CD40 MVA | Wk14 | CD8 | 3 cytokines | 0.00079 |
| R386 | Pol | G4 CD40 MVA | Wk24 | CD4 | 3 cytokines | 0.02074 |
| R386 | Pol | G4 CD40 MVA | Wk24 | CD8 | 3 cytokines | 0 |
| R388 | Gag p17 | G3 DCIR MVA | Wk24 | CD4 | 3 cytokines | 0.0127 |
| R388 | Gag p17 | G3 DCIR MVA | Wk24 | CD8 | 3 cytokines | 0 |
| R388 | Gag p24 | G3 DCIR MVA | Wk24 | CD4 | 3 cytokines | 0.019 |
| R388 | Gag p24 | G3 DCIR MVA | Wk24 | CD8 | 3 cytokines | 0.0023 |

| | | | | | | |
|---|---|---|---|---|---|---|
| R389 | Gag p17 | G4 CD40 MVA | Wk24 | CD4 | 3 cytokines | 0.00755 |
| R389 | Gag p17 | G4 CD40 MVA | Wk24 | CD8 | 3 cytokines | 0.0144 |
| R389 | Gag p24 | G4 CD40 MVA | Wk24 | CD4 | 3 cytokines | 0.03645 |
| R389 | Gag p24 | G4 CD40 MVA | Wk24 | CD8 | 3 cytokines | 0.00193 |
| R389 | Pol | G4 CD40 MVA | Wk24 | CD4 | 3 cytokines | 0.16565 |
| R389 | Pol | G4 CD40 MVA | Wk24 | CD8 | 3 cytokines | 0 |
| R390 | Gag p17 | G4 CD40 MVA | Wk14 | CD4 | 3 cytokines | 0.00103 |
| R390 | Gag p17 | G4 CD40 MVA | Wk14 | CD8 | 3 cytokines | 0 |
| R390 | Gag p17 | G4 CD40 MVA | Wk24 | CD4 | 3 cytokines | 0.00255 |
| R390 | Gag p17 | G4 CD40 MVA | Wk24 | CD8 | 3 cytokines | 0.00141 |
| R390 | Gag p24 | G4 CD40 MVA | Wk14 | CD4 | 3 cytokines | 0.00256 |
| R390 | Gag p24 | G4 CD40 MVA | Wk14 | CD8 | 3 cytokines | 0 |
| R390 | Gag p24 | G4 CD40 MVA | Wk24 | CD4 | 3 cytokines | 0.00909 |
| R390 | Gag p24 | G4 CD40 MVA | Wk24 | CD8 | 3 cytokines | 0.00517 |
| R390 | Nef | G4 CD40 MVA | Wk14 | CD4 | 3 cytokines | 0.00197 |
| R390 | Nef | G4 CD40 MVA | Wk14 | CD8 | 3 cytokines | 0 |
| R390 | Nef | G4 CD40 MVA | Wk24 | CD4 | 3 cytokines | 0.00757 |
| R390 | Nef | G4 CD40 MVA | Wk24 | CD8 | 3 cytokines | 0 |
| R390 | Pol | G4 CD40 MVA | Wk14 | CD4 | 3 cytokines | 0.0032 |
| R390 | Pol | G4 CD40 MVA | Wk14 | CD8 | 3 cytokines | 0 |
| R390 | Pol | G4 CD40 MVA | Wk24 | CD4 | 3 cytokines | 0.02135 |
| R390 | Pol | G4 CD40 MVA | Wk24 | CD8 | 3 cytokines | 0.00099 |
| R391 | Gag p17 | G4 CD40 MVA | Wk14 | CD4 | 3 cytokines | 0.00102 |
| R391 | Gag p17 | G4 CD40 MVA | Wk14 | CD8 | 3 cytokines | 0.00312 |
| R391 | Gag p17 | G4 CD40 MVA | Wk24 | CD4 | 3 cytokines | 0.0051 |
| R391 | Gag p17 | G4 CD40 MVA | Wk24 | CD8 | 3 cytokines | 0 |
| R391 | Gag p24 | G4 CD40 MVA | Wk14 | CD4 | 3 cytokines | 0.01861 |
| R391 | Gag p24 | G4 CD40 MVA | Wk14 | CD8 | 3 cytokines | 0.00166 |
| R391 | Gag p24 | G4 CD40 MVA | Wk24 | CD4 | 3 cytokines | 0.0326 |
| R391 | Gag p24 | G4 CD40 MVA | Wk24 | CD8 | 3 cytokines | 0.00971 |
