# Supplementary material for: HIV-1 T cell epitopes targeted to Rhesus macaque CD40 and DCIR: A comparative study of prototype dendritic cell targeting therapeutic vaccine candidates
Source: PLoS One. 2018 Nov 30;13(11):e0207794. doi: 10.1371/journal.pone.0207794 (PMC6267996; doi:10.1371/journal.pone.0207794)
Supplement: S5 Table — This table is the data that relates to Fig 6. Animal numbers, group, and sample time in weeks are identified. D 0 is sample just prior to study initiation. The 1/EC50 values were calculated as indicated in the Methods section. (PDF) [file pone.0207794.s009.pdf]

S5 Table. Serum HIV5pep-specific IgG responses elicited by αDCIR.HIV5pep or αCD40.HIV5pep vaccines. This table is the data that relates to Fig 6. Animal numbers, group, and sample time in weeks are identified. D 0 is sample just prior to study initiation. The 1/EC_{50} values were calculated as indicated in the Methods section.

		NHP 1	NHP 2	NHP 3	NHP 4	NHP 5	NHP 6
**D 0 (MVA #1)**	Group 1	6.7		7.0	19.1	11.9	8.5
**wk 2**	Group 1	17.5	39.3	3.5	11.3	24.0	5.8
**wk 8 (MVA #2)**	Group 1	4.7	15.1	4.5	3.9	14.7	14.2
**wk 10**	Group 1	15.3	20.1	4.4	3.9	7.3	5.6
**wk 12 (Vaccine #1) sera**	Group 1	29.2	13.1	2.6	9.3	7.0	7.2
**wk 12 (Vaccine #1) plasma**	Group 1	9.6	18.2	6.0	9.0	9.7	3.1
**wk 14**	Group 1	192.5	206.7	331.1	56.9	262.1	187.7
**wk 16 (Vaccine #2)**	Group 1	120.9	139.5	230.8	38.6	119.0	111.0
**wk 18**	Group 1	2030.5	1802.8	5592.8	1100.8	4315.9	2272.7
**wk 24 (Vaccine # 3)**	Group 1	184.2	112.8	297.7	60.6	332.3	254.3
**wk 26**	Group 1	739.6	883.4	5083.9	925.9	1995.2	2582.6
**wk 28**	Group 1	592.4	547.0	2862.9	463.0	1384.1	1149.6

**D 0 (MVA #1)**	Group 1	16.5	25.1	9.7	16.2	11.6	3.4
**wk 2**	Group 1	16.1	13.7	14.3	5.5	6.8	3.1
**wk 8 (MVA #2)**	Group 1	20.6	16.1	6.0	7.4	0.3	1.1
**wk 10**	Group 1	7.3	8.1	10.2	8.2	10.2	1.2
**wk 12 (Vaccine #1) sera**	Group 1	16.5	2.6	12.9	1.1	0.5	4.5
**wk 12 (Vaccine #1) plasma**	Group 1	14.5	13.6	12.2	10.1	1.0	0.3
**wk 14**	Group 1	284.6	168.1	299.1	332.2	120.9	23.7
**wk 16 (Vaccine #2)**	Group 1	127.2	119.8	170.7	146.7	61.1	32.3
**wk 18**	Group 1	3586.8	7917.7	3855.1	2898.6	858.4	2478.3
**wk 24 (Vaccine # 3)**	Group 1	1092.5	1621.5	1123.3	620.0	115.5	232.1
**wk 26**	Group 1	2560.8	4393.7	5277.0	3150.6	1487.7	2351.3
**wk 28**	Group 1	2138.1	3079.8	2791.0	1545.8	558.7	657.5
**D 0 (Vaccine #1)**	Group 3	1.8	3.9	9.1	0.0	5.1	39.8
**wk 2**	Group 3	318.3	265.4	506.3	280.9	390.0	234.9
**wk 4 (Vaccine #2)**	Group 3	324.8	227.8	354.1	258.5	94.0	334.7
**wk 6**	Group 3	2191.5	8944.5	9689.9	3139.7	2457.0	3096.9
**wk 12 (Vaccine #3)**	Group 3	257.9	502.5	661.8	398.2		483.3
**wk 14**	Group 3	936.3	2739.7	2828.1	1320.8		1649.3
**wk 22 (MVA)**	Group 3	164.2	262.2	305.6	191.8		147.1
**wk 24**	Group 3	71.7	250.3	404.2	192.6		372.3
**wk 28**	Group 3	168.5	210.7	370.1	148.2		390.2
**D 0 (Vaccine #1)**	Group 4	19.6	18.0	172.5	2.8	28.0	9.8
**wk 2**	Group 4	108.5	276.9	1008.1	350.8	138.0	75.5
**wk 4 (Vaccine #2)**	Group 4	112.9	137.9	823.7	239.0	108.0	79.9
**wk 6**	Group 4	2708.6	5934.7	14556.0	9009.0	2527.8	1541.8
**wk 12 (Vaccine #3)**	Group 4	122.2	163.0	674.8	1027.1	139.4	165.0
**wk 14**	Group 4	628.1	1599.7	1574.1	1950.5	524.7	505.1
**wk 22 (MVA)**	Group 4	167.9	520.6	1376.7	1509.0	212.5	200.5
**wk 24**	Group 4	268.6	454.1	1793.7	1410.4	241.0	296.7
**wk 28**	Group 4	368.7	383.4	1081.0	1001.6	228.7	284.9
