# Supplementary material for: HIV-1 T cell epitopes targeted to Rhesus macaque CD40 and DCIR: A comparative study of prototype dendritic cell targeting therapeutic vaccine candidates
Source: PLoS One. 2018 Nov 30;13(11):e0207794. doi: 10.1371/journal.pone.0207794 (PMC6267996; doi:10.1371/journal.pone.0207794)
Supplement: S6 Table — These are the primary readings for titration of the sera corresponding to the indicated samples for each of the six animals in each group. The dilution series from top to bottom were, respectively, 1/75, 1/225, 1/675, 1/2024, 1/6060, 1/18214, 1/54644, and 1/163944. These data are plotted as average values in S3 Fig. (PDF) [file pone.0207794.s010.pdf]

S6 Table. Sample titration curves used for the calculation of the serum antibody response presented in Fig 6. These are the primary readings for titration of the sera corresponding to the indicated samples for each of the six animals in each group. The dilution series from top to bottom were, respectively, 1/75, 1/225, 1/675, 1/2024, 1/6060, 1/18214, 1/54644, and 1/163944. These data are plotted as average values in S3 Fig.

NHP 1	NHP 2	NHP 3	NHP 4	NHP 5	NHP6	Sample
0.1411	0.113	0.0974	0.1603	0.2202	0.0991	G1 D0
0.0762	0.0732	0.0583	0.0616	0.0918	0.0574	G1 D0
0.0541	0.0545	0.0536	0.0539	0.0675	0.0535	G1 D0
0.0483	0.0494	0.0475	0.0491	0.0515	0.0477	G1 D0
0.0471	0.0477	0.0477	0.0488	0.0491	0.0487	G1 D0
0.0465	0.0458	0.0604	0.0476	0.0487	0.0472	G1 D0
0.0484	0.0801	0.0462	0.0477	0.0511	0.0509	G1 D0
0.047	0.0577	0.0513	0.0454	0.0483	0.0485	G1 D0
0.2543	0.6759	0.0871	0.148	0.3075	0.0909	G1 wk 2 post MVA prime 1
0.0914	0.2655	0.0609	0.0688	0.0947	0.0587	G1 wk 2 post MVA prime 1
0.0628	0.1306	0.051	0.0535	0.0647	0.0508	G1 wk 2 post MVA prime 1
0.0592	0.0726	0.0451	0.0474	0.0506	0.0472	G1 wk 2 post MVA prime 1
0.0503	0.0576	0.0489	0.0502	0.0496	0.049	G1 wk 2 post MVA prime 1
0.0492	0.0525	0.0479	0.0497	0.0495	0.0489	G1 wk 2 post MVA prime 1
0.0506	0.0488	0.0464	0.0477	0.0504	0.0459	G1 wk 2 post MVA prime 1
0.0472	0.0458	0.0462	0.048	0.046	0.0453	G1 wk 2 post MVA prime 1

0.1439	0.2658	0.1018	0.1157	0.2574	0.1393	G1 wk 8 (MVA prime 2 inj)
0.0795	0.1049	0.0628	0.0698	0.1016	0.0633	G1 wk 8 (MVA prime 2 inj)
0.0601	0.0658	0.0540	0.0556	0.0671	0.0494	G1 wk 8 (MVA prime 2 inj)
0.0519	0.0559	0.0495	0.0500	0.0530	0.0484	G1 wk 8 (MVA prime 2 inj)
0.0503	0.0499	0.0497	0.0510	0.0503	0.0490	G1 wk 8 (MVA prime 2 inj)
0.0484	0.0496	0.0498	0.0486	0.0512	0.0449	G1 wk 8 (MVA prime 2 inj)
0.0492	0.0494	0.0494	0.0502	0.0486	0.0463	G1 wk 8 (MVA prime 2 inj)
0.0510	0.0492	0.0485	0.0502	0.0482	0.0487	G1 wk 8 (MVA prime 2 inj)
0.2688	0.2816	0.0989	0.1229	0.2204	0.0956	G1 wk 10 (2 wk post MVA prime 2)
0.1069	0.0959	0.0625	0.0725	0.0900	0.0597	G1 wk 10 (2 wk post MVA prime 2)
0.0646	0.0649	0.0532	0.0572	0.0983	0.0523	G1 wk 10 (2 wk post MVA prime 2)
0.0532	0.0506	0.0479	0.0515	0.0529	0.0486	G1 wk 10 (2 wk post MVA prime 2)
0.0527	0.0534	0.0490	0.0482	0.0526	0.0505	G1 wk 10 (2 wk post MVA prime 2)
0.0469	0.0510	0.0485	0.0490	0.0481	0.0482	G1 wk 10 (2 wk post MVA prime 2)
0.0496	0.0494	0.0492	0.0480	0.0468	0.0480	G1 wk 10 (2 wk post MVA prime 2)
0.0486	0.0484	0.0459	0.0462	0.0467	0.0489	G1 wk 10 (2 wk post MVA prime 2)
0.3256	0.2175	0.0856	0.1346	0.1470	0.1009	G1 wk 12 vaccine inj plasma
0.0865	0.0900	0.0599	0.0667	0.0720	0.0595	G1 wk 12 vaccine inj plasma
0.0627	0.0612	0.0545	0.0554	0.0630	0.0525	G1 wk 12 vaccine inj plasma
0.0517	0.0506	0.0479	0.0492	0.0530	0.0483	G1 wk 12 vaccine inj plasma
0.0502	0.0480	0.0476	0.0479	0.0503	0.0507	G1 wk 12 vaccine inj plasma
0.0483	0.0479	0.0466	0.0491	0.0491	0.0466	G1 wk 12 vaccine inj plasma
0.0497	0.0473	0.0471	0.0479	0.0492	0.0499	G1 wk 12 vaccine inj plasma
0.0486	0.0469	0.0469	0.0489	0.0459	0.0462	G1 wk 12 vaccine inj plasma

0.2090	0.2759	0.0904	0.1372	0.1890	0.0903	G1 wk 12 vaccine inj sera
0.0945	0.0996	0.0577	0.0689	0.0842	0.0598	G1 wk 12 vaccine inj sera
0.0659	0.0649	0.0513	0.0535	0.0651	0.0557	G1 wk 12 vaccine inj sera
0.0540	0.0518	0.0512	0.0492	0.0508	0.0485	G1 wk 12 vaccine inj sera
0.0513	0.0473	0.0488	0.0484	0.0488	0.0497	G1 wk 12 vaccine inj sera
0.0476	0.0487	0.0468	0.0471	0.0500	0.0478	G1 wk 12 vaccine inj sera
0.0508	0.0491	0.0479	0.0481	0.0555	0.0476	G1 wk 12 vaccine inj sera
0.0486	0.0466	0.0449	0.0431	0.0442	0.0482	G1 wk 12 vaccine inj sera
1.5275	1.4430	1.5973	0.8791	1.5111	1.3735	G1 wk14 (2wk post vaccine boost)
0.9334	1.0219	1.2145	0.4185	1.1424	0.9766	G1 wk14 (2wk post vaccine boost)
0.4010	0.5170	0.7262	0.1720	0.6078	0.5308	G1 wk14 (2wk post vaccine boost)
0.1544	0.1948	0.3264	0.0870	0.2553	0.2464	G1 wk14 (2wk post vaccine boost)
0.0844	0.0906	0.1355	0.0585	0.1097	0.1080	G1 wk14 (2wk post vaccine boost)
0.0579	0.0598	0.0733	0.0530	0.0636	0.0651	G1 wk14 (2wk post vaccine boost)
0.0525	0.0547	0.0561	0.0516	0.0528	0.0554	G1 wk14 (2wk post vaccine boost)
0.0532	0.0492	0.0552	0.0497	0.0491	0.0521	G1 wk14 (2wk post vaccine boost)
1.2559	1.3611	1.5030	0.6572	1.2660	1.2298	G1 wk 16 (4wk post vaccine boost)
0.7392	0.7624	1.0448	0.2594	0.6995	0.6652	G1 wk 16 (4wk post vaccine boost)
0.2906	0.3191	0.5703	0.1153	0.2966	0.2964	G1 wk 16 (4wk post vaccine boost)
0.1344	0.1362	0.2339	0.0666	0.1240	0.1282	G1 wk 16 (4wk post vaccine boost)
0.0753	0.0752	0.0972	0.0540	0.0674	0.0673	G1 wk 16 (4wk post vaccine boost)
0.0581	0.0571	0.0639	0.0504	0.0531	0.0528	G1 wk 16 (4wk post vaccine boost)
0.0529	0.0525	0.0558	0.0484	0.0512	0.0503	G1 wk 16 (4wk post vaccine boost)
0.0494	0.0492	0.0489	0.0442	0.0467	0.0475	G1 wk 16 (4wk post vaccine boost)

0.2314	0.3941	0.1802	0.2899	0.1949	0.0828	G2 D0
0.0870	0.1349	0.0851	0.1141	0.0823	0.0575	G2 D0
0.0588	0.0701	0.0562	0.0675	0.0630	0.0533	G2 D0
0.0504	0.0536	0.0494	0.0542	0.0522	0.0490	G2 D0
0.0494	0.0503	0.0483	0.0492	0.0514	0.0489	G2 D0
0.0477	0.0481	0.0472	0.0490	0.0500	0.0478	G2 D0
0.0487	0.0480	0.0481	0.0560	0.0477	0.0480	G2 D0
0.0489	0.0470	0.0462	0.0485	0.0468	0.0464	G2 D0
0.2445	0.4028	0.2206	0.1587	0.1256	0.1009	G2 wk 2 post MVA prime 1
0.0933	0.2525	0.0887	0.0889	0.0690	0.0627	G2 wk 2 post MVA prime 1
0.0606	0.0785	0.0582	0.0559	0.0529	0.0591	G2 wk 2 post MVA prime 1
0.0522	0.0562	0.0523	0.0505	0.0487	0.0496	G2 wk 2 post MVA prime 1
0.0502	0.0558	0.0507	0.0491	0.0489	0.0490	G2 wk 2 post MVA prime 1
0.0492	0.0489	0.0476	0.0491	0.0475	0.0484	G2 wk 2 post MVA prime 1
0.0485	0.0476	0.0485	0.0474	0.0465	0.0480	G2 wk 2 post MVA prime 1
0.0483	0.0475	0.0457	0.0467	0.0473	0.0476	G2 wk 2 post MVA prime 1
0.3636	0.2265	0.1369	0.1933	0.0984	0.0752	G2 wk 8 (MVA prime 2 inj)
0.1374	0.0859	0.0742	0.0973	0.0880	0.0561	G2 wk 8 (MVA prime 2 inj)
0.0728	0.0589	0.0557	0.0616	0.0586	0.0554	G2 wk 8 (MVA prime 2 inj)
0.0539	0.0523	0.0522	0.0543	0.0510	0.0507	G2 wk 8 (MVA prime 2 inj)
0.0512	0.0497	0.0500	0.0502	0.0472	0.0506	G2 wk 8 (MVA prime 2 inj)
0.0489	0.0491	0.0441	0.0492	0.0460	0.0526	G2 wk 8 (MVA prime 2 inj)
0.0493	0.0480	0.0505	0.0484	0.0488	0.0507	G2 wk 8 (MVA prime 2 inj)
0.0493	0.0487	0.0473	0.0514	0.0502	0.0496	G2 wk 8 (MVA prime 2 inj)

0.2652	0.2196	0.1687	0.2008	0.1150	0.0858	G2 wk 10 (2 wk post MVA prime 2)
0.1431	0.1088	0.0766	0.0974	0.0607	0.0570	G2 wk 10 (2 wk post MVA prime 2)
0.0809	0.0671	0.0584	0.0640	0.0516	0.0623	G2 wk 10 (2 wk post MVA prime 2)
0.0566	0.0521	0.0505	0.0531	0.0480	0.0513	G2 wk 10 (2 wk post MVA prime 2)
0.0534	0.0516	0.0507	0.0480	0.0483	0.0490	G2 wk 10 (2 wk post MVA prime 2)
0.0490	0.0496	0.0515	0.0508	0.0490	0.0497	G2 wk 10 (2 wk post MVA prime 2)
0.0499	0.0486	0.0498	0.0482	0.0499	0.0484	G2 wk 10 (2 wk post MVA prime 2)
0.0489	0.0517	0.0487	0.0481	0.0469	0.0484	G2 wk 10 (2 wk post MVA prime 2)
0.3153	0.1638	0.1254	0.1271	0.1022	0.0959	G2 wk 12 vaccine inj plasma
0.1259	0.0969	0.0577	0.0895	0.0767	0.0601	G2 wk 12 vaccine inj plasma
0.0706	0.0693	0.0608	0.0600	0.0598	0.0533	G2 wk 12 vaccine inj plasma
0.0587	0.0588	0.0537	0.0582	0.0533	0.0532	G2 wk 12 vaccine inj plasma
0.0540	0.0529	0.0501	0.0518	0.0518	0.0498	G2 wk 12 vaccine inj plasma
0.0518	0.0509	0.0497	0.0504	0.0508	0.0503	G2 wk 12 vaccine inj plasma
0.0530	0.0513	0.0496	0.0524	0.0557	0.0518	G2 wk 12 vaccine inj plasma
0.0507	0.0520	0.0510	0.0534	0.0544	0.0477	G2 wk 12 vaccine inj plasma
0.3513	0.2820	0.1943	0.2260	0.0963	0.0846	G2 wk 12 vaccine inj sera
0.1614	0.1183	0.0811	0.1019	0.0667	0.0651	G2 wk 12 vaccine inj sera
0.0828	0.0719	0.0601	0.0666	0.0574	0.0555	G2 wk 12 vaccine inj sera
0.0594	0.0550	0.0540	0.0530	0.0530	0.0539	G2 wk 12 vaccine inj sera
0.0555	0.0537	0.0588	0.0556	0.0535	0.0537	G2 wk 12 vaccine inj sera
0.0540	0.0528	0.0570	0.0523	0.0518	0.0512	G2 wk 12 vaccine inj sera
0.0516	0.0558	0.0509	0.0503	0.0532	0.0544	G2 wk 12 vaccine inj sera
0.0513	0.0532	0.0507	0.0523	0.0549	0.0541	G2 wk 12 vaccine inj sera

1.4887	1.3239	1.4842	1.5810	1.2514	0.6199	G2 wk14 (2wk post vaccine boost)
1.1521	0.9358	1.1970	1.2173	0.7310	0.5042	G2 wk14 (2wk post vaccine boost)
0.7105	0.5066	0.7147	0.7272	0.3570	0.2058	G2 wk14 (2wk post vaccine boost)
0.3258	0.2284	0.3440	0.3522	0.1545	0.0971	G2 wk14 (2wk post vaccine boost)
0.1344	0.1063	0.1426	0.1470	0.0737	0.0645	G2 wk14 (2wk post vaccine boost)
0.0717	0.0608	0.0698	0.0775	0.0551	0.0543	G2 wk14 (2wk post vaccine boost)
0.0574	0.0527	0.0556	0.0576	0.0507	0.0506	G2 wk14 (2wk post vaccine boost)
0.0602	0.0499	0.0499	0.0509	0.0498	0.0625	G2 wk14 (2wk post vaccine boost)
1.2029	1.2271	1.3883	1.3567	0.9110	0.6543	G2 wk 16 (4wk post vaccine boost)
0.8549	0.7704	0.9073	0.8127	0.4660	0.3494	G2 wk 16 (4wk post vaccine boost)
0.3983	0.3096	0.4514	0.3659	0.1784	0.1414	G2 wk 16 (4wk post vaccine boost)
0.1649	0.1324	0.1930	0.1371	0.0791	0.0767	G2 wk 16 (4wk post vaccine boost)
0.0785	0.0737	0.0923	0.0725	0.0570	0.0578	G2 wk 16 (4wk post vaccine boost)
0.0580	0.0570	0.0603	0.0605	0.0536	0.0659	G2 wk 16 (4wk post vaccine boost)
0.0555	0.0517	0.0547	0.0518	0.0516	0.0496	G2 wk 16 (4wk post vaccine boost)
0.0490	0.0496	0.0487	0.0471	0.0476	0.0485	G2 wk 16 (4wk post vaccine boost)
0.0868	0.1265	0.2642	0.0700	0.1770	0.6317	G3 D 0
0.0622	0.0760	0.1138	0.0675	0.0913	0.2148	G3 D 0
0.0540	0.0556	0.0978	0.0549	0.0704	0.1012	G3 D 0
0.0497	0.0509	0.0536	0.0511	0.0541	0.0636	G3 D 0
0.0498	.0492	0.0502	0.0496	0.0503	0.0532	G3 D 0
0.0480	0.0479	0.0472	0.0489	0.0494	0.0495	G3 D 0
0.0483	0.0489	0.0465	0.0492	0.0495	0.0509	G3 D 0
0.0486	0.0492	0.0489	0.0490	0.0515	0.0489	G3 D 0
1.4078	1.4259	1.4821	1.4802	1.5740	1.4775	G3 2 wk post vaccine
1.3010	1.1865	1.3806	1.1495	1.2524	1.0539	G3 2 wk post vaccine

0.7482	0.6564	1.0243	0.7279	0.8556	0.6002	G3 2 wk post vaccine
0.3429	0.2932	0.5373	0.2798	0.4176	0.2818	G3 2 wk post vaccine
0.1405	0.1139	0.2044	0.1248	0.1859	0.1163	G3 2 wk post vaccine
0.0803	0.0674	0.0905	0.0767	0.0831	0.0685	G3 2 wk post vaccine
0.0604	0.0541	0.0609	0.0556	0.0600	0.0562	G3 2 wk post vaccine
0.0535	0.0504	0.0522	0.0495	0.0500	0.0495	G3 2 wk post vaccine
1.6883	1.4328	1.6327	1.5153	1.1655	1.5919	G3 4 wk post vaccine
1.2044	1.0720	1.2535	1.0635	0.5364	1.2221	G3 4 wk post vaccine
0.6538	0.5910	0.7332	0.6462	0.2506	0.7155	G3 4 wk post vaccine
0.2924	0.2737	0.3366	0.3385	0.1140	0.3516	G3 4 wk post vaccine
0.1341	0.1200	0.1475	0.1781	0.0722	0.1726	G3 4 wk post vaccine
0.0754	0.0705	0.0724	0.1053	0.0553	0.0903	G3 4 wk post vaccine
0.0570	0.0546	0.0553	0.4828	0.0513	0.0661	G3 4 wk post vaccine
0.0522	0.0526	0.0504	0.0631	0.0519	0.0548	G3 4 wk post vaccine
1.6685	1.7737	1.7288	1.5673	1.5809	1.5530	G3 wk 6 (2 wk post 2nd boost)
1.7429	1.8112	1.8457	1.7132	1.7792	1.8967	G3 wk 6 (2 wk post 2nd boost)
1.5259	1.7908	1.7333	1.6470	1.4949	1.6027	G3 wk 6 (2 wk post 2nd boost)
1.0671	1.6048	1.5291	1.2192	1.1567	1.2010	G3 wk 6 (2 wk post 2nd boost)
0.6010	1.2114	1.1384	0.7688	0.6650	0.7300	G3 wk 6 (2 wk post 2nd boost)
0.2845	0.7279	0.7660	0.3673	0.2911	0.3730	G3 wk 6 (2 wk post 2nd boost)
0.1401	0.3811	0.5984	0.1884	0.1365	0.1879	G3 wk 6 (2 wk post 2nd boost)
0.0865	0.1683	0.3022	0.0943	0.0799	0.1099	G3 wk 6 (2 wk post 2nd boost)
1.6606	1.7075	1.7782	1.6602	0.0522	1.6853	G3 wk 12 (8 wk post second boost)
1.0812	1.4485	1.5411	1.3166	0.0515	1.3623	G3 wk 12 (8 wk post second boost)
0.5177	0.8706	1.0228	0.7680	0.0559	0.9023	G3 wk 12 (8 wk post second boost)
0.2324	0.4168	0.4960	0.3776	0.0516	0.4423	G3 wk 12 (8 wk post second boost)
0.1083	0.1888	0.2196	0.1687	0.0526	0.1882	G3 wk 12 (8 wk post second boost)
0.0729	0.0954	0.1084	0.0966	0.0503	0.0996	G3 wk 12 (8 wk post second boost)
0.0586	0.0674	0.2465	0.0656	0.0495	0.0692	G3 wk 12 (8 wk post second boost)
0.0526	0.0566	0.0559	0.0592	0.0512	0.0546	G3 wk 12 (8 wk post second boost)

1.8034	1.8273	1.6233	1.7313	0.0508	1.7529	G3 wk 14 (2 wk post 3rd boost)
1.6442	1.8094	1.7514	1.7062	0.0484	1.7359	G3 wk 14 (2 wk post 3rd boost)
1.2005	1.6160	1.6484	1.3801	0.0507	1.4153	G3 wk 14 (2 wk post 3rd boost)
0.6486	1.1853	1.2210	0.8196	0.0520	0.9608	G3 wk 14 (2 wk post 3rd boost)
0.3118	0.6692	0.6955	0.4156	0.0528	0.5087	G3 wk 14 (2 wk post 3rd boost)
0.1354	0.3032	0.3391	0.1804	0.0497	0.2195	G3 wk 14 (2 wk post 3rd boost)
0.0868	0.1606	0.1629	0.0969	0.0516	0.1110	G3 wk 14 (2 wk post 3rd boost)
0.0633	0.0930	0.1044	0.0714	0.0501	0.0773	G3 wk 14 (2 wk post 3rd boost)
0.2817	0.3698	1.4092	0.1514	0.4006	0.1711	G4 D 0
0.0976	0.1518	0.9188	0.0930	0.1186	0.0773	G4 D 0
0.0634	0.0816	0.4047	0.0623	0.0791	0.0602	G4 D 0
0.0542	0.0595	0.1796	0.0543	0.0598	0.0523	G4 D 0
0.0536	0.0552	0.0905	0.0548	0.0545	0.0537	G4 D 0
0.0496	0.0512	0.0578	0.0494	0.0518	0.0515	G4 D 0
0.0522	0.0510	0.0526	0.0500	0.0501	0.0512	G4 D 0
0.0503	0.0506	0.0491	0.0524	0.0532	0.0491	G4 D 0
1.2077	1.5774	1.7095	1.5908	1.2735	1.0230	G4 2 wk post vaccine
0.6725	1.0658	1.4830	1.1651	0.8374	0.5347	G4 2 wk post vaccine
0.3312	0.6937	1.2187	0.8281	0.4065	0.2236	G4 2 wk post vaccine
0.1489	0.3143	0.8376	0.3910	0.1750	0.1019	G4 2 wk post vaccine
0.0739	0.1306	0.4216	0.1649	0.0818	0.0641	G4 2 wk post vaccine
0.0590	0.0704	0.1770	0.0871	0.0633	0.0542	G4 2 wk post vaccine
0.0523	0.0548	0.0891	0.0616	0.0534	0.0516	G4 2 wk post vaccine
0.0506	0.0507	0.0586	0.0527	0.0493	0.0500	G4 2 wk post vaccine

1.3367	1.3722	1.6993	1.5934	1.2465	1.0728	G 4 wk post vaccine
0.5111	0.7226	1.5440	0.9916	0.5968	0.4496	G 4 wk post vaccine
0.2527	0.3469	1.1588	0.5810	0.2742	0.2059	G 4 wk post vaccine
0.1075	0.1514	0.6566	0.2574	0.1204	0.1064	G 4 wk post vaccine
0.0710	0.0801	0.3123	0.1214	0.0749	0.0698	G 4 wk post vaccine
0.0569	0.0586	0.1265	0.0751	0.0574	0.0569	G 4 wk post vaccine
0.0540	0.0536	0.0740	0.0581	0.0554	0.0522	G 4 wk post vaccine
0.0510	0.0516	0.0648	0.0563	0.0539	0.0515	G 4 wk post vaccine
1.6010	1.5573	1.5373	1.5200	1.5242	1.5311	G4 wk 6 (2 wk post 2nd boost)
1.8266	2.0331	1.9512	1.9156	1.8654	1.7977	G4 wk 6 (2 wk post 2nd boost)
1.5310	1.6149	1.6193	1.5181	1.4541	1.3539	G4 wk 6 (2 wk post 2nd boost)
1.1853	1.5240	1.6714	1.6359	1.2077	0.9188	G4 wk 6 (2 wk post 2nd boost)
0.7101	1.0766	1.4553	1.3037	0.6589	0.4360	G4 wk 6 (2 wk post 2nd boost)
0.2916	0.5072	0.9140	0.7105	0.2536	0.2159	G4 wk 6 (2 wk post 2nd boost)
0.1390	0.2317	0.5639	0.3780	0.1294	0.1092	G4 wk 6 (2 wk post 2nd boost)
0.0915	0.1134	0.2654	0.1857	0.0777	0.0772	G4 wk 6 (2 wk post 2nd boost)
1.2885	1.3728	1.6423	1.7569	1.2545	1.4190	G4 wk 12 (8 wk post second boost)
0.6921	0.8691	1.3888	1.6192	0.8867	0.8347	G4 wk 12 (8 wk post second boost)
0.3316	0.4560	1.0447	1.2220	0.3777	0.4459	G4 wk 12 (8 wk post second boost)
0.1468	0.2263	0.6886	0.7728	0.1608	0.2124	G4 wk 12 (8 wk post second boost)
0.0805	0.1111	0.3718	0.3206	0.0858	0.1184	G4 wk 12 (8 wk post second boost)
0.0713	0.0683	0.1446	0.1727	0.0666	0.0788	G4 wk 12 (8 wk post second boost)
0.0582	0.0634	0.1023	0.1155	0.0584	0.0582	G4 wk 12 (8 wk post second boost)
0.0557	0.0559	0.0669	0.0732	0.0548	0.0538	G4 wk 12 (8 wk post second boost)

1.6479	1.6276	1.5532	1.6810	1.7629	1.5283	G4 wk 14 (2 wk post 3rd boost)
1.4669	1.7767	1.7096	1.5734	1.3735	1.3365	G4 wk 14 (2 wk post 3rd boost)
1.0279	1.4029	1.2580	1.4242	0.9390	1.0522	G4 wk 14 (2 wk post 3rd boost)
0.5489	0.9327	1.0062	1.0655	0.4421	0.4740	G4 wk 14 (2 wk post 3rd boost)
0.2863	0.4962	0.6186	0.6823	0.2101	0.2556	G4 wk 14 (2 wk post 3rd boost)
0.1488	0.2851	0.3581	0.3754	0.1190	0.1284	G4 wk 14 (2 wk post 3rd boost)
0.0724	0.1252	0.1734	0.1976	0.0749	0.0759	G4 wk 14 (2 wk post 3rd boost)
0.0659	0.0891	0.1058	0.1155	0.0636	0.0616	G4 wk 14 (2 wk post 3rd boost)
