# Supplementary material for: HIV-1 T cell epitopes targeted to Rhesus macaque CD40 and DCIR: A comparative study of prototype dendritic cell targeting therapeutic vaccine candidates
Source: PLoS One. 2018 Nov 30;13(11):e0207794. doi: 10.1371/journal.pone.0207794 (PMC6267996; doi:10.1371/journal.pone.0207794)
Supplement: S1 File — The tables show interferon gamma ELISPOT responses to each peptide pool tested for each NHP at every sample time. (PDF) [file pone.0207794.s011.pdf]

Date	Group	Animal	Peptide	SFC/10^6
2.18.13	1	R370	Non	0
2.18.13	1	R370	p24 pool 1	0
2.18.13	1	R370	p24 pool 2	0
2.18.13	1	R370	p24 pool 3	0
2.18.13	1	R370	p24 pool 4	0
2.18.13	1	R370	p24 pool 5	0
2.18.13	1	R370	p2/p6/p7 pool 1	0
2.18.13	1	R370	p2/p6/p7 pool 2	0
2.18.13	1	R370	p2/p6/p7 pool 3	0
2.18.13	1	R370	p17 pool 1	0
2.18.13	1	R370	p17 pool 2	0
2.18.13	1	R370	p17 pool 3	0
2.18.13	1	R370	pol pool 1	0
2.18.13	1	R370	pol pool 2	0
2.18.13	1	R370	pol pool 3	0
2.18.13	1	R370	pol pool 4	0
2.18.13	1	R370	nef pool 1	0
2.18.13	1	R370	nef pool 2	0
2.18.13	1	R370	nef pool 3	0
2.18.13	1	R370	hIgG4-HIV5pep	0
2.18.13	1	R370	hIgG4	0
2.18.13	1	R370	Cohesin-nef66 (C1077)	0
2.18.13	1	R370	Cohesin-nef66 (C1078)	0
2.18.13	1	R370	Cohesin-gag17	0
2.18.13	1	R370	Cohesin-gag253	0
2.18.13	1	R370	Cohesin–pol158	0
2.18.13	1	R370	Cohesin	0
2.18.13	1	R370	pha	1715
2.18.13	1	R371	Non	0
2.18.13	1	R371	p24 pool 1	5
2.18.13	1	R371	p24 pool 2	0
2.18.13	1	R371	p24 pool 3	5
2.18.13	1	R371	p24 pool 4	0
2.18.13	1	R371	p24 pool 5	0
2.18.13	1	R371	p2/p6/p7 pool 1	0
2.18.13	1	R371	p2/p6/p7 pool 2	0
2.18.13	1	R371	p2/p6/p7 pool 3	0
2.18.13	1	R371	p17 pool 1	0
2.18.13	1	R371	p17 pool 2	0
2.18.13	1	R371	p17 pool 3	5
2.18.13	1	R371	pol pool 1	5
2.18.13	1	R371	pol pool 2	5
2.18.13	1	R371	pol pool 3	0
2.18.13	1	R371	pol pool 4	0
2.18.13	1	R371	nef pool 1	5
2.18.13	1	R371	nef pool 2	0
2.18.13	1	R371	nef pool 3	0
2.18.13	1	R371	hIgG4-HIV5pep	0
2.18.13	1	R371	hIgG4	0
2.18.13	1	R371	Cohesin-nef66 (C1077)	0
2.18.13	1	R371	Cohesin-nef66 (C1078)	10
2.18.13	1	R371	Cohesin-gag17	0
2.18.13	1	R371	Cohesin-gag253	5
2.18.13	1	R371	Cohesin–pol158	0
2.18.13	1	R371	Cohesin	5
2.18.13	1	R371	pha	2660
2.18.13	1	R373	Non	0
2.18.13	1	R373	p24 pool 1	0
2.18.13	1	R373	p24 pool 2	0
2.18.13	1	R373	p24 pool 3	5
2.18.13	1	R373	p24 pool 4	5
2.18.13	1	R373	p24 pool 5	5
2.18.13	1	R373	p2/p6/p7 pool 1	5
2.18.13	1	R373	p2/p6/p7 pool 2	5
2.18.13	1	R373	p2/p6/p7 pool 3	0
2.18.13	1	R373	p17 pool 1	5
2.18.13	1	R373	p17 pool 2	0
2.18.13	1	R373	p17 pool 3	0
2.18.13	1	R373	pol pool 1	5
2.18.13	1	R373	pol pool 2	5
2.18.13	1	R373	pol pool 3	5
2.18.13	1	R373	pol pool 4	5
2.18.13	1	R373	nef pool 1	5
2.18.13	1	R373	nef pool 2	0
2.18.13	1	R373	nef pool 3	0
2.18.13	1	R373	hIgG4-HIV5pep	0
2.18.13	1	R373	hIgG4	0
2.18.13	1	R373	Cohesin-nef66 (C1077)	0
2.18.13	1	R373	Cohesin-nef66 (C1078)	0
2.18.13	1	R373	Cohesin-gag17	0
2.18.13	1	R373	Cohesin-gag253	0
2.18.13	1	R373	Cohesin–pol158	0
2.18.13	1	R373	Cohesin	0
2.18.13	1	R373	pha	1545
2.18.13	1	R374	Non	0
2.18.13	1	R374	p24 pool 1	0
2.18.13	1	R374	p24 pool 2	20

Date	Group	Animal	Peptide	SFC/10^6
2.18.13	2	R376	Non	0
2.18.13	2	R376	p24 pool 1	0
2.18.13	2	R376	p24 pool 2	5
2.18.13	2	R376	p24 pool 3	0
2.18.13	2	R376	p24 pool 4	0
2.18.13	2	R376	p24 pool 5	0
2.18.13	2	R376	p2/p6/p7 pool 1	0
2.18.13	2	R376	p2/p6/p7 pool 2	0
2.18.13	2	R376	p2/p6/p7 pool 3	0
2.18.13	2	R376	p17 pool 1	0
2.18.13	2	R376	p17 pool 2	0
2.18.13	2	R376	p17 pool 3	0
2.18.13	2	R376	pol pool 1	0
2.18.13	2	R376	pol pool 2	0
2.18.13	2	R376	pol pool 3	0
2.18.13	2	R376	pol pool 4	0
2.18.13	2	R376	nef pool 1	0
2.18.13	2	R376	nef pool 2	0
2.18.13	2	R376	nef pool 3	0
2.18.13	2	R376	hIgG4-HIV5pep	0
2.18.13	2	R376	hIgG4	0
2.18.13	2	R376	Cohesin-nef66 (C1077)	0
2.18.13	2	R376	Cohesin-nef66 (C1078)	0
2.18.13	2	R376	Cohesin-gag17	0
2.18.13	2	R376	Cohesin-gag253	0
2.18.13	2	R376	Cohesin–pol158	0
2.18.13	2	R376	Cohesin	0
2.18.13	2	R376	pha	2115
2.18.13	2	R377	Non	0
2.18.13	2	R377	p24 pool 1	10
2.18.13	2	R377	p24 pool 2	5
2.18.13	2	R377	p24 pool 3	5
2.18.13	2	R377	p24 pool 4	5
2.18.13	2	R377	p24 pool 5	0
2.18.13	2	R377	p2/p6/p7 pool 1	5
2.18.13	2	R377	p2/p6/p7 pool 2	5
2.18.13	2	R377	p2/p6/p7 pool 3	5
2.18.13	2	R377	p17 pool 1	20
2.18.13	2	R377	p17 pool 2	15
2.18.13	2	R377	p17 pool 3	5
2.18.13	2	R377	pol pool 1	5
2.18.13	2	R377	pol pool 2	10
2.18.13	2	R377	pol pool 3	5
2.18.13	2	R377	pol pool 4	10
2.18.13	2	R377	nef pool 1	5
2.18.13	2	R377	nef pool 2	10
2.18.13	2	R377	nef pool 3	5
2.18.13	2	R377	hIgG4-HIV5pep	5
2.18.13	2	R377	hIgG4	5
2.18.13	2	R377	Cohesin-nef66 (C1077)	10
2.18.13	2	R377	Cohesin-nef66 (C1078)	5
2.18.13	2	R377	Cohesin-gag17	10
2.18.13	2	R377	Cohesin-gag253	5
2.18.13	2	R377	Cohesin–pol158	5
2.18.13	2	R377	Cohesin	5
2.18.13	2	R377	pha	2340
2.18.13	2	R378	Non	0
2.18.13	2	R378	p24 pool 1	5
2.18.13	2	R378	p24 pool 2	5
2.18.13	2	R378	p24 pool 3	0
2.18.13	2	R378	p24 pool 4	0
2.18.13	2	R378	p24 pool 5	0
2.18.13	2	R378	p2/p6/p7 pool 1	5
2.18.13	2	R378	p2/p6/p7 pool 2	0
2.18.13	2	R378	p2/p6/p7 pool 3	0
2.18.13	2	R378	p17 pool 1	0
2.18.13	2	R378	p17 pool 2	5
2.18.13	2	R378	p17 pool 3	5
2.18.13	2	R378	pol pool 1	5
2.18.13	2	R378	pol pool 2	5
2.18.13	2	R378	pol pool 3	5
2.18.13	2	R378	pol pool 4	0
2.18.13	2	R378	nef pool 1	5
2.18.13	2	R378	nef pool 2	0
2.18.13	2	R378	nef pool 3	5
2.18.13	2	R378	hIgG4-HIV5pep	10
2.18.13	2	R378	hIgG4	0
2.18.13	2	R378	Cohesin-nef66 (C1077)	0
2.18.13	2	R378	Cohesin-nef66 (C1078)	5
2.18.13	2	R378	Cohesin-gag17	0
2.18.13	2	R378	Cohesin-gag253	20
2.18.13	2	R378	Cohesin–pol158	5
2.18.13	2	R378	Cohesin	5
2.18.13	2	R378	pha	2205
2.18.13	2	R380	Non	0
2.18.13	2	R380	p24 pool 1	0
2.18.13	2	R380	p24 pool 2	0

Date	Group	Animal	Peptide	SFC/10^6
2.18.13	3	R368	Non	0
2.18.13	3	R368	p24 pool 1	0
2.18.13	3	R368	p24 pool 2	0
2.18.13	3	R368	p24 pool 3	0
2.18.13	3	R368	p24 pool 4	0
2.18.13	3	R368	p24 pool 5	0
2.18.13	3	R368	p2/p6/p7 pool 1	5
2.18.13	3	R368	p2/p6/p7 pool 2	0
2.18.13	3	R368	p2/p6/p7 pool 3	5
2.18.13	3	R368	p17 pool 1	0
2.18.13	3	R368	p17 pool 2	0
2.18.13	3	R368	p17 pool 3	0
2.18.13	3	R368	pol pool 1	5
2.18.13	3	R368	pol pool 2	5
2.18.13	3	R368	pol pool 3	0
2.18.13	3	R368	pol pool 4	5
2.18.13	3	R368	nef pool 1	5
2.18.13	3	R368	nef pool 2	0
2.18.13	3	R368	nef pool 3	0
2.18.13	3	R368	hIgG4-HIV5pep	5
2.18.13	3	R368	hIgG4	0
2.18.13	3	R368	Cohesin-nef66 (C1077)	5
2.18.13	3	R368	Cohesin-nef66 (C1078)	0
2.18.13	3	R368	Cohesin-gag17	0
2.18.13	3	R368	Cohesin-gag253	5
2.18.13	3	R368	Cohesin–pol158	5
2.18.13	3	R368	Cohesin	5
2.18.13	3	R368	pha	2085
2.18.13	3	R375	Non	0
2.18.13	3	R375	p24 pool 1	0
2.18.13	3	R375	p24 pool 2	0
2.18.13	3	R375	p24 pool 3	0
2.18.13	3	R375	p24 pool 4	0
2.18.13	3	R375	p24 pool 5	0
2.18.13	3	R375	p2/p6/p7 pool 1	0
2.18.13	3	R375	p2/p6/p7 pool 2	0
2.18.13	3	R375	p2/p6/p7 pool 3	0
2.18.13	3	R375	p17 pool 1	0
2.18.13	3	R375	p17 pool 2	0
2.18.13	3	R375	p17 pool 3	0
2.18.13	3	R375	pol pool 1	0
2.18.13	3	R375	pol pool 2	0
2.18.13	3	R375	pol pool 3	0
2.18.13	3	R375	pol pool 4	0
2.18.13	3	R375	nef pool 1	0
2.18.13	3	R375	nef pool 2	0
2.18.13	3	R375	nef pool 3	0
2.18.13	3	R375	hIgG4-HIV5pep	0
2.18.13	3	R375	hIgG4	0
2.18.13	3	R375	Cohesin-nef66 (C1077)	0
2.18.13	3	R375	Cohesin-nef66 (C1078)	0
2.18.13	3	R375	Cohesin-gag17	0
2.18.13	3	R375	Cohesin-gag253	5
2.18.13	3	R375	Cohesin–pol158	5
2.18.13	3	R375	Cohesin	0
2.18.13	3	R375	pha	2220
2.18.13	3	R379	Non	0
2.18.13	3	R379	p24 pool 1	5
2.18.13	3	R379	p24 pool 2	0
2.18.13	3	R379	p24 pool 3	5
2.18.13	3	R379	p24 pool 4	0
2.18.13	3	R379	p24 pool 5	0
2.18.13	3	R379	p2/p6/p7 pool 1	0
2.18.13	3	R379	p2/p6/p7 pool 2	5
2.18.13	3	R379	p2/p6/p7 pool 3	0
2.18.13	3	R379	p17 pool 1	5
2.18.13	3	R379	p17 pool 2	0
2.18.13	3	R379	p17 pool 3	0
2.18.13	3	R379	pol pool 1	0
2.18.13	3	R379	pol pool 2	0
2.18.13	3	R379	pol pool 3	0
2.18.13	3	R379	pol pool 4	0
2.18.13	3	R379	nef pool 1	0
2.18.13	3	R379	nef pool 2	0
2.18.13	3	R379	nef pool 3	0
2.18.13	3	R379	hIgG4-HIV5pep	0
2.18.13	3	R379	hIgG4	0
2.18.13	3	R379	Cohesin-nef66 (C1077)	0
2.18.13	3	R379	Cohesin-nef66 (C1078)	0
2.18.13	3	R379	Cohesin-gag17	0
2.18.13	3	R379	Cohesin-gag253	10
2.18.13	3	R379	Cohesin–pol158	5
2.18.13	3	R379	Cohesin	0
2.18.13	3	R379	pha	2020
2.18.13	3	R381	Non	0
2.18.13	3	R381	p24 pool 1	0
2.18.13	3	R381	p24 pool 2	0

Date	Group	Animal	Peptide	SFC/10^6
2.18.13	4	R386	Non	0
2.18.13	4	R386	p24 pool 1	0
2.18.13	4	R386	p24 pool 2	0
2.18.13	4	R386	p24 pool 3	5
2.18.13	4	R386	p24 pool 4	0
2.18.13	4	R386	p24 pool 5	0
2.18.13	4	R386	p2/p6/p7 pool 1	0
2.18.13	4	R386	p2/p6/p7 pool 2	0
2.18.13	4	R386	p2/p6/p7 pool 3	5
2.18.13	4	R386	p17 pool 1	0
2.18.13	4	R386	p17 pool 2	0
2.18.13	4	R386	p17 pool 3	0
2.18.13	4	R386	pol pool 1	0
2.18.13	4	R386	pol pool 2	0
2.18.13	4	R386	pol pool 3	0
2.18.13	4	R386	pol pool 4	5
2.18.13	4	R386	nef pool 1	0
2.18.13	4	R386	nef pool 2	0
2.18.13	4	R386	nef pool 3	5
2.18.13	4	R386	hIgG4-HIV5pep	0
2.18.13	4	R386	hIgG4	0
2.18.13	4	R386	Cohesin-nef66 (C1077)	5
2.18.13	4	R386	Cohesin-nef66 (C1078)	0
2.18.13	4	R386	Cohesin-gag17	0
2.18.13	4	R386	Cohesin-gag253	0
2.18.13	4	R386	Cohesin–pol158	5
2.18.13	4	R386	Cohesin	5
2.18.13	4	R386	pha	TNTC
2.18.13	4	R389	Non	0
2.18.13	4	R389	p24 pool 1	0
2.18.13	4	R389	p24 pool 2	0
2.18.13	4	R389	p24 pool 3	0
2.18.13	4	R389	p24 pool 4	0
2.18.13	4	R389	p24 pool 5	0
2.18.13	4	R389	p2/p6/p7 pool 1	0
2.18.13	4	R389	p2/p6/p7 pool 2	0
2.18.13	4	R389	p2/p6/p7 pool 3	0
2.18.13	4	R389	p17 pool 1	0
2.18.13	4	R389	p17 pool 2	0
2.18.13	4	R389	p17 pool 3	0
2.18.13	4	R389	pol pool 1	5
2.18.13	4	R389	pol pool 2	0
2.18.13	4	R389	pol pool 3	0
2.18.13	4	R389	pol pool 4	0
2.18.13	4	R389	nef pool 1	0
2.18.13	4	R389	nef pool 2	0
2.18.13	4	R389	nef pool 3	5
2.18.13	4	R389	hIgG4-HIV5pep	10
2.18.13	4	R389	hIgG4	0
2.18.13	4	R389	Cohesin-nef66 (C1077)	0
2.18.13	4	R389	Cohesin-nef66 (C1078)	5
2.18.13	4	R389	Cohesin-gag17	0
2.18.13	4	R389	Cohesin-gag253	0
2.18.13	4	R389	Cohesin–pol158	15
2.18.13	4	R389	Cohesin	0
2.18.13	4	R389	pha	2130
2.18.13	4	R390	Non	0
2.18.13	4	R390	p24 pool 1	0
2.18.13	4	R390	p24 pool 2	5
2.18.13	4	R390	p24 pool 3	5
2.18.13	4	R390	p24 pool 4	5
2.18.13	4	R390	p24 pool 5	5
2.18.13	4	R390	p2/p6/p7 pool 1	5
2.18.13	4	R390	p2/p6/p7 pool 2	5
2.18.13	4	R390	p2/p6/p7 pool 3	0
2.18.13	4	R390	p17 pool 1	5
2.18.13	4	R390	p17 pool 2	5
2.18.13	4	R390	p17 pool 3	0
2.18.13	4	R390	pol pool 1	0
2.18.13	4	R390	pol pool 2	0
2.18.13	4	R390	pol pool 3	0
2.18.13	4	R390	pol pool 4	0
2.18.13	4	R390	nef pool 1	5
2.18.13	4	R390	nef pool 2	0
2.18.13	4	R390	nef pool 3	5
2.18.13	4	R390	hIgG4-HIV5pep	5
2.18.13	4	R390	hIgG4	0
2.18.13	4	R390	Cohesin-nef66 (C1077)	0
2.18.13	4	R390	Cohesin-nef66 (C1078)	5
2.18.13	4	R390	Cohesin-gag17	0
2.18.13	4	R390	Cohesin-gag253	10
2.18.13	4	R390	Cohesin–pol158	10
2.18.13	4	R390	Cohesin	0
2.18.13	4	R390	pha	2215
2.18.13	4	R391	Non	0
2.18.13	4	R391	p24 pool 1	0
2.18.13	4	R391	p24 pool 2	10

2.18.13	1	R374	p24 pool 3	25	2.18.13	2	R380	p24 pool 3	0	2.18.13	3	R381	p24 pool 3	0	2.18.13	4	R391	p24 pool 3	5
2.18.13	1	R374	p24 pool 4	0	2.18.13	2	R380	p24 pool 4	0	2.18.13	3	R381	p24 pool 4	5	2.18.13	4	R391	p24 pool 4	5
2.18.13	1	R374	p24 pool 5	0	2.18.13	2	R380	p24 pool 5	0	2.18.13	3	R381	p24 pool 5	0	2.18.13	4	R391	p24 pool 5	5
2.18.13	1	R374	p2/p6/p7 pool 1	5	2.18.13	2	R380	p2/p6/p7 pool 1	0	2.18.13	3	R381	p2/p6/p7 pool 1	0	2.18.13	4	R391	p2/p6/p7 pool 1	0
2.18.13	1	R374	p2/p6/p7 pool 2	0	2.18.13	2	R380	p2/p6/p7 pool 2	0	2.18.13	3	R381	p2/p6/p7 pool 2	0	2.18.13	4	R391	p2/p6/p7 pool 2	0
2.18.13	1	R374	p2/p6/p7 pool 3	0	2.18.13	2	R380	p2/p6/p7 pool 3	0	2.18.13	3	R381	p2/p6/p7 pool 3	0	2.18.13	4	R391	p2/p6/p7 pool 3	0
2.18.13	1	R374	p17 pool 1	10	2.18.13	2	R380	p17 pool 1	0	2.18.13	3	R381	p17 pool 1	0	2.18.13	4	R391	p17 pool 1	0
2.18.13	1	R374	p17 pool 2	10	2.18.13	2	R380	p17 pool 2	0	2.18.13	3	R381	p17 pool 2	0	2.18.13	4	R391	p17 pool 2	10
2.18.13	1	R374	p17 pool 3	0	2.18.13	2	R380	p17 pool 3	0	2.18.13	3	R381	p17 pool 3	0	2.18.13	4	R391	p17 pool 3	0
2.18.13	1	R374	pol pool 1	0	2.18.13	2	R380	pol pool 1	0	2.18.13	3	R381	pol pool 1	0	2.18.13	4	R391	pol pool 1	0
2.18.13	1	R374	pol pool 2	0	2.18.13	2	R380	pol pool 2	5	2.18.13	3	R381	pol pool 2	150	2.18.13	4	R391	pol pool 2	15
2.18.13	1	R374	pol pool 3	5	2.18.13	2	R380	pol pool 3	5	2.18.13	3	R381	pol pool 3	0	2.18.13	4	R391	pol pool 3	0
2.18.13	1	R374	pol pool 4	5	2.18.13	2	R380	pol pool 4	0	2.18.13	3	R381	pol pool 4	0	2.18.13	4	R391	pol pool 4	0
2.18.13	1	R374	nef pool 1	0	2.18.13	2	R380	nef pool 1	0	2.18.13	3	R381	nef pool 1	0	2.18.13	4	R391	nef pool 1	5
2.18.13	1	R374	nef pool 2	0	2.18.13	2	R380	nef pool 2	0	2.18.13	3	R381	nef pool 2	0	2.18.13	4	R391	nef pool 2	5
2.18.13	1	R374	nef pool 3	0	2.18.13	2	R380	nef pool 3	5	2.18.13	3	R381	nef pool 3	0	2.18.13	4	R391	nef pool 3	5
2.18.13	1	R374	hIgG4-HIV5pep	10	2.18.13	2	R380	hIgG4-HIV5pep	5	2.18.13	3	R381	hIgG4-HIV5pep	0	2.18.13	4	R391	hIgG4-HIV5pep	15
2.18.13	1	R374	hIgG4	0	2.18.13	2	R380	hIgG4	0	2.18.13	3	R381	hIgG4	0	2.18.13	4	R391	hIgG4	5
2.18.13	1	R374	Cohesin-nef66 (C1077)	0	2.18.13	2	R380	Cohesin-nef66 (C1077)	5	2.18.13	3	R381	Cohesin-nef66 (C1077)	0	2.18.13	4	R391	Cohesin-nef66 (C1077)	5
2.18.13	1	R374	Cohesin-nef66 (C1078)	5	2.18.13	2	R380	Cohesin-nef66 (C1078)	5	2.18.13	3	R381	Cohesin-nef66 (C1078)	0	2.18.13	4	R391	Cohesin-nef66 (C1078)	10
2.18.13	1	R374	Cohesin-gag17	5	2.18.13	2	R380	Cohesin-gag17	0	2.18.13	3	R381	Cohesin-gag17	0	2.18.13	4	R391	Cohesin-gag17	10
2.18.13	1	R374	Cohesin-gag253	45	2.18.13	2	R380	Cohesin-gag253	0	2.18.13	3	R381	Cohesin-gag253	0	2.18.13	4	R391	Cohesin-gag253	10
2.18.13	1	R374	Cohesin–pol158	0	2.18.13	2	R380	Cohesin–pol158	10	2.18.13	3	R381	Cohesin–pol158	100	2.18.13	4	R391	Cohesin–pol158	10
2.18.13	1	R374	Cohesin	5	2.18.13	2	R380	Cohesin	0	2.18.13	3	R381	Cohesin	0	2.18.13	4	R391	Cohesin	5
2.18.13	1	R374	pha	2320	2.18.13	2	R380	pha	1755	2.18.13	3	R381	pha	2875	2.18.13	4	R391	pha	2170
2.18.13	1	R369	Non	0	2.18.13	2	R382	Non	0	2.18.13	3	R387	Non	0	2.18.13	4	R372	Non	0
2.18.13	1	R369	p24 pool 1	5	2.18.13	2	R382	p24 pool 1	5	2.18.13	3	R387	p24 pool 1	0	2.18.13	4	R372	p24 pool 1	5
2.18.13	1	R369	p24 pool 2	0	2.18.13	2	R382	p24 pool 2	0	2.18.13	3	R387	p24 pool 2	0	2.18.13	4	R372	p24 pool 2	0
2.18.13	1	R369	p24 pool 3	5	2.18.13	2	R382	p24 pool 3	0	2.18.13	3	R387	p24 pool 3	0	2.18.13	4	R372	p24 pool 3	0
2.18.13	1	R369	p24 pool 4	0	2.18.13	2	R382	p24 pool 4	0	2.18.13	3	R387	p24 pool 4	0	2.18.13	4	R372	p24 pool 4	0
2.18.13	1	R369	p24 pool 5	0	2.18.13	2	R382	p24 pool 5	5	2.18.13	3	R387	p24 pool 5	0	2.18.13	4	R372	p24 pool 5	0
2.18.13	1	R369	p2/p6/p7 pool 1	0	2.18.13	2	R382	p2/p6/p7 pool 1	0	2.18.13	3	R387	p2/p6/p7 pool 1	0	2.18.13	4	R372	p2/p6/p7 pool 1	0
2.18.13	1	R369	p2/p6/p7 pool 2	0	2.18.13	2	R382	p2/p6/p7 pool 2	5	2.18.13	3	R387	p2/p6/p7 pool 2	0	2.18.13	4	R372	p2/p6/p7 pool 2	0
2.18.13	1	R369	p2/p6/p7 pool 3	0	2.18.13	2	R382	p2/p6/p7 pool 3	0	2.18.13	3	R387	p2/p6/p7 pool 3	0	2.18.13	4	R372	p2/p6/p7 pool 3	0
2.18.13	1	R369	p17 pool 1	5	2.18.13	2	R382	p17 pool 1	5	2.18.13	3	R387	p17 pool 1	0	2.18.13	4	R372	p17 pool 1	0
2.18.13	1	R369	p17 pool 2	0	2.18.13	2	R382	p17 pool 2	0	2.18.13	3	R387	p17 pool 2	0	2.18.13	4	R372	p17 pool 2	0
2.18.13	1	R369	p17 pool 3	5	2.18.13	2	R382	p17 pool 3	0	2.18.13	3	R387	p17 pool 3	0	2.18.13	4	R372	p17 pool 3	0
2.18.13	1	R369	pol pool 1	0	2.18.13	2	R382	pol pool 1	0	2.18.13	3	R387	pol pool 1	0	2.18.13	4	R372	pol pool 1	0
2.18.13	1	R369	pol pool 2	0	2.18.13	2	R382	pol pool 2	0	2.18.13	3	R387	pol pool 2	0	2.18.13	4	R372	pol pool 2	0
2.18.13	1	R369	pol pool 3	0	2.18.13	2	R382	pol pool 3	0	2.18.13	3	R387	pol pool 3	0	2.18.13	4	R372	pol pool 3	0
2.18.13	1	R369	pol pool 4	0	2.18.13	2	R382	pol pool 4	0	2.18.13	3	R387	pol pool 4	0	2.18.13	4	R372	pol pool 4	0
2.18.13	1	R369	nef pool 1	0	2.18.13	2	R382	nef pool 1	0	2.18.13	3	R387	nef pool 1	0	2.18.13	4	R372	nef pool 1	0
2.18.13	1	R369	nef pool 2	0	2.18.13	2	R382	nef pool 2	0	2.18.13	3	R387	nef pool 2	0	2.18.13	4	R372	nef pool 2	0
2.18.13	1	R369	nef pool 3	5	2.18.13	2	R382	nef pool 3	0	2.18.13	3	R387	nef pool 3	0	2.18.13	4	R372	nef pool 3	5
2.18.13	1	R369	hIgG4-HIV5pep	10	2.18.13	2	R382	hIgG4-HIV5pep	0	2.18.13	3	R387	hIgG4-HIV5pep	0	2.18.13	4	R372	hIgG4-HIV5pep	0
2.18.13	1	R369	hIgG4	0	2.18.13	2	R382	hIgG4	0	2.18.13	3	R387	hIgG4	0	2.18.13	4	R372	hIgG4	0
2.18.13	1	R369	Cohesin-nef66 (C1077)	0	2.18.13	2	R382	Cohesin-nef66 (C1077)	0	2.18.13	3	R387	Cohesin-nef66 (C1077)	0	2.18.13	4	R372	Cohesin-nef66 (C1077)	0
2.18.13	1	R369	Cohesin-nef66 (C1078)	5	2.18.13	2	R382	Cohesin-nef66 (C1078)	0	2.18.13	3	R387	Cohesin-nef66 (C1078)	0	2.18.13	4	R372	Cohesin-nef66 (C1078)	0
2.18.13	1	R369	Cohesin-gag17	0	2.18.13	2	R382	Cohesin-gag17	5	2.18.13	3	R387	Cohesin-gag17	0	2.18.13	4	R372	Cohesin-gag17	0
2.18.13	1	R369	Cohesin-gag253	0	2.18.13	2	R382	Cohesin-gag253	0	2.18.13	3	R387	Cohesin-gag253	0	2.18.13	4	R372	Cohesin-gag253	0
2.18.13	1	R369	Cohesin–pol158	0	2.18.13	2	R382	Cohesin–pol158	5	2.18.13	3	R387	Cohesin–pol158	0	2.18.13	4	R372	Cohesin–pol158	0
2.18.13	1	R369	Cohesin	15	2.18.13	2	R382	Cohesin	5	2.18.13	3	R387	Cohesin	0	2.18.13	4	R372	Cohesin	0
2.18.13	1	R369	pha	2180	2.18.13	2	R382	pha	2005	2.18.13	3	R387	pha	2155	2.18.13	4	R372	pha	2125
2.18.13	1	R385	Non	0	2.18.13	2	R384	Non	0	2.18.13	3	R388	Non	0	2.18.13	4	R383	Non	0
2.18.13	1	R385	p24 pool 1	0	2.18.13	2	R384	p24 pool 1	0	2.18.13	3	R388	p24 pool 1	0	2.18.13	4	R383	p24 pool 1	0
2.18.13	1	R385	p24 pool 2	0	2.18.13	2	R384	p24 pool 2	0	2.18.13	3	R388	p24 pool 2	0	2.18.13	4	R383	p24 pool 2	0
2.18.13	1	R385	p24 pool 3	5	2.18.13	2	R384	p24 pool 3	0	2.18.13	3	R388	p24 pool 3	0	2.18.13	4	R383	p24 pool 3	0
2.18.13	1	R385	p24 pool 4	0	2.18.13	2	R384	p24 pool 4	0	2.18.13	3	R388	p24 pool 4	0	2.18.13	4	R383	p24 pool 4	0
2.18.13	1	R385	p24 pool 5	0	2.18.13	2	R384	p24 pool 5	0	2.18.13	3	R388	p24 pool 5	0	2.18.13	4	R383	p24 pool 5	0
2.18.13	1	R385	p2/p6/p7 pool 1	0	2.18.13	2	R384	p2/p6/p7 pool 1	0	2.18.13	3	R388	p2/p6/p7 pool 1	0	2.18.13	4	R383	p2/p6/p7 pool 1	0
2.18.13	1	R385	p2/p6/p7 pool 2	0	2.18.13	2	R384	p2/p6/p7 pool 2	5	2.18.13	3	R388	p2/p6/p7 pool 2	0	2.18.13	4	R383	p2/p6/p7 pool 2	0
2.18.13	1	R385	p2/p6/p7 pool 3	0	2.18.13	2	R384	p2/p6/p7 pool 3	0	2.18.13	3	R388	p2/p6/p7 pool 3	0	2.18.13	4	R383	p2/p6/p7 pool 3	0
2.18.13	1	R385	p17 pool 1	0	2.18.13	2	R384	p17 pool 1	0	2.18.13	3	R388	p17 pool 1	0	2.18.13	4	R383	p17 pool 1	0
2.18.13	1	R385	p17 pool 2	0	2.18.13	2	R384	p17 pool 2	0	2.18.13	3	R388	p17 pool 2	0	2.18.13	4	R383	p17 pool 2	0
2.18.13	1	R385	p17 pool 3	5	2.18.13	2	R384	p17 pool 3	0	2.18.13	3	R388	p17 pool 3	0	2.18.13	4	R383	p17 pool 3	0
2.18.13	1	R385	pol pool 1	0	2.18.13	2	R384	pol pool 1	0	2.18.13	3	R388	pol pool 1	0	2.18.13	4	R383	pol pool 1	0
2.18.13	1	R385	pol pool 2	0	2.18.13	2	R384	pol pool 2	0	2.18.13	3	R388	pol pool 2	45	2.18.13	4	R383	pol pool 2	0
2.18.13	1	R385	pol pool 3	0	2.18.13	2	R384	pol pool 3	0	2.18.13	3	R388	pol pool 3	0	2.18.13	4	R383	pol pool 3	0
2.18.13	1	R385	pol pool 4	0	2.18.13	2	R384	pol pool 4	0	2.18.13	3	R388	pol pool 4	0	2.18.13	4	R383	pol pool 4	0
2.18.13	1	R385	nef pool 1	0	2.18.13	2	R384	nef pool 1	0	2.18.13	3	R388	nef pool 1	0	2.18.13	4	R383	nef pool 1	0
2.18.13	1	R385	nef pool 2	0	2.18.13	2	R384	nef pool 2	0	2.18.13	3	R388	nef pool 2	85	2.18.13	4	R383	nef pool 2	0
2.18.13	1	R385	nef pool 3	0	2.18.13	2	R384	nef pool 3	0	2.18.13	3	R388	nef pool 3	0	2.18.13	4	R383	nef pool 3	0
2.18.13	1	R385	hIgG4-HIV5pep	5	2.18.13	2	R384	hIgG4-HIV5pep	5	2.18.13	3	R388	hIgG4-HIV5pep	0	2.18.13	4	R383	hIgG4-HIV5pep	0
2.18.13	1	R385	hIgG4	0	2.18.13	2	R384	hIgG4	0	2.18.13	3	R388	hIgG4	0	2.18.13	4	R383	hIgG4	5
2.18.13	1	R385	Cohesin-nef66 (C1077)	0	2.18.13	2	R384	Cohesin-nef66 (C1077)	0	2.18.13	3	R388	Cohesin-nef66 (C1077)	35	2.18.13	4	R383	Cohesin-nef66 (C1077)	0
2.18.13	1	R385	Cohesin-nef66 (C1078)	5	2.18.13	2	R384	Cohesin-nef66 (C1078)	0	2.18.13	3	R388	Cohesin-nef66 (C1078)	15	2.18.13	4	R383	Cohesin-nef66 (C1078)	0
2.18.13	1	R385	Cohesin-gag17	0	2.18.13	2	R384	Cohesin-gag17	15	2.18.13	3	R388	Cohesin-gag17	0	2.18.13	4	R383	Cohesin-gag17	0
2.18.13	1	R385	Cohesin-gag253	0	2.18.13	2	R384	Cohesin-gag253	0	2.18.13	3	R388	Cohesin-gag253	15	2.18.13	4	R383	Cohesin-gag253	0
2.18.13	1	R385	Cohesin–pol158	5	2.18.13	2	R384	Cohesin–pol158	0	2.18.13	3	R388	Cohesin–pol158	55	2.18.13	4	R383	Cohesin–pol158	0
2.18.13	1	R385	Cohesin	0	2.18.13	2	R384	Cohesin	0	2.18.13	3	R388	Cohesin	0	2.18.13	4	R383	Cohesin	0
2.18.13	1	R385	pha	1340	2.18.13	2	R384	pha	2315	2.18.13	3	R388	pha	3095	2.18.13	4	R383	pha	1745
3.4.13	1	R370	Non	0	3.4.13	2	R376	Non	0	3.4.13	3	R368	Non	0	3.4.13	4	R386	Non	0
3.4.13	1	R370	p24 pool 1	0	3.4.13	2	R376	p24 pool 1	30	3.4.13	3	R368	p24 pool 1	0	3.4.13	4	R386	p24 pool 1	15
3.4.13	1	R370	p24 pool 2	5	3.4.13	2	R376	p24 pool 2	0	3.4.13	3	R368	p24 pool 2	0	3.4.13	4	R386	p24 pool 2	5
3.4.13	1	R370	p24 pool 3	0	3.4.13	2	R376	p24 pool 3	0	3.4.13	3	R368	p24 pool 3	0	3.4.13	4	R386	p24 pool 3	20
3.4.13	1	R370	p24 pool 4	0	3.4.13	2	R376	p24 pool 4	5	3.4.13	3	R368	p24 pool 4	0	3.4.13	4	R386	p24 pool 4	15
3.4.13	1	R370	p24 pool 5	0	3.4.13	2	R376	p24 pool 5	0	3.4.13	3	R368	p24 pool 5	0	3.4.13	4	R386	p24 pool 5	10

3.4.13	1	R370	p2/p6/p7 pool 1	0
3.4.13	1	R370	p2/p6/p7 pool 2	0
3.4.13	1	R370	p2/p6/p7 pool 3	0
3.4.13	1	R370	p17 pool 1	0
3.4.13	1	R370	p17 pool 2	0
3.4.13	1	R370	p17 pool 3	0
3.4.13	1	R370	pol pool 1	0
3.4.13	1	R370	pol pool 2	0
3.4.13	1	R370	pol pool 3	0
3.4.13	1	R370	pol pool 4	0
3.4.13	1	R370	nef pool 1	0
3.4.13	1	R370	nef pool 2	0
3.4.13	1	R370	nef pool 3	0
3.4.13	1	R370	hIgG4-HIV5pep	0
3.4.13	1	R370	hIgG4	0
3.4.13	1	R370	Cohesin-nef66 (C1077)	0
3.4.13	1	R370	Cohesin-nef66 (C1078)	0
3.4.13	1	R370	Cohesin-gag17	0
3.4.13	1	R370	Cohesin-gag253	0
3.4.13	1	R370	Cohesin–pol158	0
3.4.13	1	R370	Cohesin	5
3.4.13	1	R370	pha	2200
3.4.13	1	R371	Non	0
3.4.13	1	R371	p24 pool 1	0
3.4.13	1	R371	p24 pool 2	5
3.4.13	1	R371	p24 pool 3	10
3.4.13	1	R371	p24 pool 4	5
3.4.13	1	R371	p24 pool 5	0
3.4.13	1	R371	p2/p6/p7 pool 1	0
3.4.13	1	R371	p2/p6/p7 pool 2	0
3.4.13	1	R371	p2/p6/p7 pool 3	0
3.4.13	1	R371	p17 pool 1	0
3.4.13	1	R371	p17 pool 2	0
3.4.13	1	R371	p17 pool 3	0
3.4.13	1	R371	pol pool 1	0
3.4.13	1	R371	pol pool 2	5
3.4.13	1	R371	pol pool 3	0
3.4.13	1	R371	pol pool 4	0
3.4.13	1	R371	nef pool 1	0
3.4.13	1	R371	nef pool 2	0
3.4.13	1	R371	nef pool 3	25
3.4.13	1	R371	hIgG4-HIV5pep	10
3.4.13	1	R371	hIgG4	0
3.4.13	1	R371	Cohesin-nef66 (C1077)	0
3.4.13	1	R371	Cohesin-nef66 (C1078)	0
3.4.13	1	R371	Cohesin-gag17	0
3.4.13	1	R371	Cohesin-gag253	15
3.4.13	1	R371	Cohesin–pol158	0
3.4.13	1	R371	Cohesin	0
3.4.13	1	R371	pha	3235
3.4.13	1	R373	Non	0
3.4.13	1	R373	p24 pool 1	0
3.4.13	1	R373	p24 pool 2	0
3.4.13	1	R373	p24 pool 3	0
3.4.13	1	R373	p24 pool 4	0
3.4.13	1	R373	p24 pool 5	10
3.4.13	1	R373	p2/p6/p7 pool 1	5
3.4.13	1	R373	p2/p6/p7 pool 2	0
3.4.13	1	R373	p2/p6/p7 pool 3	0
3.4.13	1	R373	p17 pool 1	0
3.4.13	1	R373	p17 pool 2	0
3.4.13	1	R373	p17 pool 3	0
3.4.13	1	R373	pol pool 1	0
3.4.13	1	R373	pol pool 2	0
3.4.13	1	R373	pol pool 3	0
3.4.13	1	R373	pol pool 4	0
3.4.13	1	R373	nef pool 1	0
3.4.13	1	R373	nef pool 2	0
3.4.13	1	R373	nef pool 3	0
3.4.13	1	R373	hIgG4-HIV5pep	0
3.4.13	1	R373	hIgG4	0
3.4.13	1	R373	Cohesin-nef66 (C1077)	0
3.4.13	1	R373	Cohesin-nef66 (C1078)	0
3.4.13	1	R373	Cohesin-gag17	0
3.4.13	1	R373	Cohesin-gag253	0
3.4.13	1	R373	Cohesin–pol158	5
3.4.13	1	R373	Cohesin	0
3.4.13	1	R373	pha	2050
3.4.13	1	R374	Non	0
3.4.13	1	R374	p24 pool 1	0
3.4.13	1	R374	p24 pool 2	15
3.4.13	1	R374	p24 pool 3	0
3.4.13	1	R374	p24 pool 4	0
3.4.13	1	R374	p24 pool 5	0
3.4.13	1	R374	p2/p6/p7 pool 1	5
3.4.13	1	R374	p2/p6/p7 pool 2	5
3.4.13	1	R374	p2/p6/p7 pool 3	0
3.4.13	1	R374	p17 pool 1	0

3.4.13	2	R376	p2/p6/p7 pool 1	0
3.4.13	2	R376	p2/p6/p7 pool 2	0
3.4.13	2	R376	p2/p6/p7 pool 3	0
3.4.13	2	R376	p17 pool 1	0
3.4.13	2	R376	p17 pool 2	0
3.4.13	2	R376	p17 pool 3	0
3.4.13	2	R376	pol pool 1	0
3.4.13	2	R376	pol pool 2	10
3.4.13	2	R376	pol pool 3	5
3.4.13	2	R376	pol pool 4	5
3.4.13	2	R376	nef pool 1	0
3.4.13	2	R376	nef pool 2	5
3.4.13	2	R376	nef pool 3	0
3.4.13	2	R376	hIgG4-HIV5pep	0
3.4.13	2	R376	hIgG4	0
3.4.13	2	R376	Cohesin-nef66 (C1077)	0
3.4.13	2	R376	Cohesin-nef66 (C1078)	0
3.4.13	2	R376	Cohesin-gag17	0
3.4.13	2	R376	Cohesin-gag253	5
3.4.13	2	R376	Cohesin–pol158	5
3.4.13	2	R376	Cohesin	0
3.4.13	2	R376	pha	2600
3.4.13	2	R377	Non	0
3.4.13	2	R377	p24 pool 1	0
3.4.13	2	R377	p24 pool 2	0
3.4.13	2	R377	p24 pool 3	0
3.4.13	2	R377	p24 pool 4	0
3.4.13	2	R377	p24 pool 5	0
3.4.13	2	R377	p2/p6/p7 pool 1	20
3.4.13	2	R377	p2/p6/p7 pool 2	0
3.4.13	2	R377	p2/p6/p7 pool 3	0
3.4.13	2	R377	p17 pool 1	0
3.4.13	2	R377	p17 pool 2	0
3.4.13	2	R377	p17 pool 3	0
3.4.13	2	R377	pol pool 1	0
3.4.13	2	R377	pol pool 2	125
3.4.13	2	R377	pol pool 3	15
3.4.13	2	R377	pol pool 4	0
3.4.13	2	R377	nef pool 1	0
3.4.13	2	R377	nef pool 2	10
3.4.13	2	R377	nef pool 3	135
3.4.13	2	R377	hIgG4-HIV5pep	0
3.4.13	2	R377	hIgG4	0
3.4.13	2	R377	Cohesin-nef66 (C1077)	0
3.4.13	2	R377	Cohesin-nef66 (C1078)	0
3.4.13	2	R377	Cohesin-gag17	0
3.4.13	2	R377	Cohesin-gag253	0
3.4.13	2	R377	Cohesin–pol158	0
3.4.13	2	R377	Cohesin	0
3.4.13	2	R377	pha	3315
3.4.13	2	R378	Non	0
3.4.13	2	R378	p24 pool 1	0
3.4.13	2	R378	p24 pool 2	0
3.4.13	2	R378	p24 pool 3	0
3.4.13	2	R378	p24 pool 4	0
3.4.13	2	R378	p24 pool 5	0
3.4.13	2	R378	p2/p6/p7 pool 1	0
3.4.13	2	R378	p2/p6/p7 pool 2	0
3.4.13	2	R378	p2/p6/p7 pool 3	0
3.4.13	2	R378	p17 pool 1	0
3.4.13	2	R378	p17 pool 2	0
3.4.13	2	R378	p17 pool 3	0
3.4.13	2	R378	pol pool 1	0
3.4.13	2	R378	pol pool 2	0
3.4.13	2	R378	pol pool 3	0
3.4.13	2	R378	pol pool 4	0
3.4.13	2	R378	nef pool 1	0
3.4.13	2	R378	nef pool 2	0
3.4.13	2	R378	nef pool 3	0
3.4.13	2	R378	hIgG4-HIV5pep	0
3.4.13	2	R378	hIgG4	0
3.4.13	2	R378	Cohesin-nef66 (C1077)	0
3.4.13	2	R378	Cohesin-nef66 (C1078)	5
3.4.13	2	R378	Cohesin-gag17	5
3.4.13	2	R378	Cohesin-gag253	5
3.4.13	2	R378	Cohesin–pol158	25
3.4.13	2	R378	Cohesin	0
3.4.13	2	R378	pha	3055
3.4.13	2	R380	Non	0
3.4.13	2	R380	p24 pool 1	0
3.4.13	2	R380	p24 pool 2	0
3.4.13	2	R380	p24 pool 3	0
3.4.13	2	R380	p24 pool 4	0
3.4.13	2	R380	p24 pool 5	0
3.4.13	2	R380	p2/p6/p7 pool 1	0
3.4.13	2	R380	p2/p6/p7 pool 2	0
3.4.13	2	R380	p2/p6/p7 pool 3	0
3.4.13	2	R380	p17 pool 1	0

3.4.13	3	R368	p2/p6/p7 pool 1	0
3.4.13	3	R368	p2/p6/p7 pool 2	0
3.4.13	3	R368	p2/p6/p7 pool 3	0
3.4.13	3	R368	p17 pool 1	0
3.4.13	3	R368	p17 pool 2	0
3.4.13	3	R368	p17 pool 3	0
3.4.13	3	R368	pol pool 1	0
3.4.13	3	R368	pol pool 2	0
3.4.13	3	R368	pol pool 3	0
3.4.13	3	R368	pol pool 4	0
3.4.13	3	R368	nef pool 1	0
3.4.13	3	R368	nef pool 2	0
3.4.13	3	R368	nef pool 3	0
3.4.13	3	R368	hIgG4-HIV5pep	0
3.4.13	3	R368	hIgG4	0
3.4.13	3	R368	Cohesin-nef66 (C1077)	0
3.4.13	3	R368	Cohesin-nef66 (C1078)	5
3.4.13	3	R368	Cohesin-gag17	0
3.4.13	3	R368	Cohesin-gag253	0
3.4.13	3	R368	Cohesin–pol158	0
3.4.13	3	R368	Cohesin	0
3.4.13	3	R368	pha	2540
3.4.13	3	R375	Non	0
3.4.13	3	R375	p24 pool 1	10
3.4.13	3	R375	p24 pool 2	0
3.4.13	3	R375	p24 pool 3	0
3.4.13	3	R375	p24 pool 4	10
3.4.13	3	R375	p24 pool 5	0
3.4.13	3	R375	p2/p6/p7 pool 1	0
3.4.13	3	R375	p2/p6/p7 pool 2	5
3.4.13	3	R375	p2/p6/p7 pool 3	0
3.4.13	3	R375	p17 pool 1	0
3.4.13	3	R375	p17 pool 2	10
3.4.13	3	R375	p17 pool 3	0
3.4.13	3	R375	pol pool 1	5
3.4.13	3	R375	pol pool 2	25
3.4.13	3	R375	pol pool 3	15
3.4.13	3	R375	pol pool 4	0
3.4.13	3	R375	nef pool 1	5
3.4.13	3	R375	nef pool 2	10
3.4.13	3	R375	nef pool 3	0
3.4.13	3	R375	hIgG4-HIV5pep	5
3.4.13	3	R375	hIgG4	20
3.4.13	3	R375	Cohesin-nef66 (C1077)	0
3.4.13	3	R375	Cohesin-nef66 (C1078)	15
3.4.13	3	R375	Cohesin-gag17	20
3.4.13	3	R375	Cohesin-gag253	0
3.4.13	3	R375	Cohesin–pol158	5
3.4.13	3	R375	Cohesin	5
3.4.13	3	R375	pha	2080
3.4.13	3	R379	Non	0
3.4.13	3	R379	p24 pool 1	0
3.4.13	3	R379	p24 pool 2	0
3.4.13	3	R379	p24 pool 3	10
3.4.13	3	R379	p24 pool 4	0
3.4.13	3	R379	p24 pool 5	0
3.4.13	3	R379	p2/p6/p7 pool 1	0
3.4.13	3	R379	p2/p6/p7 pool 2	0
3.4.13	3	R379	p2/p6/p7 pool 3	10
3.4.13	3	R379	p17 pool 1	0
3.4.13	3	R379	p17 pool 2	5
3.4.13	3	R379	p17 pool 3	0
3.4.13	3	R379	pol pool 1	0
3.4.13	3	R379	pol pool 2	5
3.4.13	3	R379	pol pool 3	5
3.4.13	3	R379	pol pool 4	0
3.4.13	3	R379	nef pool 1	0
3.4.13	3	R379	nef pool 2	0
3.4.13	3	R379	nef pool 3	0
3.4.13	3	R379	hIgG4-HIV5pep	0
3.4.13	3	R379	hIgG4	0
3.4.13	3	R379	Cohesin-nef66 (C1077)	5
3.4.13	3	R379	Cohesin-nef66 (C1078)	5
3.4.13	3	R379	Cohesin-gag17	5
3.4.13	3	R379	Cohesin-gag253	0
3.4.13	3	R379	Cohesin–pol158	5
3.4.13	3	R379	Cohesin	5
3.4.13	3	R379	pha	2055
3.4.13	3	R381	Non	0
3.4.13	3	R381	p24 pool 1	5
3.4.13	3	R381	p24 pool 2	5
3.4.13	3	R381	p24 pool 3	5
3.4.13	3	R381	p24 pool 4	5
3.4.13	3	R381	p24 pool 5	0
3.4.13	3	R381	p2/p6/p7 pool 1	5
3.4.13	3	R381	p2/p6/p7 pool 2	0
3.4.13	3	R381	p2/p6/p7 pool 3	5
3.4.13	3	R381	p17 pool 1	10

3.4.13	4	R386	p2/p6/p7 pool 1	5
3.4.13	4	R386	p2/p6/p7 pool 2	5
3.4.13	4	R386	p2/p6/p7 pool 3	5
3.4.13	4	R386	p17 pool 1	15
3.4.13	4	R386	p17 pool 2	5
3.4.13	4	R386	p17 pool 3	10
3.4.13	4	R386	pol pool 1	20
3.4.13	4	R386	pol pool 2	25
3.4.13	4	R386	pol pool 3	0
3.4.13	4	R386	pol pool 4	5
3.4.13	4	R386	nef pool 1	10
3.4.13	4	R386	nef pool 2	15
3.4.13	4	R386	nef pool 3	5
3.4.13	4	R386	hIgG4-HIV5pep	10
3.4.13	4	R386	hIgG4	30
3.4.13	4	R386	Cohesin-nef66 (C1077)	10
3.4.13	4	R386	Cohesin-nef66 (C1078)	10
3.4.13	4	R386	Cohesin-gag17	25
3.4.13	4	R386	Cohesin-gag253	10
3.4.13	4	R386	Cohesin–pol158	5
3.4.13	4	R386	Cohesin	10
3.4.13	4	R386	pha	TNTC
3.4.13	4	R389	Non	0
3.4.13	4	R389	p24 pool 1	0
3.4.13	4	R389	p24 pool 2	0
3.4.13	4	R389	p24 pool 3	10
3.4.13	4	R389	p24 pool 4	15
3.4.13	4	R389	p24 pool 5	15
3.4.13	4	R389	p2/p6/p7 pool 1	0
3.4.13	4	R389	p2/p6/p7 pool 2	0
3.4.13	4	R389	p2/p6/p7 pool 3	15
3.4.13	4	R389	p17 pool 1	0
3.4.13	4	R389	p17 pool 2	5
3.4.13	4	R389	p17 pool 3	0
3.4.13	4	R389	pol pool 1	15
3.4.13	4	R389	pol pool 2	65
3.4.13	4	R389	pol pool 3	0
3.4.13	4	R389	pol pool 4	0
3.4.13	4	R389	nef pool 1	5
3.4.13	4	R389	nef pool 2	10
3.4.13	4	R389	nef pool 3	0
3.4.13	4	R389	hIgG4-HIV5pep	30
3.4.13	4	R389	hIgG4	25
3.4.13	4	R389	Cohesin-nef66 (C1077)	0
3.4.13	4	R389	Cohesin-nef66 (C1078)	35
3.4.13	4	R389	Cohesin-gag17	15
3.4.13	4	R389	Cohesin-gag253	20
3.4.13	4	R389	Cohesin–pol158	0
3.4.13	4	R389	Cohesin	5
3.4.13	4	R389	pha	TNTC
3.4.13	4	R390	Non	0
3.4.13	4	R390	p24 pool 1	0
3.4.13	4	R390	p24 pool 2	0
3.4.13	4	R390	p24 pool 3	5
3.4.13	4	R390	p24 pool 4	0
3.4.13	4	R390	p24 pool 5	0
3.4.13	4	R390	p2/p6/p7 pool 1	5
3.4.13	4	R390	p2/p6/p7 pool 2	0
3.4.13	4	R390	p2/p6/p7 pool 3	0
3.4.13	4	R390	p17 pool 1	10
3.4.13	4	R390	p17 pool 2	5
3.4.13	4	R390	p17 pool 3	10
3.4.13	4	R390	pol pool 1	15
3.4.13	4	R390	pol pool 2	20
3.4.13	4	R390	pol pool 3	5
3.4.13	4	R390	pol pool 4	5
3.4.13	4	R390	nef pool 1	0
3.4.13	4	R390	nef pool 2	0
3.4.13	4	R390	nef pool 3	0
3.4.13	4	R390	hIgG4-HIV5pep	10
3.4.13	4	R390	hIgG4	10
3.4.13	4	R390	Cohesin-nef66 (C1077)	10
3.4.13	4	R390	Cohesin-nef66 (C1078)	10
3.4.13	4	R390	Cohesin-gag17	0
3.4.13	4	R390	Cohesin-gag253	40
3.4.13	4	R390	Cohesin–pol158	10
3.4.13	4	R390	Cohesin	0
3.4.13	4	R390	pha	TNTC
3.4.13	4	R391	Non	0
3.4.13	4	R391	p24 pool 1	15
3.4.13	4	R391	p24 pool 2	0
3.4.13	4	R391	p24 pool 3	35
3.4.13	4	R391	p24 pool 4	25
3.4.13	4	R391	p24 pool 5	25
3.4.13	4	R391	p2/p6/p7 pool 1	20
3.4.13	4	R391	p2/p6/p7 pool 2	0
3.4.13	4	R391	p2/p6/p7 pool 3	20
3.4.13	4	R391	p17 pool 1	20

3.4.13	1	R374	p17 pool 2	10	3.4.13	2	R380	p17 pool 2	0	3.4.13	3	R381	p17 pool 2	10	3.4.13	4	R391	p17 pool 2	15
3.4.13	1	R374	p17 pool 3	10	3.4.13	2	R380	p17 pool 3	0	3.4.13	3	R381	p17 pool 3	5	3.4.13	4	R391	p17 pool 3	15
3.4.13	1	R374	pol pool 1	0	3.4.13	2	R380	pol pool 1	0	3.4.13	3	R381	pol pool 1	0	3.4.13	4	R391	pol pool 1	45
3.4.13	1	R374	pol pool 2	5	3.4.13	2	R380	pol pool 2	5	3.4.13	3	R381	pol pool 2	30	3.4.13	4	R391	pol pool 2	215
3.4.13	1	R374	pol pool 3	5	3.4.13	2	R380	pol pool 3	0	3.4.13	3	R381	pol pool 3	0	3.4.13	4	R391	pol pool 3	25
3.4.13	1	R374	pol pool 4	0	3.4.13	2	R380	pol pool 4	0	3.4.13	3	R381	pol pool 4	10	3.4.13	4	R391	pol pool 4	15
3.4.13	1	R374	nef pool 1	0	3.4.13	2	R380	nef pool 1	0	3.4.13	3	R381	nef pool 1	5	3.4.13	4	R391	nef pool 1	25
3.4.13	1	R374	nef pool 2	0	3.4.13	2	R380	nef pool 2	0	3.4.13	3	R381	nef pool 2	5	3.4.13	4	R391	nef pool 2	0
3.4.13	1	R374	nef pool 3	20	3.4.13	2	R380	nef pool 3	5	3.4.13	3	R381	nef pool 3	5	3.4.13	4	R391	nef pool 3	45
3.4.13	1	R374	hIgG4-HIV5pep	10	3.4.13	2	R380	hIgG4-HIV5pep	0	3.4.13	3	R381	hIgG4-HIV5pep	10	3.4.13	4	R391	hIgG4-HIV5pep	35
3.4.13	1	R374	hIgG4	0	3.4.13	2	R380	hIgG4	5	3.4.13	3	R381	hIgG4	5	3.4.13	4	R391	hIgG4	40
3.4.13	1	R374	Cohesin-nef66 (C1077)	0	3.4.13	2	R380	Cohesin-nef66 (C1077)	0	3.4.13	3	R381	Cohesin-nef66 (C1077)	5	3.4.13	4	R391	Cohesin-nef66 (C1077)	85
3.4.13	1	R374	Cohesin-nef66 (C1078)	10	3.4.13	2	R380	Cohesin-nef66 (C1078)	15	3.4.13	3	R381	Cohesin-nef66 (C1078)	10	3.4.13	4	R391	Cohesin-nef66 (C1078)	80
3.4.13	1	R374	Cohesin-gag17	5	3.4.13	2	R380	Cohesin-gag17	0	3.4.13	3	R381	Cohesin-gag17	5	3.4.13	4	R391	Cohesin-gag17	80
3.4.13	1	R374	Cohesin-gag253	5	3.4.13	2	R380	Cohesin-gag253	10	3.4.13	3	R381	Cohesin-gag253	0	3.4.13	4	R391	Cohesin-gag253	70
3.4.13	1	R374	Cohesin–pol158	0	3.4.13	2	R380	Cohesin–pol158	0	3.4.13	3	R381	Cohesin–pol158	5	3.4.13	4	R391	Cohesin–pol158	5
3.4.13	1	R374	Cohesin	0	3.4.13	2	R380	Cohesin	5	3.4.13	3	R381	Cohesin	5	3.4.13	4	R391	Cohesin	25
3.4.13	1	R374	pha	3310	3.4.13	2	R380	pha	2095	3.4.13	3	R381	pha	2245	3.4.13	4	R391	pha	TNTC
3.4.13	1	R369	Non	0	3.4.13	2	R382	Non	0	3.4.13	3	R387	Non	0	3.4.13	4	R372	Non	0
3.4.13	1	R369	p24 pool 1	20	3.4.13	2	R382	p24 pool 1	10	3.4.13	3	R387	p24 pool 1	0	3.4.13	4	R372	p24 pool 1	0
3.4.13	1	R369	p24 pool 2	55	3.4.13	2	R382	p24 pool 2	15	3.4.13	3	R387	p24 pool 2	0	3.4.13	4	R372	p24 pool 2	0
3.4.13	1	R369	p24 pool 3	15	3.4.13	2	R382	p24 pool 3	0	3.4.13	3	R387	p24 pool 3	5	3.4.13	4	R372	p24 pool 3	0
3.4.13	1	R369	p24 pool 4	5	3.4.13	2	R382	p24 pool 4	5	3.4.13	3	R387	p24 pool 4	0	3.4.13	4	R372	p24 pool 4	0
3.4.13	1	R369	p24 pool 5	10	3.4.13	2	R382	p24 pool 5	25	3.4.13	3	R387	p24 pool 5	0	3.4.13	4	R372	p24 pool 5	0
3.4.13	1	R369	p2/p6/p7 pool 1	20	3.4.13	2	R382	p2/p6/p7 pool 1	15	3.4.13	3	R387	p2/p6/p7 pool 1	0	3.4.13	4	R372	p2/p6/p7 pool 1	0
3.4.13	1	R369	p2/p6/p7 pool 2	5	3.4.13	2	R382	p2/p6/p7 pool 2	5	3.4.13	3	R387	p2/p6/p7 pool 2	0	3.4.13	4	R372	p2/p6/p7 pool 2	0
3.4.13	1	R369	p2/p6/p7 pool 3	10	3.4.13	2	R382	p2/p6/p7 pool 3	5	3.4.13	3	R387	p2/p6/p7 pool 3	0	3.4.13	4	R372	p2/p6/p7 pool 3	0
3.4.13	1	R369	p17 pool 1	10	3.4.13	2	R382	p17 pool 1	5	3.4.13	3	R387	p17 pool 1	0	3.4.13	4	R372	p17 pool 1	0
3.4.13	1	R369	p17 pool 2	15	3.4.13	2	R382	p17 pool 2	0	3.4.13	3	R387	p17 pool 2	0	3.4.13	4	R372	p17 pool 2	5
3.4.13	1	R369	p17 pool 3	5	3.4.13	2	R382	p17 pool 3	5	3.4.13	3	R387	p17 pool 3	0	3.4.13	4	R372	p17 pool 3	0
3.4.13	1	R369	pol pool 1	15	3.4.13	2	R382	pol pool 1	10	3.4.13	3	R387	pol pool 1	0	3.4.13	4	R372	pol pool 1	5
3.4.13	1	R369	pol pool 2	20	3.4.13	2	R382	pol pool 2	40	3.4.13	3	R387	pol pool 2	5	3.4.13	4	R372	pol pool 2	20
3.4.13	1	R369	pol pool 3	10	3.4.13	2	R382	pol pool 3	50	3.4.13	3	R387	pol pool 3	0	3.4.13	4	R372	pol pool 3	0
3.4.13	1	R369	pol pool 4	10	3.4.13	2	R382	pol pool 4	10	3.4.13	3	R387	pol pool 4	0	3.4.13	4	R372	pol pool 4	0
3.4.13	1	R369	nef pool 1	5	3.4.13	2	R382	nef pool 1	20	3.4.13	3	R387	nef pool 1	0	3.4.13	4	R372	nef pool 1	0
3.4.13	1	R369	nef pool 2	20	3.4.13	2	R382	nef pool 2	0	3.4.13	3	R387	nef pool 2	0	3.4.13	4	R372	nef pool 2	0
3.4.13	1	R369	nef pool 3	40	3.4.13	2	R382	nef pool 3	45	3.4.13	3	R387	nef pool 3	0	3.4.13	4	R372	nef pool 3	0
3.4.13	1	R369	hIgG4-HIV5pep	25	3.4.13	2	R382	hIgG4-HIV5pep	0	3.4.13	3	R387	hIgG4-HIV5pep	5	3.4.13	4	R372	hIgG4-HIV5pep	0
3.4.13	1	R369	hIgG4	0	3.4.13	2	R382	hIgG4	0	3.4.13	3	R387	hIgG4	0	3.4.13	4	R372	hIgG4	0
3.4.13	1	R369	Cohesin-nef66 (C1077)	5	3.4.13	2	R382	Cohesin-nef66 (C1077)	5	3.4.13	3	R387	Cohesin-nef66 (C1077)	5	3.4.13	4	R372	Cohesin-nef66 (C1077)	0
3.4.13	1	R369	Cohesin-nef66 (C1078)	15	3.4.13	2	R382	Cohesin-nef66 (C1078)	0	3.4.13	3	R387	Cohesin-nef66 (C1078)	0	3.4.13	4	R372	Cohesin-nef66 (C1078)	20
3.4.13	1	R369	Cohesin-gag17	20	3.4.13	2	R382	Cohesin-gag17	15	3.4.13	3	R387	Cohesin-gag17	0	3.4.13	4	R372	Cohesin-gag17	0
3.4.13	1	R369	Cohesin-gag253	20	3.4.13	2	R382	Cohesin-gag253	10	3.4.13	3	R387	Cohesin-gag253	5	3.4.13	4	R372	Cohesin-gag253	5
3.4.13	1	R369	Cohesin–pol158	20	3.4.13	2	R382	Cohesin–pol158	20	3.4.13	3	R387	Cohesin–pol158	5	3.4.13	4	R372	Cohesin–pol158	0
3.4.13	1	R369	Cohesin	30	3.4.13	2	R382	Cohesin	5	3.4.13	3	R387	Cohesin	0	3.4.13	4	R372	Cohesin	0
3.4.13	1	R369	pha	2875	3.4.13	2	R382	pha	3105	3.4.13	3	R387	pha	TNTC	3.4.13	4	R372	pha	2325
3.4.13	1	R385	Non	0	3.4.13	2	R384	Non	0	3.4.13	3	R388	Non	0	3.4.13	4	R383	Non	0
3.4.13	1	R385	p24 pool 1	0	3.4.13	2	R384	p24 pool 1	0	3.4.13	3	R388	p24 pool 1	0	3.4.13	4	R383	p24 pool 1	5
3.4.13	1	R385	p24 pool 2	5	3.4.13	2	R384	p24 pool 2	10	3.4.13	3	R388	p24 pool 2	0	3.4.13	4	R383	p24 pool 2	0
3.4.13	1	R385	p24 pool 3	0	3.4.13	2	R384	p24 pool 3	0	3.4.13	3	R388	p24 pool 3	15	3.4.13	4	R383	p24 pool 3	15
3.4.13	1	R385	p24 pool 4	0	3.4.13	2	R384	p24 pool 4	0	3.4.13	3	R388	p24 pool 4	0	3.4.13	4	R383	p24 pool 4	0
3.4.13	1	R385	p24 pool 5	0	3.4.13	2	R384	p24 pool 5	0	3.4.13	3	R388	p24 pool 5	0	3.4.13	4	R383	p24 pool 5	0
3.4.13	1	R385	p2/p6/p7 pool 1	0	3.4.13	2	R384	p2/p6/p7 pool 1	0	3.4.13	3	R388	p2/p6/p7 pool 1	5	3.4.13	4	R383	p2/p6/p7 pool 1	5
3.4.13	1	R385	p2/p6/p7 pool 2	0	3.4.13	2	R384	p2/p6/p7 pool 2	5	3.4.13	3	R388	p2/p6/p7 pool 2	0	3.4.13	4	R383	p2/p6/p7 pool 2	0
3.4.13	1	R385	p2/p6/p7 pool 3	0	3.4.13	2	R384	p2/p6/p7 pool 3	0	3.4.13	3	R388	p2/p6/p7 pool 3	0	3.4.13	4	R383	p2/p6/p7 pool 3	0
3.4.13	1	R385	p17 pool 1	0	3.4.13	2	R384	p17 pool 1	15	3.4.13	3	R388	p17 pool 1	0	3.4.13	4	R383	p17 pool 1	5
3.4.13	1	R385	p17 pool 2	0	3.4.13	2	R384	p17 pool 2	5	3.4.13	3	R388	p17 pool 2	0	3.4.13	4	R383	p17 pool 2	0
3.4.13	1	R385	p17 pool 3	0	3.4.13	2	R384	p17 pool 3	0	3.4.13	3	R388	p17 pool 3	0	3.4.13	4	R383	p17 pool 3	0
3.4.13	1	R385	pol pool 1	0	3.4.13	2	R384	pol pool 1	0	3.4.13	3	R388	pol pool 1	0	3.4.13	4	R383	pol pool 1	5
3.4.13	1	R385	pol pool 2	25	3.4.13	2	R384	pol pool 2	5	3.4.13	3	R388	pol pool 2	30	3.4.13	4	R383	pol pool 2	10
3.4.13	1	R385	pol pool 3	0	3.4.13	2	R384	pol pool 3	0	3.4.13	3	R388	pol pool 3	0	3.4.13	4	R383	pol pool 3	5
3.4.13	1	R385	pol pool 4	0	3.4.13	2	R384	pol pool 4	5	3.4.13	3	R388	pol pool 4	0	3.4.13	4	R383	pol pool 4	5
3.4.13	1	R385	nef pool 1	0	3.4.13	2	R384	nef pool 1	0	3.4.13	3	R388	nef pool 1	0	3.4.13	4	R383	nef pool 1	5
3.4.13	1	R385	nef pool 2	0	3.4.13	2	R384	nef pool 2	5	3.4.13	3	R388	nef pool 2	0	3.4.13	4	R383	nef pool 2	0
3.4.13	1	R385	nef pool 3	0	3.4.13	2	R384	nef pool 3	15	3.4.13	3	R388	nef pool 3	0	3.4.13	4	R383	nef pool 3	0
3.4.13	1	R385	hIgG4-HIV5pep	0	3.4.13	2	R384	hIgG4-HIV5pep	5	3.4.13	3	R388	hIgG4-HIV5pep	20	3.4.13	4	R383	hIgG4-HIV5pep	0
3.4.13	1	R385	hIgG4	0	3.4.13	2	R384	hIgG4	0	3.4.13	3	R388	hIgG4	0	3.4.13	4	R383	hIgG4	5
3.4.13	1	R385	Cohesin-nef66 (C1077)	0	3.4.13	2	R384	Cohesin-nef66 (C1077)	0	3.4.13	3	R388	Cohesin-nef66 (C1077)	15	3.4.13	4	R383	Cohesin-nef66 (C1077)	5
3.4.13	1	R385	Cohesin-nef66 (C1078)	0	3.4.13	2	R384	Cohesin-nef66 (C1078)	5	3.4.13	3	R388	Cohesin-nef66 (C1078)	85	3.4.13	4	R383	Cohesin-nef66 (C1078)	0
3.4.13	1	R385	Cohesin-gag17	0	3.4.13	2	R384	Cohesin-gag17	0	3.4.13	3	R388	Cohesin-gag17	10	3.4.13	4	R383	Cohesin-gag17	0
3.4.13	1	R385	Cohesin-gag253	0	3.4.13	2	R384	Cohesin-gag253	15	3.4.13	3	R388	Cohesin-gag253	35	3.4.13	4	R383	Cohesin-gag253	10
3.4.13	1	R385	Cohesin–pol158	0	3.4.13	2	R384	Cohesin–pol158	0	3.4.13	3	R388	Cohesin–pol158	55	3.4.13	4	R383	Cohesin–pol158	0
3.4.13	1	R385	Cohesin	0	3.4.13	2	R384	Cohesin	0	3.4.13	3	R388	Cohesin	0	3.4.13	4	R383	Cohesin	0
3.4.13	1	R385	pha	2420	3.4.13	2	R384	pha	2745	3.4.13	3	R388	pha	TNTC	3.4.13	4	R383	pha	2050
4.29.13	1	R370	Non	0	4.29.13	2	R376	Non	0	4.2.13	3	R368	Non	0	4.2.13	4	R386	Non	0
4.29.13	1	R370	p24 pool 1	5	4.29.13	2	R376	p24 pool 1	140	4.2.13	3	R368	p24 pool 1	0	4.2.13	4	R386	p24 pool 1	5
4.29.13	1	R370	p24 pool 2	5	4.29.13	2	R376	p24 pool 2	0	4.2.13	3	R368	p24 pool 2	0	4.2.13	4	R386	p24 pool 2	5
4.29.13	1	R370	p24 pool 3	0	4.29.13	2	R376	p24 pool 3	0	4.2.13	3	R368	p24 pool 3	0	4.2.13	4	R386	p24 pool 3	30
4.29.13	1	R370	p24 pool 4	0	4.29.13	2	R376	p24 pool 4	0	4.2.13	3	R368	p24 pool 4	10	4.2.13	4	R386	p24 pool 4	15
4.29.13	1	R370	p24 pool 5	0	4.29.13	2	R376	p24 pool 5	0	4.2.13	3	R368	p24 pool 5	0	4.2.13	4	R386	p24 pool 5	0
4.29.13	1	R370	p2/p6/p7 pool 1	0	4.29.13	2	R376	p2/p6/p7 pool 1	0	4.2.13	3	R368	p2/p6/p7 pool 1	0	4.2.13	4	R386	p2/p6/p7 pool 1	10
4.29.13	1	R370	p2/p6/p7 pool 2	0	4.29.13	2	R376	p2/p6/p7 pool 2	0	4.2.13	3	R368	p2/p6/p7 pool 2	5	4.2.13	4	R386	p2/p6/p7 pool 2	0
4.29.13	1	R370	p2/p6/p7 pool 3	0	4.29.13	2	R376	p2/p6/p7 pool 3	0	4.2.13	3	R368	p2/p6/p7 pool 3	0	4.2.13	4	R386	p2/p6/p7 pool 3	5
4.29.13	1	R370	p17 pool 1	0	4.29.13	2	R376	p17 pool 1	0	4.2.13	3	R368	p17 pool 1	0	4.2.13	4	R386	p17 pool 1	10
4.29.13	1	R370	p17 pool 2	0	4.29.13	2	R376	p17 pool 2	0	4.2.13	3	R368	p17 pool 2	0	4.2.13	4	R386	p17 pool 2	10
4.29.13	1	R370	p17 pool 3	0	4.29.13	2	R376	p17 pool 3	0	4.2.13	3	R368	p17 pool 3	0	4.2.13	4	R386	p17 pool 3	10
4.29.13	1	R370	pol pool 1	5	4.29.13	2	R376	pol pool 1	0	4.2.13	3	R368	pol pool 1	0	4.2.13	4	R386	pol pool 1	5

4.29.13	1	R370	pol pool 2	5	4.29.13	2	R376	pol pool 2	0	4.2.13	3	R368	pol pool 2	5	4.2.13	4	R386	pol pool 2	20
4.29.13	1	R370	pol pool 3	0	4.29.13	2	R376	pol pool 3	0	4.2.13	3	R368	pol pool 3	30	4.2.13	4	R386	pol pool 3	0
4.29.13	1	R370	pol pool 4	0	4.29.13	2	R376	pol pool 4	0	4.2.13	3	R368	pol pool 4	0	4.2.13	4	R386	pol pool 4	0
4.29.13	1	R370	nef pool 1	0	4.29.13	2	R376	nef pool 1	0	4.2.13	3	R368	nef pool 1	0	4.2.13	4	R386	nef pool 1	0
4.29.13	1	R370	nef pool 2	0	4.29.13	2	R376	nef pool 2	0	4.2.13	3	R368	nef pool 2	15	4.2.13	4	R386	nef pool 2	0
4.29.13	1	R370	nef pool 3	10	4.29.13	2	R376	nef pool 3	0	4.2.13	3	R368	nef pool 3	0	4.2.13	4	R386	nef pool 3	5
4.29.13	1	R370	hIgG4-HIV5pep	0	4.29.13	2	R376	hIgG4-HIV5pep	0	4.2.13	3	R368	hIgG4-HIV5pep	5	4.2.13	4	R386	hIgG4-HIV5pep	10
4.29.13	1	R370	hIgG4	0	4.29.13	2	R376	hIgG4	0	4.2.13	3	R368	hIgG4	0	4.2.13	4	R386	hIgG4	5
4.29.13	1	R370	Cohesin-nef66 (C1077)	0	4.29.13	2	R376	Cohesin-nef66 (C1077)	0	4.2.13	3	R368	Cohesin-nef66 (C1077)	0	4.2.13	4	R386	Cohesin-nef66 (C1077)	10
4.29.13	1	R370	Cohesin-nef66 (C1078)	0	4.29.13	2	R376	Cohesin-nef66 (C1078)	0	4.2.13	3	R368	Cohesin-nef66 (C1078)	0	4.2.13	4	R386	Cohesin-nef66 (C1078)	5
4.29.13	1	R370	Cohesin-gag17	0	4.29.13	2	R376	Cohesin-gag17	0	4.2.13	3	R368	Cohesin-gag17	35	4.2.13	4	R386	Cohesin-gag17	10
4.29.13	1	R370	Cohesin-gag253	0	4.29.13	2	R376	Cohesin-gag253	0	4.2.13	3	R368	Cohesin-gag253	0	4.2.13	4	R386	Cohesin-gag253	10
4.29.13	1	R370	Cohesin–pol158	0	4.29.13	2	R376	Cohesin–pol158	0	4.2.13	3	R368	Cohesin–pol158	5	4.2.13	4	R386	Cohesin–pol158	0
4.29.13	1	R370	Cohesin	0	4.29.13	2	R376	Cohesin	0	4.2.13	3	R368	Cohesin	0	4.2.13	4	R386	Cohesin	5
4.29.13	1	R370	pha	1555	4.29.13	2	R376	pha	2565	4.2.13	3	R368	pha	2450	4.2.13	4	R386	pha	2910
4.29.13	1	R371	Non	0	4.29.13	2	R377	Non	0	4.2.13	3	R375	Non	0	4.2.13	4	R389	Non	0
4.29.13	1	R371	p24 pool 1	120	4.29.13	2	R377	p24 pool 1	0	4.2.13	3	R375	p24 pool 1	5	4.2.13	4	R389	p24 pool 1	10
4.29.13	1	R371	p24 pool 2	65	4.29.13	2	R377	p24 pool 2	45	4.2.13	3	R375	p24 pool 2	5	4.2.13	4	R389	p24 pool 2	5
4.29.13	1	R371	p24 pool 3	0	4.29.13	2	R377	p24 pool 3	0	4.2.13	3	R375	p24 pool 3	5	4.2.13	4	R389	p24 pool 3	40
4.29.13	1	R371	p24 pool 4	0	4.29.13	2	R377	p24 pool 4	0	4.2.13	3	R375	p24 pool 4	15	4.2.13	4	R389	p24 pool 4	15
4.29.13	1	R371	p24 pool 5	0	4.29.13	2	R377	p24 pool 5	0	4.2.13	3	R375	p24 pool 5	5	4.2.13	4	R389	p24 pool 5	15
4.29.13	1	R371	p2/p6/p7 pool 1	15	4.29.13	2	R377	p2/p6/p7 pool 1	0	4.2.13	3	R375	p2/p6/p7 pool 1	10	4.2.13	4	R389	p2/p6/p7 pool 1	5
4.29.13	1	R371	p2/p6/p7 pool 2	0	4.29.13	2	R377	p2/p6/p7 pool 2	0	4.2.13	3	R375	p2/p6/p7 pool 2	15	4.2.13	4	R389	p2/p6/p7 pool 2	10
4.29.13	1	R371	p2/p6/p7 pool 3	0	4.29.13	2	R377	p2/p6/p7 pool 3	0	4.2.13	3	R375	p2/p6/p7 pool 3	5	4.2.13	4	R389	p2/p6/p7 pool 3	5
4.29.13	1	R371	p17 pool 1	0	4.29.13	2	R377	p17 pool 1	0	4.2.13	3	R375	p17 pool 1	0	4.2.13	4	R389	p17 pool 1	0
4.29.13	1	R371	p17 pool 2	0	4.29.13	2	R377	p17 pool 2	0	4.2.13	3	R375	p17 pool 2	0	4.2.13	4	R389	p17 pool 2	10
4.29.13	1	R371	p17 pool 3	0	4.29.13	2	R377	p17 pool 3	0	4.2.13	3	R375	p17 pool 3	0	4.2.13	4	R389	p17 pool 3	10
4.29.13	1	R371	pol pool 1	0	4.29.13	2	R377	pol pool 1	0	4.2.13	3	R375	pol pool 1	0	4.2.13	4	R389	pol pool 1	5
4.29.13	1	R371	pol pool 2	20	4.29.13	2	R377	pol pool 2	10	4.2.13	3	R375	pol pool 2	80	4.2.13	4	R389	pol pool 2	90
4.29.13	1	R371	pol pool 3	0	4.29.13	2	R377	pol pool 3	5	4.2.13	3	R375	pol pool 3	0	4.2.13	4	R389	pol pool 3	10
4.29.13	1	R371	pol pool 4	0	4.29.13	2	R377	pol pool 4	0	4.2.13	3	R375	pol pool 4	5	4.2.13	4	R389	pol pool 4	15
4.29.13	1	R371	nef pool 1	0	4.29.13	2	R377	nef pool 1	0	4.2.13	3	R375	nef pool 1	5	4.2.13	4	R389	nef pool 1	5
4.29.13	1	R371	nef pool 2	0	4.29.13	2	R377	nef pool 2	0	4.2.13	3	R375	nef pool 2	50	4.2.13	4	R389	nef pool 2	10
4.29.13	1	R371	nef pool 3	0	4.29.13	2	R377	nef pool 3	70	4.2.13	3	R375	nef pool 3	0	4.2.13	4	R389	nef pool 3	15
4.29.13	1	R371	hIgG4-HIV5pep	0	4.29.13	2	R377	hIgG4-HIV5pep	0	4.2.13	3	R375	hIgG4-HIV5pep	30	4.2.13	4	R389	hIgG4-HIV5pep	10
4.29.13	1	R371	hIgG4	0	4.29.13	2	R377	hIgG4	0	4.2.13	3	R375	hIgG4	10	4.2.13	4	R389	hIgG4	10
4.29.13	1	R371	Cohesin-nef66 (C1077)	0	4.29.13	2	R377	Cohesin-nef66 (C1077)	0	4.2.13	3	R375	Cohesin-nef66 (C1077)	5	4.2.13	4	R389	Cohesin-nef66 (C1077)	10
4.29.13	1	R371	Cohesin-nef66 (C1078)	0	4.29.13	2	R377	Cohesin-nef66 (C1078)	0	4.2.13	3	R375	Cohesin-nef66 (C1078)	25	4.2.13	4	R389	Cohesin-nef66 (C1078)	15
4.29.13	1	R371	Cohesin-gag17	0	4.29.13	2	R377	Cohesin-gag17	35	4.2.13	3	R375	Cohesin-gag17	170	4.2.13	4	R389	Cohesin-gag17	90
4.29.13	1	R371	Cohesin-gag253	20	4.29.13	2	R377	Cohesin-gag253	0	4.2.13	3	R375	Cohesin-gag253	10	4.2.13	4	R389	Cohesin-gag253	15
4.29.13	1	R371	Cohesin–pol158	5	4.29.13	2	R377	Cohesin–pol158	20	4.2.13	3	R375	Cohesin–pol158	0	4.2.13	4	R389	Cohesin–pol158	5
4.29.13	1	R371	Cohesin	5	4.29.13	2	R377	Cohesin	0	4.2.13	3	R375	Cohesin	0	4.2.13	4	R389	Cohesin	0
4.29.13	1	R371	pha	3315	4.29.13	2	R377	pha	TNTC	4.2.13	3	R375	pha	2635	4.2.13	4	R389	pha	2145
4.29.13	1	R373	Non	0	4.29.13	2	R378	Non	0	4.2.13	3	R379	Non	0	4.2.13	4	R390	Non	0
4.29.13	1	R373	p24 pool 1	0	4.29.13	2	R378	p24 pool 1	0	4.2.13	3	R379	p24 pool 1	15	4.2.13	4	R390	p24 pool 1	0
4.29.13	1	R373	p24 pool 2	10	4.29.13	2	R378	p24 pool 2	20	4.2.13	3	R379	p24 pool 2	5	4.2.13	4	R390	p24 pool 2	5
4.29.13	1	R373	p24 pool 3	5	4.29.13	2	R378	p24 pool 3	0	4.2.13	3	R379	p24 pool 3	65	4.2.13	4	R390	p24 pool 3	10
4.29.13	1	R373	p24 pool 4	10	4.29.13	2	R378	p24 pool 4	0	4.2.13	3	R379	p24 pool 4	35	4.2.13	4	R390	p24 pool 4	10
4.29.13	1	R373	p24 pool 5	5	4.29.13	2	R378	p24 pool 5	0	4.2.13	3	R379	p24 pool 5	0	4.2.13	4	R390	p24 pool 5	5
4.29.13	1	R373	p2/p6/p7 pool 1	5	4.29.13	2	R378	p2/p6/p7 pool 1	15	4.2.13	3	R379	p2/p6/p7 pool 1	10	4.2.13	4	R390	p2/p6/p7 pool 1	5
4.29.13	1	R373	p2/p6/p7 pool 2	0	4.29.13	2	R378	p2/p6/p7 pool 2	0	4.2.13	3	R379	p2/p6/p7 pool 2	5	4.2.13	4	R390	p2/p6/p7 pool 2	0
4.29.13	1	R373	p2/p6/p7 pool 3	0	4.29.13	2	R378	p2/p6/p7 pool 3	0	4.2.13	3	R379	p2/p6/p7 pool 3	10	4.2.13	4	R390	p2/p6/p7 pool 3	5
4.29.13	1	R373	p17 pool 1	0	4.29.13	2	R378	p17 pool 1	0	4.2.13	3	R379	p17 pool 1	5	4.2.13	4	R390	p17 pool 1	0
4.29.13	1	R373	p17 pool 2	0	4.29.13	2	R378	p17 pool 2	0	4.2.13	3	R379	p17 pool 2	10	4.2.13	4	R390	p17 pool 2	0
4.29.13	1	R373	p17 pool 3	0	4.29.13	2	R378	p17 pool 3	0	4.2.13	3	R379	p17 pool 3	5	4.2.13	4	R390	p17 pool 3	0
4.29.13	1	R373	pol pool 1	5	4.29.13	2	R378	pol pool 1	0	4.2.13	3	R379	pol pool 1	10	4.2.13	4	R390	pol pool 1	0
4.29.13	1	R373	pol pool 2	10	4.29.13	2	R378	pol pool 2	20	4.2.13	3	R379	pol pool 2	320	4.2.13	4	R390	pol pool 2	10
4.29.13	1	R373	pol pool 3	5	4.29.13	2	R378	pol pool 3	0	4.2.13	3	R379	pol pool 3	15	4.2.13	4	R390	pol pool 3	0
4.29.13	1	R373	pol pool 4	5	4.29.13	2	R378	pol pool 4	0	4.2.13	3	R379	pol pool 4	10	4.2.13	4	R390	pol pool 4	0
4.29.13	1	R373	nef pool 1	0	4.29.13	2	R378	nef pool 1	10	4.2.13	3	R379	nef pool 1	5	4.2.13	4	R390	nef pool 1	5
4.29.13	1	R373	nef pool 2	0	4.29.13	2	R378	nef pool 2	0	4.2.13	3	R379	nef pool 2	5	4.2.13	4	R390	nef pool 2	5
4.29.13	1	R373	nef pool 3	5	4.29.13	2	R378	nef pool 3	0	4.2.13	3	R379	nef pool 3	10	4.2.13	4	R390	nef pool 3	5
4.29.13	1	R373	hIgG4-HIV5pep	0	4.29.13	2	R378	hIgG4-HIV5pep	0	4.2.13	3	R379	hIgG4-HIV5pep	10	4.2.13	4	R390	hIgG4-HIV5pep	15
4.29.13	1	R373	hIgG4	0	4.29.13	2	R378	hIgG4	0	4.2.13	3	R379	hIgG4	5	4.2.13	4	R390	hIgG4	10
4.29.13	1	R373	Cohesin-nef66 (C1077)	0	4.29.13	2	R378	Cohesin-nef66 (C1077)	10	4.2.13	3	R379	Cohesin-nef66 (C1077)	10	4.2.13	4	R390	Cohesin-nef66 (C1077)	10
4.29.13	1	R373	Cohesin-nef66 (C1078)	0	4.29.13	2	R378	Cohesin-nef66 (C1078)	0	4.2.13	3	R379	Cohesin-nef66 (C1078)	30	4.2.13	4	R390	Cohesin-nef66 (C1078)	30
4.29.13	1	R373	Cohesin-gag17	5	4.29.13	2	R378	Cohesin-gag17	0	4.2.13	3	R379	Cohesin-gag17	125	4.2.13	4	R390	Cohesin-gag17	10
4.29.13	1	R373	Cohesin-gag253	0	4.29.13	2	R378	Cohesin-gag253	20	4.2.13	3	R379	Cohesin-gag253	5	4.2.13	4	R390	Cohesin-gag253	10
4.29.13	1	R373	Cohesin–pol158	30	4.29.13	2	R378	Cohesin–pol158	20	4.2.13	3	R379	Cohesin–pol158	5	4.2.13	4	R390	Cohesin–pol158	5
4.29.13	1	R373	Cohesin	5	4.29.13	2	R378	Cohesin	0	4.2.13	3	R379	Cohesin	5	4.2.13	4	R390	Cohesin	0
4.29.13	1	R373	pha	2505	4.29.13	2	R378	pha	3225	4.2.13	3	R379	pha	2295	4.2.13	4	R390	pha	2570
4.29.13	1	R374	Non	0	4.29.13	2	R380	Non	0	4.2.13	3	R381	Non	0	4.2.13	4	R391	Non	0
4.29.13	1	R374	p24 pool 1	10	4.29.13	2	R380	p24 pool 1	10	4.2.13	3	R381	p24 pool 1	0	4.2.13	4	R391	p24 pool 1	10
4.29.13	1	R374	p24 pool 2	55	4.29.13	2	R380	p24 pool 2	20	4.2.13	3	R381	p24 pool 2	1	4.2.13	4	R391	p24 pool 2	15
4.29.13	1	R374	p24 pool 3	0	4.29.13	2	R380	p24 pool 3	0	4.2.13	3	R381	p24 pool 3	5	4.2.13	4	R391	p24 pool 3	35
4.29.13	1	R374	p24 pool 4	0	4.29.13	2	R380	p24 pool 4	5	4.2.13	3	R381	p24 pool 4	0	4.2.13	4	R391	p24 pool 4	15
4.29.13	1	R374	p24 pool 5	0	4.29.13	2	R380	p24 pool 5	5	4.2.13	3	R381	p24 pool 5	0	4.2.13	4	R391	p24 pool 5	20
4.29.13	1	R374	p2/p6/p7 pool 1	0	4.29.13	2	R380	p2/p6/p7 pool 1	5	4.2.13	3	R381	p2/p6/p7 pool 1	0	4.2.13	4	R391	p2/p6/p7 pool 1	5
4.29.13	1	R374	p2/p6/p7 pool 2	10	4.29.13	2	R380	p2/p6/p7 pool 2	0	4.2.13	3	R381	p2/p6/p7 pool 2	0	4.2.13	4	R391	p2/p6/p7 pool 2	5
4.29.13	1	R374	p2/p6/p7 pool 3	0	4.29.13	2	R380	p2/p6/p7 pool 3	5	4.2.13	3	R381	p2/p6/p7 pool 3	0	4.2.13	4	R391	p2/p6/p7 pool 3	0
4.29.13	1	R374	p17 pool 1	5	4.29.13	2	R380	p17 pool 1	10	4.2.13	3	R381	p17 pool 1	0	4.2.13	4	R391	p17 pool 1	30
4.29.13	1	R374	p17 pool 2	15	4.29.13	2	R380	p17 pool 2	15	4.2.13	3	R381	p17 pool 2	0	4.2.13	4	R391	p17 pool 2	15
4.29.13	1	R374	p17 pool 3	0	4.29.13	2	R380	p17 pool 3	5	4.2.13	3	R381	p17 pool 3	0	4.2.13	4	R391	p17 pool 3	5
4.29.13	1	R374	pol pool 1	0	4.29.13	2	R380	pol pool 1	5	4.2.13	3	R381	pol pool 1	0	4.2.13	4	R391	pol pool 1	15
4.29.13	1	R374	pol pool 2	10	4.29.13	2	R380	pol pool 2	10	4.2.13	3	R381	pol pool 2	270	4.2.13	4	R391	pol pool 2	25
4.29.13	1	R374	pol pool 3	5	4.29.13	2	R380	pol pool 3	5	4.2.13	3	R381	pol pool 3	0	4.2.13	4	R391	pol pool 3	15
4.29.13	1	R374	pol pool 4	5	4.29.13	2	R380	pol pool 4	5	4.2.13	3	R381	pol pool 4	0	4.2.13	4	R391	pol pool 4	5
4.29.13	1	R374	nef pool 1	0	4.29.13	2	R380	nef pool 1	10	4.2.13	3	R381	nef pool 1	0	4.2.13	4	R391	nef pool 1	10

4.29.13	1	R374	nef pool 2	0	4.29.13	2	R380	nef pool 2	10	4.2.13	3	R381	nef pool 2	0	4.2.13	4	R391	nef pool 2	25
4.29.13	1	R374	nef pool 3	30	4.29.13	2	R380	nef pool 3	10	4.2.13	3	R381	nef pool 3	5	4.2.13	4	R391	nef pool 3	30
4.29.13	1	R374	hIgG4-HIV5pep	0	4.29.13	2	R380	hIgG4-HIV5pep	0	4.2.13	3	R381	hIgG4-HIV5pep	5	4.2.13	4	R391	hIgG4-HIV5pep	45
4.29.13	1	R374	hIgG4	0	4.29.13	2	R380	hIgG4	0	4.2.13	3	R381	hIgG4	0	4.2.13	4	R391	hIgG4	10
4.29.13	1	R374	Cohesin-nef66 (C1077)	5	4.29.13	2	R380	Cohesin-nef66 (C1077)	10	4.2.13	3	R381	Cohesin-nef66 (C1077)	0	4.2.13	4	R391	Cohesin-nef66 (C1077)	5
4.29.13	1	R374	Cohesin-nef66 (C1078)	5	4.29.13	2	R380	Cohesin-nef66 (C1078)	10	4.2.13	3	R381	Cohesin-nef66 (C1078)	5	4.2.13	4	R391	Cohesin-nef66 (C1078)	95
4.29.13	1	R374	Cohesin-gag17	0	4.29.13	2	R380	Cohesin-gag17	5	4.2.13	3	R381	Cohesin-gag17	155	4.2.13	4	R391	Cohesin-gag17	45
4.29.13	1	R374	Cohesin-gag253	0	4.29.13	2	R380	Cohesin-gag253	10	4.2.13	3	R381	Cohesin-gag253	0	4.2.13	4	R391	Cohesin-gag253	5
4.29.13	1	R374	Cohesin–pol158	5	4.29.13	2	R380	Cohesin–pol158	5	4.2.13	3	R381	Cohesin–pol158	0	4.2.13	4	R391	Cohesin–pol158	0
4.29.13	1	R374	Cohesin	0	4.29.13	2	R380	Cohesin	5	4.2.13	3	R381	Cohesin	0	4.2.13	4	R391	Cohesin	0
4.29.13	1	R374	pha	2315	4.29.13	2	R380	pha	2970	4.2.13	3	R381	pha	2015	4.2.13	4	R391	pha	2805
4.29.13	1	R369	Non	0	4.29.13	2	R382	Non	0	4.2.13	3	R387	Non	0	4.2.13	4	R372	Non	0
4.29.13	1	R369	p24 pool 1	0	4.29.13	2	R382	p24 pool 1	0	4.2.13	3	R387	p24 pool 1	0	4.2.13	4	R372	p24 pool 1	5
4.29.13	1	R369	p24 pool 2	150	4.29.13	2	R382	p24 pool 2	0	4.2.13	3	R387	p24 pool 2	0	4.2.13	4	R372	p24 pool 2	5
4.29.13	1	R369	p24 pool 3	5	4.29.13	2	R382	p24 pool 3	0	4.2.13	3	R387	p24 pool 3	35	4.2.13	4	R372	p24 pool 3	35
4.29.13	1	R369	p24 pool 4	0	4.29.13	2	R382	p24 pool 4	0	4.2.13	3	R387	p24 pool 4	0	4.2.13	4	R372	p24 pool 4	15
4.29.13	1	R369	p24 pool 5	5	4.29.13	2	R382	p24 pool 5	0	4.2.13	3	R387	p24 pool 5	0	4.2.13	4	R372	p24 pool 5	5
4.29.13	1	R369	p2/p6/p7 pool 1	5	4.29.13	2	R382	p2/p6/p7 pool 1	0	4.2.13	3	R387	p2/p6/p7 pool 1	0	4.2.13	4	R372	p2/p6/p7 pool 1	10
4.29.13	1	R369	p2/p6/p7 pool 2	0	4.29.13	2	R382	p2/p6/p7 pool 2	0	4.2.13	3	R387	p2/p6/p7 pool 2	0	4.2.13	4	R372	p2/p6/p7 pool 2	0
4.29.13	1	R369	p2/p6/p7 pool 3	0	4.29.13	2	R382	p2/p6/p7 pool 3	0	4.2.13	3	R387	p2/p6/p7 pool 3	0	4.2.13	4	R372	p2/p6/p7 pool 3	5
4.29.13	1	R369	p17 pool 1	0	4.29.13	2	R382	p17 pool 1	0	4.2.13	3	R387	p17 pool 1	0	4.2.13	4	R372	p17 pool 1	15
4.29.13	1	R369	p17 pool 2	0	4.29.13	2	R382	p17 pool 2	0	4.2.13	3	R387	p17 pool 2	0	4.2.13	4	R372	p17 pool 2	0
4.29.13	1	R369	p17 pool 3	0	4.29.13	2	R382	p17 pool 3	0	4.2.13	3	R387	p17 pool 3	0	4.2.13	4	R372	p17 pool 3	10
4.29.13	1	R369	pol pool 1	0	4.29.13	2	R382	pol pool 1	0	4.2.13	3	R387	pol pool 1	0	4.2.13	4	R372	pol pool 1	0
4.29.13	1	R369	pol pool 2	15	4.29.13	2	R382	pol pool 2	0	4.2.13	3	R387	pol pool 2	0	4.2.13	4	R372	pol pool 2	35
4.29.13	1	R369	pol pool 3	5	4.29.13	2	R382	pol pool 3	25	4.2.13	3	R387	pol pool 3	0	4.2.13	4	R372	pol pool 3	5
4.29.13	1	R369	pol pool 4	0	4.29.13	2	R382	pol pool 4	0	4.2.13	3	R387	pol pool 4	0	4.2.13	4	R372	pol pool 4	5
4.29.13	1	R369	nef pool 1	0	4.29.13	2	R382	nef pool 1	0	4.2.13	3	R387	nef pool 1	0	4.2.13	4	R372	nef pool 1	15
4.29.13	1	R369	nef pool 2	0	4.29.13	2	R382	nef pool 2	0	4.2.13	3	R387	nef pool 2	5	4.2.13	4	R372	nef pool 2	10
4.29.13	1	R369	nef pool 3	40	4.29.13	2	R382	nef pool 3	20	4.2.13	3	R387	nef pool 3	0	4.2.13	4	R372	nef pool 3	10
4.29.13	1	R369	hIgG4-HIV5pep	0	4.29.13	2	R382	hIgG4-HIV5pep	0	4.2.13	3	R387	hIgG4-HIV5pep	0	4.2.13	4	R372	hIgG4-HIV5pep	10
4.29.13	1	R369	hIgG4	0	4.29.13	2	R382	hIgG4	0	4.2.13	3	R387	hIgG4	0	4.2.13	4	R372	hIgG4	10
4.29.13	1	R369	Cohesin-nef66 (C1077)	5	4.29.13	2	R382	Cohesin-nef66 (C1077)	0	4.2.13	3	R387	Cohesin-nef66 (C1077)	0	4.2.13	4	R372	Cohesin-nef66 (C1077)	10
4.29.13	1	R369	Cohesin-nef66 (C1078)	0	4.29.13	2	R382	Cohesin-nef66 (C1078)	0	4.2.13	3	R387	Cohesin-nef66 (C1078)	10	4.2.13	4	R372	Cohesin-nef66 (C1078)	25
4.29.13	1	R369	Cohesin-gag17	0	4.29.13	2	R382	Cohesin-gag17	0	4.2.13	3	R387	Cohesin-gag17	5	4.2.13	4	R372	Cohesin-gag17	30
4.29.13	1	R369	Cohesin-gag253	5	4.29.13	2	R382	Cohesin-gag253	0	4.2.13	3	R387	Cohesin-gag253	5	4.2.13	4	R372	Cohesin-gag253	5
4.29.13	1	R369	Cohesin–pol158	0	4.29.13	2	R382	Cohesin–pol158	5	4.2.13	3	R387	Cohesin–pol158	0	4.2.13	4	R372	Cohesin–pol158	5
4.29.13	1	R369	Cohesin	5	4.29.13	2	R382	Cohesin	0	4.2.13	3	R387	Cohesin	0	4.2.13	4	R372	Cohesin	0
4.29.13	1	R369	pha	2830	4.29.13	2	R382	pha	2435	4.2.13	3	R387	pha	2430	4.2.13	4	R372	pha	2590
4.29.13	1	R385	Non	0	4.29.13	2	R384	Non	0	4.2.13	3	R388	Non	0	4.2.13	4	R383	Non	0
4.29.13	1	R385	p24 pool 1	10	4.29.13	2	R384	p24 pool 1	0	4.2.13	3	R388	p24 pool 1	0	4.2.13	4	R383	p24 pool 1	5
4.29.13	1	R385	p24 pool 2	60	4.29.13	2	R384	p24 pool 2	10	4.2.13	3	R388	p24 pool 2	0	4.2.13	4	R383	p24 pool 2	10
4.29.13	1	R385	p24 pool 3	10	4.29.13	2	R384	p24 pool 3	0	4.2.13	3	R388	p24 pool 3	5	4.2.13	4	R383	p24 pool 3	25
4.29.13	1	R385	p24 pool 4	0	4.29.13	2	R384	p24 pool 4	0	4.2.13	3	R388	p24 pool 4	10	4.2.13	4	R383	p24 pool 4	10
4.29.13	1	R385	p24 pool 5	0	4.29.13	2	R384	p24 pool 5	0	4.2.13	3	R388	p24 pool 5	0	4.2.13	4	R383	p24 pool 5	5
4.29.13	1	R385	p2/p6/p7 pool 1	15	4.29.13	2	R384	p2/p6/p7 pool 1	0	4.2.13	3	R388	p2/p6/p7 pool 1	0	4.2.13	4	R383	p2/p6/p7 pool 1	0
4.29.13	1	R385	p2/p6/p7 pool 2	10	4.29.13	2	R384	p2/p6/p7 pool 2	0	4.2.13	3	R388	p2/p6/p7 pool 2	5	4.2.13	4	R383	p2/p6/p7 pool 2	0
4.29.13	1	R385	p2/p6/p7 pool 3	0	4.29.13	2	R384	p2/p6/p7 pool 3	0	4.2.13	3	R388	p2/p6/p7 pool 3	0	4.2.13	4	R383	p2/p6/p7 pool 3	0
4.29.13	1	R385	p17 pool 1	0	4.29.13	2	R384	p17 pool 1	0	4.2.13	3	R388	p17 pool 1	0	4.2.13	4	R383	p17 pool 1	15
4.29.13	1	R385	p17 pool 2	5	4.29.13	2	R384	p17 pool 2	0	4.2.13	3	R388	p17 pool 2	5	4.2.13	4	R383	p17 pool 2	5
4.29.13	1	R385	p17 pool 3	5	4.29.13	2	R384	p17 pool 3	0	4.2.13	3	R388	p17 pool 3	0	4.2.13	4	R383	p17 pool 3	5
4.29.13	1	R385	pol pool 1	0	4.29.13	2	R384	pol pool 1	0	4.2.13	3	R388	pol pool 1	0	4.2.13	4	R383	pol pool 1	5
4.29.13	1	R385	pol pool 2	0	4.29.13	2	R384	pol pool 2	0	4.2.13	3	R388	pol pool 2	15	4.2.13	4	R383	pol pool 2	55
4.29.13	1	R385	pol pool 3	5	4.29.13	2	R384	pol pool 3	0	4.2.13	3	R388	pol pool 3	0	4.2.13	4	R383	pol pool 3	5
4.29.13	1	R385	pol pool 4	0	4.29.13	2	R384	pol pool 4	0	4.2.13	3	R388	pol pool 4	5	4.2.13	4	R383	pol pool 4	10
4.29.13	1	R385	nef pool 1	10	4.29.13	2	R384	nef pool 1	0	4.2.13	3	R388	nef pool 1	0	4.2.13	4	R383	nef pool 1	10
4.29.13	1	R385	nef pool 2	0	4.29.13	2	R384	nef pool 2	0	4.2.13	3	R388	nef pool 2	15	4.2.13	4	R383	nef pool 2	30
4.29.13	1	R385	nef pool 3	15	4.29.13	2	R384	nef pool 3	0	4.2.13	3	R388	nef pool 3	0	4.2.13	4	R383	nef pool 3	0
4.29.13	1	R385	hIgG4-HIV5pep	0	4.29.13	2	R384	hIgG4-HIV5pep	0	4.2.13	3	R388	hIgG4-HIV5pep	30	4.2.13	4	R383	hIgG4-HIV5pep	5
4.29.13	1	R385	hIgG4	0	4.29.13	2	R384	hIgG4	0	4.2.13	3	R388	hIgG4	10	4.2.13	4	R383	hIgG4	10
4.29.13	1	R385	Cohesin-nef66 (C1077)	10	4.29.13	2	R384	Cohesin-nef66 (C1077)	0	4.2.13	3	R388	Cohesin-nef66 (C1077)	5	4.2.13	4	R383	Cohesin-nef66 (C1077)	15
4.29.13	1	R385	Cohesin-nef66 (C1078)	10	4.29.13	2	R384	Cohesin-nef66 (C1078)	0	4.2.13	3	R388	Cohesin-nef66 (C1078)	45	4.2.13	4	R383	Cohesin-nef66 (C1078)	20
4.29.13	1	R385	Cohesin-gag17	0	4.29.13	2	R384	Cohesin-gag17	0	4.2.13	3	R388	Cohesin-gag17	15	4.2.13	4	R383	Cohesin-gag17	25
4.29.13	1	R385	Cohesin-gag253	20	4.29.13	2	R384	Cohesin-gag253	0	4.2.13	3	R388	Cohesin-gag253	0	4.2.13	4	R383	Cohesin-gag253	5
4.29.13	1	R385	Cohesin–pol158	15	4.29.13	2	R384	Cohesin–pol158	0	4.2.13	3	R388	Cohesin–pol158	0	4.2.13	4	R383	Cohesin–pol158	5
4.29.13	1	R385	Cohesin	0	4.29.13	2	R384	Cohesin	0	4.2.13	3	R388	Cohesin	0	4.2.13	4	R383	Cohesin	0
4.29.13	1	R385	pha	2430	4.29.13	2	R384	pha	3260	4.2.13	3	R388	pha	2925	4.2.13	4	R383	pha	2590
5.13.13	1	R370	Non	0	5.13.13	2	R376	Non	0	5.28.13	3	R368	Non	0	5.28.13	4	R386	Non	0
5.13.13	1	R370	p24 pool 1	0	5.13.13	2	R376	p24 pool 1	80	5.28.13	3	R368	p24 pool 1	5	5.28.13	4	R386	p24 pool 1	0
5.13.13	1	R370	p24 pool 2	5	5.13.13	2	R376	p24 pool 2	5	5.28.13	3	R368	p24 pool 2	0	5.28.13	4	R386	p24 pool 2	10
5.13.13	1	R370	p24 pool 3	0	5.13.13	2	R376	p24 pool 3	0	5.28.13	3	R368	p24 pool 3	0	5.28.13	4	R386	p24 pool 3	40
5.13.13	1	R370	p24 pool 4	5	5.13.13	2	R376	p24 pool 4	0	5.28.13	3	R368	p24 pool 4	0	5.28.13	4	R386	p24 pool 4	20
5.13.13	1	R370	p24 pool 5	0	5.13.13	2	R376	p24 pool 5	0	5.28.13	3	R368	p24 pool 5	0	5.28.13	4	R386	p24 pool 5	0
5.13.13	1	R370	p2/p6/p7 pool 1	5	5.13.13	2	R376	p2/p6/p7 pool 1	5	5.28.13	3	R368	p2/p6/p7 pool 1	0	5.28.13	4	R386	p2/p6/p7 pool 1	5
5.13.13	1	R370	p2/p6/p7 pool 2	5	5.13.13	2	R376	p2/p6/p7 pool 2	0	5.28.13	3	R368	p2/p6/p7 pool 2	0	5.28.13	4	R386	p2/p6/p7 pool 2	5
5.13.13	1	R370	p2/p6/p7 pool 3	0	5.13.13	2	R376	p2/p6/p7 pool 3	5	5.28.13	3	R368	p2/p6/p7 pool 3	0	5.28.13	4	R386	p2/p6/p7 pool 3	5
5.13.13	1	R370	p17 pool 1	5	5.13.13	2	R376	p17 pool 1	0	5.28.13	3	R368	p17 pool 1	0	5.28.13	4	R386	p17 pool 1	5
5.13.13	1	R370	p17 pool 2	5	5.13.13	2	R376	p17 pool 2	0	5.28.13	3	R368	p17 pool 2	5	5.28.13	4	R386	p17 pool 2	5
5.13.13	1	R370	p17 pool 3	0	5.13.13	2	R376	p17 pool 3	0	5.28.13	3	R368	p17 pool 3	5	5.28.13	4	R386	p17 pool 3	0
5.13.13	1	R370	pol pool 1	0	5.13.13	2	R376	pol pool 1	0	5.28.13	3	R368	pol pool 1	0	5.28.13	4	R386	pol pool 1	0
5.13.13	1	R370	pol pool 2	0	5.13.13	2	R376	pol pool 2	5	5.28.13	3	R368	pol pool 2	0	5.28.13	4	R386	pol pool 2	80
5.13.13	1	R370	pol pool 3	5	5.13.13	2	R376	pol pool 3	0	5.28.13	3	R368	pol pool 3	45	5.28.13	4	R386	pol pool 3	5
5.13.13	1	R370	pol pool 4	5	5.13.13	2	R376	pol pool 4	5	5.28.13	3	R368	pol pool 4	0	5.28.13	4	R386	pol pool 4	5
5.13.13	1	R370	nef pool 1	0	5.13.13	2	R376	nef pool 1	0	5.28.13	3	R368	nef pool 1	0	5.28.13	4	R386	nef pool 1	5
5.13.13	1	R370	nef pool 2	0	5.13.13	2	R376	nef pool 2	5	5.28.13	3	R368	nef pool 2	65	5.28.13	4	R386	nef pool 2	5
5.13.13	1	R370	nef pool 3	5	5.13.13	2	R376	nef pool 3	5	5.28.13	3	R368	nef pool 3	0	5.28.13	4	R386	nef pool 3	0
5.13.13	1	R370	hIgG4-HIV5pep	0	5.13.13	2	R376	hIgG4-HIV5pep	0	5.28.13	3	R368	hIgG4-HIV5pep	0	5.28.13	4	R386	hIgG4-HIV5pep	0

5.13.13	1	R370	hIgG4	0	5.13.13	2	R376	hIgG4	0	5.28.13	3	R368	hIgG4	0	5.28.13	4	R386	hIgG4	0
5.13.13	1	R370	Cohesin-nef66 (C1077)	0	5.13.13	2	R376	Cohesin-nef66 (C1077)	5	5.28.13	3	R368	Cohesin-nef66 (C1077)	10	5.28.13	4	R386	Cohesin-nef66 (C1077)	0
5.13.13	1	R370	Cohesin-nef66 (C1078)	5	5.13.13	2	R376	Cohesin-nef66 (C1078)	0	5.28.13	3	R368	Cohesin-nef66 (C1078)	0	5.28.13	4	R386	Cohesin-nef66 (C1078)	0
5.13.13	1	R370	Cohesin-gag17	5	5.13.13	2	R376	Cohesin-gag17	5	5.28.13	3	R368	Cohesin-gag17	0	5.28.13	4	R386	Cohesin-gag17	5
5.13.13	1	R370	Cohesin-gag253	5	5.13.13	2	R376	Cohesin-gag253	10	5.28.13	3	R368	Cohesin-gag253	5	5.28.13	4	R386	Cohesin-gag253	5
5.13.13	1	R370	Cohesin–pol158	15	5.13.13	2	R376	Cohesin–pol158	5	5.28.13	3	R368	Cohesin–pol158	35	5.28.13	4	R386	Cohesin–pol158	10
5.13.13	1	R370	Cohesin	0	5.13.13	2	R376	Cohesin	0	5.28.13	3	R368	Cohesin	0	5.28.13	4	R386	Cohesin	0
5.13.13	1	R370	pha	2455	5.13.13	2	R376	pha	2670	5.28.13	3	R368	pha	2815	5.28.13	4	R386	pha	3605
5.13.13	1	R371	Non	0	5.13.13	2	R377	Non	0	5.28.13	3	R375	Non	0	5.28.13	4	R389	Non	0
5.13.13	1	R371	p24 pool 1	45	5.13.13	2	R377	p24 pool 1	0	5.28.13	3	R375	p24 pool 1	5	5.28.13	4	R389	p24 pool 1	25
5.13.13	1	R371	p24 pool 2	40	5.13.13	2	R377	p24 pool 2	5	5.28.13	3	R375	p24 pool 2	0	5.28.13	4	R389	p24 pool 2	0
5.13.13	1	R371	p24 pool 3	0	5.13.13	2	R377	p24 pool 3	0	5.28.13	3	R375	p24 pool 3	10	5.28.13	4	R389	p24 pool 3	110
5.13.13	1	R371	p24 pool 4	0	5.13.13	2	R377	p24 pool 4	0	5.28.13	3	R375	p24 pool 4	10	5.28.13	4	R389	p24 pool 4	20
5.13.13	1	R371	p24 pool 5	0	5.13.13	2	R377	p24 pool 5	5	5.28.13	3	R375	p24 pool 5	0	5.28.13	4	R389	p24 pool 5	15
5.13.13	1	R371	p2/p6/p7 pool 1	5	5.13.13	2	R377	p2/p6/p7 pool 1	10	5.28.13	3	R375	p2/p6/p7 pool 1	0	5.28.13	4	R389	p2/p6/p7 pool 1	5
5.13.13	1	R371	p2/p6/p7 pool 2	5	5.13.13	2	R377	p2/p6/p7 pool 2	0	5.28.13	3	R375	p2/p6/p7 pool 2	10	5.28.13	4	R389	p2/p6/p7 pool 2	0
5.13.13	1	R371	p2/p6/p7 pool 3	0	5.13.13	2	R377	p2/p6/p7 pool 3	0	5.28.13	3	R375	p2/p6/p7 pool 3	5	5.28.13	4	R389	p2/p6/p7 pool 3	0
5.13.13	1	R371	p17 pool 1	0	5.13.13	2	R377	p17 pool 1	0	5.28.13	3	R375	p17 pool 1	10	5.28.13	4	R389	p17 pool 1	10
5.13.13	1	R371	p17 pool 2	0	5.13.13	2	R377	p17 pool 2	0	5.28.13	3	R375	p17 pool 2	5	5.28.13	4	R389	p17 pool 2	0
5.13.13	1	R371	p17 pool 3	0	5.13.13	2	R377	p17 pool 3	0	5.28.13	3	R375	p17 pool 3	5	5.28.13	4	R389	p17 pool 3	0
5.13.13	1	R371	pol pool 1	0	5.13.13	2	R377	pol pool 1	0	5.28.13	3	R375	pol pool 1	5	5.28.13	4	R389	pol pool 1	0
5.13.13	1	R371	pol pool 2	0	5.13.13	2	R377	pol pool 2	10	5.28.13	3	R375	pol pool 2	260	5.28.13	4	R389	pol pool 2	460
5.13.13	1	R371	pol pool 3	5	5.13.13	2	R377	pol pool 3	0	5.28.13	3	R375	pol pool 3	15	5.28.13	4	R389	pol pool 3	25
5.13.13	1	R371	pol pool 4	5	5.13.13	2	R377	pol pool 4	0	5.28.13	3	R375	pol pool 4	10	5.28.13	4	R389	pol pool 4	0
5.13.13	1	R371	nef pool 1	5	5.13.13	2	R377	nef pool 1	0	5.28.13	3	R375	nef pool 1	0	5.28.13	4	R389	nef pool 1	0
5.13.13	1	R371	nef pool 2	0	5.13.13	2	R377	nef pool 2	0	5.28.13	3	R375	nef pool 2	105	5.28.13	4	R389	nef pool 2	30
5.13.13	1	R371	nef pool 3	20	5.13.13	2	R377	nef pool 3	0	5.28.13	3	R375	nef pool 3	15	5.28.13	4	R389	nef pool 3	10
5.13.13	1	R371	hIgG4-HIV5pep	0	5.13.13	2	R377	hIgG4-HIV5pep	0	5.28.13	3	R375	hIgG4-HIV5pep	0	5.28.13	4	R389	hIgG4-HIV5pep	0
5.13.13	1	R371	hIgG4	0	5.13.13	2	R377	hIgG4	0	5.28.13	3	R375	hIgG4	0	5.28.13	4	R389	hIgG4	0
5.13.13	1	R371	Cohesin-nef66 (C1077)	0	5.13.13	2	R377	Cohesin-nef66 (C1077)	0	5.28.13	3	R375	Cohesin-nef66 (C1077)	20	5.28.13	4	R389	Cohesin-nef66 (C1077)	0
5.13.13	1	R371	Cohesin-nef66 (C1078)	0	5.13.13	2	R377	Cohesin-nef66 (C1078)	0	5.28.13	3	R375	Cohesin-nef66 (C1078)	0	5.28.13	4	R389	Cohesin-nef66 (C1078)	5
5.13.13	1	R371	Cohesin-gag17	0	5.13.13	2	R377	Cohesin-gag17	0	5.28.13	3	R375	Cohesin-gag17	5	5.28.13	4	R389	Cohesin-gag17	0
5.13.13	1	R371	Cohesin-gag253	10	5.13.13	2	R377	Cohesin-gag253	10	5.28.13	3	R375	Cohesin-gag253	5	5.28.13	4	R389	Cohesin-gag253	10
5.13.13	1	R371	Cohesin–pol158	45	5.13.13	2	R377	Cohesin–pol158	5	5.28.13	3	R375	Cohesin–pol158	265	5.28.13	4	R389	Cohesin–pol158	360
5.13.13	1	R371	Cohesin	0	5.13.13	2	R377	Cohesin	0	5.28.13	3	R375	Cohesin	0	5.28.13	4	R389	Cohesin	0
5.13.13	1	R371	pha	3245	5.13.13	2	R377	pha	2480	5.28.13	3	R375	pha	2685	5.28.13	4	R389	pha	2800
5.13.13	1	R373	Non	0	5.13.13	2	R378	Non	0	5.28.13	3	R379	Non	0	5.28.13	4	R390	Non	0
5.13.13	1	R373	p24 pool 1	45	5.13.13	2	R378	p24 pool 1	0	5.28.13	3	R379	p24 pool 1	0	5.28.13	4	R390	p24 pool 1	0
5.13.13	1	R373	p24 pool 2	40	5.13.13	2	R378	p24 pool 2	0	5.28.13	3	R379	p24 pool 2	0	5.28.13	4	R390	p24 pool 2	10
5.13.13	1	R373	p24 pool 3	0	5.13.13	2	R378	p24 pool 3	0	5.28.13	3	R379	p24 pool 3	40	5.28.13	4	R390	p24 pool 3	25
5.13.13	1	R373	p24 pool 4	0	5.13.13	2	R378	p24 pool 4	0	5.28.13	3	R379	p24 pool 4	25	5.28.13	4	R390	p24 pool 4	0
5.13.13	1	R373	p24 pool 5	0	5.13.13	2	R378	p24 pool 5	0	5.28.13	3	R379	p24 pool 5	5	5.28.13	4	R390	p24 pool 5	0
5.13.13	1	R373	p2/p6/p7 pool 1	5	5.13.13	2	R378	p2/p6/p7 pool 1	0	5.28.13	3	R379	p2/p6/p7 pool 1	0	5.28.13	4	R390	p2/p6/p7 pool 1	0
5.13.13	1	R373	p2/p6/p7 pool 2	0	5.13.13	2	R378	p2/p6/p7 pool 2	0	5.28.13	3	R379	p2/p6/p7 pool 2	0	5.28.13	4	R390	p2/p6/p7 pool 2	0
5.13.13	1	R373	p2/p6/p7 pool 3	0	5.13.13	2	R378	p2/p6/p7 pool 3	0	5.28.13	3	R379	p2/p6/p7 pool 3	0	5.28.13	4	R390	p2/p6/p7 pool 3	0
5.13.13	1	R373	p17 pool 1	0	5.13.13	2	R378	p17 pool 1	0	5.28.13	3	R379	p17 pool 1	10	5.28.13	4	R390	p17 pool 1	0
5.13.13	1	R373	p17 pool 2	0	5.13.13	2	R378	p17 pool 2	0	5.28.13	3	R379	p17 pool 2	5	5.28.13	4	R390	p17 pool 2	0
5.13.13	1	R373	p17 pool 3	0	5.13.13	2	R378	p17 pool 3	0	5.28.13	3	R379	p17 pool 3	0	5.28.13	4	R390	p17 pool 3	0
5.13.13	1	R373	pol pool 1	0	5.13.13	2	R378	pol pool 1	0	5.28.13	3	R379	pol pool 1	0	5.28.13	4	R390	pol pool 1	0
5.13.13	1	R373	pol pool 2	0	5.13.13	2	R378	pol pool 2	0	5.28.13	3	R379	pol pool 2	495	5.28.13	4	R390	pol pool 2	55
5.13.13	1	R373	pol pool 3	5	5.13.13	2	R378	pol pool 3	0	5.28.13	3	R379	pol pool 3	0	5.28.13	4	R390	pol pool 3	0
5.13.13	1	R373	pol pool 4	0	5.13.13	2	R378	pol pool 4	0	5.28.13	3	R379	pol pool 4	0	5.28.13	4	R390	pol pool 4	0
5.13.13	1	R373	nef pool 1	0	5.13.13	2	R378	nef pool 1	0	5.28.13	3	R379	nef pool 1	0	5.28.13	4	R390	nef pool 1	0
5.13.13	1	R373	nef pool 2	0	5.13.13	2	R378	nef pool 2	0	5.28.13	3	R379	nef pool 2	20	5.28.13	4	R390	nef pool 2	5
5.13.13	1	R373	nef pool 3	20	5.13.13	2	R378	nef pool 3	0	5.28.13	3	R379	nef pool 3	5	5.28.13	4	R390	nef pool 3	0
5.13.13	1	R373	hIgG4-HIV5pep	0	5.13.13	2	R378	hIgG4-HIV5pep	0	5.28.13	3	R379	hIgG4-HIV5pep	0	5.28.13	4	R390	hIgG4-HIV5pep	0
5.13.13	1	R373	hIgG4	0	5.13.13	2	R378	hIgG4	0	5.28.13	3	R379	hIgG4	0	5.28.13	4	R390	hIgG4	0
5.13.13	1	R373	Cohesin-nef66 (C1077)	0	5.13.13	2	R378	Cohesin-nef66 (C1077)	0	5.28.13	3	R379	Cohesin-nef66 (C1077)	0	5.28.13	4	R390	Cohesin-nef66 (C1077)	0
5.13.13	1	R373	Cohesin-nef66 (C1078)	0	5.13.13	2	R378	Cohesin-nef66 (C1078)	0	5.28.13	3	R379	Cohesin-nef66 (C1078)	0	5.28.13	4	R390	Cohesin-nef66 (C1078)	0
5.13.13	1	R373	Cohesin-gag17	0	5.13.13	2	R378	Cohesin-gag17	0	5.28.13	3	R379	Cohesin-gag17	0	5.28.13	4	R390	Cohesin-gag17	0
5.13.13	1	R373	Cohesin-gag253	10	5.13.13	2	R378	Cohesin-gag253	35	5.28.13	3	R379	Cohesin-gag253	5	5.28.13	4	R390	Cohesin-gag253	0
5.13.13	1	R373	Cohesin–pol158	45	5.13.13	2	R378	Cohesin–pol158	60	5.28.13	3	R379	Cohesin–pol158	250	5.28.13	4	R390	Cohesin–pol158	20
5.13.13	1	R373	Cohesin	0	5.13.13	2	R378	Cohesin	0	5.28.13	3	R379	Cohesin	0	5.28.13	4	R390	Cohesin	0
5.13.13	1	R373	pha	3245	5.13.13	2	R378	pha	3090	5.28.13	3	R379	pha	2485	5.28.13	4	R390	pha	2700
5.13.13	1	R374	Non	0	5.13.13	2	R380	Non	0	5.28.13	3	R381	Non	0	5.28.13	4	R391	Non	0
5.13.13	1	R374	p24 pool 1	0	5.13.13	2	R380	p24 pool 1	80	5.28.13	3	R381	p24 pool 1	0	5.28.13	4	R391	p24 pool 1	5
5.13.13	1	R374	p24 pool 2	25	5.13.13	2	R380	p24 pool 2	5	5.28.13	3	R381	p24 pool 2	0	5.28.13	4	R391	p24 pool 2	15
5.13.13	1	R374	p24 pool 3	0	5.13.13	2	R380	p24 pool 3	0	5.28.13	3	R381	p24 pool 3	0	5.28.13	4	R391	p24 pool 3	30
5.13.13	1	R374	p24 pool 4	0	5.13.13	2	R380	p24 pool 4	0	5.28.13	3	R381	p24 pool 4	5	5.28.13	4	R391	p24 pool 4	5
5.13.13	1	R374	p24 pool 5	0	5.13.13	2	R380	p24 pool 5	0	5.28.13	3	R381	p24 pool 5	0	5.28.13	4	R391	p24 pool 5	10
5.13.13	1	R374	p2/p6/p7 pool 1	0	5.13.13	2	R380	p2/p6/p7 pool 1	5	5.28.13	3	R381	p2/p6/p7 pool 1	0	5.28.13	4	R391	p2/p6/p7 pool 1	5
5.13.13	1	R374	p2/p6/p7 pool 2	5	5.13.13	2	R380	p2/p6/p7 pool 2	0	5.28.13	3	R381	p2/p6/p7 pool 2	0	5.28.13	4	R391	p2/p6/p7 pool 2	5
5.13.13	1	R374	p2/p6/p7 pool 3	0	5.13.13	2	R380	p2/p6/p7 pool 3	5	5.28.13	3	R381	p2/p6/p7 pool 3	0	5.28.13	4	R391	p2/p6/p7 pool 3	10
5.13.13	1	R374	p17 pool 1	20	5.13.13	2	R380	p17 pool 1	0	5.28.13	3	R381	p17 pool 1	0	5.28.13	4	R391	p17 pool 1	25
5.13.13	1	R374	p17 pool 2	25	5.13.13	2	R380	p17 pool 2	0	5.28.13	3	R381	p17 pool 2	0	5.28.13	4	R391	p17 pool 2	20
5.13.13	1	R374	p17 pool 3	0	5.13.13	2	R380	p17 pool 3	0	5.28.13	3	R381	p17 pool 3	0	5.28.13	4	R391	p17 pool 3	10
5.13.13	1	R374	pol pool 1	0	5.13.13	2	R380	pol pool 1	0	5.28.13	3	R381	pol pool 1	0	5.28.13	4	R391	pol pool 1	5
5.13.13	1	R374	pol pool 2	0	5.13.13	2	R380	pol pool 2	5	5.28.13	3	R381	pol pool 2	150	5.28.13	4	R391	pol pool 2	10
5.13.13	1	R374	pol pool 3	0	5.13.13	2	R380	pol pool 3	0	5.28.13	3	R381	pol pool 3	0	5.28.13	4	R391	pol pool 3	0
5.13.13	1	R374	pol pool 4	0	5.13.13	2	R380	pol pool 4	0	5.28.13	3	R381	pol pool 4	0	5.28.13	4	R391	pol pool 4	10
5.13.13	1	R374	nef pool 1	0	5.13.13	2	R380	nef pool 1	0	5.28.13	3	R381	nef pool 1	0	5.28.13	4	R391	nef pool 1	5
5.13.13	1	R374	nef pool 2	0	5.13.13	2	R380	nef pool 2	0	5.28.13	3	R381	nef pool 2	0	5.28.13	4	R391	nef pool 2	25
5.13.13	1	R374	nef pool 3	20	5.13.13	2	R380	nef pool 3	5	5.28.13	3	R381	nef pool 3	0	5.28.13	4	R391	nef pool 3	5
5.13.13	1	R374	hIgG4-HIV5pep	0	5.13.13	2	R380	hIgG4-HIV5pep	0	5.28.13	3	R381	hIgG4-HIV5pep	0	5.28.13	4	R391	hIgG4-HIV5pep	0
5.13.13	1	R374	hIgG4	0	5.13.13	2	R380	hIgG4	0	5.28.13	3	R381	hIgG4	0	5.28.13	4	R391	hIgG4	0
5.13.13	1	R374	Cohesin-nef66 (C1077)	0	5.13.13	2	R380	Cohesin-nef66 (C1077)	5	5.28.13	3	R381	Cohesin-nef66 (C1077)	0	5.28.13	4	R391	Cohesin-nef66 (C1077)	5
5.13.13	1	R374	Cohesin-nef66 (C1078)	0	5.13.13	2	R380	Cohesin-nef66 (C1078)	0	5.28.13	3	R381	Cohesin-nef66 (C1078)	0	5.28.13	4	R391	Cohesin-nef66 (C1078)	5
5.13.13	1	R374	Cohesin-gag17	0	5.13.13	2	R380	Cohesin-gag17	5	5.28.13	3	R381	Cohesin-gag17	0	5.28.13	4	R391	Cohesin-gag17	5

5.13.13	1	R374	Cohesin-gag253	35	5.13.13	2	R380	Cohesin-gag253	10	5.28.13	3	R381	Cohesin-gag253	0	5.28.13	4	R391	Cohesin-gag253	10
5.13.13	1	R374	Cohesin–pol158	60	5.13.13	2	R380	Cohesin–pol158	5	5.28.13	3	R381	Cohesin–pol158	100	5.28.13	4	R391	Cohesin–pol158	40
5.13.13	1	R374	Cohesin	0	5.13.13	2	R380	Cohesin	0	5.28.13	3	R381	Cohesin	0	5.28.13	4	R391	Cohesin	20
5.13.13	1	R374	pha	3090	5.13.13	2	R380	pha	2670	5.28.13	3	R381	pha	2875	5.28.13	4	R391	pha	3040
5.13.13	1	R369	Non	0	5.13.13	2	R382	Non	0	5.28.13	3	R387	Non	0	5.28.13	4	R372	Non	0
5.13.13	1	R369	p24 pool 1	0	5.13.13	2	R382	p24 pool 1	5	5.28.13	3	R387	p24 pool 1	0	5.28.13	4	R372	p24 pool 1	0
5.13.13	1	R369	p24 pool 2	5	5.13.13	2	R382	p24 pool 2	15	5.28.13	3	R387	p24 pool 2	0	5.28.13	4	R372	p24 pool 2	5
5.13.13	1	R369	p24 pool 3	0	5.13.13	2	R382	p24 pool 3	5	5.28.13	3	R387	p24 pool 3	0	5.28.13	4	R372	p24 pool 3	20
5.13.13	1	R369	p24 pool 4	5	5.13.13	2	R382	p24 pool 4	0	5.28.13	3	R387	p24 pool 4	0	5.28.13	4	R372	p24 pool 4	10
5.13.13	1	R369	p24 pool 5	0	5.13.13	2	R382	p24 pool 5	0	5.28.13	3	R387	p24 pool 5	0	5.28.13	4	R372	p24 pool 5	0
5.13.13	1	R369	p2/p6/p7 pool 1	0	5.13.13	2	R382	p2/p6/p7 pool 1	0	5.28.13	3	R387	p2/p6/p7 pool 1	0	5.28.13	4	R372	p2/p6/p7 pool 1	0
5.13.13	1	R369	p2/p6/p7 pool 2	0	5.13.13	2	R382	p2/p6/p7 pool 2	5	5.28.13	3	R387	p2/p6/p7 pool 2	0	5.28.13	4	R372	p2/p6/p7 pool 2	0
5.13.13	1	R369	p2/p6/p7 pool 3	0	5.13.13	2	R382	p2/p6/p7 pool 3	10	5.28.13	3	R387	p2/p6/p7 pool 3	0	5.28.13	4	R372	p2/p6/p7 pool 3	0
5.13.13	1	R369	p17 pool 1	0	5.13.13	2	R382	p17 pool 1	5	5.28.13	3	R387	p17 pool 1	0	5.28.13	4	R372	p17 pool 1	10
5.13.13	1	R369	p17 pool 2	5	5.13.13	2	R382	p17 pool 2	10	5.28.13	3	R387	p17 pool 2	0	5.28.13	4	R372	p17 pool 2	0
5.13.13	1	R369	p17 pool 3	0	5.13.13	2	R382	p17 pool 3	5	5.28.13	3	R387	p17 pool 3	0	5.28.13	4	R372	p17 pool 3	0
5.13.13	1	R369	pol pool 1	0	5.13.13	2	R382	pol pool 1	5	5.28.13	3	R387	pol pool 1	0	5.28.13	4	R372	pol pool 1	0
5.13.13	1	R369	pol pool 2	0	5.13.13	2	R382	pol pool 2	10	5.28.13	3	R387	pol pool 2	0	5.28.13	4	R372	pol pool 2	105
5.13.13	1	R369	pol pool 3	0	5.13.13	2	R382	pol pool 3	80	5.28.13	3	R387	pol pool 3	0	5.28.13	4	R372	pol pool 3	0
5.13.13	1	R369	pol pool 4	5	5.13.13	2	R382	pol pool 4	0	5.28.13	3	R387	pol pool 4	0	5.28.13	4	R372	pol pool 4	0
5.13.13	1	R369	nef pool 1	0	5.13.13	2	R382	nef pool 1	5	5.28.13	3	R387	nef pool 1	0	5.28.13	4	R372	nef pool 1	0
5.13.13	1	R369	nef pool 2	0	5.13.13	2	R382	nef pool 2	10	5.28.13	3	R387	nef pool 2	0	5.28.13	4	R372	nef pool 2	5
5.13.13	1	R369	nef pool 3	5	5.13.13	2	R382	nef pool 3	25	5.28.13	3	R387	nef pool 3	0	5.28.13	4	R372	nef pool 3	0
5.13.13	1	R369	hIgG4-HIV5pep	0	5.13.13	2	R382	hIgG4-HIV5pep	0	5.28.13	3	R387	hIgG4-HIV5pep	0	5.28.13	4	R372	hIgG4-HIV5pep	0
5.13.13	1	R369	hIgG4	0	5.13.13	2	R382	hIgG4	0	5.28.13	3	R387	hIgG4	0	5.28.13	4	R372	hIgG4	0
5.13.13	1	R369	Cohesin-nef66 (C1077)	0	5.13.13	2	R382	Cohesin-nef66 (C1077)	25	5.28.13	3	R387	Cohesin-nef66 (C1077)	0	5.28.13	4	R372	Cohesin-nef66 (C1077)	0
5.13.13	1	R369	Cohesin-nef66 (C1078)	0	5.13.13	2	R382	Cohesin-nef66 (C1078)	10	5.28.13	3	R387	Cohesin-nef66 (C1078)	0	5.28.13	4	R372	Cohesin-nef66 (C1078)	0
5.13.13	1	R369	Cohesin-gag17	0	5.13.13	2	R382	Cohesin-gag17	5	5.28.13	3	R387	Cohesin-gag17	0	5.28.13	4	R372	Cohesin-gag17	0
5.13.13	1	R369	Cohesin-gag253	0	5.13.13	2	R382	Cohesin-gag253	25	5.28.13	3	R387	Cohesin-gag253	0	5.28.13	4	R372	Cohesin-gag253	20
5.13.13	1	R369	Cohesin–pol158	0	5.13.13	2	R382	Cohesin–pol158	35	5.28.13	3	R387	Cohesin–pol158	0	5.28.13	4	R372	Cohesin–pol158	30
5.13.13	1	R369	Cohesin	0	5.13.13	2	R382	Cohesin	5	5.28.13	3	R387	Cohesin	0	5.28.13	4	R372	Cohesin	0
5.13.13	1	R369	pha	2455	5.13.13	2	R382	pha	2920	5.28.13	3	R387	pha	0	5.28.13	4	R372	pha	2400
5.13.13	1	R385	Non	0	5.13.13	2	R384	Non	0	5.28.13	3	R388	Non	0	5.28.13	4	R383	Non	0
5.13.13	1	R385	p24 pool 1	0	5.13.13	2	R384	p24 pool 1	5	5.28.13	3	R388	p24 pool 1	0	5.28.13	4	R383	p24 pool 1	0
5.13.13	1	R385	p24 pool 2	5	5.13.13	2	R384	p24 pool 2	15	5.28.13	3	R388	p24 pool 2	0	5.28.13	4	R383	p24 pool 2	0
5.13.13	1	R385	p24 pool 3	0	5.13.13	2	R384	p24 pool 3	0	5.28.13	3	R388	p24 pool 3	0	5.28.13	4	R383	p24 pool 3	90
5.13.13	1	R385	p24 pool 4	0	5.13.13	2	R384	p24 pool 4	0	5.28.13	3	R388	p24 pool 4	10	5.28.13	4	R383	p24 pool 4	15
5.13.13	1	R385	p24 pool 5	5	5.13.13	2	R384	p24 pool 5	0	5.28.13	3	R388	p24 pool 5	0	5.28.13	4	R383	p24 pool 5	5
5.13.13	1	R385	p2/p6/p7 pool 1	10	5.13.13	2	R384	p2/p6/p7 pool 1	0	5.28.13	3	R388	p2/p6/p7 pool 1	0	5.28.13	4	R383	p2/p6/p7 pool 1	0
5.13.13	1	R385	p2/p6/p7 pool 2	0	5.13.13	2	R384	p2/p6/p7 pool 2	5	5.28.13	3	R388	p2/p6/p7 pool 2	0	5.28.13	4	R383	p2/p6/p7 pool 2	0
5.13.13	1	R385	p2/p6/p7 pool 3	0	5.13.13	2	R384	p2/p6/p7 pool 3	0	5.28.13	3	R388	p2/p6/p7 pool 3	0	5.28.13	4	R383	p2/p6/p7 pool 3	0
5.13.13	1	R385	p17 pool 1	0	5.13.13	2	R384	p17 pool 1	5	5.28.13	3	R388	p17 pool 1	0	5.28.13	4	R383	p17 pool 1	25
5.13.13	1	R385	p17 pool 2	0	5.13.13	2	R384	p17 pool 2	10	5.28.13	3	R388	p17 pool 2	0	5.28.13	4	R383	p17 pool 2	0
5.13.13	1	R385	p17 pool 3	0	5.13.13	2	R384	p17 pool 3	0	5.28.13	3	R388	p17 pool 3	0	5.28.13	4	R383	p17 pool 3	0
5.13.13	1	R385	pol pool 1	0	5.13.13	2	R384	pol pool 1	5	5.28.13	3	R388	pol pool 1	0	5.28.13	4	R383	pol pool 1	0
5.13.13	1	R385	pol pool 2	10	5.13.13	2	R384	pol pool 2	10	5.28.13	3	R388	pol pool 2	45	5.28.13	4	R383	pol pool 2	295
5.13.13	1	R385	pol pool 3	0	5.13.13	2	R384	pol pool 3	80	5.28.13	3	R388	pol pool 3	0	5.28.13	4	R383	pol pool 3	0
5.13.13	1	R385	pol pool 4	0	5.13.13	2	R384	pol pool 4	0	5.28.13	3	R388	pol pool 4	0	5.28.13	4	R383	pol pool 4	0
5.13.13	1	R385	nef pool 1	0	5.13.13	2	R384	nef pool 1	5	5.28.13	3	R388	nef pool 1	0	5.28.13	4	R383	nef pool 1	0
5.13.13	1	R385	nef pool 2	0	5.13.13	2	R384	nef pool 2	10	5.28.13	3	R388	nef pool 2	85	5.28.13	4	R383	nef pool 2	125
5.13.13	1	R385	nef pool 3	0	5.13.13	2	R384	nef pool 3	25	5.28.13	3	R388	nef pool 3	0	5.28.13	4	R383	nef pool 3	0
5.13.13	1	R385	hIgG4-HIV5pep	0	5.13.13	2	R384	hIgG4-HIV5pep	0	5.28.13	3	R388	hIgG4-HIV5pep	0	5.28.13	4	R383	hIgG4-HIV5pep	0
5.13.13	1	R385	hIgG4	0	5.13.13	2	R384	hIgG4	0	5.28.13	3	R388	hIgG4	0	5.28.13	4	R383	hIgG4	0
5.13.13	1	R385	Cohesin-nef66 (C1077)	0	5.13.13	2	R384	Cohesin-nef66 (C1077)	25	5.28.13	3	R388	Cohesin-nef66 (C1077)	35	5.28.13	4	R383	Cohesin-nef66 (C1077)	0
5.13.13	1	R385	Cohesin-nef66 (C1078)	0	5.13.13	2	R384	Cohesin-nef66 (C1078)	10	5.28.13	3	R388	Cohesin-nef66 (C1078)	15	5.28.13	4	R383	Cohesin-nef66 (C1078)	0
5.13.13	1	R385	Cohesin-gag17	0	5.13.13	2	R384	Cohesin-gag17	5	5.28.13	3	R388	Cohesin-gag17	0	5.28.13	4	R383	Cohesin-gag17	10
5.13.13	1	R385	Cohesin-gag253	10	5.13.13	2	R384	Cohesin-gag253	25	5.28.13	3	R388	Cohesin-gag253	15	5.28.13	4	R383	Cohesin-gag253	30
5.13.13	1	R385	Cohesin–pol158	5	5.13.13	2	R384	Cohesin–pol158	35	5.28.13	3	R388	Cohesin–pol158	55	5.28.13	4	R383	Cohesin–pol158	50
5.13.13	1	R385	Cohesin	0	5.13.13	2	R384	Cohesin	0	5.28.13	3	R388	Cohesin	0	5.28.13	4	R383	Cohesin	0
5.13.13	1	R385	pha	2480	5.13.13	2	R384	pha	2920	5.28.13	3	R388	pha	3095	5.28.13	4	R383	pha	2490

5.28.13	1	R370	Non	0	5.28.13	2	R376	Non	0	7.23.13	3	R368	Non	0	7.23.13	4	R386	Non	0
5.28.13	1	R370	p24 pool 1	0	5.28.13	2	R376	p24 pool 1	25	7.23.13	3	R368	p24 pool 1	5	7.23.13	4	R386	p24 pool 1	0
5.28.13	1	R370	p24 pool 2	0	5.28.13	2	R376	p24 pool 2	5	7.23.13	3	R368	p24 pool 2	0	7.23.13	4	R386	p24 pool 2	5
5.28.13	1	R370	p24 pool 3	5	5.28.13	2	R376	p24 pool 3	10	7.23.13	3	R368	p24 pool 3	5	7.23.13	4	R386	p24 pool 3	40
5.28.13	1	R370	p24 pool 4	5	5.28.13	2	R376	p24 pool 4	5	7.23.13	3	R368	p24 pool 4	5	7.23.13	4	R386	p24 pool 4	15
5.28.13	1	R370	p24 pool 5	0	5.28.13	2	R376	p24 pool 5	0	7.23.13	3	R368	p24 pool 5	10	7.23.13	4	R386	p24 pool 5	20
5.28.13	1	R370	p2/p6/p7 pool 1	5	5.28.13	2	R376	p2/p6/p7 pool 1	0	7.23.13	3	R368	p2/p6/p7 pool 1	5	7.23.13	4	R386	p2/p6/p7 pool 1	0
5.28.13	1	R370	p2/p6/p7 pool 2	5	5.28.13	2	R376	p2/p6/p7 pool 2	5	7.23.13	3	R368	p2/p6/p7 pool 2	10	7.23.13	4	R386	p2/p6/p7 pool 2	0
5.28.13	1	R370	p2/p6/p7 pool 3	0	5.28.13	2	R376	p2/p6/p7 pool 3	5	7.23.13	3	R368	p2/p6/p7 pool 3	5	7.23.13	4	R386	p2/p6/p7 pool 3	10
5.28.13	1	R370	p17 pool 1	0	5.28.13	2	R376	p17 pool 1	0	7.23.13	3	R368	p17 pool 1	5	7.23.13	4	R386	p17 pool 1	5
5.28.13	1	R370	p17 pool 2	0	5.28.13	2	R376	p17 pool 2	0	7.23.13	3	R368	p17 pool 2	15	7.23.13	4	R386	p17 pool 2	5
5.28.13	1	R370	p17 pool 3	0	5.28.13	2	R376	p17 pool 3	0	7.23.13	3	R368	p17 pool 3	10	7.23.13	4	R386	p17 pool 3	5
5.28.13	1	R370	pol pool 1	10	5.28.13	2	R376	pol pool 1	5	7.23.13	3	R368	pol pool 1	0	7.23.13	4	R386	pol pool 1	10
5.28.13	1	R370	pol pool 2	5	5.28.13	2	R376	pol pool 2	5	7.23.13	3	R368	pol pool 2	15	7.23.13	4	R386	pol pool 2	65
5.28.13	1	R370	pol pool 3	5	5.28.13	2	R376	pol pool 3	15	7.23.13	3	R368	pol pool 3	40	7.23.13	4	R386	pol pool 3	0
5.28.13	1	R370	pol pool 4	5	5.28.13	2	R376	pol pool 4	0	7.23.13	3	R368	pol pool 4	15	7.23.13	4	R386	pol pool 4	0
5.28.13	1	R370	nef pool 1	0	5.28.13	2	R376	nef pool 1	5	7.23.13	3	R368	nef pool 1	5	7.23.13	4	R386	nef pool 1	10
5.28.13	1	R370	nef pool 2	0	5.28.13	2	R376	nef pool 2	40	7.23.13	3	R368	nef pool 2	30	7.23.13	4	R386	nef pool 2	0
5.28.13	1	R370	nef pool 3	0	5.28.13	2	R376	nef pool 3	10	7.23.13	3	R368	nef pool 3	0	7.23.13	4	R386	nef pool 3	5
5.28.13	1	R370	hIgG4-HIV5pep	0	5.28.13	2	R376	hIgG4-HIV5pep	0	7.23.13	3	R368	hIgG4-HIV5pep	0	7.23.13	4	R386	hIgG4-HIV5pep	0
5.28.13	1	R370	hIgG4	0	5.28.13	2	R376	hIgG4	0	7.23.13	3	R368	hIgG4	0	7.23.13	4	R386	hIgG4	0
5.28.13	1	R370	Cohesin-nef66 (C1077)	0	5.28.13	2	R376	Cohesin-nef66 (C1077)	0	7.23.13	3	R368	Cohesin-nef66 (C1077)	15	7.23.13	4	R386	Cohesin-nef66 (C1077)	10
5.28.13	1	R370	Cohesin-nef66 (C1078)	5	5.28.13	2	R376	Cohesin-nef66 (C1078)	5	7.23.13	3	R368	Cohesin-nef66 (C1078)	10	7.23.13	4	R386	Cohesin-nef66 (C1078)	10
5.28.13	1	R370	Cohesin-gag17	0	5.28.13	2	R376	Cohesin-gag17	0	7.23.13	3	R368	Cohesin-gag17	5	7.23.13	4	R386	Cohesin-gag17	0
5.28.13	1	R370	Cohesin-gag253	0	5.28.13	2	R376	Cohesin-gag253	0	7.23.13	3	R368	Cohesin-gag253	35	7.23.13	4	R386	Cohesin-gag253	10
5.28.13	1	R370	Cohesin–pol158	10	5.28.13	2	R376	Cohesin–pol158	5	7.23.13	3	R368	Cohesin–pol158	40	7.23.13	4	R386	Cohesin–pol158	50
5.28.13	1	R370	Cohesin	0	5.28.13	2	R376	Cohesin	0	7.23.13	3	R368	Cohesin	5	7.23.13	4	R386	Cohesin	10

5.28.13	1	R370	pha	1560	5.28.13	2	R376	pha	2350	7.23.13	3	R368	pha	2935	7.23.13	4	R386	pha	3150
5.28.13	1	R371	Non	0	5.28.13	2	R377	Non	0	7.23.13	3	R375	Non	0	7.23.13	4	R389	Non	0
5.28.13	1	R371	p24 pool 1	25	5.28.13	2	R377	p24 pool 1	0	7.23.13	3	R375	p24 pool 1	0	7.23.13	4	R389	p24 pool 1	0
5.28.13	1	R371	p24 pool 2	10	5.28.13	2	R377	p24 pool 2	0	7.23.13	3	R375	p24 pool 2	0	7.23.13	4	R389	p24 pool 2	0
5.28.13	1	R371	p24 pool 3	0	5.28.13	2	R377	p24 pool 3	90	7.23.13	3	R375	p24 pool 3	10	7.23.13	4	R389	p24 pool 3	60
5.28.13	1	R371	p24 pool 4	45	5.28.13	2	R377	p24 pool 4	30	7.23.13	3	R375	p24 pool 4	5	7.23.13	4	R389	p24 pool 4	5
5.28.13	1	R371	p24 pool 5	0	5.28.13	2	R377	p24 pool 5	0	7.23.13	3	R375	p24 pool 5	0	7.23.13	4	R389	p24 pool 5	5
5.28.13	1	R371	p2/p6/p7 pool 1	5	5.28.13	2	R377	p2/p6/p7 pool 1	0	7.23.13	3	R375	p2/p6/p7 pool 1	5	7.23.13	4	R389	p2/p6/p7 pool 1	0
5.28.13	1	R371	p2/p6/p7 pool 2	0	5.28.13	2	R377	p2/p6/p7 pool 2	0	7.23.13	3	R375	p2/p6/p7 pool 2	0	7.23.13	4	R389	p2/p6/p7 pool 2	0
5.28.13	1	R371	p2/p6/p7 pool 3	0	5.28.13	2	R377	p2/p6/p7 pool 3	0	7.23.13	3	R375	p2/p6/p7 pool 3	5	7.23.13	4	R389	p2/p6/p7 pool 3	0
5.28.13	1	R371	p17 pool 1	0	5.28.13	2	R377	p17 pool 1	0	7.23.13	3	R375	p17 pool 1	5	7.23.13	4	R389	p17 pool 1	5
5.28.13	1	R371	p17 pool 2	0	5.28.13	2	R377	p17 pool 2	0	7.23.13	3	R375	p17 pool 2	0	7.23.13	4	R389	p17 pool 2	0
5.28.13	1	R371	p17 pool 3	0	5.28.13	2	R377	p17 pool 3	0	7.23.13	3	R375	p17 pool 3	5	7.23.13	4	R389	p17 pool 3	0
5.28.13	1	R371	pol pool 1	0	5.28.13	2	R377	pol pool 1	0	7.23.13	3	R375	pol pool 1	5	7.23.13	4	R389	pol pool 1	0
5.28.13	1	R371	pol pool 2	125	5.28.13	2	R377	pol pool 2	910	7.23.13	3	R375	pol pool 2	175	7.23.13	4	R389	pol pool 2	500
5.28.13	1	R371	pol pool 3	0	5.28.13	2	R377	pol pool 3	0	7.23.13	3	R375	pol pool 3	10	7.23.13	4	R389	pol pool 3	0
5.28.13	1	R371	pol pool 4	0	5.28.13	2	R377	pol pool 4	0	7.23.13	3	R375	pol pool 4	0	7.23.13	4	R389	pol pool 4	0
5.28.13	1	R371	nef pool 1	0	5.28.13	2	R377	nef pool 1	0	7.23.13	3	R375	nef pool 1	5	7.23.13	4	R389	nef pool 1	0
5.28.13	1	R371	nef pool 2	20	5.28.13	2	R377	nef pool 2	15	7.23.13	3	R375	nef pool 2	60	7.23.13	4	R389	nef pool 2	75
5.28.13	1	R371	nef pool 3	0	5.28.13	2	R377	nef pool 3	15	7.23.13	3	R375	nef pool 3	15	7.23.13	4	R389	nef pool 3	0
5.28.13	1	R371	hIgG4-HIV5pep	0	5.28.13	2	R377	hIgG4-HIV5pep	0	7.23.13	3	R375	hIgG4-HIV5pep	0	7.23.13	4	R389	hIgG4-HIV5pep	0
5.28.13	1	R371	hIgG4	0	5.28.13	2	R377	hIgG4	0	7.23.13	3	R375	hIgG4	0	7.23.13	4	R389	hIgG4	0
5.28.13	1	R371	Cohesin-nef66 (C1077)	0	5.28.13	2	R377	Cohesin-nef66 (C1077)	0	7.23.13	3	R375	Cohesin-nef66 (C1077)	5	7.23.13	4	R389	Cohesin-nef66 (C1077)	0
5.28.13	1	R371	Cohesin-nef66 (C1078)	0	5.28.13	2	R377	Cohesin-nef66 (C1078)	0	7.23.13	3	R375	Cohesin-nef66 (C1078)	0	7.23.13	4	R389	Cohesin-nef66 (C1078)	5
5.28.13	1	R371	Cohesin-gag17	0	5.28.13	2	R377	Cohesin-gag17	0	7.23.13	3	R375	Cohesin-gag17	0	7.23.13	4	R389	Cohesin-gag17	0
5.28.13	1	R371	Cohesin-gag253	0	5.28.13	2	R377	Cohesin-gag253	5	7.23.13	3	R375	Cohesin-gag253	5	7.23.13	4	R389	Cohesin-gag253	115
5.28.13	1	R371	Cohesin–pol158	100	5.28.13	2	R377	Cohesin–pol158	915	7.23.13	3	R375	Cohesin–pol158	115	7.23.13	4	R389	Cohesin–pol158	345
5.28.13	1	R371	Cohesin	0	5.28.13	2	R377	Cohesin	0	7.23.13	3	R375	Cohesin	5	7.23.13	4	R389	Cohesin	0
5.28.13	1	R371	pha	2805	5.28.13	2	R377	pha	3405	7.23.13	3	R375	pha	2340	7.23.13	4	R389	pha	2740
5.28.13	1	R373	Non	0	5.28.13	2	R378	Non	0	7.23.13	3	R379	Non	0	7.23.13	4	R390	Non	0
5.28.13	1	R373	p24 pool 1	5	5.28.13	2	R378	p24 pool 1	5	7.23.13	3	R379	p24 pool 1	5	7.23.13	4	R390	p24 pool 1	0
5.28.13	1	R373	p24 pool 2	0	5.28.13	2	R378	p24 pool 2	5	7.23.13	3	R379	p24 pool 2	5	7.23.13	4	R390	p24 pool 2	0
5.28.13	1	R373	p24 pool 3	5	5.28.13	2	R378	p24 pool 3	35	7.23.13	3	R379	p24 pool 3	45	7.23.13	4	R390	p24 pool 3	0
5.28.13	1	R373	p24 pool 4	0	5.28.13	2	R378	p24 pool 4	5	7.23.13	3	R379	p24 pool 4	25	7.23.13	4	R390	p24 pool 4	0
5.28.13	1	R373	p24 pool 5	0	5.28.13	2	R378	p24 pool 5	0	7.23.13	3	R379	p24 pool 5	0	7.23.13	4	R390	p24 pool 5	0
5.28.13	1	R373	p2/p6/p7 pool 1	0	5.28.13	2	R378	p2/p6/p7 pool 1	10	7.23.13	3	R379	p2/p6/p7 pool 1	0	7.23.13	4	R390	p2/p6/p7 pool 1	0
5.28.13	1	R373	p2/p6/p7 pool 2	0	5.28.13	2	R378	p2/p6/p7 pool 2	0	7.23.13	3	R379	p2/p6/p7 pool 2	0	7.23.13	4	R390	p2/p6/p7 pool 2	0
5.28.13	1	R373	p2/p6/p7 pool 3	0	5.28.13	2	R378	p2/p6/p7 pool 3	0	7.23.13	3	R379	p2/p6/p7 pool 3	0	7.23.13	4	R390	p2/p6/p7 pool 3	0
5.28.13	1	R373	p17 pool 1	0	5.28.13	2	R378	p17 pool 1	0	7.23.13	3	R379	p17 pool 1	5	7.23.13	4	R390	p17 pool 1	0
5.28.13	1	R373	p17 pool 2	0	5.28.13	2	R378	p17 pool 2	0	7.23.13	3	R379	p17 pool 2	0	7.23.13	4	R390	p17 pool 2	0
5.28.13	1	R373	p17 pool 3	0	5.28.13	2	R378	p17 pool 3	0	7.23.13	3	R379	p17 pool 3	5	7.23.13	4	R390	p17 pool 3	0
5.28.13	1	R373	pol pool 1	5	5.28.13	2	R378	pol pool 1	0	7.23.13	3	R379	pol pool 1	0	7.23.13	4	R390	pol pool 1	0
5.28.13	1	R373	pol pool 2	5	5.28.13	2	R378	pol pool 2	125	7.23.13	3	R379	pol pool 2	645	7.23.13	4	R390	pol pool 2	10
5.28.13	1	R373	pol pool 3	0	5.28.13	2	R378	pol pool 3	10	7.23.13	3	R379	pol pool 3	0	7.23.13	4	R390	pol pool 3	0
5.28.13	1	R373	pol pool 4	0	5.28.13	2	R378	pol pool 4	0	7.23.13	3	R379	pol pool 4	10	7.23.13	4	R390	pol pool 4	0
5.28.13	1	R373	nef pool 1	0	5.28.13	2	R378	nef pool 1	0	7.23.13	3	R379	nef pool 1	5	7.23.13	4	R390	nef pool 1	0
5.28.13	1	R373	nef pool 2	10	5.28.13	2	R378	nef pool 2	5	7.23.13	3	R379	nef pool 2	45	7.23.13	4	R390	nef pool 2	0
5.28.13	1	R373	nef pool 3	0	5.28.13	2	R378	nef pool 3	0	7.23.13	3	R379	nef pool 3	0	7.23.13	4	R390	nef pool 3	0
5.28.13	1	R373	hIgG4-HIV5pep	0	5.28.13	2	R378	hIgG4-HIV5pep	0	7.23.13	3	R379	hIgG4-HIV5pep	0	7.23.13	4	R390	hIgG4-HIV5pep	0
5.28.13	1	R373	hIgG4	0	5.28.13	2	R378	hIgG4	0	7.23.13	3	R379	hIgG4	0	7.23.13	4	R390	hIgG4	0
5.28.13	1	R373	Cohesin-nef66 (C1077)	0	5.28.13	2	R378	Cohesin-nef66 (C1077)	10	7.23.13	3	R379	Cohesin-nef66 (C1077)	0	7.23.13	4	R390	Cohesin-nef66 (C1077)	0
5.28.13	1	R373	Cohesin-nef66 (C1078)	0	5.28.13	2	R378	Cohesin-nef66 (C1078)	5	7.23.13	3	R379	Cohesin-nef66 (C1078)	45	7.23.13	4	R390	Cohesin-nef66 (C1078)	0
5.28.13	1	R373	Cohesin-gag17	5	5.28.13	2	R378	Cohesin-gag17	0	7.23.13	3	R379	Cohesin-gag17	0	7.23.13	4	R390	Cohesin-gag17	0
5.28.13	1	R373	Cohesin-gag253	5	5.28.13	2	R378	Cohesin-gag253	15	7.23.13	3	R379	Cohesin-gag253	40	7.23.13	4	R390	Cohesin-gag253	5
5.28.13	1	R373	Cohesin–pol158	5	5.28.13	2	R378	Cohesin–pol158	185	7.23.13	3	R379	Cohesin–pol158	370	7.23.13	4	R390	Cohesin–pol158	15
5.28.13	1	R373	Cohesin	0	5.28.13	2	R378	Cohesin	5	7.23.13	3	R379	Cohesin	5	7.23.13	4	R390	Cohesin	0
5.28.13	1	R373	pha	1710	5.28.13	2	R378	pha	2745	7.23.13	3	R379	pha	2685	7.23.13	4	R390	pha	2405
5.28.13	1	R374	Non	0	5.28.13	2	R380	Non	0	7.23.13	3	R381	Non	0	7.23.13	4	R391	Non	0
5.28.13	1	R374	p24 pool 1	5	5.28.13	2	R380	p24 pool 1	0	7.23.13	3	R381	p24 pool 1	10	7.23.13	4	R391	p24 pool 1	10
5.28.13	1	R374	p24 pool 2	15	5.28.13	2	R380	p24 pool 2	0	7.23.13	3	R381	p24 pool 2	5	7.23.13	4	R391	p24 pool 2	0
5.28.13	1	R374	p24 pool 3	210	5.28.13	2	R380	p24 pool 3	200	7.23.13	3	R381	p24 pool 3	15	7.23.13	4	R391	p24 pool 3	15
5.28.13	1	R374	p24 pool 4	60	5.28.13	2	R380	p24 pool 4	5	7.23.13	3	R381	p24 pool 4	15	7.23.13	4	R391	p24 pool 4	5
5.28.13	1	R374	p24 pool 5	5	5.28.13	2	R380	p24 pool 5	0	7.23.13	3	R381	p24 pool 5	5	7.23.13	4	R391	p24 pool 5	0
5.28.13	1	R374	p2/p6/p7 pool 1	5	5.28.13	2	R380	p2/p6/p7 pool 1	0	7.23.13	3	R381	p2/p6/p7 pool 1	0	7.23.13	4	R391	p2/p6/p7 pool 1	0
5.28.13	1	R374	p2/p6/p7 pool 2	5	5.28.13	2	R380	p2/p6/p7 pool 2	0	7.23.13	3	R381	p2/p6/p7 pool 2	0	7.23.13	4	R391	p2/p6/p7 pool 2	0
5.28.13	1	R374	p2/p6/p7 pool 3	0	5.28.13	2	R380	p2/p6/p7 pool 3	0	7.23.13	3	R381	p2/p6/p7 pool 3	0	7.23.13	4	R391	p2/p6/p7 pool 3	0
5.28.13	1	R374	p17 pool 1	0	5.28.13	2	R380	p17 pool 1	0	7.23.13	3	R381	p17 pool 1	5	7.23.13	4	R391	p17 pool 1	0
5.28.13	1	R374	p17 pool 2	5	5.28.13	2	R380	p17 pool 2	0	7.23.13	3	R381	p17 pool 2	0	7.23.13	4	R391	p17 pool 2	10
5.28.13	1	R374	p17 pool 3	5	5.28.13	2	R380	p17 pool 3	0	7.23.13	3	R381	p17 pool 3	0	7.23.13	4	R391	p17 pool 3	0
5.28.13	1	R374	pol pool 1	5	5.28.13	2	R380	pol pool 1	0	7.23.13	3	R381	pol pool 1	0	7.23.13	4	R391	pol pool 1	0
5.28.13	1	R374	pol pool 2	5	5.28.13	2	R380	pol pool 2	0	7.23.13	3	R381	pol pool 2	155	7.23.13	4	R391	pol pool 2	0
5.28.13	1	R374	pol pool 3	5	5.28.13	2	R380	pol pool 3	25	7.23.13	3	R381	pol pool 3	0	7.23.13	4	R391	pol pool 3	0
5.28.13	1	R374	pol pool 4	5	5.28.13	2	R380	pol pool 4	0	7.23.13	3	R381	pol pool 4	0	7.23.13	4	R391	pol pool 4	0
5.28.13	1	R374	nef pool 1	5	5.28.13	2	R380	nef pool 1	0	7.23.13	3	R381	nef pool 1	0	7.23.13	4	R391	nef pool 1	5
5.28.13	1	R374	nef pool 2	30	5.28.13	2	R380	nef pool 2	80	7.23.13	3	R381	nef pool 2	20	7.23.13	4	R391	nef pool 2	5
5.28.13	1	R374	nef pool 3	10	5.28.13	2	R380	nef pool 3	75	7.23.13	3	R381	nef pool 3	0	7.23.13	4	R391	nef pool 3	0
5.28.13	1	R374	hIgG4-HIV5pep	0	5.28.13	2	R380	hIgG4-HIV5pep	0	7.23.13	3	R381	hIgG4-HIV5pep	0	7.23.13	4	R391	hIgG4-HIV5pep	0
5.28.13	1	R374	hIgG4	0	5.28.13	2	R380	hIgG4	0	7.23.13	3	R381	hIgG4	0	7.23.13	4	R391	hIgG4	0
5.28.13	1	R374	Cohesin-nef66 (C1077)	0	5.28.13	2	R380	Cohesin-nef66 (C1077)	30	7.23.13	3	R381	Cohesin-nef66 (C1077)	10	7.23.13	4	R391	Cohesin-nef66 (C1077)	5
5.28.13	1	R374	Cohesin-nef66 (C1078)	5	5.28.13	2	R380	Cohesin-nef66 (C1078)	5	7.23.13	3	R381	Cohesin-nef66 (C1078)	10	7.23.13	4	R391	Cohesin-nef66 (C1078)	0
5.28.13	1	R374	Cohesin-gag17	5	5.28.13	2	R380	Cohesin-gag17	0	7.23.13	3	R381	Cohesin-gag17	0	7.23.13	4	R391	Cohesin-gag17	0
5.28.13	1	R374	Cohesin-gag253	105	5.28.13	2	R380	Cohesin-gag253	40	7.23.13	3	R381	Cohesin-gag253	40	7.23.13	4	R391	Cohesin-gag253	25
5.28.13	1	R374	Cohesin–pol158	20	5.28.13	2	R380	Cohesin–pol158	25	7.23.13	3	R381	Cohesin–pol158	70	7.23.13	4	R391	Cohesin–pol158	25
5.28.13	1	R374	Cohesin	5	5.28.13	2	R380	Cohesin	0	7.23.13	3	R381	Cohesin	0	7.23.13	4	R391	Cohesin	0
5.28.13	1	R374	pha	3225	5.28.13	2	R380	pha	2535	7.23.13	3	R381	pha	2705	7.23.13	4	R391	pha	2870
5.28.13	1	R369	Non	0	5.28.13	2	R382	Non	0	7.23.13	3	R387	Non	0	7.23.13	4	R372	Non	0
5.28.13	1	R369	p24 pool 1	0	5.28.13	2	R382	p24 pool 1	0	7.23.13	3	R387	p24 pool 1	0	7.23.13	4	R372	p24 pool 1	0
5.28.13	1	R369	p24 pool 2	50	5.28.13	2	R382	p24 pool 2	5	7.23.13	3	R387	p24 pool 2	0	7.23.13	4	R372	p24 pool 2	0

5.28.13	1	R369	p24 pool 3	375	5.28.13	2	R382	p24 pool 3	15	7.23.13	3	R387	p24 pool 3	0	7.23.13	4	R372	p24 pool 3	0
5.28.13	1	R369	p24 pool 4	15	5.28.13	2	R382	p24 pool 4	40	7.23.13	3	R387	p24 pool 4	0	7.23.13	4	R372	p24 pool 4	0
5.28.13	1	R369	p24 pool 5	0	5.28.13	2	R382	p24 pool 5	0	7.23.13	3	R387	p24 pool 5	0	7.23.13	4	R372	p24 pool 5	5
5.28.13	1	R369	p2/p6/p7 pool 1	0	5.28.13	2	R382	p2/p6/p7 pool 1	5	7.23.13	3	R387	p2/p6/p7 pool 1	0	7.23.13	4	R372	p2/p6/p7 pool 1	0
5.28.13	1	R369	p2/p6/p7 pool 2	0	5.28.13	2	R382	p2/p6/p7 pool 2	0	7.23.13	3	R387	p2/p6/p7 pool 2	0	7.23.13	4	R372	p2/p6/p7 pool 2	0
5.28.13	1	R369	p2/p6/p7 pool 3	0	5.28.13	2	R382	p2/p6/p7 pool 3	0	7.23.13	3	R387	p2/p6/p7 pool 3	0	7.23.13	4	R372	p2/p6/p7 pool 3	0
5.28.13	1	R369	p17 pool 1	0	5.28.13	2	R382	p17 pool 1	5	7.23.13	3	R387	p17 pool 1	0	7.23.13	4	R372	p17 pool 1	0
5.28.13	1	R369	p17 pool 2	0	5.28.13	2	R382	p17 pool 2	0	7.23.13	3	R387	p17 pool 2	0	7.23.13	4	R372	p17 pool 2	0
5.28.13	1	R369	p17 pool 3	0	5.28.13	2	R382	p17 pool 3	0	7.23.13	3	R387	p17 pool 3	0	7.23.13	4	R372	p17 pool 3	0
5.28.13	1	R369	pol pool 1	0	5.28.13	2	R382	pol pool 1	5	7.23.13	3	R387	pol pool 1	0	7.23.13	4	R372	pol pool 1	10
5.28.13	1	R369	pol pool 2	0	5.28.13	2	R382	pol pool 2	5	7.23.13	3	R387	pol pool 2	0	7.23.13	4	R372	pol pool 2	190
5.28.13	1	R369	pol pool 3	0	5.28.13	2	R382	pol pool 3	375	7.23.13	3	R387	pol pool 3	0	7.23.13	4	R372	pol pool 3	0
5.28.13	1	R369	pol pool 4	0	5.28.13	2	R382	pol pool 4	0	7.23.13	3	R387	pol pool 4	0	7.23.13	4	R372	pol pool 4	0
5.28.13	1	R369	nef pool 1	0	5.28.13	2	R382	nef pool 1	0	7.23.13	3	R387	nef pool 1	0	7.23.13	4	R372	nef pool 1	0
5.28.13	1	R369	nef pool 2	210	5.28.13	2	R382	nef pool 2	65	7.23.13	3	R387	nef pool 2	0	7.23.13	4	R372	nef pool 2	0
5.28.13	1	R369	nef pool 3	5	5.28.13	2	R382	nef pool 3	5	7.23.13	3	R387	nef pool 3	0	7.23.13	4	R372	nef pool 3	0
5.28.13	1	R369	hIgG4-HIV5pep	0	5.28.13	2	R382	hIgG4-HIV5pep	0	7.23.13	3	R387	hIgG4-HIV5pep	0	7.23.13	4	R372	hIgG4-HIV5pep	0
5.28.13	1	R369	hIgG4	0	5.28.13	2	R382	hIgG4	0	7.23.13	3	R387	hIgG4	0	7.23.13	4	R372	hIgG4	0
5.28.13	1	R369	Cohesin-nef66 (C1077)	20	5.28.13	2	R382	Cohesin-nef66 (C1077)	0	7.23.13	3	R387	Cohesin-nef66 (C1077)	0	7.23.13	4	R372	Cohesin-nef66 (C1077)	0
5.28.13	1	R369	Cohesin-nef66 (C1078)	0	5.28.13	2	R382	Cohesin-nef66 (C1078)	0	7.23.13	3	R387	Cohesin-nef66 (C1078)	0	7.23.13	4	R372	Cohesin-nef66 (C1078)	0
5.28.13	1	R369	Cohesin-gag17	0	5.28.13	2	R382	Cohesin-gag17	0	7.23.13	3	R387	Cohesin-gag17	0	7.23.13	4	R372	Cohesin-gag17	0
5.28.13	1	R369	Cohesin-gag253	110	5.28.13	2	R382	Cohesin-gag253	20	7.23.13	3	R387	Cohesin-gag253	0	7.23.13	4	R372	Cohesin-gag253	5
5.28.13	1	R369	Cohesin–pol158	5	5.28.13	2	R382	Cohesin–pol158	245	7.23.13	3	R387	Cohesin–pol158	0	7.23.13	4	R372	Cohesin–pol158	115
5.28.13	1	R369	Cohesin	0	5.28.13	2	R382	Cohesin	0	7.23.13	3	R387	Cohesin	0	7.23.13	4	R372	Cohesin	0
5.28.13	1	R369	pha	2635	5.28.13	2	R382	pha	2505	7.23.13	3	R387	pha	0	7.23.13	4	R372	pha	2230
5.28.13	1	R385	Non	0	5.28.13	2	R384	Non	0	7.23.13	3	R388	Non	0	7.23.13	4	R383	Non	0
5.28.13	1	R385	p24 pool 1	0	5.28.13	2	R384	p24 pool 1	0	7.23.13	3	R388	p24 pool 1	0	7.23.13	4	R383	p24 pool 1	5
5.28.13	1	R385	p24 pool 2	10	5.28.13	2	R384	p24 pool 2	10	7.23.13	3	R388	p24 pool 2	10	7.23.13	4	R383	p24 pool 2	5
5.28.13	1	R385	p24 pool 3	0	5.28.13	2	R384	p24 pool 3	70	7.23.13	3	R388	p24 pool 3	0	7.23.13	4	R383	p24 pool 3	120
5.28.13	1	R385	p24 pool 4	15	5.28.13	2	R384	p24 pool 4	80	7.23.13	3	R388	p24 pool 4	15	7.23.13	4	R383	p24 pool 4	10
5.28.13	1	R385	p24 pool 5	0	5.28.13	2	R384	p24 pool 5	5	7.23.13	3	R388	p24 pool 5	0	7.23.13	4	R383	p24 pool 5	5
5.28.13	1	R385	p2/p6/p7 pool 1	0	5.28.13	2	R384	p2/p6/p7 pool 1	0	7.23.13	3	R388	p2/p6/p7 pool 1	5	7.23.13	4	R383	p2/p6/p7 pool 1	0
5.28.13	1	R385	p2/p6/p7 pool 2	0	5.28.13	2	R384	p2/p6/p7 pool 2	0	7.23.13	3	R388	p2/p6/p7 pool 2	0	7.23.13	4	R383	p2/p6/p7 pool 2	10
5.28.13	1	R385	p2/p6/p7 pool 3	0	5.28.13	2	R384	p2/p6/p7 pool 3	5	7.23.13	3	R388	p2/p6/p7 pool 3	0	7.23.13	4	R383	p2/p6/p7 pool 3	10
5.28.13	1	R385	p17 pool 1	0	5.28.13	2	R384	p17 pool 1	0	7.23.13	3	R388	p17 pool 1	0	7.23.13	4	R383	p17 pool 1	15
5.28.13	1	R385	p17 pool 2	0	5.28.13	2	R384	p17 pool 2	5	7.23.13	3	R388	p17 pool 2	0	7.23.13	4	R383	p17 pool 2	15
5.28.13	1	R385	p17 pool 3	5	5.28.13	2	R384	p17 pool 3	0	7.23.13	3	R388	p17 pool 3	5	7.23.13	4	R383	p17 pool 3	10
5.28.13	1	R385	pol pool 1	0	5.28.13	2	R384	pol pool 1	5	7.23.13	3	R388	pol pool 1	0	7.23.13	4	R383	pol pool 1	10
5.28.13	1	R385	pol pool 2	25	5.28.13	2	R384	pol pool 2	0	7.23.13	3	R388	pol pool 2	30	7.23.13	4	R383	pol pool 2	480
5.28.13	1	R385	pol pool 3	0	5.28.13	2	R384	pol pool 3	75	7.23.13	3	R388	pol pool 3	0	7.23.13	4	R383	pol pool 3	5
5.28.13	1	R385	pol pool 4	0	5.28.13	2	R384	pol pool 4	0	7.23.13	3	R388	pol pool 4	5	7.23.13	4	R383	pol pool 4	5
5.28.13	1	R385	nef pool 1	0	5.28.13	2	R384	nef pool 1	10	7.23.13	3	R388	nef pool 1	0	7.23.13	4	R383	nef pool 1	5
5.28.13	1	R385	nef pool 2	5	5.28.13	2	R384	nef pool 2	20	7.23.13	3	R388	nef pool 2	45	7.23.13	4	R383	nef pool 2	140
5.28.13	1	R385	nef pool 3	10	5.28.13	2	R384	nef pool 3	30	7.23.13	3	R388	nef pool 3	0	7.23.13	4	R383	nef pool 3	5
5.28.13	1	R385	hIgG4-HIV5pep	0	5.28.13	2	R384	hIgG4-HIV5pep	0	7.23.13	3	R388	hIgG4-HIV5pep	0	7.23.13	4	R383	hIgG4-HIV5pep	0
5.28.13	1	R385	hIgG4	0	5.28.13	2	R384	hIgG4	0	7.23.13	3	R388	hIgG4	0	7.23.13	4	R383	hIgG4	0
5.28.13	1	R385	Cohesin-nef66 (C1077)	5	5.28.13	2	R384	Cohesin-nef66 (C1077)	0	7.23.13	3	R388	Cohesin-nef66 (C1077)	90	7.23.13	4	R383	Cohesin-nef66 (C1077)	25
5.28.13	1	R385	Cohesin-nef66 (C1078)	0	5.28.13	2	R384	Cohesin-nef66 (C1078)	0	7.23.13	3	R388	Cohesin-nef66 (C1078)	40	7.23.13	4	R383	Cohesin-nef66 (C1078)	5
5.28.13	1	R385	Cohesin-gag17	0	5.28.13	2	R384	Cohesin-gag17	5	7.23.13	3	R388	Cohesin-gag17	10	7.23.13	4	R383	Cohesin-gag17	15
5.28.13	1	R385	Cohesin-gag253	0	5.28.13	2	R384	Cohesin-gag253	5	7.23.13	3	R388	Cohesin-gag253	65	7.23.13	4	R383	Cohesin-gag253	45
5.28.13	1	R385	Cohesin–pol158	20	5.28.13	2	R384	Cohesin–pol158	45	7.23.13	3	R388	Cohesin–pol158	90	7.23.13	4	R383	Cohesin–pol158	250
5.28.13	1	R385	Cohesin	0	5.28.13	2	R384	Cohesin	0	7.23.13	3	R388	Cohesin	0	7.23.13	4	R383	Cohesin	5
5.28.13	1	R385	pha	2225	5.28.13	2	R384	pha	2970	7.23.13	3	R388	pha	2690	7.23.13	4	R383	pha	2650

6.24.13	1	R370	Non	0	6.24.13	2	R376	Non	0
6.24.13	1	R370	p24 pool 1	0	6.24.13	2	R376	p24 pool 1	45
6.24.13	1	R370	p24 pool 2	0	6.24.13	2	R376	p24 pool 2	0
6.24.13	1	R370	p24 pool 3	0	6.24.13	2	R376	p24 pool 3	45
6.24.13	1	R370	p24 pool 4	0	6.24.13	2	R376	p24 pool 4	5
6.24.13	1	R370	p24 pool 5	0	6.24.13	2	R376	p24 pool 5	0
6.24.13	1	R370	p2/p6/p7 pool 1	0	6.24.13	2	R376	p2/p6/p7 pool 1	0
6.24.13	1	R370	p2/p6/p7 pool 2	0	6.24.13	2	R376	p2/p6/p7 pool 2	0
6.24.13	1	R370	p2/p6/p7 pool 3	0	6.24.13	2	R376	p2/p6/p7 pool 3	0
6.24.13	1	R370	p17 pool 1	0	6.24.13	2	R376	p17 pool 1	5
6.24.13	1	R370	p17 pool 2	0	6.24.13	2	R376	p17 pool 2	10
6.24.13	1	R370	p17 pool 3	0	6.24.13	2	R376	p17 pool 3	0
6.24.13	1	R370	pol pool 1	0	6.24.13	2	R376	pol pool 1	0
6.24.13	1	R370	pol pool 2	15	6.24.13	2	R376	pol pool 2	25
6.24.13	1	R370	pol pool 3	35	6.24.13	2	R376	pol pool 3	90
6.24.13	1	R370	pol pool 4	0	6.24.13	2	R376	pol pool 4	0
6.24.13	1	R370	nef pool 1	0	6.24.13	2	R376	nef pool 1	0
6.24.13	1	R370	nef pool 2	5	6.24.13	2	R376	nef pool 2	165
6.24.13	1	R370	nef pool 3	0	6.24.13	2	R376	nef pool 3	105
6.24.13	1	R370	hIgG4-HIV5pep	0	6.24.13	2	R376	hIgG4-HIV5pep	0
6.24.13	1	R370	hIgG4	0	6.24.13	2	R376	hIgG4	0
6.24.13	1	R370	Cohesin-nef66 (C1077)	0	6.24.13	2	R376	Cohesin-nef66 (C1077)	35
6.24.13	1	R370	Cohesin-nef66 (C1078)	0	6.24.13	2	R376	Cohesin-nef66 (C1078)	20
6.24.13	1	R370	Cohesin-gag17	0	6.24.13	2	R376	Cohesin-gag17	0
6.24.13	1	R370	Cohesin-gag253	0	6.24.13	2	R376	Cohesin-gag253	10
6.24.13	1	R370	Cohesin–pol158	20	6.24.13	2	R376	Cohesin–pol158	10
6.24.13	1	R370	Cohesin	0	6.24.13	2	R376	Cohesin	5
6.24.13	1	R370	pha	1815	6.24.13	2	R376	pha	2700
6.24.13	1	R371	Non	0	6.24.13	2	R377	Non	0
6.24.13	1	R371	p24 pool 1	25	6.24.13	2	R377	p24 pool 1	5
6.24.13	1	R371	p24 pool 2	25	6.24.13	2	R377	p24 pool 2	10
6.24.13	1	R371	p24 pool 3	5	6.24.13	2	R377	p24 pool 3	145
6.24.13	1	R371	p24 pool 4	35	6.24.13	2	R377	p24 pool 4	50
6.24.13	1	R371	p24 pool 5	0	6.24.13	2	R377	p24 pool 5	0

6.24.13	1	R371	p2/p6/p7 pool 1	5	6.24.13	2	R377	p2/p6/p7 pool 1	5
6.24.13	1	R371	p2/p6/p7 pool 2	0	6.24.13	2	R377	p2/p6/p7 pool 2	0
6.24.13	1	R371	p2/p6/p7 pool 3	0	6.24.13	2	R377	p2/p6/p7 pool 3	0
6.24.13	1	R371	p17 pool 1	0	6.24.13	2	R377	p17 pool 1	5
6.24.13	1	R371	p17 pool 2	0	6.24.13	2	R377	p17 pool 2	10
6.24.13	1	R371	p17 pool 3	0	6.24.13	2	R377	p17 pool 3	0
6.24.13	1	R371	pol pool 1	0	6.24.13	2	R377	pol pool 1	0
6.24.13	1	R371	pol pool 2	455	6.24.13	2	R377	pol pool 2	1480
6.24.13	1	R371	pol pool 3	0	6.24.13	2	R377	pol pool 3	20
6.24.13	1	R371	pol pool 4	0	6.24.13	2	R377	pol pool 4	0
6.24.13	1	R371	nef pool 1	0	6.24.13	2	R377	nef pool 1	0
6.24.13	1	R371	nef pool 2	55	6.24.13	2	R377	nef pool 2	90
6.24.13	1	R371	nef pool 3	10	6.24.13	2	R377	nef pool 3	25
6.24.13	1	R371	hIgG4-HIV5pep	0	6.24.13	2	R377	hIgG4-HIV5pep	0
6.24.13	1	R371	hIgG4	0	6.24.13	2	R377	hIgG4	0
6.24.13	1	R371	Cohesin-nef66 (C1077)	10	6.24.13	2	R377	Cohesin-nef66 (C1077)	0
6.24.13	1	R371	Cohesin-nef66 (C1078)	5	6.24.13	2	R377	Cohesin-nef66 (C1078)	0
6.24.13	1	R371	Cohesin-gag17	0	6.24.13	2	R377	Cohesin-gag17	10
6.24.13	1	R371	Cohesin-gag253	15	6.24.13	2	R377	Cohesin-gag253	50
6.24.13	1	R371	Cohesin–pol158	280	6.24.13	2	R377	Cohesin–pol158	1255
6.24.13	1	R371	Cohesin	10	6.24.13	2	R377	Cohesin	5
6.24.13	1	R371	pha	2750	6.24.13	2	R377	pha	2990
6.24.13	1	R373	Non	0	6.24.13	2	R378	Non	0
6.24.13	1	R373	p24 pool 1	0	6.24.13	2	R378	p24 pool 1	20
6.24.13	1	R373	p24 pool 2	5	6.24.13	2	R378	p24 pool 2	15
6.24.13	1	R373	p24 pool 3	60	6.24.13	2	R378	p24 pool 3	145
6.24.13	1	R373	p24 pool 4	0	6.24.13	2	R378	p24 pool 4	15
6.24.13	1	R373	p24 pool 5	5	6.24.13	2	R378	p24 pool 5	5
6.24.13	1	R373	p2/p6/p7 pool 1	5	6.24.13	2	R378	p2/p6/p7 pool 1	5
6.24.13	1	R373	p2/p6/p7 pool 2	0	6.24.13	2	R378	p2/p6/p7 pool 2	5
6.24.13	1	R373	p2/p6/p7 pool 3	0	6.24.13	2	R378	p2/p6/p7 pool 3	0
6.24.13	1	R373	p17 pool 1	5	6.24.13	2	R378	p17 pool 1	0
6.24.13	1	R373	p17 pool 2	0	6.24.13	2	R378	p17 pool 2	10
6.24.13	1	R373	p17 pool 3	0	6.24.13	2	R378	p17 pool 3	10
6.24.13	1	R373	pol pool 1	0	6.24.13	2	R378	pol pool 1	0
6.24.13	1	R373	pol pool 2	0	6.24.13	2	R378	pol pool 2	800
6.24.13	1	R373	pol pool 3	0	6.24.13	2	R378	pol pool 3	30
6.24.13	1	R373	pol pool 4	0	6.24.13	2	R378	pol pool 4	5
6.24.13	1	R373	nef pool 1	0	6.24.13	2	R378	nef pool 1	5
6.24.13	1	R373	nef pool 2	55	6.24.13	2	R378	nef pool 2	65
6.24.13	1	R373	nef pool 3	10	6.24.13	2	R378	nef pool 3	5
6.24.13	1	R373	hIgG4-HIV5pep	0	6.24.13	2	R378	hIgG4-HIV5pep	0
6.24.13	1	R373	hIgG4	0	6.24.13	2	R378	hIgG4	0
6.24.13	1	R373	Cohesin-nef66 (C1077)	0	6.24.13	2	R378	Cohesin-nef66 (C1077)	40
6.24.13	1	R373	Cohesin-nef66 (C1078)	5	6.24.13	2	R378	Cohesin-nef66 (C1078)	20
6.24.13	1	R373	Cohesin-gag17	0	6.24.13	2	R378	Cohesin-gag17	20
6.24.13	1	R373	Cohesin-gag253	90	6.24.13	2	R378	Cohesin-gag253	45
6.24.13	1	R373	Cohesin–pol158	25	6.24.13	2	R378	Cohesin–pol158	700
6.24.13	1	R373	Cohesin	5	6.24.13	2	R378	Cohesin	0
6.24.13	1	R373	pha	2555	6.24.13	2	R378	pha	2975
6.24.13	1	R374	Non	0	6.24.13	2	R380	Non	0
6.24.13	1	R374	p24 pool 1	0	6.24.13	2	R380	p24 pool 1	10
6.24.13	1	R374	p24 pool 2	10	6.24.13	2	R380	p24 pool 2	15
6.24.13	1	R374	p24 pool 3	285	6.24.13	2	R380	p24 pool 3	490
6.24.13	1	R374	p24 pool 4	40	6.24.13	2	R380	p24 pool 4	20
6.24.13	1	R374	p24 pool 5	0	6.24.13	2	R380	p24 pool 5	5
6.24.13	1	R374	p2/p6/p7 pool 1	0	6.24.13	2	R380	p2/p6/p7 pool 1	15
6.24.13	1	R374	p2/p6/p7 pool 2	0	6.24.13	2	R380	p2/p6/p7 pool 2	5
6.24.13	1	R374	p2/p6/p7 pool 3	0	6.24.13	2	R380	p2/p6/p7 pool 3	15
6.24.13	1	R374	p17 pool 1	0	6.24.13	2	R380	p17 pool 1	10
6.24.13	1	R374	p17 pool 2	0	6.24.13	2	R380	p17 pool 2	10
6.24.13	1	R374	p17 pool 3	0	6.24.13	2	R380	p17 pool 3	5
6.24.13	1	R374	pol pool 1	0	6.24.13	2	R380	pol pool 1	5
6.24.13	1	R374	pol pool 2	5	6.24.13	2	R380	pol pool 2	70
6.24.13	1	R374	pol pool 3	0	6.24.13	2	R380	pol pool 3	50
6.24.13	1	R374	pol pool 4	0	6.24.13	2	R380	pol pool 4	10
6.24.13	1	R374	nef pool 1	0	6.24.13	2	R380	nef pool 1	0
6.24.13	1	R374	nef pool 2	45	6.24.13	2	R380	nef pool 2	155
6.24.13	1	R374	nef pool 3	5	6.24.13	2	R380	nef pool 3	130
6.24.13	1	R374	hIgG4-HIV5pep	0	6.24.13	2	R380	hIgG4-HIV5pep	0
6.24.13	1	R374	hIgG4	0	6.24.13	2	R380	hIgG4	0
6.24.13	1	R374	Cohesin-nef66 (C1077)	0	6.24.13	2	R380	Cohesin-nef66 (C1077)	15
6.24.13	1	R374	Cohesin-nef66 (C1078)	0	6.24.13	2	R380	Cohesin-nef66 (C1078)	25
6.24.13	1	R374	Cohesin-gag17	0	6.24.13	2	R380	Cohesin-gag17	0
6.24.13	1	R374	Cohesin-gag253	145	6.24.13	2	R380	Cohesin-gag253	225
6.24.13	1	R374	Cohesin–pol158	5	6.24.13	2	R380	Cohesin–pol158	20
6.24.13	1	R374	Cohesin	0	6.24.13	2	R380	Cohesin	0
6.24.13	1	R374	pha	2840	6.24.13	2	R380	pha	2160
6.24.13	1	R369	Non	0	6.24.13	2	R382	Non	0
6.24.13	1	R369	p24 pool 1	5	6.24.13	2	R382	p24 pool 1	10
6.24.13	1	R369	p24 pool 2	100	6.24.13	2	R382	p24 pool 2	15
6.24.13	1	R369	p24 pool 3	765	6.24.13	2	R382	p24 pool 3	130
6.24.13	1	R369	p24 pool 4	15	6.24.13	2	R382	p24 pool 4	130
6.24.13	1	R369	p24 pool 5	0	6.24.13	2	R382	p24 pool 5	0
6.24.13	1	R369	p2/p6/p7 pool 1	5	6.24.13	2	R382	p2/p6/p7 pool 1	5
6.24.13	1	R369	p2/p6/p7 pool 2	15	6.24.13	2	R382	p2/p6/p7 pool 2	0
6.24.13	1	R369	p2/p6/p7 pool 3	10	6.24.13	2	R382	p2/p6/p7 pool 3	0
6.24.13	1	R369	p17 pool 1	30	6.24.13	2	R382	p17 pool 1	5

6.24.13	1	R369	p17 pool 2	0
6.24.13	1	R369	p17 pool 3	10
6.24.13	1	R369	pol pool 1	0
6.24.13	1	R369	pol pool 2	25
6.24.13	1	R369	pol pool 3	5
6.24.13	1	R369	pol pool 4	5
6.24.13	1	R369	nef pool 1	0
6.24.13	1	R369	nef pool 2	770
6.24.13	1	R369	nef pool 3	30
6.24.13	1	R369	hIgG4-HIV5pep	0
6.24.13	1	R369	hIgG4	0
6.24.13	1	R369	Cohesin-nef66 (C1077)	180
6.24.13	1	R369	Cohesin-nef66 (C1078)	0
6.24.13	1	R369	Cohesin-gag17	0
6.24.13	1	R369	Cohesin-gag253	540
6.24.13	1	R369	Cohesin–pol158	20
6.24.13	1	R369	Cohesin	0
6.24.13	1	R369	pha	3365
6.24.13	1	R385	Non	0
6.24.13	1	R385	p24 pool 1	0
6.24.13	1	R385	p24 pool 2	15
6.24.13	1	R385	p24 pool 3	20
6.24.13	1	R385	p24 pool 4	130
6.24.13	1	R385	p24 pool 5	10
6.24.13	1	R385	p2/p6/p7 pool 1	5
6.24.13	1	R385	p2/p6/p7 pool 2	15
6.24.13	1	R385	p2/p6/p7 pool 3	0
6.24.13	1	R385	p17 pool 1	10
6.24.13	1	R385	p17 pool 2	5
6.24.13	1	R385	p17 pool 3	0
6.24.13	1	R385	pol pool 1	0
6.24.13	1	R385	pol pool 2	460
6.24.13	1	R385	pol pool 3	0
6.24.13	1	R385	pol pool 4	0
6.24.13	1	R385	nef pool 1	0
6.24.13	1	R385	nef pool 2	50
6.24.13	1	R385	nef pool 3	0
6.24.13	1	R385	hIgG4-HIV5pep	0
6.24.13	1	R385	hIgG4	0
6.24.13	1	R385	Cohesin-nef66 (C1077)	15
6.24.13	1	R385	Cohesin-nef66 (C1078)	5
6.24.13	1	R385	Cohesin-gag17	5
6.24.13	1	R385	Cohesin-gag253	25
6.24.13	1	R385	Cohesin–pol158	120
6.24.13	1	R385	Cohesin	5
6.24.13	1	R385	pha	1890

6.24.13	2	R382	p17 pool 2	5
6.24.13	2	R382	p17 pool 3	0
6.24.13	2	R382	pol pool 1	0
6.24.13	2	R382	pol pool 2	60
6.24.13	2	R382	pol pool 3	520
6.24.13	2	R382	pol pool 4	0
6.24.13	2	R382	nef pool 1	0
6.24.13	2	R382	nef pool 2	175
6.24.13	2	R382	nef pool 3	15
6.24.13	2	R382	hIgG4-HIV5pep	0
6.24.13	2	R382	hIgG4	0
6.24.13	2	R382	Cohesin-nef66 (C1077)	20
6.24.13	2	R382	Cohesin-nef66 (C1078)	0
6.24.13	2	R382	Cohesin-gag17	5
6.24.13	2	R382	Cohesin-gag253	55
6.24.13	2	R382	Cohesin–pol158	360
6.24.13	2	R382	Cohesin	0
6.24.13	2	R382	pha	3010
6.24.13	2	R384	Non	0
6.24.13	2	R384	p24 pool 1	15
6.24.13	2	R384	p24 pool 2	25
6.24.13	2	R384	p24 pool 3	120
6.24.13	2	R384	p24 pool 4	80
6.24.13	2	R384	p24 pool 5	5
6.24.13	2	R384	p2/p6/p7 pool 1	0
6.24.13	2	R384	p2/p6/p7 pool 2	10
6.24.13	2	R384	p2/p6/p7 pool 3	15
6.24.13	2	R384	p17 pool 1	5
6.24.13	2	R384	p17 pool 2	10
6.24.13	2	R384	p17 pool 3	0
6.24.13	2	R384	pol pool 1	0
6.24.13	2	R384	pol pool 2	5
6.24.13	2	R384	pol pool 3	90
6.24.13	2	R384	pol pool 4	0
6.24.13	2	R384	nef pool 1	0
6.24.13	2	R384	nef pool 2	90
6.24.13	2	R384	nef pool 3	15
6.24.13	2	R384	hIgG4-HIV5pep	0
6.24.13	2	R384	hIgG4	0
6.24.13	2	R384	Cohesin-nef66 (C1077)	5
6.24.13	2	R384	Cohesin-nef66 (C1078)	10
6.24.13	2	R384	Cohesin-gag17	5
6.24.13	2	R384	Cohesin-gag253	35
6.24.13	2	R384	Cohesin–pol158	25
6.24.13	2	R384	Cohesin	0
6.24.13	2	R384	pha	2430
